# Cognitive functions and underlying parameters of human brain physiology are associated with chronotype

**DOI:** 10.1038/s41467-021-24885-0

**Published:** 2021-08-03

**Authors:** Mohammad Ali Salehinejad, Miles Wischnewski, Elham Ghanavati, Mohsen Mosayebi-Samani, Min-Fang Kuo, Michael A. Nitsche

**Affiliations:** 1 Department of Psychology and Neurosciences, Leibniz Research Centre for Working Environment and Human Factors, Dortmund, Germany; 2 International Graduate School of Neuroscience, Ruhr-University Bochum, Bochum, Germany; 3 Donders Institute for Brain, Cognition, and Behaviour, Radboud University Nijmegen, Nijmegen, The Netherlands; 4 Department of Psychology, Ruhr-University Bochum, Bochum, Germany; 5 Department of Neurology, University Medical Hospital Bergmannsheil, Bochum, Germany

**Keywords:** Circadian rhythms and sleep, Cognitive neuroscience, Learning and memory, Neuronal physiology, Human behaviour

## Abstract

Circadian rhythms have natural relative variations among humans known as chronotype. Chronotype or being a morning or evening person, has a specific physiological, behavioural, and also genetic manifestation. Whether and how chronotype modulates human brain physiology and cognition is, however, not well understood. Here we examine how cortical excitability, neuroplasticity, and cognition are associated with chronotype in early and late chronotype individuals. We monitor motor cortical excitability, brain stimulation-induced neuroplasticity, and examine motor learning and cognitive functions at circadian-preferred and non-preferred times of day in 32 individuals. Motor learning and cognitive performance (working memory, and attention) along with their electrophysiological components are significantly enhanced at the circadian-preferred, compared to the non-preferred time. This outperformance is associated with enhanced cortical excitability (prominent cortical facilitation, diminished cortical inhibition), and long-term potentiation/depression-like plasticity. Our data show convergent findings of how chronotype can modulate human brain functions from basic physiological mechanisms to behaviour and higher-order cognition.

## Abstract (web summary)

How being a “morning person” or “evening person” affects human cognition and brain physiology is not well understood. Here the authors show evidence of an association of chronotype with cognitive functions and related physiological parameters.

## Introduction

Circadian rhythms are basic, daily cyclical processes that affect a wide range of physiological and behavioral manifestations and show significant variations in the human population^1^. Circadian *preference* or “chronotype” describes an individual’s physical and behavioral preference for earlier or later sleep timing as a result of coupling between internal circadian cycles and the need for sleep^2^. The modulatory effects of circadian rhythms on basic physiological processes (e.g., cell cycle, body temperature, sleep–wake cycle) in living organisms are well-established. Research performed during the last two decades has been primarily dedicated to molecular and cellular links between circadian rhythms and respective physiological processes in mammals, including humans^3,4^. In recent years, respective research interest was broadened to fields, such as genetics^2^, brain physiology^5^, and cognition^6,7^.

This renewed interest in the “time-of-day” and “circadian rhythm” effects on human brain physiology and cognition is fueled by technological advances in human cognitive neuroscience^5,6^. Given that the modern lifestyle is becoming less dependent on the 24-h day–night cycle, an increased understanding of how the human brain and cognitive functions are influenced by chronotype and optimal time-of-day, has broad implications for human well-being, public health, working environments, school performance^8^, and disease-related pathophysiology^9–11^. Here, we explored the interaction of chronotype and time-of-day on those aspects of human brain physiology, including cortical excitability and neuroplasticity, that determine adaptive behavior in both healthy humans and clinical populations. We also investigated motor learning and higher-order cognitive functions, such as attention and working memory, and their associations with respective physiological processes, to reveal mechanisms of chronotype-dependent performance differences.

Technological advances in neurosciences introduced noninvasive brain stimulation (NIBS) as a safe method for studying and directly modifying brain functions in humans^12^. Several NIBS techniques and protocols, including transcranial magnetic stimulation (TMS) and transcranial electrical stimulation (tES), are widely used to non-invasively monitor and induce changes of cortical excitability, and neuroplasticity^12,13^ that underlie behavior and cognition. Cortical excitability refers to responsiveness and response selectivity of cortical neurons to an input processed by the brain and is, therefore, a fundamental aspect of human brain functioning and cognition^5,14^. TMS, which is based on principles of electromagnetic induction, can be applied in different paradigms to measure various aspects of cortical excitability^15^. These paradigms provide information about different neurotransmitter systems involved in corticocortical and corticospinal excitability (e.g., glutamatergic, dopaminergic, GABAergic, cholinergic systems). Monitoring cortical excitability with TMS enhances our understanding of the physiology of brain functions and cognition^12,15^, as well as basic synaptic mechanisms involving long-term potentiation (LTP) or long-term depression (LTD)-like plasticity^13^.

Cortical excitability can be also modulated via induction of LTD/LTD-like plasticity, providing feasible opportunities for examining a specific and mechanistic contribution of cortical regions to human behavior^16,17^. Transcranial direct current stimulation (tDCS) is a tES technique that can modulate and induce changes in cortical excitability via a weak, painless electrical current applied to the scalp^12,18^. TDCS effects on cortical excitability are polarity-specific, with anodal stimulation inducing LTP-like plasticity and cathodal stimulation inducing LTD-like plasticity at the macroscopic level in humans^19,20^. Mechanisms of plasticity induction via tDCS were demonstrated in previous animal^21,22^ and human studies. These mechanisms are based on alterations of resting membrane potentials (for the acute effects) as well as glutamatergic, GABAergic, and calcium alterations, involving NMDA and AMPA receptors (for LTP LTD-like plasticity)^20,23^. Both, LTP and LTD-like processes are assumed important physiological substrates of learning and memory formation^17^. In this line, tDCS has been shown to modulate learning and memory formation^24^. Accordingly, if the propensity to develop neuroplasticity in the brain is modulated by chronotype, we expect to see respective effects on behavior, especially learning, and memory formation.

**Fig. 1:**
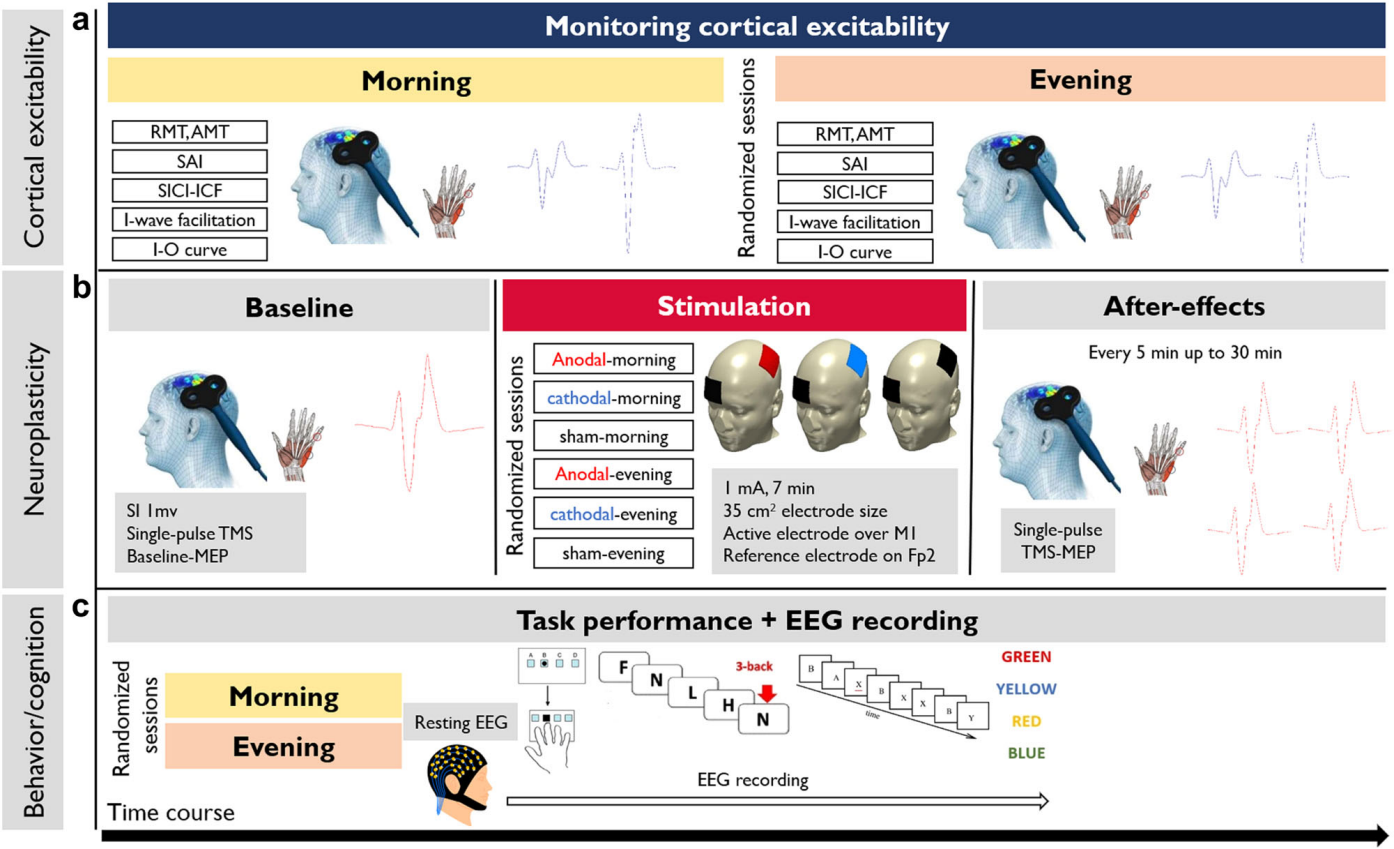
Course of study. **a** Sessions 1–2: Using single-pulse and double-pulse TMS protocols, corticospinal and corticocortical excitability were monitored at the circadian-preferred and circadian non-preferred times at fixed times for each group (8:00, 19:00) with at least 1-week interval. **b** Sessions 3–8: each participant attended six sessions of tDCS in randomized order. TDCS sessions started at a fixed time in the morning and evening (with at least 1-week between session interval). First, baseline cortical excitability was measured by inducing MEPs over the left M1 and measuring MEP of the target muscle (right abductor digiti minimi muscle). Following a baseline measurement of 25 MEPs, 7 min of anodal, cathodal, or sham stimulation was delivered. MEP measurements were then conducted immediately in epochs of every 5 min up to 30 min after tDCS. **c** Sessions 9–10: Following the resting-EEG acquisition, participants performed motor learning and cognitive (working memory, attention) tasks in two randomly assigned sessions at the same time in the morning and evening while their EEG was recorded (1-week interval). The order of tasks was counterbalanced across participants. All tasks (SRTT, N-back, Stroop, and AX-CPT) were presented on a computer screen (15.6″in. Samsung) in a soundproof electromagnetic shielded room during EEG recording. RMT   resting motor threshold, AMT   active motor threshold, SAI   short-latency afferent inhibition, SICI-ICF   short intracortical inhibition and facilitation, I–O curve input–output curve, TMS   transcranial magnetic stimulation, MEP   motor-evoked potential, SI 1 mv The stimulation intensity required to evoke MEPs with a peak-to-peak amplitude of an average of 1 mV, M1   primary left motor cortex, Fp2 right supraorbital area, EEG   electroencephalography, SRTT   serial reaction time task, AX-CPT   AX-continuous performance test.

Animal studies show a strong circadian impact on hippocampal plasticity and LTP^25,26^. Similarly, neural excitability in invertebrates^27^ and cortical excitability in the human motor cortex^28,29^ are modulated by circadian rhythms. However, the relevance of circadian preference for human cortical excitability and respective cognitive functions, and also brain plasticity and learning and memory formation are not well-studied. Increased understanding of respective mechanisms is important, not only for extending a basic knowledge of human brain functions but also because of the broader implications and applications to our daily life circumstances, such as working and educational environments. In this study, we first systematically investigated the modulatory impact of chronotype and time of day on cortical excitability and stimulation-induced neuroplasticity in the model of the human motor cortex. In the next step, we explored how chronotype is associated with performance on a motor learning task which is associated with motor cortical plasticity^30^. Finally, we investigated the association of chronotype with higher-order cognitive functions that are dependent on cortical excitability and usually controlled by non-motor areas (e.g., prefrontal cortex). In all behavioral tasks, we recorded electroencephalography (EEG) to further explore electrophysiological correlates of cognition under different chronotypes and times of the day. All measurements were conducted on two groups of “early chronotypes (ECs)” (i.e., morning type), and “late chronotypes (LCs)” (i.e., evening-types) at two fixed times in the morning and evening to capture circadian peaks and troughs at participants’ circadian-preferred and non-preferred times (Fig. 1). The sleep/wake timing, amount of sleep, ambient light, and seasonal variations during the experiment were controlled for or taken into account (see “Methods”).

## Results

### Enhanced corticospinal excitability, and cortical facilitation, but reduced inhibition at the circadian-preferred time

We first monitored corticospinal and intracortical excitability of the human motor cortex at circadian-preferred and non-preferred times. Unless otherwise stated in this article, circadian-preferred time refers to morning and evening for ECs and LCs and circadian non-preferred time refers to evening and morning for EC and LCs respectively. We obtained input–output curve (I–O curve), as a measure of global corticospinal excitability, and intracortical facilitation (ICF) as a measure of cortical facilitation. Short-interval cortical inhibition (SICI), I-wave facilitation, and short-latency afferent inhibition (SAI) were applied as cortical inhibition protocols. These TMS protocols are based on different neurotransmitter systems related to cortical facilitation (glutamatergic) and inhibition (GABAergic, cholinergic)^31–33^ (see “Methods”). Age, gender, and BMI did not covariate with motor-evoked potentials (MEPs) obtained from TMS protocols in the ANOVA analyses (Table 1).

**Table 1:** The results of mixed-factorial ANOVAs for cortical excitability parameters measured via different TMS protocols.

ppTMS protocol	Factor	df	*F*	*P*	*η*p^2^
I–O curve	Age	1	2.714	0.111	0.091
	Gender	1	0.259	0.614	0.009
	BMI	1	2.410	0.132	0.081
	Chronotype	1	0.359	0.553	0.013
	Daytime	1	0.099	0.754	0.003
	Intensity_(100%,110%, 130%,150% RMT)_	1.14	0.165	0.919	0.006
	Chronotype × daytime	1	25.432	**<0.001**	0.485
	Chronotype × intensity	1.14	0.522	0.499	0.018
	Daytime × intensity	1.29	1.570	0.203	0.054
	Chronotype × daytime × intensity	1.29	15.792	<**0.001**	0.369
SICI-ICF	Age	1	0.047	0.828	0.001
	Gender	1	0.611	0.441	0.022
	BMI	1	0.142	0.708	0.005
	Chronotype	1	0.224	0.639	0.008
	Daytime	1	0.217	0.644	0.007
	ISI_(2,3,5,10,15 ms)_	2.55	1.754	0.126	0.061
	Chronotype × daytime	1	72.168	**<0.001**	0.727
	Chronotype × ISI	2.55	0.255	0.826	0.009
	Daytime × ISI	3.49	0.708	0.569	0.025
	Chronotype × daytime × ISI	3.49	13.441	**<0.001**	0.332
I-wave facilitation	Age	1	0.022	0.882	0.001
	Gender	1	1.854	0.184	0.064
	BMI	1	0.001	0.980	0.001
	Chronotype	1	0.204	0.654	0.007
	Daytime	1	1.092	0.305	0.038
	ISI_(early,middle,late)_	1.35	1.126	0.315	0.040
	Chronotype × daytime	1	44.240	**<0.001**	0.621
	Chronotype × ISI	1.35	0.788	0.416	0.028
	Daytime × ISI	1.18	2.066	0.158	0.071
	Chronotype × daytime × ISI	1.18	1.087	0.316	0.038
SAI	Age	1	0.001	>0.999	0.001
	Gender	1	0.074	0.786	0.002
	BMI	1	0.351	0.557	0.012
	Chronotype	1	0.377	0.544	0.013
	Daytime	1	0.610	0.441	0.022
	ISI_(20,40 ms)_	1.96	1.359	0.265	0.047
	Chronotype × daytime	1	114.205	**<0.001**	0.808
	Chronotype × ISI	1.96	0.312	0.732	0.011
	Daytime × ISI	1.61	1.175	0.309	0.041
	Chronotype × daytime × ISI	1.61	30.106	**<0.001**	0.527

*ppTMS* paired-pulse TMS, *MEP* motor-evoked potentials, *chronotype* early and late chronotypes (ECs, LCs), *daytime* morning vs evening in each group, *ISI* interstimulus interval (in ms), *I–O* curve input–output curve, *SICI-ICF* short-latency intracortical inhibition and facilitation, *SAI* short-latency afferent inhibition, *RMT* resting motor threshold.

*Note*: Mixed-factorial ANOVAs with repeated measures (ISI × daytime × chronotype) were performed with ISIs, TMS intensity (in I-O curve only), and time of day (morning vs evening) as within-subject factors, chronotype (ECs vs LCs) as the between-subject factor, and age, gender and BMI as covariates. In case of statistical significance, post hoc comparisons were performed using Bonferroni-corrected *t* tests (two-sided). Significant effects are bold (where *P* < 0.05).

#### Input–output curve (I/O curve)

**Fig. 2:**
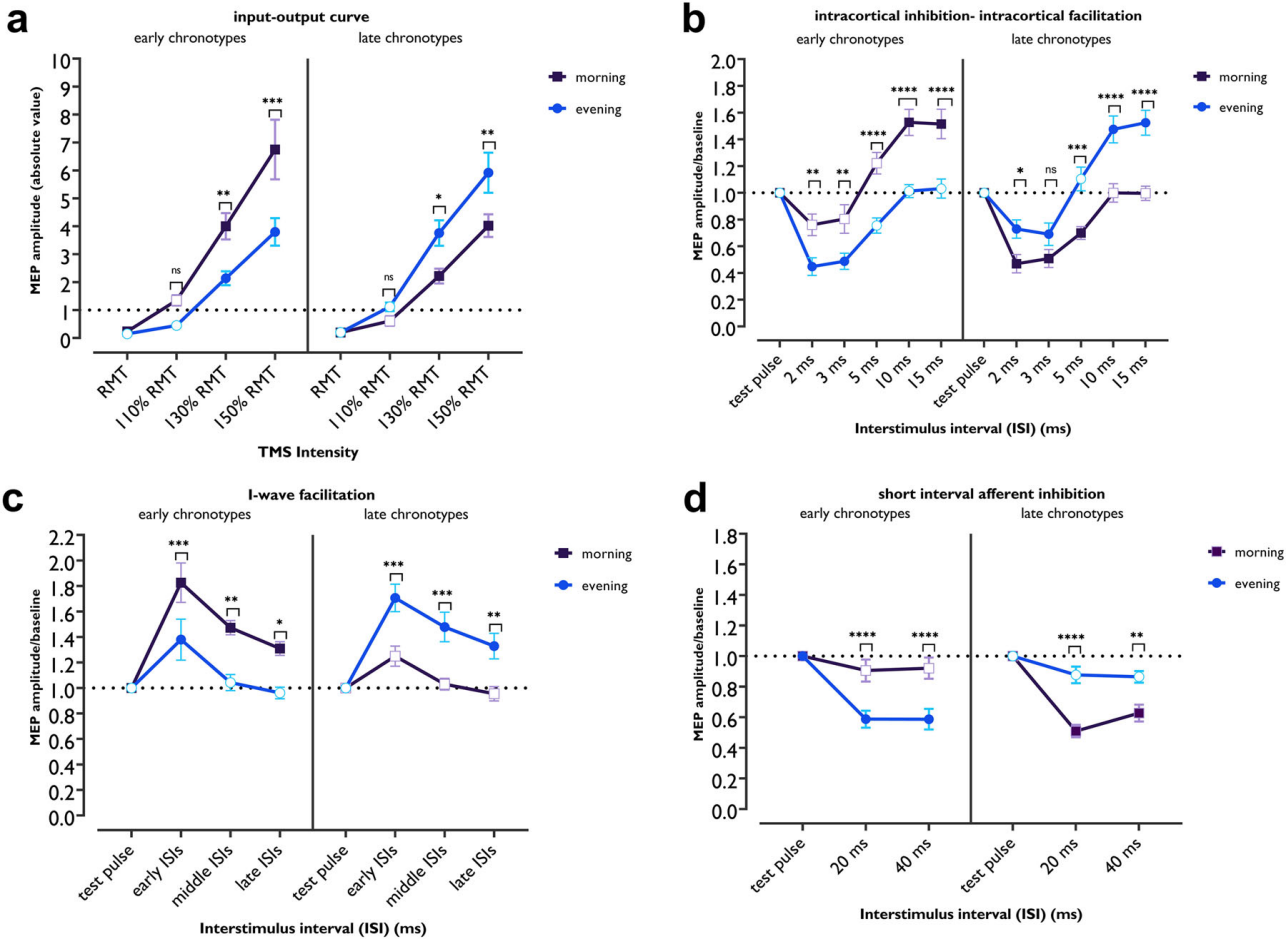
Monitoring corticospinal and corticocortical excitability with TMS protocols. Cortical excitability data were analyzed using mixed-factorial ANOVAs with repeated measures (see Table 1). **a** Global corticospinal excitability monitored by MEP amplitudes at different TMS intensities via the I–O curve protocol. ECs showed significantly higher corticospinal excitability in the morning than in the evening (*t*_130%_ = 3.19, *P* = 0.004; *t*_150%_ = 5.054, *P* < 0.001), and compared to the same time in LCs (*t*_130%_ = 3.05, *P* = 0.007; *t*_150%_ = 4.67, *P* < 0.001), and LCs display enhanced excitability in the evening (*t*_130%_ = 2.64, *P* = 0.026; *t*_150%_ = 3.248, *P* = 0.004), and compared to the same time in ECs (*t*_*130%*_ = 2.78, *p* = 0.017; *t*_*150%*_ = 3.63, *p* = 0.004) a*t* higher TMS intensities. **b** Intracortical inhibition and facilitation measured by the SICI-ICF paired-pulse (pp)TMS protocol. Significantly higher cortical inhibition in the evening and morning were observed for ECs (*t*_*ISI2*_ = 3.09, *p* = 0.006; *t*_*ISI3*_ = 3.13, *p* = 0.005) and LCs (*t*_*ISI2*_ = 2.57, *p* = 0.031; *t*_*ISI3*_ = 1.80, *p* = 0.217), respectively. In contrast, cortical facilitation was significantly enhanced in the morning, and evening for ECs (*t*_*ISI10*_ = 5.09, *P* < 0.001; *t*_*ISI15*_ = 4.79, *P* < 0.001) and LCs (*t*_*ISI10*_ = 4.71, *P* < 0.001; *t*_*ISI15*_ = 5.23, *P* < 0.001), respectively. **c** I-wave facilita*t*ion for monitoring GABA-dependent intracortical inhibition. Cortical excitability was significantly facilitated for early, middle, and late ISIs in the morning for ECs (*t*_early_ = 3.84, *P* = 0.009; *t*_middle_ = 3.70, *P* = 0.001; *t*_late_ = 2.992, *P* = 0.018) and in the evening for LCs (*t*_early_ = 3.92, *P* = 0.007; *t*_middle_ = 3.85, *P* = 0.009; *t*_late_ = 3.214, *P* = 0.009), indicative for less cortical inhibition. **d** Inhibitory effect of peripheral nerve stimulation on motor cortical inhibition, as measured by SAI. ECs showed more prominent cortical inhibition in the evening (*t*_*ISI20*_ = 4.76, *P* = 0.001; *t*_*ISI40*_ = 4.99, *P* < 0.001), whereas LCs showed more cortical inhibition in the morning (*t*_*ISI20*_ = 5.50, *P* < 0.001; *t*_*ISI40*_ = 3.56, *P* < 0.001). All pairwise comparisons were calculated using Bonferroni-correc*t*ed *t* tests (two-sided). *n* = 32 (16 per group). Data are presented as mean values ± SEM (standard error of means). Filled symbols represent a significant difference of MEP amplitudes compared to the respective test pulses (for SICI-ICF, I-wave, SAI) or MEP at RMT intensity (for I-O curve). Asterisks [*] represent statistically significant comparisons of each outcome measure across time of day for each group. ECs early chronotypes, LCs late chronotypes, MEP   motor-evoked potential, RMT   resting motor threshold, ms milliseconds; I–O curve input–output curve, ISI   interstimulus interval.

The I–O curve is a global measure of corticospinal excitability^34^ obtained by eliciting MEPs at a range of different TMS intensities (see “Methods”). The slope of the I–O curve reflects the excitability of corticospinal neurons modulated by glutamatergic activity at higher TMS intensities^34,35^. The ANOVA results show significant interactions of chronotype × daytime × TMS intensity (*F*_1.29_ = 15.79, *P* = 0.001; *η*p^2^ = 0.36) and chronotype×daytime (*F*_1_ = 25.43, *P* = 0.001; *η*p^2^ = 0.48) but no main effects of chronotype and daytime (morning vs evening) alone on the slope of the I–O curve (Table 1). Bonferroni-corrected post hoc comparisons showed that MEP amplitudes were significantly larger at 130 and 150% of resting motor threshold (RMT) intensity in the morning for ECs and in the evening for LCs compared to their circadian non-preferred time and the same timepoint in the other group (Fig. 2a).

#### SICI-ICF

In this double-pulse TMS protocol, the interstimulus interval (ISI) between a subthreshold conditioning stimulus and a suprathreshold test stimulus determines inhibitory (ISIs 2 and 3 ms) or facilitatory (ISIs 10 and 15 ms) effects on cortical excitability^36^, which are reflected by a reduction or enhancement of MEP amplitudes (see “Methods”). The results of the ANOVA show significant interactions of chronotype** × **daytime (*F*_1_ = 72.16, *P* = 0.001, *η*p^2^ = 0.72) and chronotype × daytime × ISI (*F*_3.49_ = 13.44, *P* = 0.001, *η*p^2^ = 0.33), but no significant effect of chronotype and time of day alone (Table 1). Bonferroni-corrected post hoc comparisons of MEP amplitudes revealed that both, ECs and LCs showed a significant increase of intracortical inhibition at ISIs of 2 and 3 ms at their circadian non-preferred time, compared with the single pulse-elicited MEP amplitudes (baseline) and respective ISIs at their circadian-preferred time (Fig. 2b). Cortical inhibition occurred in LCs in the evening too which was, however, significantly lower vs morning. Regarding intracortical facilitation, MEP amplitudes at ISIs of 10 and 15 ms were significantly increased only at the circadian-preferred time in both groups, when compared with single-pulse-elicited MEP amplitudes (baseline), respective ISIs at their circadian non-preferred time and the same timepoint in the other group (Fig. 2b). Together, these results demonstrate a significantly lower cortical inhibition and higher cortical facilitation at the circadian-preferred time in both groups.

#### I-wave facilitation

Another method to monitor cortical inhibition is to explore facilitatory interaction between I-waves in the motor cortex that originates from corticospinal neurons^37^. In this TMS protocol, a suprathreshold stimulus is followed by a subthreshold second stimulus at different ISIs. I-wave peaks are mainly observed at three ISIs occurring at about 1.1–1.5, 2.3–2.9, and 4.1–4.4 ms after test pulse application^37^. We grouped these ISIs in epochs of early, middle, and late ISIs and analyzed the MEP amplitude means. The ANOVA shows significant interactions of chronotype × daytime (*F*_1_ = 44.24, *P* = 0.001; *η*p^2^ = 0.62) but no main effects of chronotype and time of day on I-wave peaks (Table 1). Bonferroni-corrected post hoc comparisons showed a significant increase of I-wave peaks for early, middle, and late ISIs, as compared to single-pulse MEPs in both groups at their circadian-preferred time of day. Moreover, I-wave peaks were significantly facilitated at the circadian-preferred time vs the non-preferred time in each group and the same time in the other group (Fig. 2c). These results indicate reduced GABAergic inhibition at the circadian-preferred time.

#### SAI

SAI is a measure of cortical inhibition and reflects inhibitory modulation of the motor cortex via somatosensory inhibitory afferents. In this protocol, the TMS stimulus is coupled with peripheral nerve stimulation that has an inhibitory effect on motor cortex excitability at ISIs of 20 and 40 ms^38^. This inhibitory effect is linked to cholinergic^31^ and GABAergic^38^ systems. ANOVA results show significant interactions of chronotype × daytime (*F*_1_ = 114.20, *P* = 0.001; *η*p^2^ = 0.62) and chronotype × daytime × ISI (*F*_1.61_ = 30.10, *P* = 0.001; *η*p^2^ = 0.52), but no significant main effects of chronotype and time of day (Table 1). Bonferroni-corrected post hoc comparisons revealed a significantly pronounced inhibitory effect of peripheral stimulation on cortical excitability at the circadian non-preferred time in both groups compared to the single TMS pulse. Moreover, cortical inhibition was significantly reduced in each group at their circadian-preferred vs the non-preferred time and between groups at the respective timepoints (Fig. 2d). This result is consistent with that of SICI, suggesting a reduction of cortical inhibition at circadian-preferred times.

Taken together, we monitored cortical excitability in ECs and LCs and found a strong dependence of motor cortical excitability parameters on chronotype and time-of-day, indicative of a prominent association of these factors with the excitability-related neurotransmitter systems. When participants were at their circadian-preferred time, they showed higher levels of corticospinal excitability and cortical facilitation, and a lower level of cortical inhibition (Fig. 2), in accordance with a higher glutamatergic and lower GABAergic activity during the circadian-preferred, as compared to the non-preferred time of day. Neither the baseline measurements of protocols nor the stimulation intensity required to evoke MEP did differ across groups and times of day (Supplementary Tables 1 and 2). The results thus cannot be explained by different stimulation intensities across times of the day.

### LTP/LTD-like plasticity in the motor cortex is facilitated at the circadian-preferred time in early and late chronotypes

**Fig. 3:**
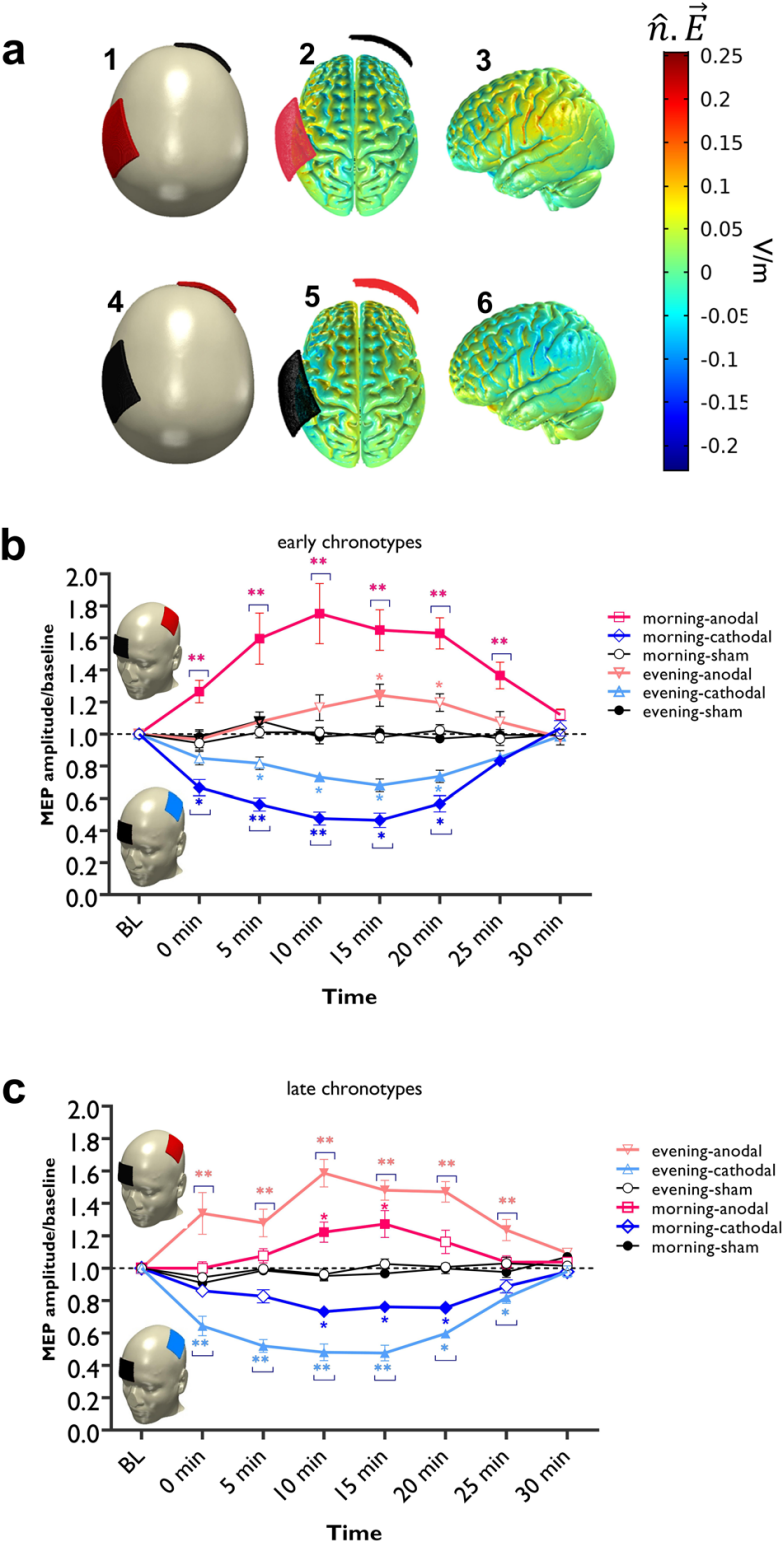
LTP/LTD-like plasticity induction in the motor cortex. **a** 3D model of the current flow distribution inside the head was calculated based on the finite element method using COMSOL Multiphysics software version 5.2 (details in supplementary information). The electrical current flow induced by 1.0 mA stimulation intensity, and electrode positions C3-Fp2, for anodal (a1, 2, 3), and cathodal (a4, 5, 6) stimulation over the motor cortex is shown. *ñ*.E refers to the absolute electrical field. **b**, **c** post-tDCS cortical excitability alterations after anodal, cathodal, and sham stimulation at the circadian-preferred and non-preferred times in early (**b**) and late (**c**) chronotypes (*n* = 32, 16 per group). The results of the repeated measures ANOVA showed significant interactions of stimulation × chronotype × daytime and stimulation × chronotype × daytime × timeline (see Table 2). The main effects of time of day and chronotype were not significant, however, they significantly interacted. Stimulation and timepoint did not significantly interact with chronotype or time of day (Table 2). Post hoc comparisons (Bonferroni-corrected *t* tests, two-tailed) of MEP amplitudes to respective baseline values, the sham condition, and respective stimulation conditions at different times of day are marked by symbols in the figures. Filled symbols indicate a significant difference of cortical excitability against the respective baseline values. The floating symbol [*] indicates a significant difference between the real vs sham tDCS conditions, and the floating symbol [**] indicates an additional significant difference between respective timepoints of tDCS conditions at the circadian-preferred vs circadian non-preferred times. Sham stimulation did not induce any significant change in cortical excitability. Data are presented as mean values ± SEM. MEP   motor-evoked potential, C3   left motor cortex, Fp2 right supraorbital area, V/m volts per meter.

Having demonstrated that cortical excitability in the motor cortex is chronotype-dependent, we were next interested in determining how the time-of-day-dependent variation of cortical excitability affects LTP/LTD-like plasticity in early and late chronotypes. We predicted that motor cortical plasticity should be facilitated at the circadian-preferred time too. To test this hypothesis, we stimulated the primary motor cortex with anodal, cathodal, and sham (control condition) tDCS (1 mA, 7 min, Fig. 3a) in each group at the same time in the morning and evening (six sessions, weekly). We then monitored neuroplastic effects of tDCS via single-pulse TMS with a fixed medium intensity before and after the intervention (see “Methods”). Depending on the stimulation polarity, tDCS results in LTP-like or LTD-like plasticity. With the chosen protocol, anodal tDCS enhances, while cathodal tDCS diminishes motor cortex excitability^39^ which can last for an hour or longer after tDCS^39,40^. Analysis of blinding efficacy showed that participants could not discern between active and respective sham tDCS conditions (Supplementary Table 6). Side effects were minor and did not differ between intervention conditions, except for the tingling and burning sensations (Supplementary Tables 4 and 5). Age, gender, and BMI did not covariate with TMS-induced MEP in the ANOVA analyses. ANOVA results showed significant interactions of chronotype × daytime stimulation × chronotype × daytime and stimulation × chronotype × daytime × timepoint (*F*_6.78_ = 10.82, *P* = 0.001; *η*p^2^ = 0.28) but no interaction of stimulation × chronotype and timepoint × chronotype. The main effects of chronotype and time of day were not significant (Table 2).

**Table 2:** The results of mixed-factorial ANOVA for the effect of tDCS on MEP amplitudes in early and late chronotypes.

Factor	df	*F*	*P*	*η*p^2^
Age	1	0.898	0.351	0.032
Gender	1	0.001	0.996	0.001
BMI	1	1.728	0.199	0.060
Chronotype	1	0.743	0.396	0.026
Daytime	1	0.037	0.848	0.001
Stimulation _(anodal,cathodal,sham)_	1.196	0.585	0.478	0.021
Timepoint	4.206	1.113	0.354	0.039
Chronotype × daytime	1	8.632	**0.007**	0.242
Chronotype × stimulation	1.196	1.001	0.339	0.035
Daytime × stimulation	1.764	3.766	**0.035**	0.122
Chronotype × timepoint	4.206	1.564	0.185	0.054
Daytime × timepoint	4.066	0.529	0.716	0.019
Stimulation × timepoint	5.857	0.822	0.551	0.029
Chronotype × daytime × stimulation	1.764	55.098	**<0.001**	0.671
Chronotype × daytime × timepoint	4.066	1.382	0.244	0.048
Chronotype × daytime × stimulation × daytime	6.784	10.826	**<0.001**	0.286

*tDCS* transcranial direct current stimulation, MEP motor-evoked potentials.

*Note*: Individual averages of the normalized MEP were analyzed using a mixed-factorial design with repeated measures ANOVA (stimulation × timepoint × daytime × chronotype) with stimulation condition (anodal, cathodal, sham), timepoint (eight levels), and time of day (morning vs evening) as within-subject factors, chronotype (early vs late) as between-subject factor, and age, gender and BMI as covariates. In the case of significant ANOVA results, post hoc comparisons of MEP amplitudes at each timepoint were performed using Bonferroni-corrected post hoc *t* tests (two-sided). Significant effects are bold (where *P* < 0.05), *n* = 32 (16 per group).

#### Anodal stimulation

We analyzed the effects of anodal tDCS on MEP amplitudes compared to the baseline, across daytime, and against sham condition via Bonferroni-corrected post hoc *t* tests. For ECs, MEP amplitudes significantly increased at 5, 10, 15, 20, and 25 min after intervention in the morning, but only at 15 min in the evening, as compared to baseline MEP. Importantly, the increase of MEP amplitudes in the morning was significantly higher at all timepoints except for 30 min after the stimulation, when compared to the evening session and against the sham intervention (Fig. 3b). A reversed pattern of response was found for the LCs. Anodal tDCS significantly increased MEP amplitudes at 5, 10, 15, 20, and 25 min after stimulation in the evening and only at 10 and 15 min in the morning. The increase of MEP amplitudes in the evening was significantly higher when compared to the morning session and against the sham intervention for all timepoints, except 30 min after stimulation (Fig. 3c).

#### Cathodal stimulation

Here, respective post hoc *t* tests showed that cathodal tDCS resulted in a significant decrease of MEP amplitudes in both chronotypes at their circadian-preferred time compared to the baseline MEP and against sham at all times points, except for 25 and 30 min after stimulation (Fig. 3b, c). Both groups showed a significant decrease of MEP amplitudes at 10, 15, and 20 min after cathodal stimulation at their circadian non-preferred time as well. However, the MEP decrease after stimulation was significantly larger in the morning for ECs (at 5 and 10 min) and in the evening for the LCs (0, 5, 10, 15), when compared to MEP size at the respective circadian non-preferred time (Fig. 3b, c).

Together, these results indicate that tDCS-induced LTP- and LTD-like plasticity of the motor cortex (after anodal and cathodal stimulation), which are dependent on glutamate and GABA activity^41^, were significantly stronger and longer-lasting in both, ECs and LCs at their circadian-preferred time. This aligns with our findings of higher cortical facilitation and lower cortical inhibition in the motor cortex at the circadian-preferred time, as described earlier.

### Association of chronotype with behavioral and electrophysiological aspects of motor learning

We found daytime-specific impact of chronotype on basic physiological functions of the motor cortex. LTP/LTD are important physiological foundations for learning and memory formation. The concentration of GABA^42^ and glutamate^17^ is important for motor learning and synaptic strengthening as well. If circadian preference modulates LTP/LTD processes and respective neurotransmitter systems, as shown in the previous section, superior learning is expected at the circadian-preferred time. To this end, we investigated sequence motor learning using the serial motor learning task (SRTT) and its electrophysiological correlates and predicted enhanced motor learning at the circadian-preferred time. To test this hypothesis, participants in both groups performed SRTT at the same time in the morning and evening during EEG recording (see “Methods”).

**Fig. 4:**
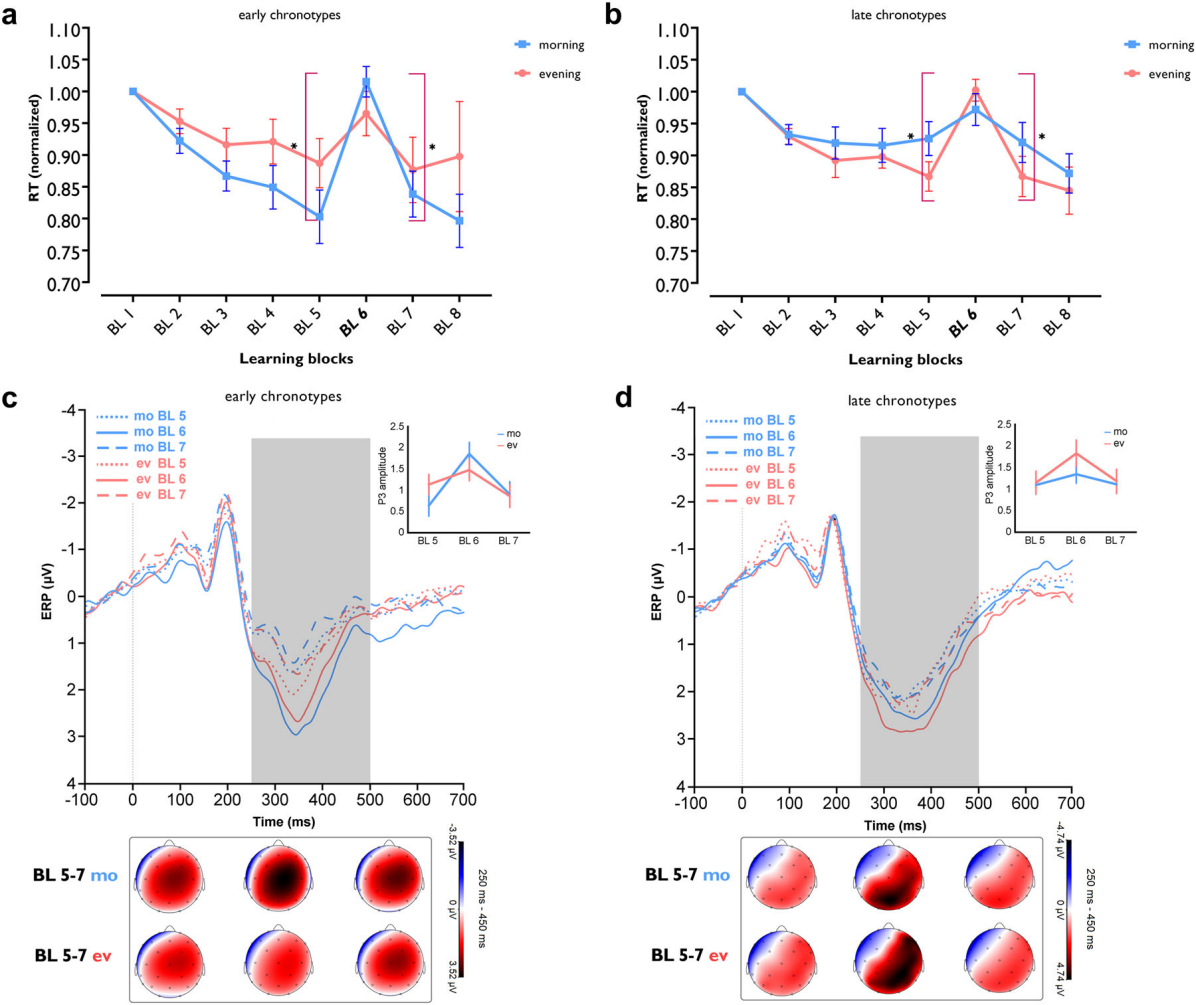
Chronotype affects motor learning performance and ERP correlates. Behavioral and electrophysiological data were analyzed using a mixed-factorial design with repeated measures ANOVA (see Table 3). **a**, **b** The RT difference between blocks 6–5 mostly exclusively represents sequence learning. In ECs, the RT difference between these blocks was significant both in the morning and evening (*t*_morning_ = 5.70, *P* < 0.001, *t*_evening_ = 2.93, *P* = 0.010), but blocks 6–5 RT difference in the morning vs evening was significantly larger (*t* = 2.90, *P* = 0.012). In LCs, the respective RT difference was significant both in the evening and morning (*t*_evening_ = 8.78, *P* < 0.001; *t*_morning_ = 2.40, *P* = 0.029), and blocks RT difference was significantly larger in the evening vs morning (*t* = 2.72, *P* = 0.016). The RT difference between blocks 6 and 7 was significant in the morning and evening for both ECs (*t*_morning_ = 5.53, *P* < 0.001; *t*_evening_ = 3.39, *P* = 0.004) and LCs (*t*_evening_ = 5.06, *P* < 0.001; *t*_morning_ = 2.43, *P* = 0.028). Blocks 6–7 RT difference in the morning vs evening was only marginally significant for LCs (*t* = 2.11, *P* = 0.052). Asterisks [*] represent s*t*atistically significant differences between learning blocks RT (BL 6-5, BL 6-7]. The brackets refer to RT difference between blocks 6 vs 5 and 6 vs 7 in the morning for ECs and evening for LCs. **c**, **d** The P300 component of electrode Pz was calculated per block in both groups. Pairwise comparisons show that ECs displayed a significantly larger P300 at block 6 vs block 5 in the morning (*t* = 4.63, *P* < 0.001) vs evening (*t* = 1.29, *P* = 0.198), whereas LCs showed reversed results in the evening (*t* = 3.22, *P* = 0.001) vs morning (*t* = 1.18, *P* = 0.239). The P300 positivity of electrode Pz was significantly reduced at block 7 vs block 6 in ECs (*t*_morning_ = 3.62, *P* < 0.001; *t*_evening_ = 2.33, *P* = 0.022) and LCs (*t*_morning_ = 1.11, *P* = 0.269; *t*_evening_ = 3.07, *P* = 0.002) at their circadian-preferred time. All pairwise comparisons in panels. **a**–**d** are calculated using Student’s *t* test (*P* < 0.05, two-tailed, non-corrected). *n* = 31 (15 ECs, 16 LCs; data of one EC participant are excluded due to sequence awareness). Data are presented as mean values ± SEM. RT   reaction time, BL   block, mo morning, ev evening, P3   P300 component, ms milliseconds, ERP   event-related potential, µV microvolt, ECs early chronotypes, LCs late chronotypes.

We analyzed the differences in the standardized reaction time (RT) of block 5 vs 6, which is indicative of motor sequence learning acquisition, and block 6 vs 7 which is indicative of motor sequence learning retention (for absolute RT see Supplementary Fig. 1). The 3 × 2 × 2 ANOVA results showed a significant interaction of block × chronotype × daytime (*F*_1.89_ = 9.97, *P* < 0.001; *η*p^2^ = 0.27) but no interaction of block × daytime, chronotype × daytime, or main effect of chronotype and daytime (Fig. 4 and Table 3). Post hoc comparisons *t* tests revealed that both groups significantly displayed longer RT at block 6 compared to block 5, indicative of sequence motor learning, at their circadian-preferred time (Fig. 4a, b). Importantly, the blocks 6–5 RT difference was significantly larger in both groups at their circadian-preferred time compared to the respective non-preferred time. To test if the learning sequence was preserved after the presentation of random stimuli in block 6, we analyzed RT differences of block 6 vs 7 too. The results showed that sequence learning was significantly retained at the circadian-preferred time as well (Fig. 4a, b). Baseline block and block 6 RT, which contain stimuli in random order, did not significantly differ across morning and evening sessions in both groups (*F*_1_ = 3.39, *P* = 0.076) and therefore, a generally slower RT at the circadian non-preferred time cannot explain RT differences in the learning blocks. In addition, we analyzed the number of errors and RT variability in the learning blocks and found that both groups committed more errors at block 6 at their circadian non-preferred time (Supplementary Fig. 2a, b).

**Table 3:** Results of mixed factorial ANOVAs for cognitive task performance and respective ERP components.

Task	Measurement	Factor	df	*F*	*P*	*η*p^2^
SRTT (motor learning)	RT	Age	1	2.607	0.118	0.091
		Gender	1	0.366	0.549	0.013
		BMI	1	0.918	0.346	0.034
		Learning blocks (BL5-7)	1.63	1.722	0.195	0.062
		Chronotype	1	0.712	0.406	0.026
		Daytime	1	3.217	0.084	0.110
		Chronotype × daytime	1	2.431	0.130	0.085
		Learning blocks × chronotype	1.63	3.938	**0.034**	0.131
		Learning blocks × daytime	1.89	2.815	0.072	0.097
		Learning blocks × chronotype × daytime	1.89	9.971	<**0.001**	0.277
	P300	Age	1	3.137	0.088	0.010
		Gender	1	0.042	0.837	0.001
		BMI	1	0.642	0.430	0.024
		Learning blocks (BL5-7)	1.74	0.528	0.592	0.019
		Chronotype	1	3.394	0.076	0.115
		Daytime	1	2.593	0.119	0.090
		Chronotype × daytime	1	1.486	0.233	0.054
		Learning blocks × chronotype	1.74	0.311	0.734	0.011
		Learning blocks × daytime	1.99	0.771	0.467	0.028
		Learning blocks × chronotype × daytime	1.99	6.058	**0.004**	0.188
3-back (working memory)	Accuracy	Age	1	0.614	0.439	0.022
		Gender	1	0.010	0.920	0.001
		BMI	1	0.791	0.381	0.028
		Chronotype	1	1.311	0.262	0.046
		Daytime	1	0.073	0.788	0.002
		Daytime × chronotype	1	10.347	**0.003**	0.277
	d prime	Age	1	0.336	0.566	0.012
		Gender	1	0.049	0.826	0.001
		BMI	1	0.256	0.616	0.009
		Chronotype	1	0.768	0.388	0.027
		Daytime	1	0.022	0.881	0.001
		Daytime × chronotype	1	10.82	**0.002**	0.286
	RT	Age	1	0.053	0.818	0.001
		Gender	1	2.174	0.151	0.074
		BMI	1	1.665	0.207	0.058
		Chronotype	1	0.083	0.775	0.003
		Daytime	1	0.580	0.452	0.021
		Daytime × chronotype	1	1.353	0.254	0.047
	P300-Pz	Age	1	0.988	0.328	0.035
		Gender	1	4.694	0.039	0.148
		BMI	1	0.432	0.516	0.015
		Chronotype	1	0.936	0.341	0.033
		Daytime	1	0.001	0.988	0.001
		Daytime × chronotype	1	12.395	**0.001**	0.314
	P300-Cz	Age	1	0.943	0.340	0.033
		Gender	1	0.001	0.999	0.001
		BMI	1	0.595	0.447	0.021
		Chronotype	1	0.137	0.714	0.005
		Daytime	1	0.959	0.336	0.034
		Daytime × chronotype	1	11.077	**0.002**	0.290
Stroop (selective attention)	Stroop block RT	Age	1	0.333	0.568	0.012
		Gender	1	0.162	0.690	0.005
		BMI	1	0.379	0.543	0.013
		Chronotype	1	0.038	0.845	0.001
		Daytime	1	2.768	0.107	0.093
		Congruency	1	0.653	0.425	0.023
		Daytime × chronotype	1	22.378	**<0.001**	0.453
		Congruency × chronotype	1	0.688	0.414	0.024
		Congruency × daytime	1	0.003	0.955	0.001
		Congruency × chronotype daytime	1	0.548	0.465	0.019
	Congruent trials RT	Age	1	0.388	0.538	0.014
		Gender	1	0.019	0.890	0.001
		BMI	1	0.286	0.596	0.010
		Chronotype	1	0.008	0.926	0.001
		Daytime	1	2.249	0.145	0.076
		Daytime × chronotype	1	15.707	**<0.001**	0.367
	Incongruent trials RT	Age	1	0.279	0.601	0.010
		Gender	1	0.404	0.530	0.014
		BMI	1	0.462	0.502	0.016
		Chronotype	1	0.083	0.775	0.003
		Daytime	1	2.649	0.115	0.089
		Daytime × chronotype	1	24.622	**<0.001**	0.476
	RT variability	Age	1	0.805	0.377	0.028
		Gender	1	0.530	0.472	0.019
		BMI	1	0.991	0.328	0.035
		Chronotype	1	1.113	0.300	0.039
		Daytime	1	0.014	0.906	0.001
		Daytime × chronotype	1	22.475	**<0.001**	0.454
	N200-Fz (congruent trials)	Age	1	0.110	0.742	0.004
		Gender	1	1.563	0.222	0.056
		BMI	1	0.013	0.906	0.001
		Chronotype	1	1.312	0.262	0.048
		Daytime	1	0.001	0.982	0.001
		Daytime × chronotype	1	17.125	**<0.001**	0.397
	N200-Fz (incongruent trials)	Age	1	0.259	0.614	0.009
		Gender	1	1.662	0.208	0.060
		BMI	1	0.016	0.899	0.001
		Chronotype	1	2.382	0.134	0.083
		Daytime	1	0.043	0.835	0.001
		Daytime × chronotype	1	7.178	**0.012**	0.216
AX-CPT (sustained attention)	Accuracy	Age	1	1.824	0.187	0.063
		Gender	1	0.942	0.340	0.033
		BMI	1	0.035	0.852	0.001
		Chronotype	1	0.508	0.481	0.018
		Daytime	1	0.001	0.994	0.001
		Daytime × chronotype	1	14.169	**<0.001**	0.344
	RT	Age	1	0.210	0.649	0.007
		Gender	1	0.270	0.607	0.009
		BMI	1	0.754	0.392	0.027
		Chronotype	1	0.002	0.958	0.001
		Daytime	1	0.614	0.439	0.022
		Daytime × chronotype	1	19.390	**<0.001**	0.417
	P300-Pz	Age	1	0.237	0.629	0.008
		Gender	1	5.017	**0.033**	0.151
		BMI	1	0.805	0.377	0.027
		Chronotype	1	0.244	0.624	0.008
		Daytime	1	0.837	0.367	0.029
		Daytime × chronotype	1	5.337	**0.028**	0.160

*BL* block, *RT* reaction time, *SRTT* serial reaction time task, *AX-CPT* AX-continuous performance test.

*Note*: Dependent variables were entered in a mixed-model ANOVA with chronotype (early vs late) as the between-subject factor, time of day (morning vs evening) as the within-subject factor, and age, gender and BMI as covariates. In SRTT, block (5, vs 6, 6 vs 7) was entered as an additional within-subject factor. For significant ANOVAs, post hoc comparisons of dependent variables across time of day (morning vs evening) were performed using uncorrected paired-sample *t* tests (two-sided). d prime = it is an index of and is calculated as d = ZHit—ZFA where Hit represents the proportion of hits when a signal is present (hits/(hits + misses)), also known as the hit rate, and FA represents the proportion of false alarms when a signal is absent (false alarms/(false alarms + correct negative)), the false-alarm rate. Significant effects are bold (where *P* < 0.05).

Next, we explored electrophysiological correlates of motor learning by analyzing event-related potentials (ERPs) of the learning blocks (see “Methods”). The P300 component is evoked in response to stimuli of low probability and consists of the P3a (250–280 ms, reflecting frontal activity) and P3b (250–500 ms, reflecting temporoparietal activity) waves^43^. It is affected by stimulus characteristics, including stimulus sequence^44,45^ and is related to motor decision mechanisms^46^. We expected a higher-amplitude P300 component when the learned sequence of stimuli is violated (random block 6), at the circadian-preferred times which resulted in superior motor learning. To test for statistical significance, we analyzed the P300 amplitudes (250–500 ms) of blocks (5–7) and amplitude differences at block 5 vs 6 (sequence acquisition) and block 6 vs 7 (sequence retention). The ANOVA results revealed a significant interaction of block × chronotype × daytime (*F*_1.99_ = 6.58, *P* = 0.004; *η*p^2^ = 0.18) for P300 at the Pz electrode, but no significant main effects of chronotype and daytime (Fig. 4 and Table 3). Post hoc comparisons confirmed our prediction and both, early and late chronotypes had a significantly larger P300 amplitude in block 6 compared to blocks 5 and 7 (indicative of sequence learning) at their circadian-preferred time (ECs: mean ± SEM_morning_, 1.84 ± 0.26 µV, mean ± SEM_evening_, 1.46 ± 0.25 µV; LCs: mean ±  SEM_morning_, 1.86 ± 0.28 µV, mean ± SEM_evening_, 2.54 ± 0.42 µV) (Fig. 4c, d). We found a similar trend of P300 amplitude at the P3 electrode (Supplementary Fig. 3). Together, these results highlight the relationship between the circadian-preferred time and recruitment of motor learning-specific electrophysiological processes that are associated with performance. It should be noted though that ERP amplitude enhancement at the circadian-preferred time could reflect enhanced learning, but also the faster frequency of movements caused by learning-related faster RT.

We also explored the association between motor learning, and plasticity by calculating correlations between MEPs amplitudes and motor sequence learning. Briefly, we found a positive correlation between anodal tDCS effects (MEP amplitude enhancement) and sequence learning (blocks 6–5 RT difference) in the evening for LCs (*r* = 0.543, *P* = 0.017). No significant correlation between sequence learning and tDCS-induced plasticity was found in ECs (see Supplementary Information).

### Behavioral and electrophysiological correlates of cognition are enhanced at the circadian-preferred time

Our results also indicated chronotype-specific variations of cortical excitability. Due to the links between cortical excitability, especially glutamate and GABA regulating, and cognitive processes^13,47^, we next sought to determine the association of chronotype with higher cognitive functions (e.g., working memory, attention), and respective ERP components as physiological indicators of information processing. All participants conducted the 3-back letter (working memory task), Stroop and AX-continuous performance tasks (AX-CPT) (attention tasks), during EEG recording at their circadian-preferred and non-preferred times (see “Methods”). Age, gender, and BMI did not covariate with the task outcome measures in the ANOVA analyses (Table 3).

**Fig. 5:**
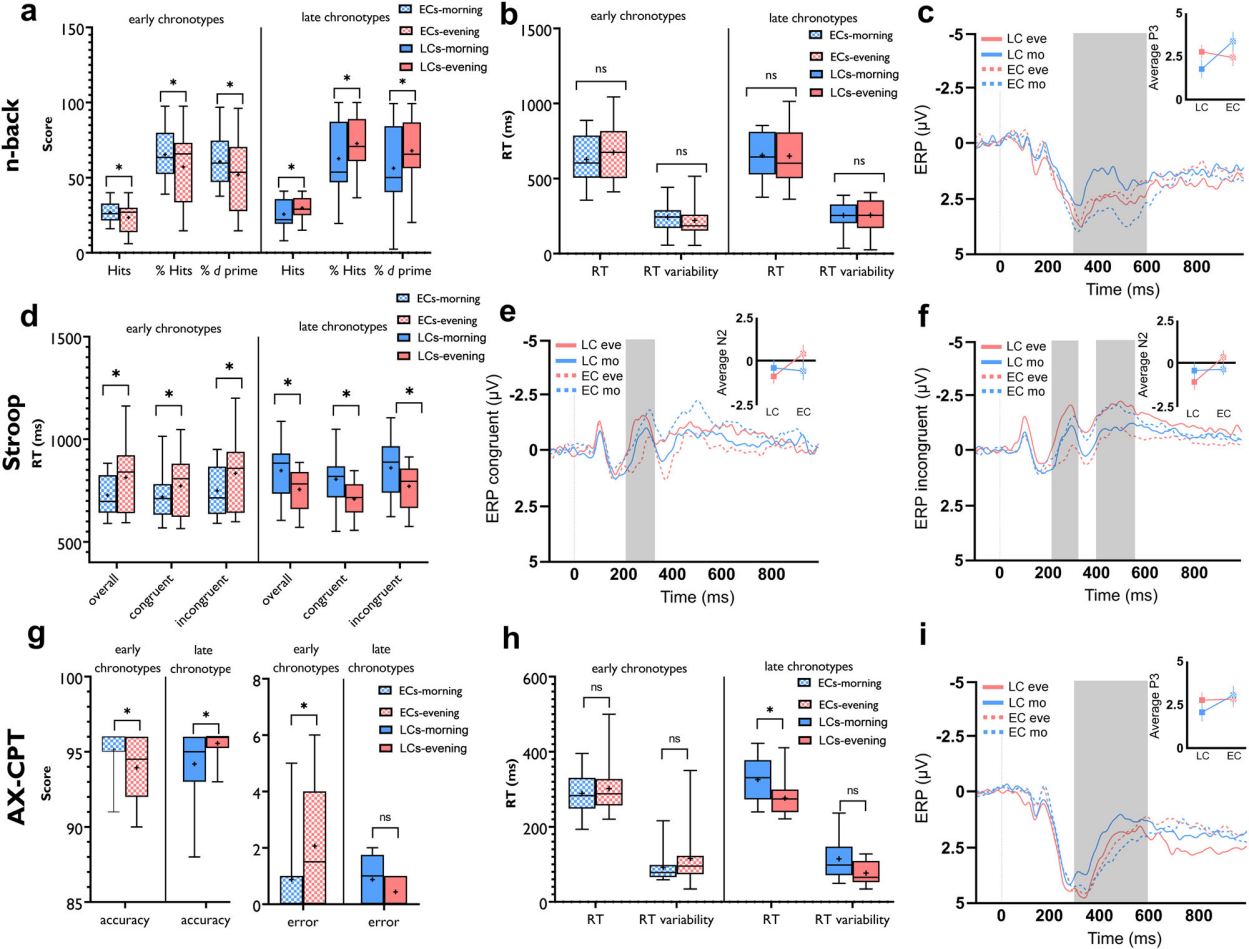
Association of chronotype with higher-order cognition (working memory and attention). Behavioral and electrophysiological data were analyzed using mixed-factorial (daytime × chronotype) ANOVAs (see Table 3). **a** ECs had significantly higher “Hits” and percentage of “Hits” in the morning vs evening (*t* = 2.53, *P* = 0.023), whereas LCs showed the reverse pattern of results (*t* = 2.36, *P* = 0.032) in the 3-back working memory task. Both groups showed the same pattern for the *d* prime (*t*_ECs_ = 2.55, *P* = 0.022; *t*_LCs_ = 2.49, *P* = 0.025) showing a significantly higher accuracy percentage and d prime value at their circadian-preferred time. **b** RT was not significantly different across time of day in the groups (*t*_ECs_ = 1.13, *P* = 0.275; *t*_LCs_ = 0.18, *P* = 0.858). **c** ECs displayed a larger P300 component in the morning vs evening (*t* = 3.62, *P* = 0.003) and LCs showed a larger P300 in the evening (*t* = 2.27, *P* = 0.038) at electrode Pz. **d** Both, ECs (*t*_morning_ = 1.91, *P* = 0.074; *t*_evening_ = 5.88, *P* < 0.001) and LCs (*t*_morning_ = 4.32, *P* = 0.001; *t*_evening_ = 5.60, *P* < 0.001) displayed a stronger Stroop interference effect (RT_incongruent_ − RT_congruent_) at their circadian non-preferred time. RT of the congruent, incongruent and overall trials were significantly slower at the circadian non-preferred time (ECs: *t*_con_ = 1.66, *P* = 0.117; *t*_incon_ = 2.63, *P* = 0.019; *t*_overall_ = 3.34, *P* = 0.004; LCs: *t*_con_ = 4.13, *P* = 0.001; *t*_incon_ = 5.26, *P* < 0.001; *t*_overall_ = 5.17, *P* < 0.001). **e**, **f** The N200 amplitude at electrode Fz was calculated in the Stroop stage for both, congruent and incongruent trials. It was larger for both groups at their circadian-preferred times, but the difference was significant only in ECs (*t*_con_ = 4.81, *P* = 0.001; *t*_incon_ = 2.65, *P* = 0.018). However, chorotypes had a significant effect on the “morning vs evening” N200 amplitude difference (see “Results”). For the N450 component details see supplementary Fig. 4f. **g**, **h** In the AX-CPT, both groups showed enhanced sustained attention, as indexed by higher accuracy (ECs: *t* = 2.64, *P* = 0.018; LCs: *t* = 2.62, *P* = 0.019) at their circadian-preferred times. The respective RT difference was significant in LCs only (*t* = 4.22, *P* = 0.001). **i** The P300 difference be*t*ween morning and evening was significantly larger only for LCs (*t* = 2.63, *P* = 0.019). In **a**, **b**, **d**, **g**, **h**, the horizontal bar shows the median, the + shows the mean, the upper and lower boundaries show the 25th and 75th percentiles, respectively, and the whiskers show 5–95 percentile. All pairwise comparisons in panels **a**–**d** are calculated using Student’s *t* test (*P* < 0.05, two-tailed). *n* = 32 (16 per group). Data are presented as mean values ± SEM. ECs early chronotypes, LCs late chronotypes, eve evening, mo morning, RT  reaction time, AX-CPT   AX-continuous performance test, ERP   event-related potential, µV microvolt, P3   P300 component, N2   N200, ns nonsignificant. Asterisks [*] represent statistically significant differences.

For working memory performance, the ANOVA results revealed a significant chronotype** × **daytime interaction (*F*_1_ = 10.34, *P* = 0.003; *η*p^2^ = 0.27) for the N-back hits, which is the primary outcome of interest in this task (Table 3). Post hoc Student’s *t* tests showed a significantly enhanced WM performance in the morning for ECs and evening for LCs (Fig. 5a). The percentage of hits was 65.39% and 57.15% for ECs in the morning and evening respectively and 72.71% and 62.65 for LCs in the evening and morning respectively. In addition, we calculated the sensitivity index *d* (or d prime) which represents the proportion of hits rate minus the proportion of false-alarm rate. A significant interaction of chronotype**×**daytime (*F*_1_ = 10.82, *P* = 0.003; *η*p^2^ = 0.28) was found with no main effect of chronotype or time of day (Table 2). Post hoc Student’s *t* tests showed a significantly enhanced *d* prime index in the morning for ECs and evening for LCs (Fig. 5a). RT and RT variability were not different across time of day (Fig. 5b). Next, we analyzed electrophysiological correlates of N-back task performance and found a significant interaction of chronotype × daytime in the P300 component at electrode Pz (*F*_1_ = 12.39, *P* < 0.001; *η*p^2^ = 0.31), and Cz (*F*_1_ = 11.07, *P* = 0.002; *η*p^2^ = 0.29) which is an indicator of memory-updating processes^48^. Post hoc comparisons via Student’s *t* tests showed that performance during the circadian-preferred time was related to a larger P300 amplitude under the Pz electrode in both groups (ECs: mean ± SEM_morning_, 3.37 ± 0.50 µV, mean ± SEM_evening_, 2.43 ± 0.47µV; LCs: mean ± SEM_morning_, 1.76 ± 0.48 µV, mean ± SEM_evening_, 2.75 ± 0.39 µV) (Fig. 5c). The same trend was observed for electrode Cz (Supplementary Fig. 4a, b).

For Stroop task performance, a similar trend of response was observed. We found a significant chronotype**×**daytime interaction on overall RT (*F*_1_ = 22.37, *P* < 0.001; *η*p^2^ = 0.45), RT of congruent trials (*F*_1_ = 15.70, *P* < 0.001; *η*p^2^ = 0.36) and RT of incongruent trials (*F*_1_ = 24.62, *P* < 0.001; *η*p^2^ = 0.47). The main effects of time of day and chronotype were not significant (Fig. 5, Table 3). Post hoc comparisons of RTs revealed a significantly less Stroop effect (faster RT to incongruent trials) at the circadian-preferred time in both groups (Fig. 5d). This indicates less Stroop interference in participants when they were conducting the task at their circadian-preferred time. The same pattern of results was observed for RT variability in the Stroop block. The results of the ANOVA showed a significant interaction of chronotype × daytime (*F*_1_ = 22.47, *P* < 0.001; *η*p^2^ = 0.45) and post hoc comparisons revealed a significantly reduced variability of RT in the Stroop block at the circadian-preferred time for both groups (ECs: *t* = 2.81, *P* = 0.013; LCs: *t* = 4.62, *P* < 0.001). Performance accuracy was not significantly affected. The N200 and N450 are two prominent ERP markers related to Stroop conflict and are observed at frontocentral and centroparietal regions^49,50^. The less Stroop effect we observed at the circadian-preferred times was associated with larger N200 and N450 amplitudes which are indicative of higher selective attention and discriminating ability for conflicting stimuli. The results of ANOVA for the N200 amplitudes showed a significant interaction of chronotype × daytime on overall (*F*_1_ = 22.47, *P* < 0.001; *η*p^2^ = 0.45), congruent (*F*_1_ = 17.12, *P* < 0.001; *η*p^2^ = 0.39) and incongruent (*F*_1_ = 7.17, *P* = 0.012; *η*p^2^ = 0.21) trials for the electrodes Fz. The N200 component of both congruent and incongruent trials was larger at the circadian-preferred times in both groups (Fig. 5e, f), but the respective difference was significant only in ECs (incongruent: mean ± SEM_morning_, −0.38 ± 0.31 µV, mean ± SEM_evening_, 0.33 ± 0.36 µV; congruent: mean ± SEM_morning_, −0.59 ± 0.29 µV, mean ± SEM_evening_, 0.41 ± 0.38 µV). However, when we compared amplitude difference values from morning to evening in all participants, chronotype had a significant effect (incongruent: *F*_1_ = 7.57, *P* = 0.010; *η*p^2^ = 0.21; congruent: *F*_1_ = 14.49, *P* < 0.001; *η*p^2^ = 0.33) yielding higher negativity of N200 at circadian-preferred times in both groups. The same pattern of response was found for the Cz electrode and the N450 component of the Fz electrode (Supplementary Fig. 4c–f).

The next performed task was AX-CPT which is a lower cognitive-demanding task for measuring sustained attention (see “Methods”). Analysis of the behavioral data showed a significant interaction of chronotype × daytime on both accuracy (*F*_1_ = 14.16, *P* < 0.001; *η*p^2^ = 0.34) and RT (*F*_1_ = 19.39, *P* < 0.001; *η*p^2^ = 0.41). Both groups performed more accurately when the task was conducted at their circadian-preferred time. With regard to RT, only the LCs showed a significantly faster RT at their circadian-preferred time (Fig. 5g, h). The P300 serves as an attentional index to stimulus and memory storage, which are required in the AX-CPT. Analysis of this ERP component showed a significant interaction of chronotype** × **daytime on the P300 component at electrode Pz (*F*_1_ = 5.33, *P* = 0.028; *η*p^2^ = 0.16). Post hoc *t* tests indicated that the circadian-preferred time was significantly related to a larger P300 amplitude only in the LCs (LCs: mean ± SEM_morning_, 2.04 ± 0.49 µV, mean ± SEM_evening_, 2.74 ± 0.42 µV; ECs: mean ± SEM_morning_, 3.03 ± 0.50 µV, mean ±  SEM_evening_, 2.80 ± 0.44 µV) (Fig. 5i).

### No difference in subjective sleepiness rating and EEG marker of sleep pressure across groups

**Fig. 6:**
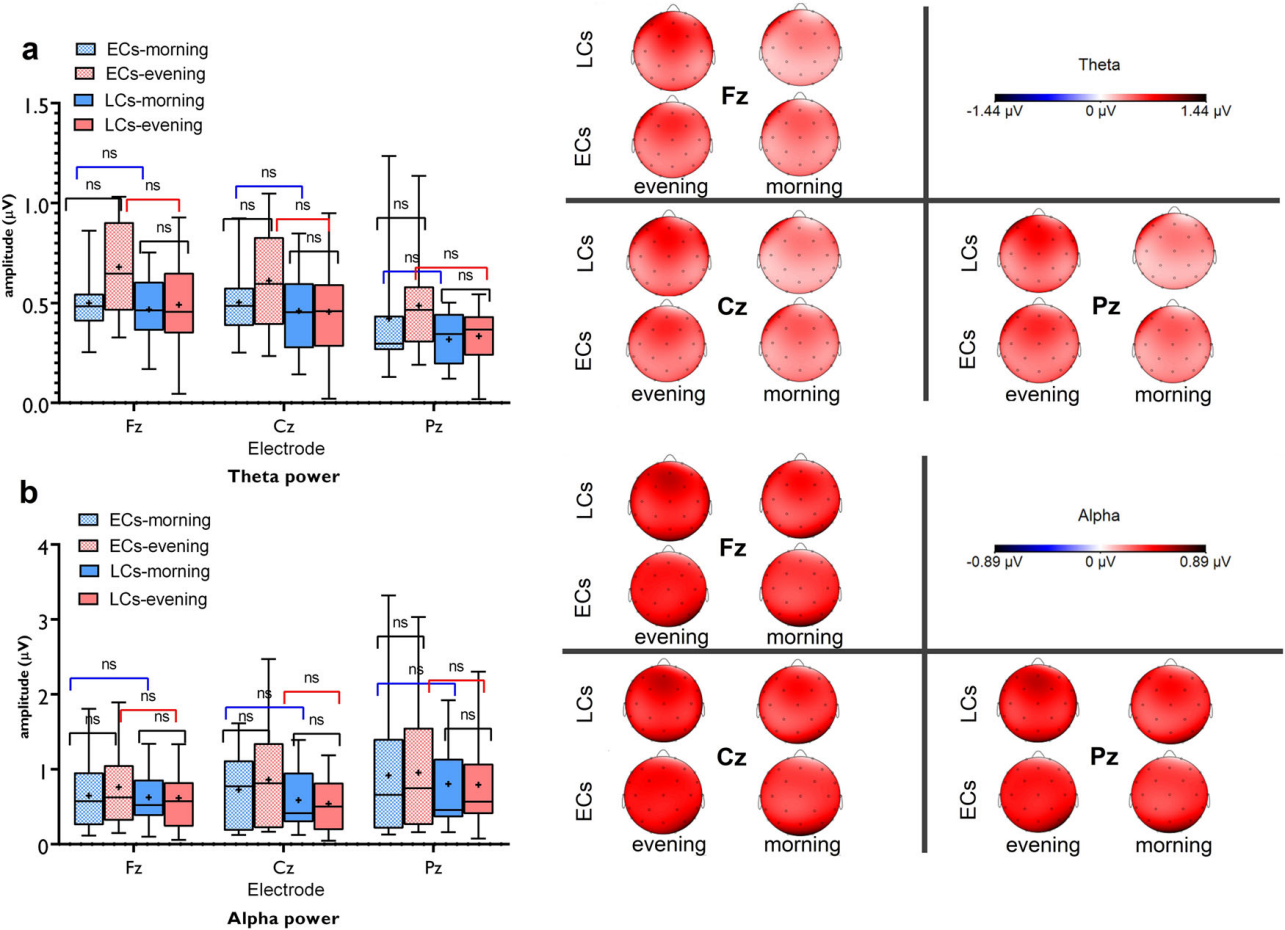
Resting-EEG theta and alpha oscillations at the circadian-preferred and non-preferred time for ECs and LCs. **a** The results of 3 (Fz, Cz, Pz electrodes) × 2 (chronotype) × 2 (daytime) ANOVA showed no significant interaction of electrode × chronotype × daytime (*F*_1.36_ = 1.65, *P* = 0.207) or electrode × chronotype (*F*_1.90_ = 0.20, *P* = 0.806) or electrode × daytime (*F*_1.36_ = 2.17, *P* = 0.142) on theta oscillations. The interaction of chronotype × daytime was marginally significant (*F*_1_ = 4.65, *P* = 0.040). Post hoc comparisons showed no significant difference of theta oscillations between groups in the morning (*t*_Fz_ = 0.36, *P* > 0.999; *t*_Cz_ = 0.53, *P* > 0.999; *t*_Pz_ = 1.22, *P* > 0.999) and evening (*t*_Fz_ = 2.42, *P* = 0.097; *t*_Cz_ = 1.88, *P* = 0.371; *t*_Pz_ = 1.86, *P* = 0.382). **b** For alpha oscillations, no significant interaction of electrode × chronotype × daytime (*F*_1.14_ = 0.30, *P* = 0.627) or electrode × chronotype (*F*_1.36_ = 1.17, *P* = 0.302), electrode × daytime (*F*_1.14_ = 0.35, *P* = 0.615) or chronotype × daytime (*F*_1_ = 1.68, *P* = 0.204) were found. Pairwise comparisons are calculated by post hoc *t* tests (paired, two-sided, *P* < 0.05). *n* = 30 (15 ECs, 15 LCs; data of one participant in each group are excluded due to noise). Data are presented as mean values ± SEM. The horizontal bar shows the median, the + shows the mean, the upper and lower boundaries show the 25th and 75th percentiles, respectively and the whiskers show the 5–95 percentile. ECs early chronotypes, LCs late chronotypes, ns nonsignificant, µV microvolt. [*] indicates a significant difference.

 All participants were moderate early and chronotypes which reduces the variability of sleep-wake cycle. However, potential sleep pressure at non-preferred times may still interfere with chronotype-specific effects on brain physiology and cognition. We did not directly control for the potential influence of sleep and sleep pressure, but used indirect measures of sleep pressure (e.g., subject sleepiness rating, EEG markers of sleep pressure). To this end, participants rated their sleepiness with the Karolinska Sleepiness Scale before each test session. All participants had at least 8 h of sleep before each session (see “Methods”). The results of ANOVA showed no significant interaction of chronotype × daytime (*F*_1_ = 1.03, *P* = 0.325) or main effects of chronotype and time of day. This indicates that there was no significant difference between the sleepiness ratings in or between both groups across different times of the day. Furthermore, we analyzed resting EEG theta oscillations, which is an objective marker of sleep pressure^51^ in both groups in the morning and evening. There were no significant differences in the theta oscillations at frontocentral electrodes (Fz, Cz, Pz) when we compared both groups in the morning and evening (Fig. 6a). No significant differences were neither observed in each group across different times of the day. The same pattern of results was observed for alpha oscillations (Fig. 6b).

## Discussion

**Fig. 7:**
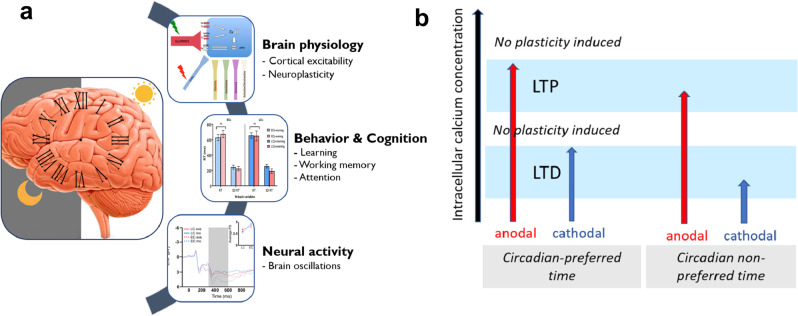
Chronotype, human brain physiology and cognition. **a** A schematic illustration depicting the converging impact of chronotype on brain physiology, behavior, and cognition. **b** Proposed mechanism of the neuroplasticity induction at circadian-preferred and non-preferred time based on the association between intracellular calcium concentration (*x* axis) and induction of tDCS-induced neuroplastic changes. Gradual enhancement of calcium concentration can either induce LTD, LTP, or no plasticity. The LTP-like plasticity induced by tDCS is linked to higher intracellular calcium concentration while LTD-like plasticity induced by tDCS is linked to a lower intracellular calcium concentration. It can be assumed that intracellular calcium concentration at the optimal time of the day is at an optimal level leading to stronger tDCS-induced LTP/D-like plasticity. This would be at least partially related to the higher glutamatergic and lower GABAergic activation at the circadian-preferred time, shown by cortical excitability data. LTP   long-term potentiation, LTD   long-term depression.

Previous studies have shown that chronotype has distinctive behavioral, physical, and genetic manifestations in humans^2^. The goal of this study was to determine how human cognition and related brain physiology are modulated by chronotype. Here, we show converging evidence of how chronotypes and time-of-day are associated with behavioral/cognitive performance of healthy individuals and demonstrate the physiological foundations of these effects by daytime-dependent cortical excitability, neuroplasticity, and brain information processing parameters (Fig. 7a). A specific causal effect of chronotype on these variables, however, cannot be definitely concluded unless the confounding influence of sleep pressure is controlled for directly.

The significantly higher cortical facilitation and lower cortical inhibition at the circadian-preferred time in both chronotypes argue for specific differences of cortical physiology mediated by chronotype and time of day. Specifically, our results suggest that at the circadian-preferred time, intracortical facilitation is enhanced predominantly by increased activity of glutamatergic synapses. Conversely, cortical inhibition is significantly pronounced at the circadian non-preferred time presumably through enhanced GABAergic activation. These results are in accordance with evidence from animal studies, which show a circadian regulation of GABA and glutamate^52–54^ highlighting the importance of excitatory and inhibitory systems in cortical excitability. Specifically, GABA is an important synchronizer of the suprachiasmatic nucleus, the major structure involved in circadian rhythms, whose activity varies throughout the day^55^. Studies in humans identified regulation of the GABAergic system, and respective alterations of cortical inhibition/facilitation within the circadian cycle as well^5,28^. Yet, chronotype effects on brain physiology were not specifically addressed in previous studies. Here, we demonstrate chronotype-specific modulation of cortical excitability.

In line with these results, we found that LTP/LTD-like neuroplasticity, which depends on the glutamatergic and GABAergic systems, is modulated by chronotype too. Evidence from primary motor cortex models in humans and animals show that tDCS-induced plasticity is driven by activation of NMDA receptors, and gated by reduction of GABA activity^20^. Specifically, anodal stimulation LTP-like after effect is thought to be caused by a major enhancement of NMDA receptor activity, while cathodal stimulation-generated LTD-like plasticity is suggested to involve a minor enhancement of NMDA receptors, driven by reduction of glutamate. Moreover, both kinds of plasticity seem to require reduction of GABA activity^23,41^. We argue that chronotype-specific differences of glutamatergic facilitation and GABAergic inhibition at circadian-preferred and non-preferred times, as described above, can explain the plasticity differences we obtained. A brain state of enhanced glutamatergic activity, and reduced GABAergic inhibition, as present at the circadian-preferred times of day for early and late chronotype,  would facilitate plasticity induction presumably via the optimal intracellular calcium concentration which determines the plasticity zones (Fig. 7b). In line with this, we demonstrate that active tDCS, compared to sham stimulation, induces LTP/LTD-like neuroplasticity depending on the circadian preference, which is associated with cortical excitability measures. These findings are consistent with previous animal studies that revealed a strong effect of the circadian clock on hippocampal plasticity^56^ and complement those of human studies that showed that plasticity response to a given paired-associative stimulation is regulated by circadian rhythms^16^.

The important question here is whether these chronotype-specific differences in brain plasticity influence learning and memory formation that depend on brain plasticity as well^57^. Providing proof of this, we found that motor sequence learning and retention, and their ERP components, follow the same chronotype-facilitating effect, and plasticity induction and motor learning at circadian-preferred times are associated. This observation makes sense because tDCS-induced neuroplasticity in the motor cortex and behavioral motor learning share intracortical mechanisms^17^. Furthermore, evidence from magnetic resonance spectroscopy shows a learning-specific reduction of GABA concentration in the motor cortex during motor learning^42^. In agreement with this, we found less GABAergic cortical inhibition at the circadian-preferred time which was associated with stronger motor learning at the behavioral level. Together, these convergent findings establish an important relevance of chronotype for motor cortex functionality from basic physiological functions (cortical excitability, LTP-like neuroplasticity) to behavioral and electrophysiological levels.

Cortical excitability alterations are expected to be associated with changes in behavioral and cognitive performance^13^. We found that important cognitive processing, including working memory and attentional functioning, show similar performance differences related to chronotype-dependent time-of-day indicating that modulatory effects of chronotype are extended to non-motor areas. Animal studies have demonstrated common mechanisms (e.g., neurotransmitter release, synaptic excitability, and neuronal activity) underlying circadian rhythm and memory formation^26^. For working memory, several circadian-related molecular mechanisms are proposed, including circadian clock-gated changes in glutamate receptor activity^58^. These mechanisms are in line with the enhanced cortical excitability caused by increased glutamatergic facilitation and LTP-like plasticity at the circadian-preferred time which we found in our excitability measures. Regarding attentional functioning, the peaks and troughs in circadian rhythms can differentially affect performance^6^. Our data are in agreement with these findings by showing an association between physiological plasticity and excitability data, which are indicative of daytime- and chronotype-dependent differences of glutamate and GABA activity, and cognitive performance of chronotype at respective times of day (for details of respective correlations see Supplementary Information). Moreover, task-specific ERP components were correlated with cognitive performance as well. The N200 and P300 components reflect stimulus identification/distinction and memory-updating processes^48^, suggesting a link between circadian preference and the physiological foundation of cognitive processing. These effects were more clear for the tasks with a higher cognitive load, such as the 3-back letter task, in line with findings of cognitive load-dependency of circadian effects^6^.

The findings of this study have specific implications for the field of human neurophysiology and cognitive neuroscience as well as broad implications for human behavior in healthy and clinical populations. “Time-of-day” and chronotype are important, but less-studied determinants of cortical plasticity induction by NIBS techniques^13,16^. Our results show that cortical excitability and neuroplasticity are strongly related to chronotype in humans. It is tempting to speculate that chronotype could account for variability in the efficacy of NIBS, and might explain partially heterogeneous effects in previous studies. This assumption may be likewise relevant for the performance of various cognitive tasks and suggest screening for chronotype or considering its modulatory effects. Being studied at the optimal time of day, having sufficient sleep and control of interfering factors might enhance the homogeneity of results, as our data suggest. Beyond genetic determinants^2^, chronotype is dependent on social pressures and our modern lifestyle that is increasingly deviating from the 24-h cycle. It can be misaligned in situations that impose unexpected changes on life such as a pandemic^59^. Given that chronotype has a clear effect on sleep timing^2^, it is highly relevant for working and educational environments. Working at a circadian-antagonistic time can disrupt the circadian cycle and thereby the shift workers’ health^60^. Learning materials and studying, which are dependent on learning, memory, and attention, can be hindered at circadian non-preferred times. At the clinical level, it can affect the therapeutic efficacy of NIBS and other interventions. For example, the ability to learn novel motor skills is central for the rehabilitation after a stroke, which could vary depending on patients’ chronotype, and timing of rehabilitation. Beyond the interactions with general well-being and some neuropsychiatric conditions^61^, circadian disruption is also linked to cardiometabolic disorders and the pathophysiology of neurodegenerative diseases (e.g., Parkinson’s and Alzheimer’s diseases)^60^ and should be considered in personalized medicine and timing of interventions for higher efficacy.

Our findings, though convergent at different levels, need to be interpreted with care. First, the observed findings cannot be solely attributed to chronotype. There are likely interindividual confounding effects due to sleep pressure that were not accounted for directly. These can be caused by different sleep–wake patterns of early and late chronotypes and the fact that experimental timing was fixed across groups. It is important to note here that our study question was whether brain physiology and cognition differ at different times of the day due to chronotype but not different levels of sleep pressure observed in different chronotypes. Therefore, we picked a fixed time of measurement based on sleep–wake cycle and level of activity across groups^62^ and did not individually adapt the experiment timing. Second, our measures of sleep pressure (e.g., subjective rating of sleepiness, resting EEG markers) are indirect. Direct and specific measures of sleep parameters and homeostasis (e.g., polysomnography) would be advantageous to reliably account for the confounding effect of sleep pressure. Finally, although we imposed at least 8 h of sleep before each experimental session and instructed participants to comply with this during the experimental course, it would be advantageous to extend this control during and before the course of the experiments to guarantee proper circadian entrainment, particularly in late chronotypes. Having that said, our findings still show a strong association of chronotype with human brain physiology and cognition, and importantly the observed pattern here differs from the same parameters of brain physiology and cognition under sleep pressure^63^.

Our physiological measures were based on the motor system and indirect measures of the involved neurotransmitters, yet they suggest systematic effects of chronotype at the whole-brain level. Interestingly, chronotype-specific individual differences in brain anatomy (e.g., grey matter volume) have been described by some studies^64^, which could affect tDCS-induced neuroplasticity induction either in a facilitatory or inhibitory way. It is unlikely that differences in brain structure were the driving force of the results because both groups showed enhanced plasticity at specific times of the day congruent with their respective chronotype. Furthermore, although there was no significant difference between baseline MEPs and % of MSO across conditions in both groups, which supports the reliability of the acquired data, the use of neuronavigation for stimulation of the motor cortex might have been advantageous to further enhance the reproducibility of the TMS coil position. Finally, although NIBS allows us to causally modify and induce neuroplasticity in humans non-invasively, it is worth acknowledging that the evidence for synaptic plasticity induction by NIBS, including tDCS, comes from animal and pharmacological studies, and is thus indirect^65–67^. In conclusion, our results show an association of circadian preference with learning and cognition including memory formation and attentional functions as well as the brain physiology underlying these cognitive processes including cortical excitability, neuroplasticity, and electrophysiological correlates of cognitive processes. These findings cannot be exclusively attributed to chronotype, but are likely affected by the interaction of chronotype and individual sleep–wake cycle.

## Methods

### Participants

Thirty-two healthy young adult volunteers (16 females, mean age=26.62 ± 5.01) qualified as early chronotype (ECs) or late chronotype (LCs) were recruited from the Technical University of Dortmund, Ruhr-University Bochum, and the surrounding community. Chronotype was determined based on the scores on the Morningness–Eveningness Questionnaire (DMEQ)^68^. Of 269 volunteers who completed the DMEQ during the course of the experiment, 69 were evening-types (6 definite evening types) and 35 were morning types (5 definite morning types). To limit variability between chronotypes in sleep timing and duration, only moderate chronotypes were included. Sixteen individuals from each chronotype (moderate type) who met the inclusion criteria were included in the ECs (8 females, *N* = 16) and LCs (8 females, *N* = 16) groups. The sample size was calculated a priori based on power analyses which showed that for a medium effect size (partial eta squared = 0.10) (suggested for NIBS studies^69^), a minimum of 24 subjects is required to achieve 95% power at an alpha of 0.05 for the primary statistical test of a mixed-model ANOVA design. We increased the sample size to 32 to fully counterbalance task order in each group (*N* = 16) and compensate for unforeseen variability and dropouts. As gender and age may explain variation in chronotype, we balanced participants’ gender and kept the age range to early adulthood. All participants were right-handed, non-smokers and underwent a medical screening to verify no history of neurological diseases, epilepsy or seizures, medication, metal implants, and current pregnancy. Each participant took part in a test TMS session to become acquainted with experiencing stimulation and understanding the study protocol. Female subjects were not examined during the luteal phase of the menstrual cycle (around week 3 following menstruation) to ensure that hormonal changes that affect cortical excitability^70^ would not interfere with their chronotype. Caffeine intake and other factors potentially interfering with cortical excitability (e.g., massive physical activity) were controlled before each experimental session and in case the respective criteria were not met, these sessions were postponed. This study conformed to the Declaration of Helsinki guidelines and was approved by the Institutional Review Board of the Leibniz Research Centre for Working Environment and Human Factors. Participants gave informed consent and received monetary compensation. They were free to withdraw at any time.

### Morningness–eveningness questionnaire (MEQ)

The German version of the MEQ^71^ (DMEQ)^68^ was used to identify chronotypes. It consists of 19 questions that ask individuals to determine their “feeling best” rhythms, indicate preferred clock time blocks rather than the actual real time for sleep and engagement in other daily/weekly activities (e.g., physical exercise, tests, work), and assess morning alertness, morning appetite, and evening tiredness. Each question has a score and the sum scores range from 16 to 86, with scores below 42 indicating evening type or late chronotype (LC), and scores higher than 58 indicating morning type or early chronotype (EC). Definite evening (16–30), moderate evening (31–41), intermediate or neutral (42–58), moderate morning (59–69), and definite morning (70–86) are the five chronotype categories identified by the MEQ. The questionnaire shows high reported reliability and has a significant correlation with circadian rhythm-related hormonal changes, including melatonin^68^. In addition to the MEQ, we defined the dichotomous chronotype phenotype by asking two identical questions (“Are you naturally a night person or a morning person?”)^2^. Individuals with discordant or neutral responses to both were excluded. The mean scores of chronotype for the early and late chronotypes were 62.25 ± 3.47 and 35.37 ± 4.31, showing that both groups represent moderate early and late chronotypes.

### Experiment timing, sleep/wake cycle, and light variation

Timing of the experiments was determined based on the average of sleep times and level of activity of early and late chronotypes based on previous studies^6,62^ as well as daily life routine schedules. For morning sessions, the start time was chosen based on the norm start of schooling and working (8:00 in the morning). This is also a time when both chronotypes have the lowest levels of activity^62^. The evening time (19:00) was specified based on the time when both chronotypes have a comparable level of activity according to previous dataset profiles^62^. Both groups were instructed to go to bed (at around 22:00–23:00), and get up in the morning at identical times of the day, and have at least 8 hours of sleep for morning sessions (mean_ECs_ = 8.41 h, mean_LCs_ = 8.11 h). For the evening session, participants were allowed to go to bed at their preferred time but before 24:00 and were also allowed to have more than 8 hours of sleep if needed (mean_ECs_ = 8.36 h, mean_LCs_ = 8.38 h). All participants were strongly recommended to comply with the sleep/wake cycle instruction and in case of irregularities, poor quality of sleep, and sleepiness (measured by participants’ rating before each session), the experimental session was postponed to another day. Subjective sleepiness of participants and their alertness were evaluated with the Karolinska sleepiness scale (KSS)^72^ which measures sleepiness in a 1–10 Likert-type scale (Supplementary Information). No significant difference was observed between the sleepiness ratings of both early and late chronotypes in the morning sessions (mean_ECs_ = 3.2, mean_LCs_ = 3.71; *t* = 1.61, *P* = 0.11) and evening sessions (mean_ECs_ = 3.58, mean_LCs_ = 3.65; *t* = 0.25, *P* = 0.80). Due to the variability of seasonal light, experimental sessions did not take place in summer when the difference between sunrise and sunset is largest in Germany. For other months, the experiment was scheduled to be randomly distributed across other seasons in both groups. Furthermore, ambient light was kept constant with a standard exposure intensity for indoor light (around 500 lux) across all experimental sessions in the morning and evening. In the cognitive sessions, tasks and EEG recordings were conducted in a soundproof shielded room with a constant temperature of 24–25 °C for morning and evening sessions.

### Cortical excitability monitoring with TMS

Single-pulse and paired-pulse TMS protocols of resting motor threshold (RMT), active motor threshold (AMT), I–O curve, short intracortical inhibition and facilitation (SICI-ICF), intracortical I-wave facilitation, and short-latency afferent inhibition (SAI) were used to monitor corticospinal and intracortical excitability in the motor cortex. RMT, AMT, and I–O curve examine corticospinal excitability, SICI-ICF measures both, intracortical facilitation and inhibition, and intracortical I-wave facilitation and SAI are measures of intracortical inhibition of the human motor cortex^31,32,36^.

#### Single-pulse MEP, resting and active motor threshold

Single-pulse biphasic TMS at 0.25 Hz ± 10% (random) was delivered by a PowerMag ppTMS magnetic stimulator (Mag&More, Munich, Germany) through a figure-of-eight magnetic coil (diameter of one winding, 70 mm; peak magnetic field, 2 T) held 45° to the midline and applied over the left primary motor cortex. Surface MEPs were recorded from the right abductor digiti minimi muscle (ADM) with gold cup electrodes in a belly-tendon montage. Signals were amplified, and filtered (1000; 3 Hz–3 KHz) using D440-2 (Digitimer, Welwyn Garden City, UK) and were digitized (sampling rate, 5 kHz) with a micro 1401 AD converter (Cambridge Electronic Design, Cambridge, UK), controlled by Signal Software (Cambridge Electronic Design, v. 2.13). The RMT was examined using the TMS Motor Threshold Assessment Tool (MTAT 2.0, http://www.clinicalresearcher.org/software.htm)^73^ and was determined as the lowest stimulator intensity required to evoke a peak-to-peak MEP of 50 µV in the relaxed ADM muscle in at least five out of ten consecutive trials. The AMT was determined as the lowest stimulator intensity required to elicit MEP response of ∼200–300 μV during moderate tonic contraction of the right ADM muscle (∼20% of the maximum muscle strength)^74^ in at least three of six consecutive trials.

#### Input–output curve (I–O curve)

The I–O curve is a TMS single-pulse protocol that reflects the excitability of corticospinal neurons. It is modulated by glutamatergic activity and refers to the increase of MEP amplitudes with increasing TMS intensity^32^. The slope of the recruitment curve increases at higher TMS intensities with higher glutamatergic and adrenergic transmission and decreases by drugs that enhance effects of GABA^32,35^. In the I–O curve protocol, MEP amplitudes in the relaxed right ADM muscle were measured in four blocks with different stimulus intensities (100%, 110%, 130%, and 150% RMT)^75^, each block with 15 pulses, and a mean (MEP amplitudes) was calculated for each intensity.

#### Short-latency intracortical inhibition and intracortical facilitation (SICI-ICF)

The SICI-ICF is a TMS paired-pulse protocol for monitoring of GABAergic-mediated cortical inhibition and glutamate-mediated cortical facilitation^32^. In this protocol, a subthreshold conditioning stimulus (determined as 70% of AMT) is followed by a suprathreshold test stimulus which was adjusted to evoke a baseline MEP of ∼1 mV. The paired stimuli are presented in interstimulus intervals (ISI) of 2, 3, 5, 10, and 15 ms^36^. ISIs of 2 and 3 ms represent short-latency intracortical inhibition (SICI) and have inhibitory effects on test pulse MEP amplitudes, and ISIs of 10 and 15 ms represent intracortical facilitation (ICF) and have enhancing effects on single-pulse TMS-elicited MEP amplitudes^36,76,77^. The stimuli (subthreshold and suprathreshold stimuli) were organized in blocks in which each ISI and one single test stimulus were applied once in pseudorandomized order. Each block was repeated 15 times, which resulted in a total of 90 single-pulse or paired-pulse MEP per session. The exact interval between the paired pulses was randomized (4 ± 0.4 s).

#### Short-interval intracortical I-wave facilitation

This TMS protocol is based on I (indirect) waves which refer to high-frequency repetitive discharges of corticospinal neurons produced by single-pulse stimulation of the motor cortex^78^ (for a detailed review see refs. ^37,78^). In this protocol, two successive stimuli (supra- and subthreshold) are separated by short ISIs, but this protocol involves a suprathreshold first stimulus and a subthreshold second stimulus^37^. The ISIs range from 1.1 ms to 4.5 ms latency and are presented in pseudorandomized order. We grouped ISIs to early (mean MEP at ISIs 1.1, 1.3, 1.5 ms), middle (mean MEP at ISIs 2.3, 2.5, 2.7, 2.9 ms), and late (mean MEP at ISIs 4.1, 4.3, 4.5 ms) epochs. The intensity of the first conditioning suprathreshold stimulus (S1) is adjusted to produce a baseline MEP of ∼1 mV when given alone and is followed by a second subthreshold stimulus (S2) that was set to 70% of RMT)^75^. For each ISI, 15 pulses were recorded. Another 15 pulses were recorded for the control MEPs, in which the suprathreshold stimulus (S1) was given alone and adjusted to achieve a baseline MEP of ∼1 mV. The pairs of stimuli were organized in blocks in which each ISI and one test pulse was represented once, and were pseudorandomized. This TMS paired-pulse protocol (a first suprathreshold stimulus and a second subthreshold stimulus) has facilitatory effects on MEP peaks^37^ that occur at ISIs of about 1.3, 2.6, and 4.2 ms. This effect is suggested to be produced as a result of elicited I-waves (indirect waves: descending volleys produced by indirect activation of pyramidal tract neurons via presynaptic neurons) by the subthreshold S2 and is controlled by GABA-related neural circuites^35,37,79^

#### Short-latency afferent inhibition (SAI)

SAI is a TMS protocol coupled with peripheral nerve stimulation and is based on the concept that peripheral somatosensory inputs have an inhibitory effect on motor cortex excitability at short intervals (e.g., 20–40 ms)^38^. SAI has been linked with cholinergic^31^ and GABAergic systems^33^ at the cortical level. In this protocol, single-pulse TMS serves as test stimulus and is adjusted to evoke a MEP response with a peak‐to‐peak amplitude of ~1 mV. The conditioning afferent stimuli were single pulses (200 µs) of electrical stimulation applied to the right ulnar nerve at the wrist level (cathode proximal) through bipolar electrodes connected to a Digitimer D185 stimulator (Digitimer Ltd., Welwyn Garden City, UK). The conditioning afferent stimuli were applied with an intensity of ~2.5–3 times of perceptual threshold adjusted to evoke a minimal visible twitch of the thenar muscles^31^, followed by a single TMS pulse (test stimulus) applied over the motor cortical representation of the right ADM. The stimuli were applied in blocks containing the test stimulus alone (control condition) and two paired-stimuli blocks with ISIs of 20 and 40 ms in pseudorandomized order. Each block was repeated 20 times, resulting in a total of 60 trials.

#### Experimental procedure

Cortical excitability monitoring sessions took place once in the morning and once in the evening at the same fixed time for all study sessions. Measurements were scheduled to start at 8:30 am for the morning session and 7:00 pm for the evening session after the preparation stage (motor cortex hotspot identification, RMT, and AMT determination procedures). There was a 1-week interval between each session. Participants were instructed not to consume caffeine, alcohol, or engage in strenuous physical activities 24 h prior to each session to ensure a stable level of motor-cortical excitability. In each session, participants were seated comfortably in a reclining chair, with a pillow resting under the right arm and a vacuum pillow around the neck to prevent head movement. First, the hotspot (the coil position over the primary motor area that produces the largest MEP in the right ADM with a given medium TMS intensity) was identified with TMS. The stimulation intensity was then adjusted to evoke MEPs with a peak-to-peak amplitude of an average of 1 mV, as described above. Following this step, RMT and AMT were obtained as described above. A 10 min break was allowed after recording AMT in order to avoid an effect of muscle contraction on the next measurements. After the break, the following TMS protocols were measures to monitor cortical excitability: SAI, SICI-ICF, I-wave facilitation, and I/O curve. The order of measures was randomized except for the SAI which was always the first measure as it required a preparation time of about 10 min which was scheduled to take place during the 10-min break. This was done to keep the length of the session as close as possible to tDCS sessions and maintain participants in their circadian-preferred and no-preferred times. In the case of single test pulse-generated MEP alterations of >20% during the session, stimulation intensities were adjusted^80^. Each cortical excitability session took 60–70 min. All TMS protocols were conducted with a PowerMag ppTMS magnetic stimulator (Mag & More, Munich, Germany) through a figure-of-eight magnetic coil (diameter of one winding, 70 mm; peak magnetic field, 2 T) held 45° to the midline and applied over the left primary motor cortex.. The device was equipped for the application of both single-pulse TMS and paired-pulse TMS.

### Neuroplasticity induction with tDCS

#### Direct current stimulation of the motor cortex

Electrical direct current was applied through a pair of saline-soaked surface sponge electrodes (35 cm^2^) and delivered through a battery-driven constant current stimulator (neuroConn GmbH, Ilmenau, Germany). The target electrode was fixed over the motor-cortical representation area of the right ADM as identified by TMS, and the reference electrode was placed over the contralateral supraorbital area. The distance on the scalp between the edges of the electrodes was kept at a minimum of 6 cm to reduce shunting of current through the scalp^81^. Based on the randomized condition, anodal, cathodal, or sham tDCS with 1 mA intensity were applied for 7 min with 15 s ramp up/down at the beginning and end of stimulation. For the sham condition, stimulation was delivered for 30 s, with a 30 s ramp up and down. Using this procedure, participants are not able to distinguish between real and sham tDCS^82^. We simulated electrical current flow in the head induced by this protocol to show how the primary motor cortex is affected by tDCS (Fig. 3a). The model is based on the standard head model which does not take into account potential structural differences (e.g., white matter) in the brains of different chronotypes^64^. The model should thus be taken into account for illustrative purposes only.

#### MEP monitoring from TMS

Single-pulse MEPs were obtained in the same manner as described in the previous section, and TMS intensity was set to evoke MEPs of ~1-mV peak-to-peak amplitude.

#### Experimental procedure

After screening participants for suitability for tDCS, each participant attended six sessions of tDCS (morning anodal, morning cathodal, morning sham, and evening anodal, evening cathodal, evening sham) in randomized order. TDCS sessions started at a fixed time in the morning and evening and there was a 1-week interval between sessions. Morning sessions started at 8:00 am and evening sessions at 6:30 pm; starting time of tDCS was scheduled to take place around 8:30 in the morning session and 7:00 in the evening session, following the preparation stage which took ~20–30 min. In each session, participants were seated comfortably in a reclining chair, with a pillow positioned under the right arm and a vacuum pillow around the neck to prevent head movement. At the beginning of each session, baseline cortical excitability was measured by first inducing MEPs over the left M1 to identify the region which produced the largest MEP of the target muscle (right ADM) with a given TMS intensity. The region was then marked, and subsequent pulses were delivered from this optimal position. Stimulation intensity was adjusted to reach a peak-to-peak MEP amplitude of 1 mV (SI1mV), which was then used for the remaining measurements. Following a baseline measurement of 25 MEPs, 7 min of anodal, cathodal, or sham stimulation was delivered, as described above. After removal of the tDCS electrodes, MEP measurements were conducted immediately in epochs of every 5 min up to 30 min after tDCS (7 total epochs). This tDCS protocol produces polarity-specific short aftereffects that fade away before 30 min after stimulation^40^. At the end of each session, participants completed a side effect survey asking to rate the presence and severity of visual phenomena, itching, tingling, burning, and pain during stimulation each on a 0-5 Likert scale and also to guess the stimulation intensity they received (i.e., 0 mA intensity or 1 mA intensity). Each tDCS session took around 60 min.

### Behavioral measures: motor learning and cognitive tasks

We used one specific behavioral task to measure motor learning which involves critically the primary motor cortex, including LTP-like plasticity of this region^17^. In addition, participants performed three cognitive tasks to monitor working memory and attentional functioning.

#### Serial reaction time task (SRTT)

The SRTT is a motor sequence learning task. Performance on this task is associated with increased activity and cortical excitability of the motor, premotor and supplementary motor areas and early learning affects primarily the primary motor cortex^57,83,84^. In this task, sequence motor learning is indicated by a reduction of reaction time to press the appropriate button on a response box after the presentation of a visually cued stimulus on a computer screen. Participants are instructed to push the respective button with the respective finger of the right hand (index finger for Button 1, middle finger for Button 2, ring finger for Button 3, and little finger for Button 4). A visual cue (here a black dot) appears at one of four positions arranged horizontally on a computer screen. Each screen position corresponds to the respective button of the response box. Participants are instructed to press the button corresponding to the position of the dots as fast as possible. The duration of each trial is defined by the participant’s response time (RT) and the task duration is ~15–20 min. At the end of each stimulus–response pair, there is a 500 ms delay before the next cue is presented. The task consists of eight blocks of 120 trials each (960 trials in total). In blocks 1 and 6, the sequence of dots follows a pseudorandom order in a way that dots are presented equally frequently in each position and never in the same position in two subsequent trials. During fixed-sequence blocks (blocks 2–5, 7–8), the stimuli appear according to a fixed 12-item sequence, which is repeated 10 times (e.g., A–B–A–D–B–C–D–A–C–B–D–C). The averaged RT difference in block 5 (sequence order) vs block 6 (random order) is the primary measure of motor learning acquisition as it indicates the response to sequence learning vs sequence learning-independent performance. The RT difference between block 6 (random order) and block 7 (sequenced order) is suggested to indicate additionally learning retention. In addition to RT, which is the major indicator of implicit motor learning, RT variability and accuracy were also calculated as outcome variables. Participants were not told about the repeating sequence and at the end of the session, they were asked whether they noticed a sequence and if so, to write the sequence in order to assess explicit learning of the task. In such cases, respective data were excluded from the final analysis. Two different sequences of the task, with no overlapping parts, and comparable difficulties, were presented in the two sessions in a counterbalanced order.

#### Cognitive tasks

*3-back letter task*. The WM task in our study involved a letter variant version of the n‐back task in which subjects should indicate whether a letter presented on the screen (the “target letter”) matched the letter previously presented (the “cue” letter)^85^. Here, we used a 3-back version of task^86^ in which “Hits” (correct responses) were defined as any letter identical to the one presented three trials back. Stimuli were pseudorandom sequences of 10 letters (A–J) presented at a fixed central location on a computer screen. Each letter was visible for 200 ms with an 1800 ms interstimulus interval, making the difficulty level of the task high. The letters were presented in black on a white background and subtended 2.4 cm (when viewed at 50 cm eye-to screen distance). Participants completed two blocks consisting of 44 (practice block) and 143 trials (main block), respectively, resulting in a total number of 187 trials. A short break (5–20 s) between blocks was provided to allow participants to rest. Two different versions of the task were employed in two sessions (morning session and evening session) and condition order was randomized across participants. Reaction times and accuracy measures were obtained for each trial.

*The Stroop color-word task*. The Stroop interference task is a neuropsychological test extensively used for measuring selective attention, cognitive inhibition, and information processing speed^87,88^. We used a computerized Stroop color/word test similar to the Victoria version, based on the previous studies^88^. In the Stroop word, the color names were written in black and in the Stroop color, some XXXs were presented in red, green, yellow, and blue ink and participants had to respond with the correspondent keys. In the Stroop color-word task, which was of our specific interest, participants are presented with either “congruent” or “incongruent” color words. In the incongruent trials, the color of the ink in which the word was displayed was different from the meaning of the word (for example, the word “red” was written in blue) while in the congruent trials both, word and color of the ink were identical. Stimuli were presented on a screen with a black background for 2000 ms with a 500 ms interstimulus interval. The size of the stimuli was 1.4 cm at ~50 cm eye-to-screen distance. A response box with only four keys, colored in red, blue, yellow, and green, was placed in front of the subjects and subjects had to press the corresponding key of the color in which the word was written. We increased the number of congruent and incongruent trials in the color-word task, as compared to the Stroop word and Stroop color blocks, to increase the power of the EEG analyses. In total, the Stroop interference block included 40 congruent and 120 incongruent trials, resulting in a total of 160 trials.

*AX-continuous performance test (AX-CPT)*. The AX-CPT is used for assessing attentional functioning (sustained or transient attention), or executive control, depending on the applied versions, which include baseline, proactive control, and reactive control^89,90^. Here, we used the baseline version of the task, which is shorter (~15 min), less demanding, and measures transient attention^90^. Visual stimuli were white letters on a dark background appearing one at a time on a computer screen for 150 ms each with a 2000 ms interstimulus interval. Subjects were instructed to press a button with the right index finger whenever the letter A (correct cue) was followed by the letter X (correct target) as quickly and accurately as possible. All other sequences were to be ignored, including sequences in which an incorrect cue (designated “B”, but comprising all letters other than A or X) was followed by the target letter (X), or sequences in which a correct cue (A) was followed by an incorrect target (designated “Y”, but comprising all letters other than A or X). The AX sequences are presented with a high probability, to guarantee a strong response bias. The tasks consisted of 240 pairs of letters (480 trials) with 40% “AX”, 40% “BY”, 10% “BX”, and 10% of “AY”. Accuracy and RT were recorded for the target trials.

#### Procedure

Participants performed the tasks in two randomly assigned sessions in the morning and evening at the same time the previous sessions took place with at least 1-week interval. The order of tasks was counterbalanced across participants, with the exception of the SRTT, which was always conducted first and was scheduled to begin around the time cortical excitability monitoring and tDCS were applied. All tasks (SRTT, N-back, Stroop, and AX-CPT) were presented on a computer screen (15.6″in. Samsung) via E-prime software^91^, at the viewing distance from the monitor was ~50 cm. The tasks were conducted in a soundproof electromagnetic shielded room during EEG recording.

### EEG

#### EEG recording

EEG was recorded continuously during cognitive task performance from 30 scalp electrodes positioned according to the international 10–20 system using the NeurOne Tesla EEG amplifier (Bittium, NeurOne, Bittium Corporation, Finland) with a sampling rate of 1000 Hz. The electrodes included: Fp1, Fp2, Fz, F3, F4, F7, F8, Fc1, Fc2, Fc5, Fc6, Cz, C3, C4, T7, T8, Tp9, Tp10, Cp1, Cp2, Cp5, Cp6, Pz, P3, P4, P7, P8, Iz, O1, and O2, and were mounted on the head with a cap (EASYCAP GmbH, Herrsching, Germany). The reference electrode was positioned on FCz, and the ground electrode was placed at the AFz position. The electrodes were connected to the head using high-viscosity electrolyte gel (SuperVisc, Easycap, Herrsching, Germany). All impedances were kept below 10 kΩ throughout the experimental sessions. EEG data were collected in a shielded room and no observed spectral peaks at 50 Hz. Raw EEG data were recorded and stored for offline analysis using BrainVision Analyzer 2.1 (Brain Products GmbH, München, Germany). EEG recording included resting-state measurement, and consisted of eyes open and closed states alternating every 60 s for 4 min, and task-based measurement.

#### EEG data preprocessing and analysis

EEG recordings were band-pass filtered offline between 1 and 30 Hz (48 dB/Octave) and re-referenced to an average reference. The VEOG signal via the Fp2 channel was used for dealing with eye movement artifacts in ERP recordings using the Gratton and Coles method^92^ embedded in the BrainVision Analyzer 2.1. EEG data were then time-locked to the stimulus of interest onset in each task. Epochs started 100 ms before the stimulus onset and ended 700 ms after stimulus onset in the SRTT, 100 ms before the target onset and ended 1000 ms after target onset in the 3-back and AX-CPT tasks, and 100 ms before the stimulus onset and ended 1000 ms after stimulus onset in the Stroop task (both congruent and incongruent trials). Epochs were baseline-corrected using a -100-0-ms time window. Artifacts were identified using a combination of automated (artifacts greater than 100 µV peak-to-peak) and manual selection processes. Segments were removed based on this automatic selection, and visual inspection to identify artifacts due to other sources of non‐neurogenic activity. The remaining epochs were averaged for calculating the average ERP. The average ERP of blocks 5, 6, 7 in the SRTT task was based on 120 trials per block. In the N-back and AX-CPT tasks, the average ERP of hits (correct response) were based on 40 and 96 trials, respectively. In the Stroop task, the average ERP of congruent and incongruent trials was based on 40 and 120 trials, respectively. For the analyses, the following averaged components were investigated: (1) the P300 at electrode Pz, Cz, and P3 within a time window of 250–500 ms after stimulus onset in the SRTT learning blocks (blocks 5, 6, 7), (2) the P300 at electrode Fz and Cz within a time window of 300–600 ms^93,94^ after target stimulus onset in the 3-back task, (3) the N200 and N450 at electrode Fz and Cz within time windows of 200–300 ms and 400–550 ms, respectively, after congruent and incongruent trials onset^50^ in the Stroop task, and (4) the P300 at electrodes Fz and Cz within a time window of 300–600 ms^93,95^ after target onset (when target letter X was preceded by cue A) in the AX-CPT task. The time windows were selected based on previous studies and designated as the maximum positive or negative deflection occurring at the post-stimulus latency window. EEG resting-state data was preprocessed in the manner explained above. Here, the data were segmented into 2-s epochs. A fast Fourier transform analysis (Hanning window length: 10%) was performed on the epochs to obtain spectral power levels in the beta (13–30 Hz), alpha (7–13 Hz), theta (4–7 Hz), and delta (1–4 Hz) range.

### Statistical analysis

Data analyses were conducted with the statistical package SPSS, version 26.0 (IBM, SPSS, Inc., Chicago, IL) and the GraphPad Prism 8.2.1 (GraphPad Software, San Diego, California). Figures were created using Microsoft PowerPoint and Prism 8.2.1. Main analyses were conducted using the mixed-model ANOVAs with both between-and-within-subject factors. Mauchly’s test of sphericity was conducted, and the Greenhouse–Geisser correction was applied when necessary. The normality and homogeneity of variance of the data collected from different measures were confirmed by Shapiro–Wilk and Levin tests, respectively. The sample size was predetermined using power analysis (see “Participants” section).

#### Cortical excitability data analysis

For the TMS protocols with a double-pulse condition (i.e., SICI-ICF, I-wave facilitation, SAI), the resulting mean values were normalized to the respective test pulse. First, mean values were calculated individually and then interindividual means were calculated for each condition. For the I–O curves, absolute MEP values were used. To test for statistical significance, mixed-factorial ANOVAs with repeated measures (ISI × daytime × chronotype) were performed with ISIs, TMS intensity (in I–O curve only), and time of day (morning vs evening) as within-subject factors, chronotype (ECs vs LCs) as the between-subject factor, MEP amplitude as the dependent variable and age, gender and BMI as covariates. In case of significant results of the ANOVA, post hoc comparisons were performed using Bonferroni-corrected post hoc *t* tests to compare mean MEP amplitudes of each condition against the baseline MEP and to contrast circadian-preferred vs circadian non-preferred times within and between groups. To determine if single-pulse conditions differed across time of day, they were entered as dependent variables in a mixed-factorial ANOVA with the time of day (morning, evening) as a within-subject factor and chronotype (ECs vs LCs) as a between-subject factor. The mean values of the single-pulse conditions (control condition) did not differ between morning and evening sessions for either group in SICI-ICF, I-wave facilitation, and SAI (Supplementary information).

#### tDCS-induced neuroplasticity data analysis

The peak-to-peak amplitude of the 25 MEPs obtained for each timepoint (BL, 0, 5, 10, 15, 20, 25, 30 min after tDCS) was calculated and averaged together for each tDCS condition in the morning and evening. To determine if individual baseline tmeasures differed between session, SI1mV and Baseline MEP were entered as dependent variables in a repeated measures ANOVA with the session (six levels) as a within-subject factor and chronotype (ECs vs LCs) as a between-subject factor. Baseline MEP amplitudes (absolute values) for each tDCS condition did not significantly differ (Supplementary Table 3). The mean MEP amplitude for each measurement timepoint was normalized to the session’s baseline (individual quotient of the mean from the baseline mean) resulting in values representing either increased (>1.0) or decreased (<1.0) excitability. Individual averages of the normalized MEP from each timepoint were then calculated and entered as dependent variables in a mixed-factorial design with repeated measures ANOVA (stimulation × timepoint × daytime × chronotype) with stimulation condition (anodal, cathodal, sham), timepoint (eight levels), and time of day (morning vs evening) as within-subject factors and chronotype (ECs vs LCs) as between-subject factor and age, gender, and BMI as covariates. In case of significant ANOVA results, post hoc comparisons of MEP amplitudes at each timepoint were performed using Bonferroni-corrected post hoc *t* tests to examine if an active stimulation resulted in a significant difference relative to sham (comparison 1), baseline (comparison 2), the respective stimulation condition at circadian-preferred vs circadian non-preferred times (comparison 3), and the between-group comparisons at respective timepoints (comparison 4).

#### Behavioral data analysis

Means of RT and accuracy for SRTT blocks 5, 6, and 7 were calculated. Trials with wrong responses, as well as those with RTs of <150 ms^96,97^ or >3000 ms, and trials which deviated by 3 standard deviations or more from the average individual response time, were discarded^98^. One participant could identify the stimuli sequence and the respective data were excluded from the analysis. Mean RTs were standardized to Block 1 for each subject at each measurement time separately. The standard deviation of RTs for each subject and learning block was calculated as an index of variability of RTs. The mean RT, RT variability, and accuracy of blocks were entered as dependent variables in mixed-factorial ANOVA with repeated measures (block × daytime × chronotype) with blocks (5, vs 6, 6 vs 7) and time of day (morning vs evening) as within-subject factors and chronotype (ECs vs LCs) as between-subject factor. Because the RT differences between blocks 5 vs 6 and 6 vs 7 were those of major interest, post hoc comparisons were performed on RT difference between these blocks using paired-sample *t* tests (two-tailed, *P* < 0.05) without correction for multiple comparisons. For 3-back, Stroop and AX-CPT tasks, mean, and standard deviation of RT and accuracy were calculated and entered as dependent variables in mixed-factorial ANOVAs (daytime × chronotype) with the time of day (morning vs evening) and chronotype as within-subject and between-subject factors, respectively. Age, gender, and BMI were entered as covariates in all ANOVAs. For significant ANOVA results, post hoc comparisons of dependent variables across time of day (morning vs evening) were performed using paired-sample *t* tests (two-tailed, *P* < 0.05) without correction for multiple comparisons, since we compared only two conditions.

#### Correlational analyses

To assess the relationship between induced neuroplasticity and motor sequence learning, and the relationship between cortical excitability and cognitive task performance we used bivariate linear regression analysis (Pearson’s correlation, one-tailed). For the first correlation, we used individual grand-averaged MEP amplitudes obtained from anodal and cathodal tDCS pooled for the timepoints between 0, and 20 min after interventions, which showed plastic responses in all conditions at preferred times of day, and individual motor learning performance obtained from block 5 vs 6 RT differences for both, the circadian-preferred (morning for ECs, evening for LCs) and non-preferred (evening for ECs, morning for LCs) times. For the second correlation, we used individual grand-averaged MEP amplitudes obtained from each TMS protocol and individual accuracy/RT obtained from each task both at the circadian-preferred and non-preferred times.

#### EEG data analysis

EEG data preprocessing and analysis were described in the previous section. For all tasks, individual ERP means were grand averaged and entered as dependent variables in mixed-factorial ANOVAs (daytime × chronotype) with the time of day (morning vs evening) as within-subject and chronotype (ECs vs LCs) as between-subject factors. No correction was used for investigating multiple electrode locations. EEG data of one participant in the Stroop task which were not recorded properly due to technical difficulties and of another participant whose SRTT behavioral data was excluded were discarded.

### Reporting summary

Further information on research design is available in the Nature Research Reporting Summary linked to this article.

## Supplementary information

Supplementary information

Peer Review File

Reporting summary

## Data Availability

The datasets generated and/or analyzed during the current study are not publicly available due to institutional regulations, ethics, and confidentiality agreements, but are available from the corresponding author upon reasonable request. Source data are provided with this paper.

## Source data

Source data
