# Supplementary material for: Cognitive functions and underlying parameters of human brain physiology are associated with chronotype
Source: Nat Commun. 2021 Aug 3;12:4672. doi: 10.1038/s41467-021-24885-0 (PMC8333420; doi:10.1038/s41467-021-24885-0)
Supplement: Supplementary file 1 — Supplementary information [file 41467_2021_24885_MOESM1_ESM.pdf]

Supplementary information

Supplementary Results

1. *Cortical excitability*

1.1.TMS protocols threshold values

MEP values of single-pulse conditions and other control conditions, as well as subject-specific baseline sensitivity to TMS (defined as the percentage of maximum stimulator output required for the SI1mV MEP amplitude), are summarized in Supplementary Table 1. For each TMS protocol, we compared single-pulse condition MEPs (control condition) and the SI1mV obtained at different daytimes by a 2-factorial ANOVA with daytime (morning, evening) as the within-subject and chronotype (ECs, LCs) as the between-subject factor. The results of the respective ANOVAs show that the mean values of the single-pulse conditions MEP, MEP amplitude at RMT (for the I-O curve protocol), %MSO for RMT, and SI_{1mv} did not significantly differ between time of day and groups (Supplementary Table 2). Only for AMT, the interaction of chronotype×daytime was significant, and post hoc t-tests (two-tailed, $p<0.05$) revealed a significant difference between AMT values only for ECs across daytime (t = 3.58, p = 0.003).

2. *Neuroplasticity supplementary results*

2.1. Baseline MEP difference

The average baseline MEPs (absolute value) as well as the TMS stimulus intensity for 1 mV amplitude (SI1mV) obtained for each tDCS condition (anodal, cathodal, sham) are summarized in Supplementary Table 3. Baseline MEP obtained from all stimulation conditions (6 values) for both groups were analyzed with a 2 × 6 factorial ANOVA. The results show no significant interaction of chronotype×baseline MEP ($F_{4.39}$= 1.68, p= 0.150) and no main effects of baseline MEP ($F_{4.39}$= 1.88, p= 0.111) and chronotype (F_1= 0.39, p= 0.535). A similar 2 × 6 factorial ANOVA was conducted for SI1mV amplitudes (6 values), and no interaction of chronotype×SI1mV ($F_{2.88}$= 1.77, p= 0.160) and no main effects of SI1mV ($F_{2.88}$= 0.97, p= 0.440) and chronotype (F_1= 0.01, p= 0.933) were found.

2.2. Reported tDCS side effects

Supplementary information

The reported side effects during each tDCS session for both ECs and LCs are summarized in Supplementary Table 4. The results of the ANOVA conducted for each side effect show a significant effect of tDCS on tingling and burning sensation during stimulation, but no significant effects for visual phenomena, itching, and pain (Supplementary Table 5). Pairwise comparisons of tingling ratings with post *hoc* t-tests revealed significant differences between morning anodal vs morning sham in ECs (t= 3.56, p= 0.003). In LCs, the tingling rating was significantly different only during evening cathodal vs evening sham (t= 2.17, p= 0.046). For the "burning" rating, a significant difference was found between evening anodal vs evening sham (t= 2.65, p= 0.018) in ECs, and a significant difference was found between evening cathodal vs evening sham (t= 2.36, p= 0.032) in LCs. The intensity of the reported side effects was in general low.

2.3.tDCS Blinding efficacy

To explore blinding efficacy, we asked participants to guess whether they received real tDCS (1 mA) or sham tDCS (0 mA) after each stimulation condition. Using the Chi-square Test for Associations, we explored whether participants could correctly discern each real stimulation condition (morning anodal, morning cathodal, evening anodal, evening cathodal) from its respective sham condition (morning sham, evening sham). The results of the respective Chi-square tests show no significant differences of participants' guesses between each real stimulation vs sham stimulation in both, ECs and LCs, and the whole group of participants (All) (Supplementary Table 6).

2.4.3D Modeling of the electrical current flow

Three-dimensional models of electrical current flow in the head induced by tDCS protocol (anodal M1, cathodal Fp2, 1 mA) applied to an adult head (New York (ICBM-NY) head[1]). The MR images was first segmented into 6 tissue types: gray matter (GM), white matter (WM), CSF, skull, scalp, and air cavities using the SPM8 software package (Welcome Trust Center for Neuroimaging, London, UK) with an improved tissue probability map. A custom MATLAB script (MATLAB R2016b, MathWork Inc., Natick, MA, USA) was then used to correct for segmentation errors made by SPM[2]. Then, a 3D model of the segmented images, with addition of the electrodes and saline-soaked sponges, was designed using the Simpleware software package version 5 (Synopsys, Mountain View, CA). Finally, the current flow distribution inside the head was

calculated based on the finite element method using COMSOL Multiphysics software package version 5.2 (COMSOL Inc., Burlington, MA). The conductivity values used for each tissue type were as follows (in S/m): GM: 0.276; WM: 0.126; CSF: 1.65; skull: 0.015; scalp: 0.465; air: 2.5 9 10-14; saline-soaked sponge: 1.5; electrode rubber: 29 [3, 4].

3. *Implicit motor learning*

To test if the learning sequence was preserved after the presentation of random stimuli in block 6, we analyzed RT differences of block 6 vs 7 too. Here, the respective ANOVA results show a significant block×chronotype×daytime interaction (F_1=10.76, p=0.003; ηp^2=0.27), but no interaction of block×chronotype (F_1=1.81, p=0.188), block×daytime (F_1=0.05, p=0.824), chronotype×daytime (F_1=0.08, p=0.771), or main effects of chronotype (F_1=0.11, p=0.733) and daytime (F_1=0.03, p=0.852). Bonferroni-corrected post *hoc* comparisons revealed a significantly shorter RT at block 7, indicative of sequence retention, at their circadian-preferred time for both chronotypes (Fig. 4.a, b, main text). At the non-preferred time, this difference was only significant for the LCs.

3.1. Absolute Reaction Time

We also analyzed SRTT task performance based on the absolute RT values. The results of the 2 (daytime) × 2 (chronotype) × 3 (block 5-7) ANOVA show significant interactions of block×chronotype×daytime ($F_{1.74}$=10.79, p<0.001; ηp^2=0.27), chronotype×daytime (F_1=8.21, p=0.007; ηp^2=0.22) and learning blocks (F_1=55.16, p<0.001; ηp^2=0.65), but no interaction of chronotype×block ($F_{1.51}$=1.53, p=0.224), block×daytime ($F_{1.74}$=0.43, p=0.621), or main effects of chronotype (F_1=1.60, p=0.215), and daytime (F_1=0.02, p=0.896). Post *hoc* comparisons of blocks revealed that both groups had significantly faster RT at blocks 5 and 7 and longer RT at block 6 at their circadian-preferred time (Supplementary Fig. 1).

Supplementary information

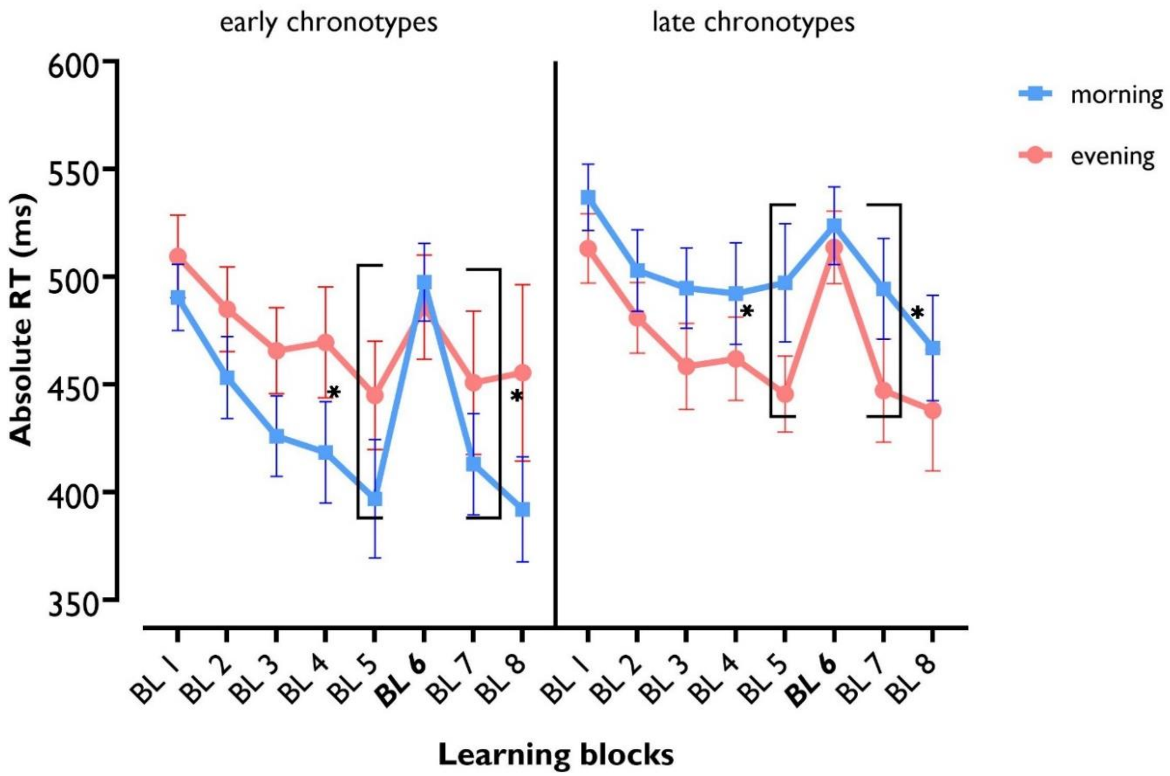

Supplementary Fig. 1. Chronotype affects motor learning performance dependent on the daytime. Absolute RTs were analyzed using a mixed-factorial (daytime×chronotype) design ANOVA. The RT difference between blocks 5 and 6 represents mostly exclusively sequence learning. In early chronotypes, the RT difference between these blocks was significant only in the morning ($t_{morning}$=3.05, p=0.002, $t_{evening}$=1.28, p=0.202). In late chronotypes, the respective RT difference was significant only in the evening ($t_{morning}$=0.82, p=0.405, $t_{evening}$=2.13, p=0.037). The RT difference between blocks 6, and 7 was significant in the morning for ECs ($t_{morning}$=2.56, p=0.010; $t_{evening}$=1.09, p=0.272) and in the evening for LCs ($t_{evening}$=2.08, p=0.038; $t_{morning}$=0.91, p=0.358). All pairwise comparisons are calculated using *Student's* t-test (paired, two-sided, p <0.05). n=31 (15 early and 16 late chronotypes). Data are presented as mean values±SEM. BL = block; RT = reaction time; ms = milliseconds. Asterisks [*] represent statistically significant differences between learning blocks RT (BL 6-5, BL 6-7]. In early chronotypes, the brackets refer to RT difference between blocks 6 vs 5 and 6 vs 7 in the morning. In late chronotypes, the brackets refer to RT difference between blocks 6 vs 5 and 6 vs 7 in the evening.

3.2.Error rate

We analyzed the number of errors in the learning blocks (block 5-7) to see if the error rate was affected by circadian preferred vs non-preferred times. The results of the 2 (daytime) × 2 (chronotype) × 3 (block 5-7) ANOVA show a significant interaction of chronotype×daytime (F_1=9.88, p=0.004; ηp^2=0.25) and main effect of block ($F_{1.71}$=4.57, p=0.019; ηp^2=0.13). *Post hoc* t-tests showed that both, ECs and LCs conducted more errors at block 6 compared to block 5 at their circadian non-preferred time (t_{ECs} = 2.36, p=0.032; t_{LCs}=2.54, p=0.023). *Post hoc* comparisons revealed no significant difference between error rates in block 6 vs 7 in both groups, although the number of committed errors in block 6 was numerically larger in both groups at their circadian

non-preferred time (Supplementary Fig. 2a). No significant interactions of block×chronotype×daytime ($F_{1.73}$=2.29, p=0.118), block×chronotype ($F_{1.71}$=1.77, p=0.184), block×daytime ($F_{1.73}$=0.85, p=0.416), or main effect of chronotype (F_{1}=1.76, p=0.194) and daytime (F_{1}=0.14, p=0.705) were found.

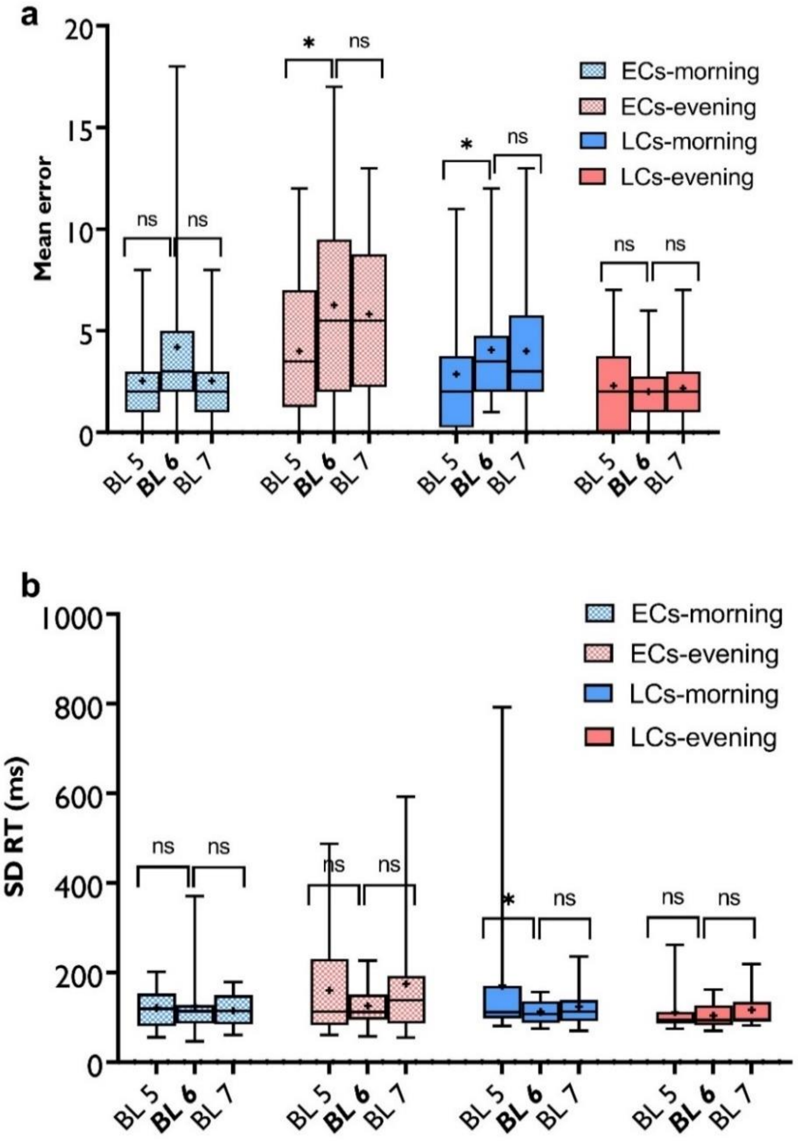

Supplementary Fig. 2. **Error rate and RT variability in the learning blocks for ECs and LCs**. Mean error and RT variability were analyzed using a mixed-factorial (daytime×chronotype) ANOVA. **a**, In both ECs and LCs, participants significantly committed more errors in block 6 vs block 5 only at the circadian non-preferred time ($ECs_{evening:}$ p=0.032; $LCs_{morning:}$ p=0.023). The error rate difference from block 6 to 7 was not significant in both groups. **b**, ECs and LCs displayed numerically higher RT variability in learning blocks at the circadian non-preferred time. The difference was, however, significant only for LCs in the morning (p=0.047). All pairwise comparisons are calculated by *post hoc* t-tests (paired, two-sided, p<0.05). n = 31 (15 ECs, 16 LCs). Data are presented as mean values±SEM. The horizontal bar shows the median, the + shows the mean, the upper and lower boundaries show the 25th and 75th percentiles, respectively and the whiskers show the 5-95 percentile. BL = block; ECs = early

chronotypes; LCs = late chronotypes; ns = nonsignificant; RT = reaction time; SDRT = reaction time variability; ms = millisecond. Asterisks [*] indicate a significant difference.

1.1.RT variability

In addition to the RT, which is the primary outcome variable of interest in this task, we also analyzed RT variability of learning blocks. The results of the 2 (daytime) × 2 (chronotype) × 3 (block 5-7) ANOVA show a significant interaction of chronotype×daytime (F_1=6.77, p=0.014; ηp^2=0.13), but no interaction of block×chronotype×daytime ($F_{1.59}$=2.56, p=0.099), block×chronotype ($F_{1.53}$=0.37, p=0.632), block×daytime ($F_{1.59}$=1.82, p=0.179), or main effects of block (F_1=1.55, p=0.223), chronotype (F_1=0.68, p=0.413), and daytime (F_1=0.22, p=0.641. *Post hoc* t-tests showed a significant increase of RT variability in block 5 vs 6 only for LCs in the morning (t= 2.00, p= 0.047) and a similar trend was observed for ECs in the evening as well. No significant difference between RT variability was found in block 7 vs block 6 (Supplementary Fig. 2b).

2. *EEG supplementary results*

2.1. SRTT

In addition to the Pz electrode, which is a major electrode of interest for the analysis of the P300 component, we analyzed other electrodes at temporal-parietal regions relevant for P300 activity, and identified a similar trend for chronotype-, and daytime-dependent alterations of the P300 amplitude at the P3 electrode. To test for statistical significance, we analyzed P-300 amplitudes (250-500 ms) of blocks (blocks 5-7), and amplitude differences at block 5 vs 6 (learning acquisition), and block 6 vs 7 (learning retention). The ANOVA results reveal a trendwise interaction of block×chronotype×daytime ($F_{1.96}$=2.64, p=0.080), and a significant main effect of block (F_{1846}=11.35, p<0.001), for P-300 at the P3 electrode, but no significant main effects of chronotypes (F_1=2.91, p=0.099) and daytime (F_1=1.05, p=0.312). *Post hoc* comparisons of P300 amplitudes over blocks showed a significantly larger P-300 amplitude in block 6 vs block 5 only in ECs at their circadian-preferred time (t=2.07, p=0.039) (BL5: mean±$SEM_{morning}$, 0.48±0.20µV; mean±$SEM_{evening}$, 0.95±0.26µV; BL6: mean±$SEM_{morning}$, 1.22±0.24µV; mean±$SEM_{evening}$, 1.02±0.23µV) (Supplementary Fig. 3a). Analysis of the P-300 amplitude differences ($P300_{block6}$-$P300_{block5}$) show a significant interaction of chronotype×daytime (F_1=4.70, p=0.038; ηp^2=0.14) in

the P3 electrode, and no significant main effects of chronotype (F_1=0.33, p=0.567) and daytime (F_1=1.85, p=0.184). The P300 amplitude difference between Block 5 and 6 was significant only in ECs in the morning compared to the respective difference in the evening (t=2.63, p=0.009) (mean±$SEM_{morning}$, 0.73±0.23μV; mean±$SEM_{evening}$, 0.06±0.19μV), but not in LCs (mean±$SEM_{morning}$, 0.20±0.09μV; mean±$SEM_{evening}$, 0.36±0.23μV) (Supplementary Fig. 3b). The ANOVA results for Block 6-7 amplitude differences show no significant interaction of chronotype × daytime or main effects of chronotype and daytime.

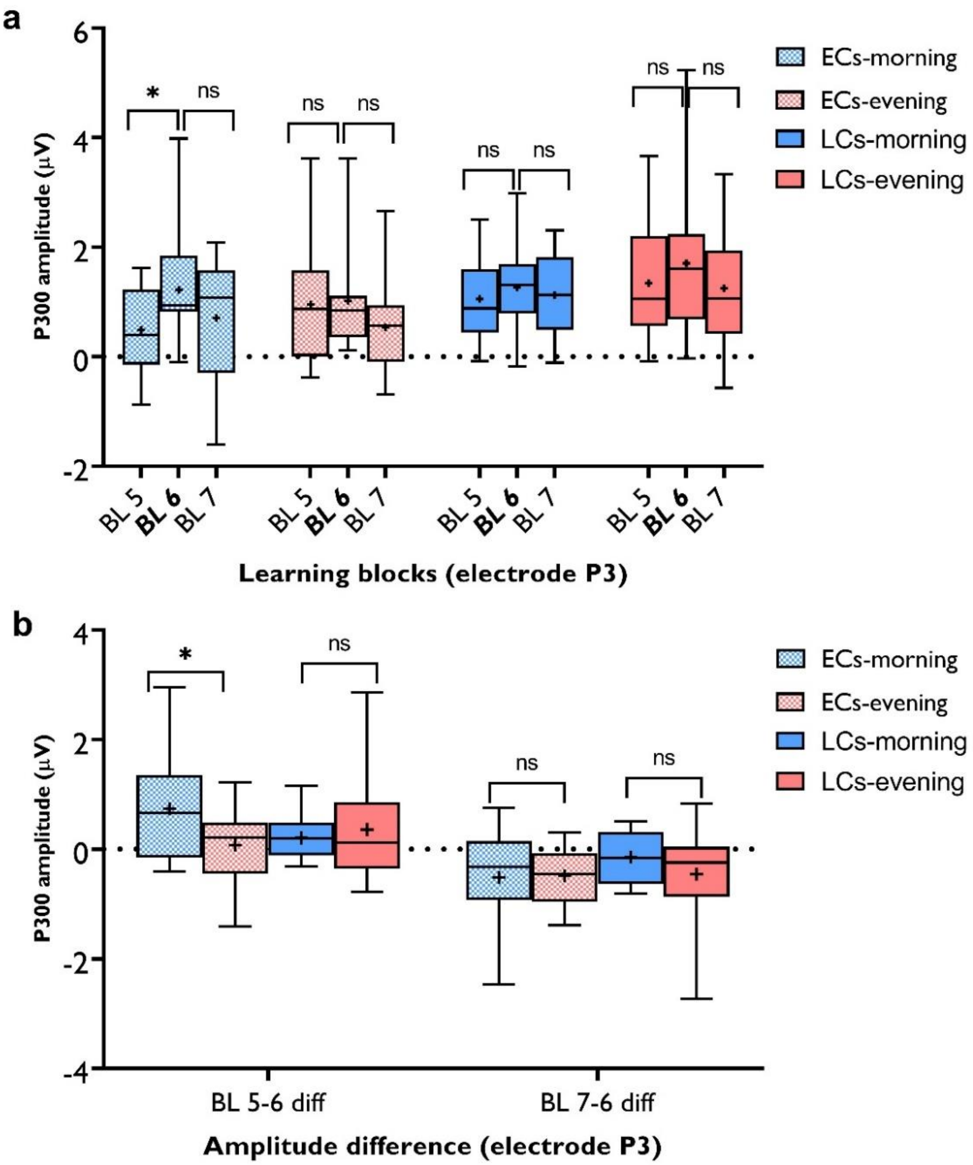

Supplementary Fig. 3: **P300 amplitudes of electrode P3 during motor sequence learning for ECs and LCs.** ERP amplitudes were analyzed using a mixed-factorial (daytime×chronotype) ANOVA. **a** P300 component was calculated per block in both groups. Pairwise comparisons show that only ECs displayed a significantly larger P300 at block 6 vs block 5 in the morning (p=0.039). **b** The P300 amplitude difference of learning blocks was calculated

for each group at circadian preferred vs non-preferred times. Only ECs displayed a significantly larger P300 amplitude difference in the morning compared to the respective value in the evening (p=0.009). All pairwise comparisons are calculated by *post hoc* t-tests (paired, two-sided, p<0.05). n = 31 (15 ECs, 16 LCs). Data are presented as mean values±SEM. The horizontal bar shows the median, the + shows the mean, the upper and lower boundaries show the 25th and 75th percentiles, respectively and the whiskers show the 5-95 percentile. BL = block; ECs = early chronotypes; LCs = late chronotypes; ns = nonsignificant. [*] indicates a significant difference.

2.2. Working memory and attention tasks

Beyond Pz, also the contribution of Cz to the P300 and N200 components was evaluated for the cognitive tasks, including working memory, Stroop test, and AX-CPT. For working memory, the results of the 2 × 2 ANOVA show a significant interaction of chronotype×daytime for the P300 component (F_1=11.19, p=0.002; ηp^2=0.27), but no significant main effects of chronotype (F_1=0.28, p=0.600) and daytime (F_1=2.05, p=0.162). *Post hoc* comparisons via *Student´s* t-tests show that working memory performance during the circadian-preferred time was related to a larger P300 amplitude under the Cz electrode in both groups (ECs: mean±$SEM_{morning}$, 3.66±0.51µV; mean±$SEM_{evening}$, 2.40±0.48µV; LCs: mean±$SEM_{morning}$, 2.35±0.71µV; mean±$SEM_{evening}$, 2.85±0.67µV), but this difference was significant only for ECs (t=3.59, p=0.003) (Supplementary Fig. 4a,b).

For the Stroop test, the results of the 2 × 2 ANOVA for the N200 amplitudes show a significant interaction of chronotype×daytime for overall trials (F_1=10.58, p=0.003; ηp^2=0.26), as well as congruent (F_1=10.12, p=0.003; ηp^2=0.26) and incongruent (F_1=6.28, p=0.018; ηp^2=0.18) trials. *Post hoc* t-tests showed that the N200 component of both, congruent and incongruent trials over the Cz electrode was larger only for ECs in the morning compared to the evening (t_{con}=3.77, p=0.002; t_{incon}=2.57, p=0.021) (Supplementary Fig. 4c,d). The main effects of daytime (F_1=2.38, p=0.133), and congruency (F_1=1.35, p=0.254) were not significant, but the main effect of chronotype was significant (F_1=6.28, p=0.032) for overall trials. When we compared the amplitude difference values from morning to evening in a one-way ANOVA, chronotype had a significant effect (incongruent: F_1=5.90, p=0.022; ηp^2=0.17; congruent: F_1=9.92, p=0.004; ηp^2=0.25) which indicates higher negativity of N200 at circadian-preferred times in both groups.

Supplementary information

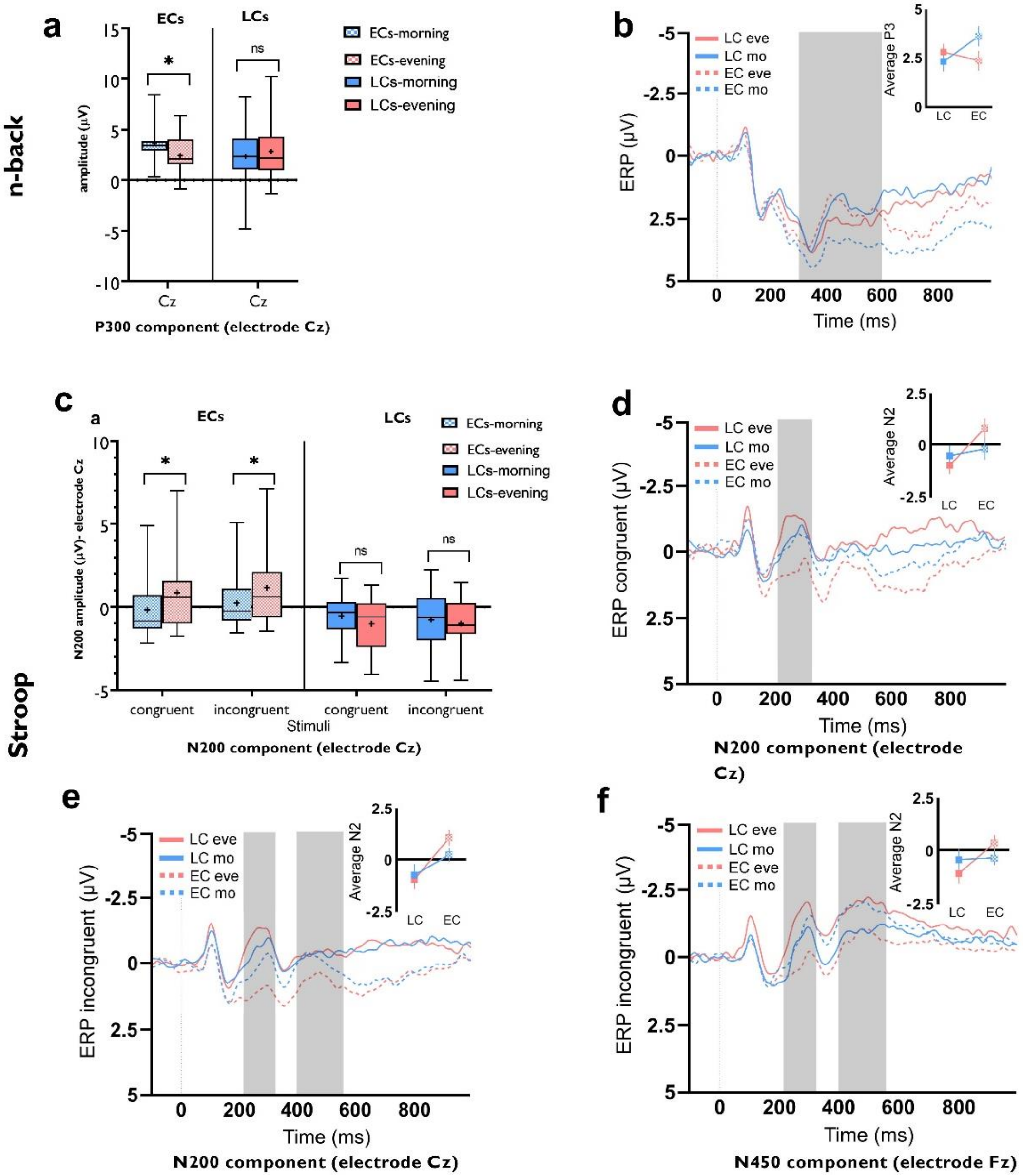

Supplementary Fig. 4: **ERP components of working memory and attention task performance at electrode Cz across time of day for ECs and LCs.** Behavioral and electrophysiological data were analyzed using a mixed-factorial (daytime×chronotype) ANOVA. **a, b** The P300 component was calculated for both groups at electrode Cz. ECs displayed a significantly larger P300 in the morning compared to the evening at electrode Cz (p=0.003). **c, d, e** The N200 component at electrode Cz was calculated for congruent (**d**) and incongruent (**e**) trials in each group at circadian preferred vs non-preferred times. Pairwise comparisons show that only ECs displayed a significantly larger N200 amplitude in the morning compared to the respective value in the evening for both congruent (p=0.002) and incongruent (p=0.021) trials. The amplitude difference of the N200 component (N200 morning – N200 evening) is, however, significant for both groups. **f** The N450 component was calculated for incongruent trials at electrode Fz in both groups. The N450 component is larger only for ECs in the morning. The amplitude difference of the N450 component (N450 morning – N450 evening) is however significant for both groups. All pairwise comparisons are calculated by *post hoc* t-tests (paired, two-sided, p<0.05). n=31 (15 ECs, 16 LCs). Data are presented as mean values±SEM. In **a** and **c**, the horizontal bar shows the median, the + shows the mean, the upper and lower boundaries show the 25th and 75th percentiles, respectively and the whiskers show the 5-95 percentile. BL = block; ECs = early chronotypes; LCs = late chronotypes; ns = nonsignificant; eve = evening; mo = morning; P3 = P300 component; N2 = N200; ms = millisecond. [*] indicates a significant difference.

Supplementary information

In addition to N200, we analyzed the N450 which is a prominent ERP marker related to Stroop conflict, especially for incongruent trials and is usually observed at frontocentral and centroparietal electrode positions. The results of the mixed-model ANOVA (2 × 2 × 2) showed a significant interaction of chronotype×daytime (F_1=5.78, p=0.023; ηp^2=0.16) and a significant main effect of congruency (F_1=7.23, p=0.012; ηp^2=0.20) for the Fz electrode only. The interactions of congruency×daytime, congruency×chronotype, and congruency×daytime×chronotype and the main effects of daytime and chronotype were not significant. Post *hoc* t-tests showed that the N450 component over the Fz electrode was larger for both, congruent and incongruent trials only for ECs in the morning as compared to the evening (t_{con}=2.77, p=0.014; t_{incon}=2.57, p=0.021) (Supplementary Fig. 4f). The results of the separate 2 × 2 ANOVAs conducted for the congruent and incongruent trials showed a significant interaction of chronotype×daytime (F_1=4.41, p=0.044; ηp^2=0.16) only for incongruent trials. However, when we compared the amplitude difference values between morning to evening in a one-way ANOVA, chronotype had a significant effect on both, incongruent (F_1=4.41, p=0.044; ηp^2=0.13), and congruent (F_1=4.80, p=0.037; ηp^2=0.14) trials, indicating higher negativity of the N450 at the circadian-preferred times of both groups. Analysis of the N200 amplitude of the electrode Cz showed no significant interaction of chronotype×daytime or main effects of chronotype and daytime on the N200 amplitude difference.

3. Correlational analyses

3.1. Correlation between sequence learning and plasticity induction

To explore the association between motor learning and plasticity, we calculated the correlation between the respective parameters. In LCs, anodal tDCS effects (MEP amplitude enhancement) were positively correlated with sequence learning (block 6 - 5 RT difference) in the evening (r=0.543, p=0.017), indicating that larger LTP-like plasticity effects were associated with enhanced sequence learning. Furthermore, cathodal tDCS-induced LTD-like plasticity (MEP amplitude reduction) in the morning for LCs was positively correlated with sequence learning in the morning (r=0.441, p=0.043), which means that reduced LTD-like plasticity was associated with poor sequence learning at the circadian non-preferred time. In ECs, we did not see a significant correlation between sequence learning and tDCS-induced plasticity in the morning and evening.

Supplementary information

3.2. Correlation between cortical excitability, working memory, and attention

The correlation between performance in the 3-back letter task, Stroop test and AX-CPT with the cortical excitability results was investigated. For ECs, RT of working memory performance in the evening was negatively correlated with cortical inhibition measured by I-wave facilitation ($r_{ISI\text{-}late}$= -0.453, p=0.039), indicating that performance was slower with increased cortical inhibition in the evening. Accuracy of working memory in the evening was positively correlated with cortical inhibition measured by I-wave facilitation protocol at early ISI ($r_{ISI\text{-}early}$= 0.500, p=0.024) which means that also accuracy decreased with increased inhibition (marked by larger MEP) in the evening. Also in ECs, accuracy of working memory in the morning was negatively correlated with cortical inhibition, as measured by I-wave facilitation at late ISI ($r_{ISI\text{-}late}$= -0.519, p=0.020), which means improved accuracy was associated with increased inhibition in the morning. In LCs, we found a significant positive correlation between working memory accuracy and cortical facilitation measured by ICF at ISI 10 ms in the evening (r_{ISI10}=0.561, p<0.012), which means that enhanced accuracy was associated with larger cortical facilitation.

In the Stroop test, which is a measure of selective attention, intracortical facilitation (ICF, ISI-15), was positively correlated with response accuracy in the evening for LCs (r=0.743, p<0.001), which means that accuracy was improved with increased cortical facilitation. Similarly, corticospinal excitability (measure by the I-O curve protocol at 150 % RMT intensity) and Stroop test RT were negatively correlated in the evening for LCs ($r_{overall\ trials}$= -0.464, p=0.035; $r_{incongruent\ trials}$= -0.473, p=0.032), indicating that enhanced selective attention was associated with increased cortico-spinal excitability at I-O curve intensities which are affected relevantly by glutamatergic activity. With regard to cortical inhibition, larger MEP obtained from the SAI protocol (ISI 40 ms) in the evening, which is indicative of less inhibition, and Stroop test RT were negatively correlated only for LCs in the evening ($r_{overall\ trials}$= -0.487, p=0.028; $r_{incongruent\ trials}$= -0.465, p=0.035; $r_{congruent\ trials}$= -0.507, p=0.023). This indicates that selective attention improved with reduced inhibition. In sum, better task performance in the evening for LCs was significantly associated with higher cortical facilitation and lower cortical inhibition. We found no correlation between cortico-cortical/corticospinal excitability and selective attention in ECs.

For AX-CPT task performance, accuracy was negatively correlated with cortical inhibition measured by the SAI protocol at ISI 20 ms (r= -0.452, p=0.039) in the morning for ECs, which

indicates that improved accuracy was associated with increased cortical inhibition at the circadian-preferred time in this chronotype. In LCs, accuracy in the evening was positively ($r_{accuracy}$= 0.521, p=0.019) correlated with cortico-spinal facilitation (I-O curve) at 110% RMT intensity, whereas RT was negatively (r_{RT}= -0.467, p=0.031) correlated with the same protocol at 150% of RMT intensity. This indicates that improved task performance (i.e., higher accuracy and faster RT) were associated with increased cortico-spinal facilitation. Taken together, improved sustained attention at the circadian-preferred time, measured by higher accuracy of or shorter RT in AX-CPT performance, was associated with higher cortical inhibition in ECs and higher cortico-spinal facilitation in LCs.

3.3. Correlation between chronotype score, physiological parameters, and behavior

We also explored potential correlations between the participants' scores on DMEQ (i.e., circadian preference) and major outcome measures. No significant correlations were observed between DMEQ scores and cortical excitability except for intracortical inhibition (measured by SICI) of ECs obtained in the morning, which was positively correlated with DMEQ scores (r= 0.658, p=0.006). No significant correlations were neither found between DMEQ scores and neuroplasticity, or major outcome measures of the behavioral tasks.

3.4. Correlation between cognitive tasks

We also explored correlations of performance between the cognitive tasks. No significant correlations were observed between working memory and motor learning at the group level and in ECs alone. In LCs, however, a significant correlation was found between working memory RT in the evening and learning block of SRTT in the evening (r_{BL5}= -0.513, p=0.042; r_{BL6}= -0.614, p=0.011) indicating faster RT in both tasks in the evening were associated. For other tasks, no significant correlations were found. Overall, these data show no robust correlations of performance between the different tasks.

Supplementary information

Supplementary Tables

Supplementary Table 1: Baseline measurements of the cortical excitability protocols

**Protocol**	**measurement**	**daytime**	**Group**	
			**ECs**	**LCs**
Single-pulse MEP	SI 1_{mV} (%)	morning	51.31 ± 9.82	50.43 ± 8.46
		evening	50.50 ± 10.19	50.87 ± 7.91
RMT	%MSO	morning	42.87 ± 7.54	41.81 ± 6.88
		evening	41.68 ± 7.15	42.12 ± 7.09
AMT	%MSO	morning	35.18 ± 6.95	34.40 ± 8.23
		evening	33.53 ± 6.97	34.96 ± 8.18
I-O curve	MEP amplitude at RMT	morning	0.235 ± 0.213	0.197 ± 0.204
		evening	0.149 ± 0.091	0.200 ± 0.133
SICI-ICF	Single-pulse MEP	morning	1.073 ± 0.335	0.941 ± 0.152
		evening	0.910 ± 0.342	0.980 ± 0.121
I-wave facilitation	Single-pulse MEP	morning	1.020 ± 0.341	1.041± 0.119
		evening	0.945 ± 0.318	1.04 ± 0.261
SAI	Single-pulse MEP	morning	1.237 ± 0.512	1.178 ± 0.438
		evening	1.194 ± 0.269	1.195 ± 0.175

Note: MEP = motor-evoked potentials; ECs = early chronotypes; LCs = late chronotypes; SI_{1mv} (%) = maximum stimulator output (%MSO) required for the SI1mV MEP amplitude; I-O curve = input-output curve; SICI-ICF = short latency intracortical inhibition and facilitation; SAI = short-latency afferent inhibition; RMT = resting motor threshold; AMT = Active motor threshold.

Supplementary Table 2: Results of 2 × 2 ANOVAs for control MEPs (RMT intensity MEP for I-O curve, single-pulse MEP for other protocols), SI1mV, and %MSO for RMT/AMT.

**Protocol**	**Measurement**	**Factor**	***df***	***F***	***p***
Single-pulse MEP	SI_{1mV}	daytime	1	0.12	0.724
		chronotype	1	1.40	0.244
		daytime×chronotype	1	0.001	0.938
RMT	%MSO	daytime	1	0.86	0.359
		chronotype	1	0.01	0.901
		daytime×chronotype	1	2.54	0.120
AMT	%MSO	daytime	1	3.23	0.082
		chronotype	1	0.015	0.903
		daytime×chronotype	1	13.30	<**0.001**
I-O curve	MEP amplitude at RMT	daytime	1	1.04	0.315
		chronotype	1	0.02	0.882
		daytime×chronotype	1	1.169	0.288
SICI-ICF	Single-pulse MEP	daytime	1	0.98	0.329
		chronotype	1	0.22	0.641
		daytime×chronotype	1	2.65	0.113

Supplementary information

I-wave facilitation	Single-pulse MEP	daytime	1	0.23	0.631
		chronotype	1	0.83	0.367
		daytime×chronotype	1	0.37	0.547
SAI	Single-pulse MEP	daytime	1	0.017	0.895
		chronotype	1	0.112	0.739
		daytime×chronotype	1	0.090	0.765

Mixed-factorial ANOVAs (daytime×chronotype) were performed with time of day (morning vs evening) as within-subject factors and chronotype (ECs vs LCs) as the between-subject factor. In case of significant results of the ANOVA, post hoc comparisons were performed using post hoc t-tests (p<0.05, two-sided). *Note*: MEP = motor-evoked potentials; chronotype = early (ECs) and late (LCs) chronotypes; SI_{1mv} (%) = maximum stimulator output (%MSO) required for the SI1mV MEP amplitude; I-O curve = input-output curve; SICI-ICF = short latency intracortical inhibition and facilitation; SAI = short-latency afferent inhibition; RMT = resting motor threshold; AMT = active motor threshold.

Supplementary Table 3: Baseline measurements of the neuroplasticity sessions

**tDCS**	**daytime**	**baseline MEP**		**SI_{1mV} (%)**	
		**ECs**	**LCs**	**ECs**	**LCs**
Anodal stimulation	morning	0.981 ± 0.084	0.957 ± 0.060	50.500 ± 9.919	50.406 ± 9.593
	evening	0.980 ± 0.068	0.977 ± 0.070	49.562 ±10.282	49.875 ± 7.219
Cathodal stimulation	morning	0.976 ± 0.072	0.983 ± 0.066	49.906 ± 10.188	49.718 ± 8.606
	evening	1.001 ± 0.058	1.006 ± 0.067	50.281 ± 10.494	49.312 ± 9.183
Sham stimulation	morning	0.981 ± 0.058	0.998 ± 0.054	49.562 ± 9.069	51.876 ± 10.057
	evening	0.972 ± 0.076	1.029 ± 0.054	50.062 ± 9.453	50.312 ± 7.400

Note: tDCS = transcranial direct current stimulation; MEP = motor-evoked potentials; ECs = early chronotypes; LCs = late chronotypes; SI_{1mv} (%) = maximum stimulator output (%MSO) required for the SI1mV MEP amplitude.

Supplementary Table 4: Reported side effects of tDCS during stimulation.

**Side effects**	**Group**	**Anodal-morning**	**Cathodal-morning**	**Sham-morning**	**Anodal-evening**	**Cathodal-evening**	**Sham-evening**
Visual phenomenon	ECs	0.062±0.25	0.06±0.25	0.00	0.06±0.25	0.00	0.00
	LCs	0.062±0.25	0.12±0.34	0.00	0.12±0.34	0.00	0.00
Itching	ECs	1.31±1.07	1.25±1.23	0.81±0.65	1.06±0.68	0.93±0.99	0.81±0.83
	LCs	1.25±1.18	0.56±0.81	0.68±0.87	1.31±1.01	0.68±0.79	0.68±0.70
Tingling	ECs	1.56±0.96	1.31±1.13	0.75±0.68	1.56±0.81	1.31±1.13	0.75±0.68
	LCs	1.68±1.19	1.37±0.71	1.06±0.99	1.12±0.88	1.43±0.96	0.81±0.54
Burning	ECs	1.12±1.40	1.06±1.12	0.68±0.94	1.06±1.12	0.62±0.95	0.25±0.44
	LCs	0.93±0.99	0.75±0.93	0.43±0.72	0.75±0.93	1.31±1.40	0.50±0.96
Pain	ECs	0.43±0.72	0.25±0.57	0.12±0.50	0.12±0.34	0.12±0.34	0.06±0.25
	LCs	0.12±0.34	0.18±0.54	0.00	0.25±0.68	0.06±0.25	0.96±0.54

Note: The presence and intensity of tDCS side-effects were rated on a numerical scale ranging from zero to five, zero representing no and five extremely strong sensations. Data are presented as mean ± SD. ECs = early chronotypes; LCs = late chronotypes.

Supplementary information

Supplementary Table 5: Repeated-measures ANOVA results for the presence and intensity of reported tDCS side-effects

Side effects	*Source*	*df*	*F*	*p*	ηp^2
Visual phenomenon	tDCS	2.68	1.89	0.142	0.059
	chronotype	1	0.37	0.542	0.012
	tDCS×chronotype	2.68	0.22	0.860	0.007
Itching	tDCS	3.83	2.30	0.064	0.071
	chronotype	1	0.93	0.341	0.030
	tDCS×chronotype	3.83	1.01	0.404	0.032
Tingling	tDCS	4.55	4.39	**0.001**	0.127
	chronotype	1	0.06	0.803	0.002
	tDCS×chronotype	4.55	0.68	0.621	0.022
Burning	tDCS	3.25	2.64	**0.048**	0.081
	chronotype		0.01	0.923	0.001
	tDCS×chronotype	3.25	1.60	0.190	0.050
Pain	tDCS	3.75	0.97	0.423	0.031
	chronotype	1	0.64	0.427	0.021
	tDCS×chronotype	3.75	0.97	0.423	0.031

Note: The presence and intensity of reported side-effects during tDCS were analyzed by repeated-measures mixed-model ANOVAs with tDCS (6 values) as the within-subject and chronotype (ECs, LCs) as the between-subject factors. Significant effects of tingling and burning, but no significant effects of other side-effects, were revealed. Significant effects are marked in **bold** (where $P < 0.05$). Pairwise comparisons are calculated using *Student's* t-test (two-sided). $n = 32$ (16 per group).

Supplementary Table 6: Chi-square Test for Association results for exploring guessing among participants.

Stimulation condition	Group	*df*	χ^2	*p*
anodal morning vs sham morning	ECs	1	0.837	0.350
	LCs	1	0.00	1.000
	All	1	0.269	0.604
cathodal morning vs sham morning	ECs	1	2.798	0.094
	LCs	1	1.333	0.248
	All	1	3.521	0.061
anodal evening vs sham evening	ECs	1	1.340	0.247
	LCs	1	2.286	0.131
	All	1	0.083	0.773
cathodal evening vs sham evening	ECs	1	0.027	0.869
	LCs	1	0.291	0.590
	All	1	0.169	0.681

Note: ECs = early chronotypes; LCs = late chronotypes. $p<0.05$ (two-sided).

Supplementary information

Supplementary References

1. Huang, Y., Parra, L.C. & Haufe, S. The New York Head-A precise standardized volume conductor model for EEG source localization and tES targeting. *Neuroimage* **140**, 150-162 (2016).
2. Huang, Y., *et al.* Automated MRI segmentation for individualized modeling of current flow in the human head. *J Neural Eng* **10**, 066004 (2013).
3. Datta, A., *et al.* Gyri-precise head model of transcranial direct current stimulation: Improved spatial focality using a ring electrode versus conventional rectangular pad. *Brain Stimulation* **2**, 201-207.e201 (2009).
4. Wagner, T., *et al.* Transcranial direct current stimulation: A computer-based human model study. *NeuroImage* **35**, 1113-1124 (2007).
