# Supplementary material for: Cognitive functions and underlying parameters of human brain physiology are associated with chronotype
Source: Nat Commun. 2021 Aug 3;12:4672. doi: 10.1038/s41467-021-24885-0 (PMC8333420; doi:10.1038/s41467-021-24885-0)
Supplement: Supplementary file 3 — Reporting summary [file 41467_2021_24885_MOESM3_ESM.pdf]

nature research

Corresponding author(s): Michael A. Nitsche

Last updated by author(s): Mar 23, 2020

Reporting Summary

Nature Research wishes to improve the reproducibility of the work that we publish. This form provides structure for consistency and transparency in reporting. For further information on Nature Research policies, see Authors & Referees and the Editorial Policy Checklist .

Statistics

For all statistical analyses, confirm that the following items are present in the figure legend, table legend, main text, or Methods section.

n/a	Confirmed	
☐	☒	The exact sample size (*n*) for each experimental group/condition, given as a discrete number and unit of measurement
☐	☒	A statement on whether measurements were taken from distinct samples or whether the same sample was measured repeatedly
☐	☒	The statistical test(s) used AND whether they are one- or two-sided<br>*Only common tests should be described solely by name; describe more complex techniques in the Methods section.*
☒	☐	A description of all covariates tested
☐	☒	A description of any assumptions or corrections, such as tests of normality and adjustment for multiple comparisons
☐	☒	A full description of the statistical parameters including central tendency (e.g. means) or other basic estimates (e.g. regression coefficient) AND variation (e.g. standard deviation) or associated estimates of uncertainty (e.g. confidence intervals)
☐	☒	For null hypothesis testing, the test statistic (e.g. *F*, *t*, *r*) with confidence intervals, effect sizes, degrees of freedom and *P* value noted<br>*Give P values as exact values whenever suitable.*
☒	☐	For Bayesian analysis, information on the choice of priors and Markov chain Monte Carlo settings
☒	☐	For hierarchical and complex designs, identification of the appropriate level for tests and full reporting of outcomes
☐	☒	Estimates of effect sizes (e.g. Cohen's *d*, Pearson's *r*), indicating how they were calculated

Our web collection on statistics for biologists contains articles on many of the points above.

Software and code

Policy information about availability of computer code

Data collection	Behavioral tasks were presented by the E-prime software (E-Prime 2.0).<br>EEG data was recorded using the NeurOne Tesla EEG amplifier (NeurOne; Bittium Biosignals Ltd., Kuopio, Finland).<br>Transcranial Magnetic and electrical Stimulation (TMS, tDCS) were delivered with a PowerMag ppTMS magnetic stimulator magnetic stimulator (Mag & More, Munich, Germany) and a battery-driven constant current stimulator (neuroConn GmbH, Ilmenau, Germany). Motor evoked potentials (MEPs) were amplified and digitized using D440-2 (Digitimer, Welwyn Garden City, UK) and a micro 1401 AD converter (Cambridge Electronic Design, Cambridge, UK) and collected by Signal Software (Cambridge Electronic Design, v. 2.13). Resting Motor Threshold (RMT) was examined using the TMS Motor Threshold Assessment Tool (MTAT 2.0).<br>Modeling of current flow distribution in the head was calculated using COMSOL Multiphysics software v. 5.2 (COMSOL Inc., MA, USA).
Data analysis	IBM SPSS 26 (IBM, SPSS, Inc., Chicago, IL), GraphPad Prism 8.2.1 (GraphPad Software, San Diego, California), Signal Software (Cambridge Electronic Design, v. 2.13), BrainVision Analyzer 2.1 (Brain Products GmbH, München, Germany), Microsoft Excel (Microsoft Corporation),SPM8 software package (Welcome Trust Center for Neuroimaging, London, UK), Simpleware software package version 5 (Synopsys, Mountain View, CA), COMSOL Multiphysics software version 5.2 (COMSOL Inc., MA, USA), MATLAB R2016b (MathWork Inc., Natick, MA, USA).

For manuscripts utilizing custom algorithms or software that are central to the research but not yet described in published literature, software must be made available to editors/reviewers. We strongly encourage code deposition in a community repository (e.g. GitHub). See the Nature Research guidelines for submitting code & software for further information.

Data

Policy information about availability of data

All manuscripts must include a data availability statement. This statement should provide the following information, where applicable:

- Accession codes, unique identifiers, or web links for publicly available datasets
- A list of figures that have associated raw data
- A description of any restrictions on data availability

The datasets generated and/or analysed during the current study are not publicly available due to institutional regulations, ethics, and confidentiality agreements, but are available from the corresponding author upon reasonable request. Source data are provided with this paper.

Field-specific reporting

Please select the one below that is the best fit for your research. If you are not sure, read the appropriate sections before making your selection.

☐ Life sciences ☒ Behavioural & social sciences ☐ Ecological, evolutionary & environmental sciences

For a reference copy of the document with all sections, see nature.com/documents/nr-reporting-summary-flat.pdf

Behavioural & social sciences study design

All studies must disclose on these points even when the disclosure is negative.

Study description

This is a quantitative experimental study. The study design is randomized, cross-over single-blinded. We conducted mixed-model repeated measures ANOVAs with chronotype as the between-subject factor and daytime, session and variable-specific values (e.g., learning blocks in SRTT; ISI in TMS protocols) as the within-subject factors.

Research sample

Participants were students from the Technical University of Dortmund, Ruhr-University Bochum and other young participants from the Ruhr area in Germany. Participants were recruited via posting announcements on the "Research Participation Pool" of the local institute via the social media, as well ass local and electronic bulletin boards. They first completed the DMEQ online/on paper and were initially selected in case they met early and late chronotype qualifications based on their answers. Two-hundred and sixty-nine volunteers who completed the DMEQ during the course of the experiment, sixty-nine were evening-types (6 definite evening-types) and thirty-five were morning-types (5 definite morning-types). Thirty-two volunteers (16 females, mean age=26.43, SD=4.95) who were qualified as early or late chronotype and met the inclusion criteria (healthy, right-handed, non-smoker, no metal implants in head, no implanted electronic devices, no history of neurological problems or head injury) were included in the early-chronotype (8 females, N=16) and late-chronotype (8 females, N=16) groups. The sample is homogenous in gender (equal number of males and females) and age (young participant) and is representative of the target population of this age range. The main rationale of the study was to see how human cognition and underlying brain physiology are affected by circadian preference in two groups of early and late chronotypes.

Sampling strategy

Sample size was calculated a-priori based on power analyses which showed that for a medium effect size (partial eta squared=0.10) (suggested for NIBS studies), a minimum of 24 subjects is required to achieve 95% power at an alpha of 0.05 for the primary statistical test with a mixed model repeated-measures mixed-ANOVA design. We increased the sample size to 32 to fully counterbalance tasks order in each group (N=16) and compensate for unforeseen variability and dropouts.

Data collection

Cortical excitability sessions (two sessions with TMS protocols) took place once in the morning and once in the evening at the same fixed time. Measurements were scheduled to start at 8:30 am for the morning session and 7:00 pm for the evening session after the preparation stage (motor cortex hotspot identification, RMT, and AMT determination procedures). There was a one-week interval between each session. Participants were instructed not to consume caffeine, alcohol, or engage in strenuous physical activities 24 h prior to each session to ensure a stable level of motor-cortical excitability. All TMS protocols were conducted with a PowerMag lab 30 magnetic stimulator (Mag & More, Munich, Germany) through a figure-of-eight magnetic coil (diameter of one winding, 70 mm; peak magnetic field, 2T). Signals were amplified, and filtered (1000; 3Hz - 3 KHz) using D440-2 (Digitimer, Welwyn Garden City, UK) and were digitized (sampling rate, 5 kHz) with a micro 1401 AD converter (Cambridge Electronic Design, Cambridge, UK), controlled by Signal Software (Cambridge Electronic Design, v. 2.13). The RMT was examined using the TMS Motor Threshold Assessment Tool (MTAT 2.0, http://www.clinicalresearcher.org/software.htm).

Neuroplasticity induction sessions (6 sessions including (morning anodal, morning cathodal, morning sham, and evening anodal, evening cathodal, evening sham) in randomized order) started at a fixed time in the morning and evening and there was a one-week interval between sessions. Morning sessions started at 8:00 am and evening sessions at 6:30 pm; starting time of tDCS was scheduled to take place around 8:30 in the morning session and 7:00 in the evening session, following the preparation stage which took roughly 20-30 min. Single-pulse MEPs were obtained in the same manner as described in the previous section, and TMS intensity was set to evoke MEPs of approximately 1-mV peak-to-peak amplitude. In all of the cortical excitability and neuroplasticity induction sessions, only the experimenter and the participant were in the testing room. Each stimulation session took about 1 hour long.

For the behavioral/cognitive sessions (two sessions), participants performed the tasks in two randomly-assigned sessions in the morning and evening at the same time the previous sessions took place with at least one-week interval. The order of tasks was counterbalanced across participants, with the exception of the SRTT, which was always conducted first and was scheduled to begin around the time cortical excitability was monitored and tDCS were applied. All tasks (SRTT, N-back, Stroop and AX-CPT) were presented on a computer screen (15.6"in. Samsung) via E-prime software (v.2.0) at the viewing distance from the monitor was approximately 50 cm. The tasks were conducted in a soundproof electro-magnetic shielded room during EEG recording. The experimenter was not present in the testing room and monitored the participant remotely on a PC screen. EEG was recorded continuously during cognitive task performance from 30 scalp electrodes positioned according to the international 10–20 system using the NeurOne Tesla EEG amplifier (Bittium, NeurOne, Bittium Corporation, Finland) with a sampling rate of 1000 Hz. The electrodes were connected to the head using high-viscosity electrolyte

	gel (SuperVisc, Easycap, Herrsching, Germany). Raw EEG data were recorded and stored for offline analysis using BrainVision Analyzer 2.1 (Brain Products GmbH, München, Germany). Each cognitive session took around 1.5 hours with cap preparation time (around 30 min). The experimenter was not blind to study hypotheses and stimulation conditions. In all of 10 experimental sessions, no one expect the experimenter and the participant were in the testing room.
Timing	Data collection started from November 2017- and took about 1.5 year and finished on March 2019. In summers , when the days are long in the state of North Rhine-Westphalia (Germany) and could affect dark/light cycle especially in the evening, no data collection was conducted from May to August 2018. This was to prevent potential effects of too different dark/light cycles in the evening and morning measurement. In other months, the experiment was scheduled to be randomly distributed across other seasons in both groups.
Data exclusions	One participant could identify the stimuli sequence in the SRTT and the respective behavioral and EEG data were excluded from the analysis. The exclusion criteria for the SRTT were pre-established according to previous studies in which being able to identify the stimuli sequence is an exclusion criterion in this implicit motor learning task. Moreover, resting-EEG data of one participant from each group (n=2) were excluded from the final analysis due to noise
Non-participation	No participants dropped out/declined participation.
Randomization	This study had a randomized, cross-over, between- and within-subject group design. Group (chronotype) allocation could not be randomized because this is based on trait characteristics. All of the experimental sessions (2 cortical excitability sessions, 6 neuroplasticity session, 2 behavioral/EEG sessions) were applied in a randomized-order. In each group, neuroplasticty sessions assignment (6 sessions) was randomly done for each participant using a randomizer website (https://www.randomizer.org/) . Order of excitability sessions (two) and behavioral/cognitive sessions (two) were determined using a counter-balanced order. The order of behavioral tasks was also in a counterbalanced order.

Reporting for specific materials, systems and methods

We require information from authors about some types of materials, experimental systems and methods used in many studies. Here, indicate whether each material, system or method listed is relevant to your study. If you are not sure if a list item applies to your research, read the appropriate section before selecting a response.

Materials & experimental systems

n/a	Involved in the study
☒	☐ Antibodies
☒	☐ Eukaryotic cell lines
☒	☐ Palaeontology
☒	☐ Animals and other organisms
☐	☒ Human research participants
☒	☐ Clinical data

Methods

n/a	Involved in the study
☒	☐ ChIP-seq
☒	☐ Flow cytometry
☒	☐ MRI-based neuroimaging

Human research participants

Policy information about studies involving human research participants

Population characteristics	see above.
Recruitment	Recruitment flyers were posted on the social media, the host Institute "Participation Pool System", and local and electronic bulletin boards. Participants first completed the DMEQ questionnaire online/on paper and were initially selected in case they were qualified as early and late chronotype. Of two-hundred and sixty-nine volunteers who completed the DMEQ during the course of the experiment, sixty-nine were evening-types (6 definite evening-types) and thirty-five were morning-types (5 definite morning-types). Thirty-two volunteers (16 females, mean age=26.43, SD=4.95) who were qualified as early or late chronotype and met the inclusion criteria (healthy, right-handed, non-smoker, no metal implants in head, no implanted electronic devices, no history of neurological problems or head injury) were included in the early-chronotype (8 females, N=16) and late-chronotype (8 females, N=16) groups. They then took part in a test TMS session to become acquainted with experiencing stimulation and were informed about the experimental course. In case they agreed to participate, and signed the informed consent form, they were recruited for the experiment. At any point in the study, participants were free to withdraw from the study. We did not include participants with neutral chronotype for the experiment, thus the results cannot be interpreted with respect to neutral chronotypes. Furthermore, volunteers with no access to media in which the study was announced, as well as volunteers with reservations about neurophysiological experiments, and people with regular employment (for those it might have been difficult to follow the numerous sessions at potentially problematic time points, which were taking some hours each), might not have applied for participation, thus the study results are not representative at the level of the whole population
Ethics oversight	This study conformed to the Declaration of Helsinki guidelines and was approved by the Institutional Review Board of the Leibniz Research Centre for Working Environment and Human Factors.

Note that full information on the approval of the study protocol must also be provided in the manuscript.
