# Supplementary material for: Cognitive functions and underlying parameters of human brain physiology are associated with chronotype
Source: Nat Commun. 2021 Aug 3;12:4672. doi: 10.1038/s41467-021-24885-0 (PMC8333420; doi:10.1038/s41467-021-24885-0)
Supplement: Supplementary file 2 — Peer Review File [file 41467_2021_24885_MOESM2_ESM.pdf]

Reviewers' comments:

Reviewer #1 (Remarks to the Author):

This study was performed to examine the effects of chronotype on motor cortical physiology, plasticity and behaviour. The authors demonstrated an interaction between chronotype and time of day for motor cortical excitability and paired-pulse TMS measures of excitation and inhibition, which taken together suggest that subjects had decreased excitability / increased inhibition at their non-preferred time of day. The time of day also had a significant effect on plasticity induction with tDCS, with the largest effects being seen at the preferred time of day. Finally, chronotype had significant effects on both motor learning and working memory tasks.

This is an impressive study involving a substantial degree of data collection to answer an interesting question in several different ways. It adds substantially to the literature by extending our understanding of the neurophysiological underpinnings of chronotypic differences. I have a few comments and suggestions for the authors, which I hope will aid the manuscript.

Major Points

1. I take the authors point, but I am not sure that the link between stimulation-induced neuroplasticity and motor learning is as well as established as they suggest (line 110). I think that this is a stretch and should be rephrased.
2. Given the variability inherent in tDCS protocols, how was the study powered? I think more details would be helpful here.
3. Given the importance of circadian rhythms, did the authors ensure that they kept records of sleep/wake cycles, caffeine intake, (nicotine intake?) other things that might affect a person’s circadian rhythm? Was sleepiness or equivalent taken into account?
4. Given the previously reported effects of several factors, including a number of genetic variants (e.g BDNF phenotype) the results presented in Figure 3 look remarkably homogenous. Could the authors comment on why this might be, compared with similar data (answering different questions) from other labs?
5. There is some evidence that chronotype affects other brain metrics that might then affect those demonstrated here, for example, DTI and grey matter volumes (e.g. Rosenberg et al., Neuroimage 2014; Rosenberg et al., BS&F 2018). How would more/less grey matter interact with the effects of tDCS, for example? The current model shown in figure 3 is for a standard brain, but the current density (and hence, probably, the effectiveness) must depend on the individual anatomy, which may be affected by chronotype. I understand that the authors will not have the data to answer this directly, but a discussion would be useful.
6. The authors have numerical data on the DMEQ which presumably could be used to quantify the degree of “evening preference” or morning preference. Are there correlations between the major findings (particularly the physiology and the behaviour) and this DMEQ score? While I understand the study was not powered to examine this, I think it might be of interest, and would strengthen the arguments if such a relationship was present.

Minor points.

1. The title probably needs rephrasing – cognitive function and their physiological parameters are chronotype-dependent? The grammar doesn’t quite work at the moment, though I have no issue with the content.
2. A minor point, but daytime would probably better be “time of day”.
3. While taking the IO slope as the dependent variable would reduce effects of differences in rMT – were there differences between the sessions in the rMT – both in terms of the amplitude of the MEPs evoked, and more interestingly, the % of stimulator output used to evoke them?
4. I appreciate this group's transparency with their statistics, and the ANOVA tables are helpful with such complex approaches. Please could these be added somewhere for the other results too?

Supplementary tables would be fine, but it is helpful to see them all for clarity.
5. I am not sure I understand the statement "Female subjects were not examined during the menstrual period to ensure hormonal changes would not interfere with their chronotype. Hormonal changes occur throughout the menstrual cycle and not just during bleeding. Please could the authors clarify?
6. Lines 296-8 I understand that there is not space to put all the results in the main text, but it would be useful to briefly summarise the key points here.
7. Line 365 – should read chronotypes
8. I like the idea of figure 6, but I am not sure that it adds anything to the study. Could it be a graphical abstract? Or maybe there could be some mechanistic hypotheses added? At the moment it seems somewhat redundant.
9. Line 497 – should read "one week" I imagine.

Reviewer #2 (Remarks to the Author):

I really enjoyed reading this manuscript. It is very well written, thoughtful with a measured narrative based on a set of studies that were clearly a great deal of work examining multiple levels of the biological hierarchy from synaptic function to behaviour. The work is nicely illustrated and convincing. I have some comments that may strengthen the work further.

Motor cortical excitability changed with the preferred time of day. As a consequence, are the differences in, for example, the double TMS experiments simply because a different stimulation intensity is being used and so what is being reported is simply a different position within the double pulse curve? For example, and admittedly an extreme example for illustrative purposes – at the preferred time of day 30% of machine output might be applied but at the non-preferred time of day 80% of machine output is used (due to changes in excitability). Then a change in the curve is really because there is a change in stimulation intensity (due to excitability changes) as opposed to some additional change that is specifically revealed in the double pulse experiments. Is it potentially possible that if the machine output had stayed the same – the excitability would show a change (MEP magnitude with chronotype and time of day) – however the double pulse curve would not differ significantly? Thus, the differences in the double pulse curve are driven by changes in excitability (i.e., non-independent measure). I think this is an important issue, which regardless of the concern being misplaced (or not) needs discussed to help other readers.

It would be helpful to know time of sleep onset, time of waking, sleep duration, and the duration from waking to the start of the experiment for each participant (if possible). For example, from information through sleep-logs. I am suggesting this for two reasons: (a) the relationship between sleep and plasticity is topical (see below) and (b) to provide further information about the circadian cycle of the different groups (i.e., the phase lag between the groups).

Sleep and plasticity have been linked. For example, sleep deprivation impairs the same brain stimulation markers of plasticity used in the current work. Plasticity might then reasonably be expected to be higher in the hours following sleep than after substantial time awake. At first sight this would appear inconsistent with the current work that places the emphasis on chronotype rather than sleep proximity upon modifying plasticity. Perhaps these principles operate concurrently in all individuals, which would suggest that sleep proximity had some influence upon the plasticity measures – for instance, is plasticity "enhancement" at the circadian preferred time greatest when proximal to sleep? This would predict that plasticity and performance are greatest when the preferred time of day is most proximal to sleep. Broadly I am seeking for a way to reconcile both chronotype and sleep as important modifying factors upon plasticity.

It would also be helpful to know whether there was any seasonal variation in the recruitment and conduct of the experiments. The interest here being in seasonal light variation and its entrainment upon sleep-wake/circadian cycles. It would be reassuring to know or have stated that the experiments were randomly distributed across seasons on the different chronotypes.

Brain stimulation has been used to provide insight into synaptic function. One of the great strengths of this is that it allows plasticity to be measured in humans. However, as yet, there is not any direct evidence showing that stimulation affects synaptic function. There is good circumstantial evidence (i.e., those factors, e.g., pharmacological that modify plasticity also modify the effects of stimulation), but there is no direct evidence that stimulation in the living human modifies plasticity. It might be worth acknowledging the extent of the strength of evidence for brain stimulation as a measure of plasticity to provide further balance.

The sequence learning task uses a delay between the response and subsequent stimulus of 500ms. A delay of this duration has been associated with participants developing awareness for the sequence more easily than shorter delays (for example of 200ms). Yet, it doesn't appear as though any participant became aware of the sequence, and nor the criteria/criterion for their removal.

I wasn't quite sure what the statement "two different versions of the task were used in the two sessions" (see page 43) meant. I thought perhaps it meant that different sequences were used during the two sessions. If so, it would be helpful to know what those sequences were, if there were any triplets in common, and any effect of order. If not, does this mean that the same sequence was used during the two sessions – wouldn't this lead to an order effect with transfer of skill to the second session – and then what does it mean that different versions were used?

The duration of each trial in the sequence learning task was determined by the participant's response time. As a consequence, faster response times lead to shorter trials and more frequent movements. The greater learning during preferred times would lead to faster response times and more frequent movements. This then leads to a confound in the interpretation of the EEG work: are the differences due to learning, or the movement frequency associated with greater learning. Either way the EEG work provides nicely converging evidence with the behavioural work --- it is more an issue of being clear about what is shown by the EEG and the limits of interpretation.

Was there a correlation between the performance in the various behavioural tasks? An absence of a correlation would suggest that the effects of chronotype upon behaviour operate independently upon one another; alternatively, a correlation might suggest that the effects are really due to changes within one task (and associated circuits) driving changes in another task. Earlier work has suggested a relationship between working memory and sequence learning (for example the work by Racheal Seidler and colleagues).

The work seems to have implications for the design of further studies. For example, the removal of participants that have a clear chronotypes preference. The inclusion of such participants could perhaps reduce the appearance of a circadian influence on excitability, plasticity and behavioural performance?

Reviewer #3 (Remarks to the Author):

Salehinejad et al. investigated chronotype-dependent modulation of human brain function in a multimodal approach including electrophysiology, neurostimulation and cognition. I first want to acknowledge the large amount of work involved and multiple aspects considered as well as the exhaustive methodological explanation favouring replicability. There is however a fundamental misconception of the study protocol that invalidates all the chronotype-related interpretations and turns down my initial enthusiasm. Because of this, the intrinsic novelty of the topic and potential findings with respect to existing literature is questionable.
MAJOR ISSUES
1. The protocol does not allow to conclude on chronotype differences for the following reasons:
1.1. clock time of data acquisition was fixed at 8:30AM and 7PM. Chronotype not only differ in the preferred time of day to perform tasks but also in the sleep-wake schedule, particularly so in extreme

chronotype which, presumably, constituted part of the sample. This means that for a morning chronotype 8:30AM and 7PM can correspond to respectively, e.g. ~2.5h and 11h after wake up time, while for an evening time this could correspond to 2h BEFORE wake up time and 8.5h after wake up time. In other words the authors are not comparing comparable things across their 2 groups of interest. Nb: The term “preferred time” is therefore not applicable to this study, particularly for the evening type.
1.2. sleep-wake history was not controlled. There is no report of a regular sleep schedule prior to the study. This means that i) we cannot place any of the participant in time to response to the uncertainty reported in 1.1., ii) we do not know whether participants slept similarly between both experimental sessions which took place 1 week apart and iii) we do not know how much participants slept prior to the study when previous literature clear established the influence of lack of sleep on basic cortical function. It would not be surprising to have an evening participant sleeping less for the morning assessment and at different times in order to cope with the very early 8:30AM session
1.3. we do not know whether any regular sleep would be the preferred schedule or an imposed schedule due to social obligation or because the experimenter imposed a fixed schedule.
This means that when comparing chronotypes, the authors may be in fact assessing the impact of time-of-day / elapse time wake from ~2h before wake up time (i.e. following partial sleep loss) to ~11h after wake up time across 2 groups of participants that completd part of the protocol. In support of this assumption (which I cannot proof or disproof due to lack of information), part of the data of evening type in the evening correspond to an intermediate between morning and evening session in morning time, when considering values in absolute terms (e.g. Fig 2a, Fig 3 b-c). This also implies that a lot of the potential novelty of the manuscript (i.e. chronotype) is removed.
2. The reproducibility of the positioning of the equipment across session is unclear. Most TMS systems come nom with neuronavigation which can guarantee high reproducibility of the TMS coil position across sessions. It seems that such a system was not used here such it is not possible to assess this aspect.
3. It seems that the statistical model used did not include important covariates such as sex, age and BMI (which is linked overall health status). Statistics need to be recomputed with at least these covariates included. This applies also to correlation analyses between behaviour and neurophysiology which cannot only consist in Pearson’s like approach. In addition, although the authors report using mixed repeated measure ANOVA in their statistics they do not state whether subject was the random factor or whether other factors were set as random. Likewise, the type of correlation applied between repeated measures is not specified.

OTHER ISSUES
- Beyond the major issue raised above the rationale for the choice of 8:30AM and 7PM is not provided
- The reference list should mention the original Horne and Ostberg publication for chronotype assessment. It’ll be much clearer to the reader that may take the questionnaire as the Munich Chronotype Questionnaire.
- The type of power mag stimulator needs to be specified
- Ambient light levels during the experiments need to be specified as light may directly impact alertness and therefore potentially basic brain function.
- The percentage of hits included in the 3-back task has be provided. In addition, accuracy is not fully appropriate for n-back performance assessment. D-prime is more appropriate to take into account the unbalanced repartition of target and non-target items.
- Accuracy is not a standard outcome measure of the Stroop task but rather RT variability between congruent and incongruent trials. The author should focus on the latter.

Authors' Response to the Review Comments

Journal: Nature Communications

Manuscript #: NCOMMS-20-13170-T

Title of Paper: Cognitive functions and respective parameters of human brain physiology are chronotype-dependent

Authors: MA Salehinejad, M Wischnewski, E Ghanavati, M Mosayebi-Samani, M-F Kuo, and MA Nitsche

Reviewers' comments:

Reviewer #1 (Remarks to the Author):

This study was performed to examine the effects of chronotype on motor cortical physiology, plasticity and behaviour. The authors demonstrated an interaction between chronotype and time of day for motor cortical excitability and paired-pulse TMS measures of excitation and inhibition, which taken together suggest that subjects had decreased excitability / increased inhibition at their non-preferred time of day. The time of day also had a significant effect on plasticity induction with tDCS, with the largest effects being seen at the preferred time of day. Finally, chronotype had significant effects on both motor learning and working memory tasks.

This is an impressive study involving a substantial degree of data collection to answer an interesting question in several different ways. It adds substantially to the literature by extending our understanding of the neurophysiological underpinnings of chronotypic differences. I have a few comments and suggestions for the authors, which I hope will aid the manuscript.

RESPONSE: We appreciate the reviewer's time and effort in reading our work and the positive evaluation s/he made. The comments are insightful and improved the quality of our manuscript. In what follows, we provide a one-by-one response to each comment and revised the main text accordingly. For the convenience of the reviewer, we pasted the revised version of the text here as well highlighted in blue.

Major Points

1. I take the authors point, but I am not sure that the link between stimulation-induced neuroplasticity and motor learning is as well as established as they suggest (line 110). I think that this is a stretch and should be rephrased.

RESPONSE: We agree with the reviewers' point and rephrased the sentence [please see p. 5, highlighted in blue].

REVISED text, P. 5:

In this study, we first systematically investigated the impact of chronotype and time-of-day effects on *cortical excitability* and stimulation-induced *neuroplasticity* in the model of the human motor cortex. In the next step, we explored how chronotype affects performance on a *motor learning* task which is associated with motor cortical plasticity[1].

2. Given the variability inherent in tDCS protocols, how was the study powered? I think more details would be helpful here.

RESPONSE: In the methods participant section (page 35), we now describe in detail the power analysis and how we determined sample size based on previous tDCS work (e.g., Minarik et al., 2016) [please see p. 33, highlighted in blue].

REVISED text, P.35:
Sample size was calculated a-priori based on a power analyses which showed that for a medium effect size (partial eta squared=0.10) (suggested for NIBS studies[2]), a minimum of 24 subjects is required to achieve 95% power at an alpha of 0.05 for the primary statistical test of a mixed model ANOVA design. We increased the sample size to 32 to fully counterbalance task order in each group (N=16) and compensate for unforeseen variability and dropouts.

3. Given the importance of circadian rhythms, did the authors ensure that they kept records of sleep/wake cycles, caffeine intake, (nicotine intake?) other things that might affect a person's circadian rhythm? Was sleepiness or equivalent taken into account?

RESPONSE: Thank you for the comment. Yes, we controlled for sleep duration, sleep/wake cycle, caffeine intake, nicotine intake, and other potentially interfering factors. All participants were non-smokers [please see page 35-36 highlighted in blue]. Regarding the sleep/wake cycle and sleepiness, we added a new section in the methods explaining how we controlled for these factors [please see page 37-38, highlighted in blue and green].

REVISED text, P.35-36:
All participants were right-handed non-smokers and underwent a medical screening to verify no history of neurological diseases, epilepsy or seizures, medication, metal implants, and current pregnancy. Each participant took part in a test TMS session to become acquainted with experiencing stimulation and understanding the study protocol. Female subjects were not examined during the luteal phase of the menstrual cycle (around week 3 following menstruation) to ensure that hormonal changes that affect cortical excitability [3] would not interfere with their chronotype. Caffeine intake and other factors potentially interfering with cortical excitability (e.g., massive physical activity) were controlled before each experimental session and in case the respective criteria were not met, these sessions were postponed.

REVISED text, P.37-38:
Experiment timing, sleep/wake cycle, and light variation
Timing of the experiments was determined based on the average of sleep times and level of activity of early and late chronotypes based on previous studies [4] as well as daily life routine schedules. For morning sessions, the start time was chosen based on the norm start of schooling and working (8:00 in the morning). This is also a time when both chronotypes have the lowest levels of activity [4]. The evening time (19:00) was specified based on the time when both chronotypes have a comparable level of activity according to previous dataset profiles [4]. Both groups were instructed to go to bed (at around 22:00-23:00), and get up in the morning at identical times of the day, and have at least 8 hours of sleep for morning sessions ($mean_{ECs}$ = 8.41, $mean_{LCs}$ = 8.11). For the evening session, participants were allowed to go to bed at their preferred time but before 24:00 and were also allowed to have more than 8 hours of sleep if needed ($mean_{ECs}$ = 8.36, $mean_{LCs}$ = 8.38). All participants were strongly recommended to comply with the sleep/wake cycle instruction and in case of irregularities, poor quality of sleep, and sleepiness (measured by participants' rating before each session), the experimental session was postponed to another day. Subjective sleepiness of participants and their alertness were evaluated with the Karolinska sleepiness scale (KSS)[5] which measures sleepiness in a 1-10 Likert-type scale (supplementary materials). No significant difference was observed between the sleepiness ratings of both early and late chronotypes in the morning sessions ($mean_{ECs}$ = 3.2, $mean_{LCs}$ = 3.71; t = 1.61, p = 0.11) and evening sessions ($mean_{ECs}$ = 3.58, $mean_{LCs}$ = 3.65; t = 0.25, p = 0.80). Due to the variability of seasonal light, experimental sessions did not take place in summer when the difference between sunrise and sunset is largest in Germany. For other months, the experiment was scheduled to be randomly distributed across other seasons in both groups. Furthermore, ambient light was kept constant (around 500 lux) across all experimental sessions in the morning and evening. In the cognitive sessions, tasks and EEG recordings were conducted in a sound-proof shielded room with constant temperature of 24-25°C for morning and evening sessions.

4. Given the previously reported effects of several factors, including a number of genetic variants (e.g BDNF phenotype) the results presented in Figure 3 look remarkably homogenous. Could the authors comment on why this might be, compared with similar data (answering different questions) from other labs?

RESPONSE: Thank you for this comment. Our tDCS results are indeed homogeneous at the group level, this however does not exclude interindividual variability [please see below scatterplots of individual absolute data]. We think that stimulation at the optimal time, with sufficient sleep, and control of interfering factors are potential reasons for the relatively homogenous pattern of response in both preferred and non-preferred times. These data suggest that if tDCS is applied in a carefully controlled situation and with optimal brain states, then we can expect to have homogenous results. We discuss this also in the discussion [p. 25, highlighted in green and blue] as an implication for the variability of results in NIBS research.

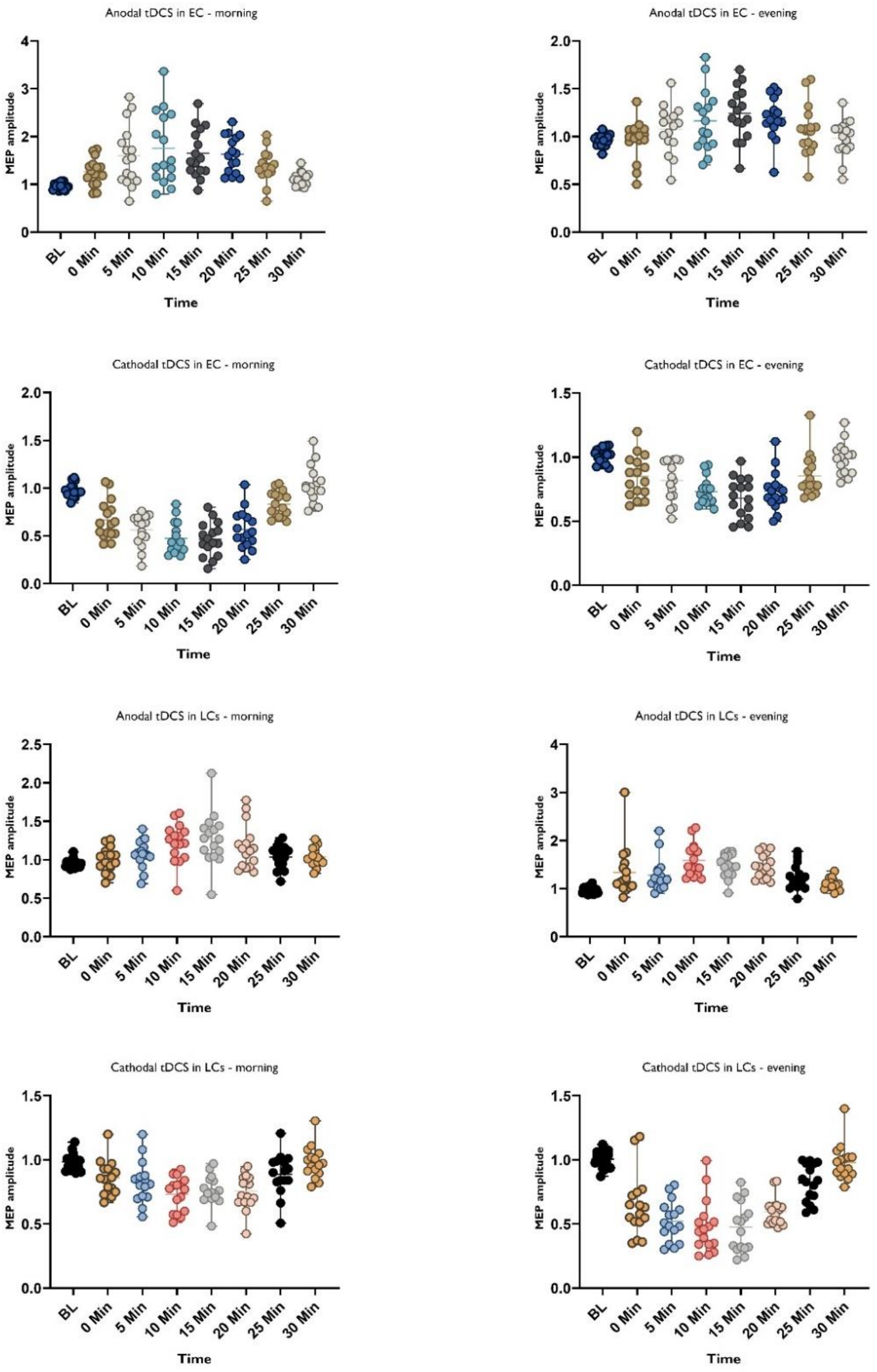

REVISED text, P.25:
The findings of this study have specific implications for the field of human neurophysiology and cognitive neuroscience as well as broad implications for human behavior in healthy and clinical populations. “Time-of-day” and chronotype are important, but rarely studied determinants of cortical plasticity induction by NIBS techniques[6, 7]. Our results show that cortical excitability and neuroplasticity are chronotype-dependent in humans. It is tempting to speculate that chronotype could account for variability in the efficacy of NIBS, and might explain partially heterogeneous effects in previous studies. This assumption might be likewise relevant for the performance of various cognitive tasks and suggest screening or considering its modulatory effects. Being studied at the optimal time of day, having sufficient sleep and control of interfering factors might enhance homogeneity of results, as our data suggest.

5. There is some evidence that chronotype affects other brain metrics that might then affect those demonstrated here, for example, DTI and grey matter volumes (e.g. Rosenberg et al., Neuroimage 2014; Rosenberg et al., BS&F 2018). How would more/less grey matter interact with the effects of tDCS, for example? The current model shown in figure 3 is for a standard brain, but the current density (and hence, probably, the effectiveness) must depend on the individual anatomy, which may be affected by chronotype. I understand that the authors will not have the data to answer this directly, but a discussion would be useful.

RESPONSE: Thank you for the comment. We agree with the reviewer’s point. The purpose of the 3D-modeling of the current flow was simply to show to the broad readership electrical fields induced by the intervention. We thus did not discuss the modeling results in the main text but just mentioned it in the legend of Fig. 3. Nevertheless, as suggested, we mention the point of chronotype-dependent brain anatomy differences, and their possible impact on stimulation effects now in the discussion [please see p. 26-27, highlighted in blue]. We also added a clarification to the methods section to avoid misconceptions about the meaning of the modelling [please see methods page 43, highlighted in blue].

REVISED text, P.26-27:
Our physiological measures were based on the motor system and indirect measures of the involved neurotransmitters, yet they suggest systematic effects of chronotype at the whole-brain level. Interestingly, chronotype-specific individual differences in brain anatomy (e.g. grey matter volume) have been described by some studies[8], which could affect tDCS-induced neuroplasticity induction either in a facilitatory or inhibitory way. It is unlikely that differences of brain structure were the driving force of the results because both groups showed enhanced plasticity at specific times of the day congruent with their respective chronotype. It was beyond the scope of our study to investigate chronotype-specific differences of brain structure, if any. This, however, would be an interesting question for future neuroimaging studies to add additional evidence to the findings of current study.

REVISED text, P.43:
Based on the randomized condition, anodal, cathodal, or sham tDCS with 1 mA intensity were applied for 7 min with 15 s ramp up/down at the beginning and end of stimulation. For the sham condition, stimulation was delivered for 30 s, with a 30 s ramp up and down. Using this procedure, participants are not able to distinguish between real and sham tDCS[9]. We simulated electrical current flow in the head induced by this protocol to show how the primary motor cortex is affected by tDCS (Fig. 3a). The model is based on the standard head model which does not take into account potential structural differences (e.g. white matter) in the brains of different chronotypes [8]. The model should thus be taken into account for illustrative purposes only.

6. The authors have numerical data on the DMEQ which presumably could be used to quantify the degree of “evening preference” or morning preference. Are there correlations between the major findings (particularly the physiology and the behaviour) and this DMEQ score? While I understand the study was not powered to examine this, I think it might be of interest, and would strengthen the arguments if such a relationship was present.

RESPONSE: Thank you for making this point. We had to limit our words to meet the journal guidelines which is why not all data are presented. In the methods, however we mentioned that both groups were moderate types. We now added the numerical data too [please see page 36-37, highlighted in blue]. We calculated the respective correlations for each chronotype separately as suggested. There was no significant correlation between DMEQ scores and mean MEP amplitudes obtained from each stimulation condition. Similarly, no significant correlations were found between DMEQ scores and cortical excitability, except for cortical inhibition obtained by the SICI protocol. The correlations between task performance and DMEQ scores were neither significant, except for Stroop accuracy. Given that the study was not powered to examine this (as the reviewer put it) and that this outcome measure was not significantly different across groups in the morning and evening, we would prefer not to add these results to the manuscript. We added these data however as **appendix 1** at the end of the response letter [please see end of this letter].

REVISED text P.36-37:
The questionnaire shows high reported reliability and has a significant correlation with circadian rhythm-related hormonal changes, including melatonin[10]. In addition to the MEQ, we defined the dichotomous chronotype phenotype by asking two identical questions ("Are you naturally a night person or a morning person?")[11]. Individuals with discordant or neutral responses to both were excluded. The mean scores of chronotype for the early and late chronotypes were 62.25±3.47 and 35.37±4.31, showing that both groups represent moderate early and late chronotypes.

REVISED text, supplementary materials:

1.1. Correlation between chronotype score, physiological parameters, and behavior

We also explored potential correlations between the participants' scores on DMEQ (i.e., circadian preference) and major outcome measures for both groups separately. No significant correlations were observed between DMEQ scores and cortical excitability except for intracortical inhibition (measured by SICI) of ECs obtained in the morning, which was positively correlated with DMEQ scores (r= 0.658, p=0.006). No significant correlations were neither found between DMEQ scores and neuroplasticity, or major outcome measures of the behavioral tasks.

Minor points.

1. The title probably needs rephrasing – cognitive function and their physiological parameters are chronotype-dependent? The grammar doesn't quite work at the moment, though I have no issue with the content.
RESPONSE: Thank you for your suggestion. The title is indeed "*Cognitive functions and **respective** parameters of human brain physiology are chronotype-dependent*".

2. A minor point, but daytime would probably better be "time of day".
RESPONSE: Thank you for your suggestion. As far as we can see, both words are grammatically correct and we used both in the manuscript. In the results, we used the term "daytime" as a factor in the analyses. However, as "time of day" has more stress on time, which was an important variable in our study, we thought this would convey our points/argument better. But we would be willing to change terms if this reviewer insists.

3. While taking the IO slope as the dependent variable would reduce effects of differences in rMT – were there differences between the sessions in the rMT – both in terms of the amplitude of the MEPs evoked, and more interestingly, the % of stimulator output used to evoke them?
RESPONSE: Thank you very much for the comment. This point is important, and we have described in detail the threshold values of all TMS protocols, including MEP size at RMT intensity (for the I-O curve

protocol), %MSO for RMT and SI_{1mv} in the supplementary material [Please see section 1.1 and Tables S1, S2 in the supplementary materials highlighted in blue].

Revised text, supplementary material.

1.1. TMS protocols threshold values

MEP values of single-pulse conditions and other control conditions, as well as subject-specific baseline sensitivity to TMS (defined as the percentage of maximum stimulator output required for the SI1mV MEP amplitude), are summarized in Table S1. For each TMS protocol, we compared single-pulse condition MEPs (control condition) and the SI1mV obtained at different daytimes by a 2-factorial ANOVA with daytime (morning, evening) as the within-subject and chronotype (ECs, LCs) as the between-subject factor. The results of the respective ANOVAs show that the mean values of the single-pulse conditions MEP, MEP amplitude at RMT (for the I-O curve protocol), %MSO for RMT, and SI_{1mv} did not significantly differ between time of day and groups (Table S2). Only for AMT, the interaction of chronotype×daytime was significant, and post hoc t-tests revealed a significant difference between AMT values only for ECs across daytime (t = 3.58, *p* = 0.003).

Table S1: Baseline measurements of the cortical excitability protocols

**Protocol**	**measurement**	**daytime**	**Group**	
			**ECs**	**LCs**
Single-pulse MEP	SI 1_{mV} (%)	morning	51.31 ± 9.82	50.43 ± 8.46
		evening	50.50 ± 10.19	50.87 ± 7.91
RMT	%MSO	morning	42.87 ± 7.54	41.81 ± 6.88
		evening	41.68 ± 7.15	42.12 ± 7.09
AMT	%MSO	morning	35.18 ± 6.95	34.40 ± 8.23
		evening	33.53 ± 6.97	34.96 ± 8.18
I-O curve	MEP amplitude at RMT	morning	0.235 ± 0.213	0.197 ± 0.204
		evening	0.149 ± 0.091	0.200 ± 0.133
SICI-ICF	Single-pulse MEP	morning	1.073 ± 0.335	0.941 ± 0.152
		evening	0.910 ± 0.342	0.980 ± 0.121
I-wave facilitation	Single-pulse MEP	morning	1.020 ± 0.341	1.041± 0.119
		evening	1.237 ± 0.512	1.178 ± 0.438
SAI	Single-pulse MEP	morning	1.237 ± 0.512	1.178 ± 0.438
		evening	1.194 ± 0.269	1.195 ± 0.175

Note: MEP = motor-evoked potential; ECs = early chronotypes; LCs = late chronotypes; SI_{1mv} (%) = maximum stimulator output (%MSO) required for the SI1mV MEP amplitude; I-O curve = input-output curve; SICI-ICF = short latency intracortical inhibition and facilitation; SAI = short-latency afferent inhibition; RMT = resting motor threshold; AMT = Active motor threshold.

Table S2: Results of 2 × 2 ANOVAs for control MEPs (RMT intensity MEP for I-O curve, single-pulse MEP for other protocols), SI1mV, and %MSO for RMT/AMT.

**Protocol**	**Measurement**	**Factor**	***df***	***F***	***p***
Single-pulse MEP	SI_{1mV}	daytime	1	0.12	0.724
		chronotype	1	1.40	0.244
		daytime×chronotype	1	0.001	0.938
RMT	%MSO	daytime	1	0.86	0.359
		chronotype	1	0.01	0.901
		daytime×chronotype	1	2.54	0.120
AMT	%MSO	daytime	1	3.23	0.082
		chronotype	1	0.015	0.903
		daytime×chronotype	1	13.30	<**0.001**

I-O curve	MEP amplitude at RMT	daytime	1	1.04	0.315
		chronotype	1	0.02	0.882
		daytime×chronotype	1	1.169	0.288
SICI-ICF	Single-pulse MEP	daytime	1	0.98	0.329
		chronotype	1	0.22	0.641
		daytime×chronotype	1	2.65	0.113
I-wave facilitation	Single-pulse MEP	daytime	1	0.23	0.631
		chronotype	1	0.83	0.367
		daytime×chronotype	1	0.37	0.547
SAI	Single-pulse MEP	daytime	1	0.017	0.895
		chronotype	1	0.112	0.739
		daytime×chronotype	1	0.090	0.765

Note: MEP = motor-evoked potential; chronotype = early (ECs) and late (LCs) chronotypes; SI_{1mv} (%) = maximum stimulator output (%MSO) required for the SI1mV MEP amplitude; I-O curve = input-output curve; SICI-ICF = short latency intracortical inhibition and facilitation; SAI = short-latency afferent inhibition; RMT = resting motor threshold; AMT = active motor threshold.

4. I appreciate this group's transparency with their statistics, and the ANOVA tables are helpful with such complex approaches. Please could these be added somewhere for the other results too? Supplementary tables would be fine, but it is helpful to see them all for clarity.

RESPONSE: Thank you for this suggestion. We have space for another Table according to journal guidelines and added statistics for the cognition and EEG part as suggested (please see below and on page 66, highlighted in blue). For neuroplasticity results, we mentioned all statistics either in the text [page 11, highlighted in blue] or the figure legend [page 31, highlighted in blue], unfortunately, an additional table would exceed the limitations of the journal.

Table S2: Results of ANOVAs for cognitive task performance and respective ERP components.

**Task**	**Measurement**	**Factor**	***df***	***F***	***p***	ηp^2
SRTT (motor learning)	BL 5 vs 6 RT	learning blocks	1	70.31	**<0.001**	0.708
		chronotype	1	0.41	0.527	0.014
		daytime	1	0.015	0.902	0.001
		learning blocks×chronotype	1	4.07	0.053	0.123
		learning blocks×daytime	1	0.77	0.399	0.025
		learning blocks×chronotype×daytime	1	22.91	**<0.001**	0.441
	BL 7 vs 6 RT	learning blocks	1	72.18	**<0.001**	0.731
		chronotype	1	0.11	0.733	0.004
		daytime	1	0.35	0.852	0.001
		learning blocks×chronotype	1	1.81	0.188	0.059
		learning blocks×daytime	1	0.50	0.824	0.002
		learning blocks×chronotype×daytime	1	10.76	**0.003**	0.271
	P300	learning blocks (BL5-7)	1	29.20	**<0.001**	0.502
		chronotype	1	3.29	0.080	0.102
		daytime	1	0.62	0.438	0.21
		learning blocks×chronotype	1	0.33	0.716	0.011
		learning blocks×daytime	1	0.97	0.384	0.032
		learning blocks×chronotype×daytime	1	8.11	**<0.001**	0.219
3-back (working memory)	Accuracy	chronotype	1	0.881	0.365	0.029
		daytime	1	0.117	0.735	0.004
		daytime×chronotype	1	11.70	**0.002**	0.281

	d prime	chronotype<br>daytime<br>daytime×chronotype	1<br>1<br>1	0.60<br>0.22<br>12.46	0.444<br>0.642<br>**<0.001**	0.020<br>0.007<br>0.293
	RT	chronotype<br>daytime<br>daytime×chronotype	1<br>1<br>1	0.01<br>0.70<br>1.08	0.978<br>0.407<br>0.305	0.001<br>0.02<br>0.035
	P300-Pz	chronotype<br>daytime<br>daytime×chronotype	1<br>1<br>1	1.13<br>0.01<br>14.49	0.295<br>0.927<br>**<0.001**	0.037<br>0.001<br>0.326
	P300-Cz	chronotype<br>daytime<br>daytime×chronotype	1<br>1<br>1	0.28<br>2.05<br>11.19	0.600<br>0.162<br>**0.002**	0.009<br>0.064<br>0.272
Stroop (selective attention)	Stroop block RT	chronotype<br>daytime<br>congruency<br>daytime×chronotype<br>congruency×chronotype<br>congruency×daytime<br>congruency×chronotype daytime	1<br>1<br>1<br>1<br>1<br>1<br>1	0.15<br>0.48<br>75.34<br>20.84<br>1.02<br>1.94<br>0.85	0.696<br>0.490<br>**<0.001**<br>**<0.001**<br>0.320<br>0.174<br>0.362	0.005<br>0.016<br>0.715<br>0.410<br>0.033<br>0.061<br>0.028
	Congruent trials RT	chronotype<br>daytime<br>daytime×chronotype	1<br>1<br>1	0.72<br>1.21<br>14.30	0.791<br>0.279<br>**<0.001**	0.002<br>0.039<br>0.323
	Incongruent trials RT	chronotype<br>daytime<br>daytime×chronotype	1<br>1<br>1	0.25<br>0.02<br>23.08	0.619<br>0.870<br>**<0.001**	0.008<br>0.001<br>0.435
	RT variability	chronotype<br>daytime<br>daytime×chronotype	1<br>1<br>1	0.79<br>0.35<br>25.60	0.378<br>0.558<br>**<0.001**	0.026<br>0.012<br>0.460
	N200-Fz (congruent trials)	chronotype<br>daytime<br>daytime×chronotype	1<br>1<br>1	1.58<br>3.06<br>19.04	0.218<br>0.090<br>**<0.001**	0.052<br>0.096<br>0.396
	N200-Fz (incongruent trials)	chronotype<br>daytime<br>daytime×chronotype	1<br>1<br>1	2.56<br>0.09<br>8.66	0.120<br>0.767<br>**0.006**	0.081<br>0.003<br>0.230
AX-CPT (sustained attention)	Accuracy	chronotype<br>daytime<br>daytime×chronotype	1<br>1<br>1	0.38<br>0.07<br>13.81	0.541<br>0.788<br>**<0.001**	0.013<br>0.002<br>0.315
	RT	chronotype<br>daytime<br>daytime×chronotype	1<br>1<br>1	0.08<br>5.21<br>14.55	0.769<br>**0.031**<br>**<0.001**	0.003<br>0.146<br>0.331
	P300-Pz	chronotype<br>daytime<br>daytime×chronotype	1<br>1<br>1	0.69<br>1.44<br>5.46	0.412<br>0.239<br>**0.026**	0.023<br>0.046<br>0.154

Note: BL = Block; RT = Reaction Time; SRTT = Serial Reaction time Task. d prime = this index of performance is calculated as d = ZHit – ZFA where Hit represents the proportion of hits when a signal is present (hits/(hits + misses)), also known as the hit rate, and FA represents the proportion of false alarms when a signal is absent (false alarms/(false alarms + correct negative)), the false-alarm rate. Significances are marked in **bold** (where $p < 0.05$).

5. I am not sure I understand the statement "Female subjects were not examined during the menstrual period to ensure hormonal changes would not interfere with their chronotype. Hormonal changes occur throughout the menstrual cycle and not just during bleeding. Please could the authors clarify?

RESPONSE: Thank you for the comment. We did not mean "during bleeding" but during the time when cortical excitability is shown to be affected by the menstrual cycle. We added more information to clarify [please see page 35 highlighted in blue].

REVISED text P.35:

Female subjects were not examined during the luteal phase of the menstrual cycle (around week 3 following menstruation) to ensure that hormonal changes that affect cortical excitability [3] would not interfere with their chronotype.

6. Lines 296-8 I understand that there is not space to put all the results in the main text, but it would be useful to briefly summarise the key points here.

RESPONSE: Thank you for the comment. We summarized the key points as suggested

REVISED text P. 17:

We also explored the association between motor learning, and plasticity by calculating correlations between MEPs amplitudes and motor sequence learning. Briefly, we found a positive correlation between anodal tDCS effects (MEP amplitude enhancement) and sequence learning (block 6 - 5 RT difference) in the evening for LCs (r=0.543, p=0.017). No significant correlation between sequence learning and tDCS-induced plasticity was found in ECs (for detailed results see Supplementary Material)

7. Line 365 – should read chronotypes

REVISED text P.21:

Here we showed a converging impact of chronotypes on time-of-day dependent behavioural/cognitive performance of healthy individuals, and demonstrated the physiological foundations of these effects by daytime-dependent cortical excitability, neuroplasticity, and brain information processing processes.

8. I like the idea of figure 6, but I am not sure that it adds anything to the study. Could it be a graphical abstract? Or maybe there could be some mechanistic hypotheses added? At the moment it seems somewhat redundant.

RESPONSE: Thank you for the comment. We updated figure 6 with a proposed mechanistic hypothesis.

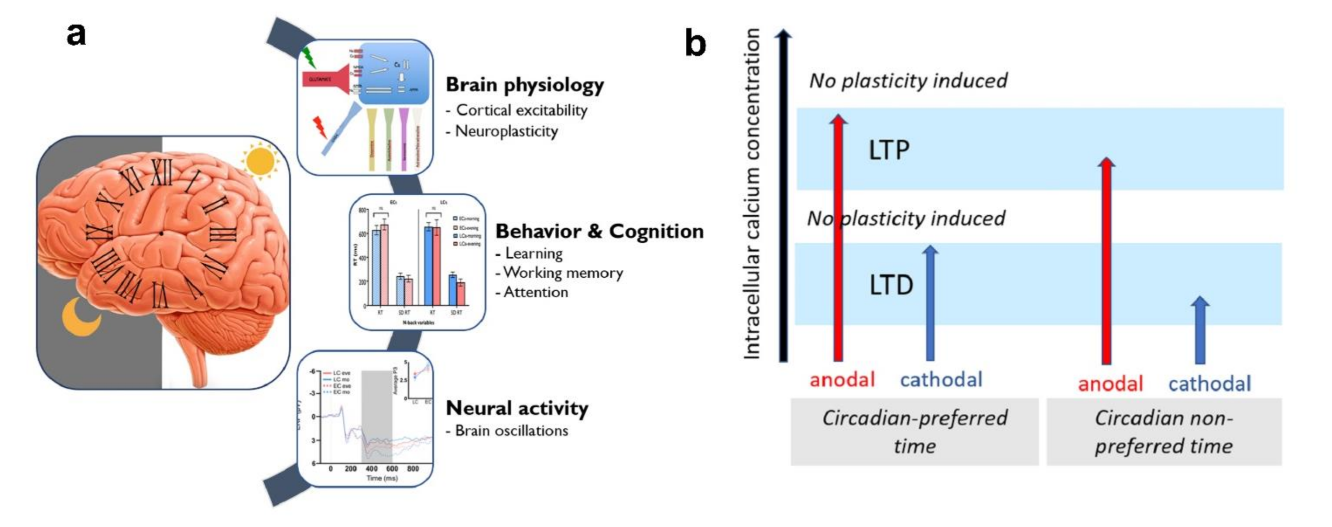

Fig. 6: **a**, A schematic illustration depicting the converging impact of chronotype on brain physiology, behavior, and cognition. **b**, Proposed mechanism of the neuroplasticity induction at circadian-preferred and non-preferred time based on the association between intracellular calcium concentration (x-axis) and induction of tDCS-induced neuroplastic changes. Gradual enhancement of calcium concentration can either induce LTD, LTP or no plasticity. The LTP-like plasticity induced by tDCS is linked to higher intracellular calcium concentration while LTD-like plasticity induced by tDCS is linked to a lower intracellular calcium concentration. It can be

assumed that intracellular calcium concentration at the optimal time of the day is at an optimal level leading to stronger tDCS-induced LTP/D like plasticity. This would be at least partially related to the higher glutamatergic and lower GABAergic activation at the circadian preferred time, shown by cortical excitability data.

9. Line 497 – should read “one week” I imagine.

REVISED text P.27:

Following the resting-EEG acquisition, participants performed motor learning and cognitive (working memory, attention) tasks in two randomly-assigned sessions at the same time in the morning and evening while their EEG was recorded (one-week interval).

Reviewer #2 (Remarks to the Author):

I really enjoyed reading this manuscript. It is very well written, thoughtful with a measured narrative based on a set of studies that were clearly a great deal of work examining multiple levels of the biological hierarchy from synaptic function to behaviour. The work is nicely illustrated and convincing. I have some comments that may strengthen the work further.

RESPONSE: We appreciate the reviewer's time and effort in reading our work, positive evaluation of our work, and the insightful comments s/he offered. In what follows, we provide a one-by-one response to each comment and revised the main text accordingly. For the convenience of the reviewer, we pasted the revised version of the text here as well in highlighted in green or blue.

1- Motor cortical excitability changed with the preferred time of day. As a consequence, are the differences in, for example, the double TMS experiments simply because a different stimulation intensity is being used and so what is being reported is simply a different position within the double pulse curve? For example, and admittedly an extreme example for illustrative purposes – at the preferred time of day 30% of machine output might be applied but at the non-preferred time of day 80% of machine output is used (due to changes in excitability). Then a change in the curve is really because there is a change in stimulation intensity (due to excitability changes) as opposed to some additional change that is specifically revealed in the double pulse experiments. Is it potentially possible that if the machine output had stayed the same – the excitability would show a change (MEP magnitude with chronotype and time of day) – however the double pulse curve would not differ significantly? Thus, the differences in the double pulse curve are driven by changes in excitability (i.e., non-independent measure). I think this is an important issue, which regardless of the concern being misplaced (or not) needs discussed to help other readers.

RESPONSE: Thank you very much for the comment. Due to lack of space, we had described this information in the supplementary material. The threshold values for all TMS protocols including baseline/test-pulse sensitivity to TMS (defined as the percentage of maximum stimulator output required for the SI1mV MEP amplitude), MEP amplitude at RMT (for I-O curve protocol), %MSO for RMT, and SI1mv are presented in the supplementary material and there was no significant difference between these values. This rules out that changes in the excitability were due to changes of stimulation intensity [Please see section 1.1 and Tables S1, S2 in the supplementary materials highlighted in blue].

REVISED text, supplementary material:

1.1. TMS protocols threshold values

MEP values of single-pulse conditions and other control conditions, as well as subject-specific baseline sensitivity to TMS (defined as the percentage of maximum stimulator output required for the SI1mV MEP amplitude), are summarized in Table S1. For each TMS protocol, we compared single-pulse condition MEPs (control condition) and the SI1mV obtained at different daytimes by a 2-factorial ANOVA with daytime (morning, evening) as the within-subject and chronotype (ECs, LCs) as the between-subject factor. The results of the respective ANOVAs show that the mean values of the single-pulse conditions MEP, MEP intensity at RMT (for I-O curve protocol), %MSO for RMT, and SI_{1mv} did not significantly differ between time of day and groups (Table S2). Only for AMT, the interaction of chronotype×daytime was significant, and post hoc t-tests revealed a significant difference between AMT values only for ECs across daytime (t = 3.58, *p* = 0.003).

Table S1: Baseline measurements of the cortical excitability protocols

Protocol	measurement	daytime	Group	
			ECs	LCs
Single-pulse MEP	$SI\ 1_{mV}$ (%)	morning	51.31 ± 9.82	50.43 ± 8.46
		evening	50.50 ± 10.19	50.87 ± 7.91

RMT	%MSO	morning	42.87 ± 7.54	41.81 ± 6.88
		evening	41.68 ± 7.15	42.12 ± 7.09
AMT	%MSO	morning	35.18 ± 6.95	34.40 ± 8.23
		evening	33.53 ± 6.97	34.96 ± 8.18
I-O curve	MEP amplitude at RMT	morning	0.235 ± 0.213	0.197 ± 0.204
		evening	0.149 ± 0.091	0.200 ± 0.133
SICI-ICF	Single-pulse MEP	morning	1.073 ± 0.335	0.941 ± 0.152
		evening	0.910 ± 0.342	0.980 ± 0.121
I-wave facilitation	Single-pulse MEP	morning	1.020 ± 0.341	1.041± 0.119
		evening	1.237 ± 0.512	1.178 ± 0.438
SAI	Single-pulse MEP	morning	1.237 ± 0.512	1.178 ± 0.438
		evening	1.194 ± 0.269	1.195 ± 0.175

Note: MEP = motor-evoked potential; ECs = early chronotypes; LCs = late chronotypes; SI_{1mv} (%) = maximum stimulator output (%MSO) required for the SI1mV MEP amplitude; I-O curve = input-output curve; SICI-ICF = short latency intracortical inhibition and facilitation; SAI = short-latency afferent inhibition; RMT = resting motor threshold; AMT = Active motor threshold.

Table S2: Results of 2 × 2 ANOVAs for control MEPs (RMT intensity MEP for I-O curve, single-pulse MEP for other protocols), SI1mV, and %MSO for RMT/AMT.

**Protocol**	**Measurement**	**Factor**	***df***	***F***	***p***
Single-pulse MEP	SI_{1mV}	daytime	1	0.12	0.724
		chronotype	1	1.40	0.244
		daytime×chronotype	1	0.001	0.938
RMT	%MSO	daytime	1	0.86	0.359
		chronotype	1	0.01	0.901
		daytime×chronotype	1	2.54	0.120
AMT	%MSO	daytime	1	3.23	0.082
		chronotype	1	0.015	0.903
		daytime×chronotype	1	13.30	<**0.001**
I-O curve	RMT intensity MEP	daytime	1	1.04	0.315
		chronotype	1	0.02	0.882
		daytime×chronotype	1	1.169	0.288
SICI-ICF	Single-pulse MEP	daytime	1	0.98	0.329
		chronotype	1	0.22	0.641
		daytime×chronotype	1	2.65	0.113
I-wave facilitation	Single-pulse MEP	daytime	1	0.23	0.631
		chronotype	1	0.83	0.367
		daytime×chronotype	1	0.37	0.547
SAI	Single-pulse MEP	daytime	1	0.017	0.895
		chronotype	1	0.112	0.739
		daytime×chronotype	1	0.090	0.765

Note: MEP = motor-evoked potential; chronotype = early (ECs) and late (LCs) chronotypes; SI_{1mv} (%) = maximum stimulator output (%MSO) required for the SI1mV MEP amplitude; I-O curve = input-output curve; SICI-ICF = short latency intracortical inhibition and facilitation; SAI = short-latency afferent inhibition; RMT = resting motor threshold; AMT = active motor threshold.

2- It would be helpful to know time of sleep onset, time of waking, sleep duration, and the

duration from waking to the start of the experiment for each participant (if possible). For example, from information through sleep-logs. I am suggesting this for two reasons: (a) the relationship between sleep and plasticity is topical (see below) and (b) to provide further information about the circadian cycle of the different groups (i.e., the phase lag between the groups).

RESPONSE: Thank you for the suggestion. We did not force participants to go to bed at a specific time but defined a range in which subjects should have gone to sleep (from 22:00-2300). Also, participants had to have at least 8 h of sleep and we controlled for these parameters before each experimental session. Additionally, we measured participants' sleepiness before each session and results show no significant difference across groups. In case there was a significant sleepiness, the session was held [please see page 37, highlighted in blue and yellow]. More details are explained in response to the next comment.

REVISED text, p 37:
Experiment timing, sleep/wake cycle, and light variation
Timing of the experiments was determined based on the average of sleep times and level of activity of early and late chronotypes based on previous studies [4] as well as daily life routine schedules. For morning sessions, the start time was chosen based on the norm start of schooling and working (8:00 in the morning). This is also a time when both chronotypes have the lowest levels of activity [4]. The evening time (19:00) was specified based on the time when both chronotypes have a comparable level of activity according to previous dataset profiles [4]. Both groups were instructed to go to bed (at around 22:00-23:00), and get up in the morning at identical times of the day, and have at least 8 hours of sleep for morning sessions ($mean_{ECs}$ = 8.41, $mean_{LCs}$ = 8.11). For the evening session, participants were allowed to go to bed at their preferred time but before 24:00 and were also allowed to have more than 8 hours of sleep if needed ($mean_{ECs}$ = 8.36, $mean_{LCs}$ = 8.38). All participants were strongly recommended to comply with the sleep/wake cycle instruction and in case of irregularities, poor quality of sleep, and sleepiness (measured by participants' rating before each session), the experimental session was postponed to another day. Subjective sleepiness of participants and their alertness were evaluated with the Karolinska sleepiness scale (KSS)[5] which measures sleepiness in a 1-10 Likert type scale (supplementary materials). No significant difference was observed between the sleepiness ratings of both early and late chronotypes in the morning sessions ($mean_{ECs}$ = 3.2, $mean_{LCs}$ = 3.71; t = 1.61, p = 0.11) and evening sessions ($mean_{ECs}$ = 3.58, $mean_{LCs}$ = 3.65; t = 0.25, p = 0.80).

3- Sleep and plasticity have been linked. For example, sleep deprivation impairs the same brain stimulation markers of plasticity used in the current work. Plasticity might then reasonably be expected to be higher in the hours following sleep than after substantial time awake. At first sight this would appear inconsistent with the current work that places the emphasis on chronotype rather than sleep proximity upon modifying plasticity. Perhaps these principles operate concurrently in all individuals, which would suggest that sleep proximity had some influence upon the plasticity measures – for instance, is plasticity "enhancement" at the circadian preferred time greatest when proximal to sleep? This would predict that plasticity and performance are greatest when the preferred time of day is most proximal to sleep. Broadly I am seeking for a way to reconcile both chronotype and sleep as important modifying factors upon plasticity.

RESPONSE: Thank you for the suggestion. The reviewer's point makes sense and we had in mind that sleep could interfere with chronotype-related results in case participants do not have enough sleep especially for the morning sessions. As explained in response to the previous comment, we controlled for sleep-wake cycle and obtained a sleepiness rating before each session and added respective information in the revision [please see previous comment p.37, highlighted in blue and yellow]. Furthermore, this concern was raised also by the other reviewers. In order to address this concern, we provide new data from another project in which we measured the impact of lack of sleep on the same physiological parameters. Our results showed a completely different pattern of cortical excitability and neuroplasticity as a result of sleep deprivation. This shows that plasticity will be affected differently

under lack of sleep compared to preferred vs non-preferred time of day. We also analyzed resting EEG theta activity which is a marker of sleep pressure and found no significant differences [please see p. 21. Highlighted in yellow]. For more details on these new analyses please refer to our response to the 3rd reviewer.

REVISED text, p 21:

No difference in sleepiness rating and physiological marker of sleep pressure across groups

The different sleep-wake cycle of early and late chronotypes indicates that both groups could have different sleepiness which can interfere with chronotype-specific effects on brain physiology and cognition. To resolve this concern, we controlled the sleep-wake cycle and sleep duration of both groups within a defined range (see methods for details) and rated participants' sleepiness with the Karolinska Sleepiness Scale before each test session. All participants had at least 8 h of sleep before each session (see methods). The results of ANOVA showed no significant interaction of chronotype×daytime (F_1=1.03, p=0.325) or main effects of chronotype and daytime. This indicates that there was no significant difference between the sleepiness ratings in or between both groups across different times of day. Furthermore, we analyzed resting EEG theta oscillations, which is a marker of sleep pressure[12] in both groups in the morning and evening and found no significant differences in the theta oscillations when we compared both groups in the morning and evening. No significant differences were neither observed in each group across different times of the day. The same pattern of results was observed for alpha oscillations (supplementary materials).

4- It would also be helpful to know whether there was any seasonal variation in the recruitment and conduct of the experiments. The interest here being in seasonal light variation and its entrainment upon sleep-wake/circadian cycles. It would be reassuring to know or have stated that the experiments were randomly distributed across seasons on the different chronotypes.

RESPONSE: Thank you for the comment. We added respective information in the text [Please see p.37-38, highlighted in green].

REVISED text, p 37-38:

Due to the variability of seasonal light, experimental sessions did not take place in summer when the difference between sunrise and sunset is largest in Germany. For other months, the experiment was scheduled to be randomly distributed across other seasons in both groups. Furthermore, ambient light was kept constant (around 500 lux) across all experimental sessions in the morning and evening. In the cognitive sessions, tasks and EEG recordings were conducted in a sound-proof shielded room with constant temperature of 24-25°C for morning and evening sessions.

5- Brain stimulation has been used to provide insight into synaptic function. One of the great strengths of this is that it allows plasticity to be measured in humans. However, as yet, there is not any direct evidence showing that stimulation affects synaptic function. There is good circumstantial evidence (i.e., those factors, e.g., pharmacological that modify plasticity also modify the effects of stimulation), but there is no direct evidence that stimulation in the living human modifies plasticity. It might be worth acknowledging the extent of the strength of evidence for brain stimulation as a measure of plasticity to provide further balance.

RESPONSE: Thank you for the suggestion. We absolutely agree and mention this limitation in the discussion [Please see p.27, highlighted in green].

REVISED text, p 27:

Finally, although NIBS allows us to causally modify and induce neuroplasticity in humans non-invasively, it is worth to acknowledge that the evidence for synaptic plasticity induction by NIBS, including tDCS, comes from animal and pharmacological studies, and is thus indirect[13-15].

6- The sequence learning task uses a delay between the response and subsequent stimulus of 500ms. A delay of this duration has been associated with participants developing awareness for the sequence more easily than shorter delays (for example of 200ms). Yet, it

doesn't appear as though any participant became aware of the sequence, and nor the criteria/criterion for their removal.

RESPONSE: Thank you for the comment. We adopted the task features from versions used in previous works of our group which showed that it measures implicit motor learning [16]. Furthermore, 500 ms is the commonly used interval in other studies with SRT tasks as well [17, 18]. But it is worth noting that participants are instructed to respond as fast as possible and RT is the primary outcome of interest. We explained in the task description [page 46, highlighted in green] about the awareness criterion and excluded the data if the sequence was correctly identified. Indeed, one EC participant correctly identified the sequence and his/her data was excluded as shown in Fig.4 legend [page 32, highlighted in green].

REVISED text, p 32:
All pairwise comparisons are calculated using *Student's* t-test. *n*=31 (15 ECs, 16 LCs; data of one EC participant excluded due to sequence awareness). All error bars represent the s.e.m across participants.

REVISED text, p 46:
Participants were not told about the repeating sequence and at the end of the session, they were asked whether they noticed a sequence and if so, to write the sequence in order to assess explicit learning of the task. In such case, respective data were excluded from the final analysis.

7- I wasn't quite sure what the statement "two different versions of the task were used in the two sessions" (see page 43) meant. I thought perhaps it meant that different sequences were used during the two sessions. If so, it would be helpful to know what those sequences were, if there were any triplets in common, and any effect of order. If not, does this mean that the same sequence was used during the two sessions – wouldn't this lead to an order effect with transfer of skill to the second session – and then what does it mean that different versions were used?

RESPONSE: Thank you for the comment. We clarified this point [please see pages 46, highlighted in green].

REVISED text, p 46:
Two different sequences of the task, with no overlapping parts, and comparable difficulties, were presented in the two sessions in counterbalanced order.

8- The duration of each trial in the sequence learning task was determined by the participant's response time. As a consequence, faster response times lead to shorter trials and more frequent movements. The greater learning during preferred times would lead to faster response times and more frequent movements. This then leads to a confound in the interpretation of the EEG work: are the differences due to learning, or the movement frequency associated with greater learning. Either way the EEG work provides nicely converging evidence with the behavioural work --- it is more an issue of being clear about what is shown by the EEG and the limits of interpretation.

RESPONSE: Thank you for your comment. We have to clarify that in this task the frequency of finger movements is fixed across sessions. Therefore, faster RT does not mean more frequent movements but faster movement due to enhanced learning, which would however also enhance movement frequency. This is a point worth considering and we highlight it in the EEG results [please see p. 16, highlighted in green].

REVISED text, p. 15:
Together, these results highlight the relationship between the circadian-preferred time and recruitment of motor learning-specific electrophysiological processes that are associated with performance. One aspect to consider in interpreting the ERP amplitude enhancements at the circadian-preferred time is however that these could reflect enhanced learning, but also the faster frequency of movements caused

by learning-related faster RT.

9- Was there a correlation between the performance in the various behavioural tasks? An absence of a correlation would suggest that the effects of chronotype upon behaviour operate independently upon one another; alternatively, a correlation might suggest that the effects are really due to changes within one task (and associated circuits) driving changes in another task. Earlier work has suggested a relationship between working memory and sequence learning (for example the work by Racheal Seidler and colleagues).

RESPONSE: Thank you for suggestion. This is an interesting point however, out study was not powered for this type of correlational analyses. Nonetheless, we explored the suggested correlations and added the results to the supplementary material (please refer to supplementary material, page 11, highlighted in green). No correlations were observed at the whole group data (both ECs and LCs). In addition, in early chronotypes, no significant correlations were found between working memory and motor learning in the morning, but a negative correlation between performance speed in both tasks was observed for LCs in the evening. For the other tasks, we found no significant relevant correlations as well. Overall, these data show no robust correlations between the performance of different tasks.

REVISED text, supplementary material, p 12:

1.1. Correlation between cognitive tasks

We also explored correlations of performance between the cognitive tasks. No significant correlations were observed between working memory and motor learning at the group level and in ECs alone. In LCs, however, a significant correlation was found between working memory RT in the evening and the sequence learning block 6 of the SRTT in the evening (r_{BL5}= -0.513, p=0.042; r_{BL6}= -0.614, p=0.011) indicating faster RT in both tasks in the evening were associated. For the other tasks, no significant correlations were found. Overall, these data show no robust correlations of performance between the different tasks.

10- The work seems to have implications for the design of further studies. For example, the removal of participants that have a clear chronotypes preference. The inclusion of such participants could perhaps reduce the appearance of a circadian influence on excitability, plasticity and behavioural performance?

RESPONSE: Thank you for your comment. We absolutely agree and had briefly mentioned this in the discussion, but added more details in the revision [please see p. 25, highlighted in green]. These results suggest that chronotypes should be screened in NIBS studies that investigate brain physiology and cognition. We don't necessarily suggest exclusion of early or late chronotypes, but e.g. adapting the experiment time at the circadian preferred time, or identify a time of the day which works for all chronotypes reasonably well. This is a novel finding in the field of NIBS that calls for studying potential factors that contribute to variability of results in NIBS research.

Revised text, p 25:

The findings of this study have specific implications for the field of human neurophysiology and cognitive neuroscience as well as broad implications for human behavior in healthy and clinical populations. "Time-of-day" and chronotype are important, but less-studied determinants of cortical plasticity induction by NIBS techniques [6, 7]. Our results show that cortical excitability and neuroplasticity are chronotype-dependent in humans. It is tempting to speculate that chronotype could account for variability of NIBS effects and might explain partially heterogeneous effects in previous studies. This assumption might be likewise relevant for the performance of various cognitive tasks and suggests to screen or consider these modulatory effects.

Reviewer #3 (Remarks to the Author):

Salehinejad et al. investigated chronotype-dependent modulation of human brain function in a multimodal approach including electrophysiology, neurostimulation and cognition. I first want to acknowledge the large amount of work involved and multiple aspects considered as well as the exhaustive methodological explanation favouring replicability. There is however a fundamental misconception of the study protocol that invalidates all the chronotype-related interpretations and turns down my initial enthusiasm. Because of this, the intrinsic novelty of the topic and potential findings with respect to existing literature is questionable.

RESPONSE: Thank you for reading our work and providing important comments and suggestions. We understand the concerns raised by the reviewer and in what follows we address them. Some of the concerns were due to a misunderstanding caused by a lack of information we provided in the original version. Due to the word limit and the study size (that includes 4 different large datasets), we had to choose the most relevant information. We now added this information in the revision. Furthermore, we have additional data from another dataset (in what follows) as well as new data from this dataset (EEG) that addresses the major concern of this reviewer. For the reviewer's convenience, we pasted the changes of the main text here as well.

MAJOR ISSUES
1. The protocol does not allow to conclude on chronotype differences for the following reasons:
1.1. clock time of data acquisition was fixed at 8:30AM and 7PM. Chronotype not only differ in the preferred time of day to perform tasks but also in the sleep-wake schedule, particularly so in extreme chronotype which, presumably, constituted part of the sample. This means that for a morning chronotype 8:30AM and 7PM can correspond to respectively, e.g. ~2.5h and 11h after wake up time, while for an evening time this could correspond to 2h BEFORE wake up time and 8.5h after wake up time. In other words the authors are not comparing comparable things across their 2 groups of interest. Nb: The term "preferred time" is therefore not applicable to this study, particularly for the evening type.
1.2. sleep-wake history was not controlled. There is no report of a regular sleep schedule prior to the study. This means that i) we cannot place any of the participant in time to response to the uncertainty reported in 1.1., ii) we do not know whether participants slept similarly between both experimental sessions which took place 1 week apart and iii) we do not know how much participants slept prior to the study when previous literature clear established the influence of lack of sleep on basic cortical function. It would not be surprising to have an evening participant sleeping less for the morning assessment and at different times in order to cope with the very early 8:30AM session
1.3. we do not know whether any regular sleep would be the preferred schedule or an imposed schedule due to social obligation or because the experimenter imposed a fixed schedule. This means that when comparing chronotypes, the authors may be in fact assessing the impact of time-of-day / elapse time wake from ~2h before wake up time (i.e. following partial sleep loss) to ~11h after wake up time across 2 groups of participants that completd part of the protocol. In support of this assumption (which I cannot proof or disproof due to lack of information), part of the data of evening type in the evening correspond to an intermediate between morning and evening session in morning time, when considering values in absolute terms (e.g. Fig 2a, Fig 3 b-c). This also implies that a lot of the potential novelty of the manuscript (i.e. chronotype) is removed.

Thank you for the comments. In what follows, we address the concerns raised by this reviewer by adding additional information, data, and analyses:

First (in response to 1.1), We mention in the methods that all participants were *moderate* early or late chronotype and extreme types were excluded to reduce sleep-wake schedule variability. We did not

explicitly mention this in the original version of the manuscript due to space limits but now clarified this in the revision and provide numerical scores of DMEQ scores as well [please see page 36-37, and below highlighted in blue].

Second (in response to 1.1, 1.2, 1.3), As we clarify in the revision, both groups had to have at least 8 h of sleep. Furthermore, subjective sleepiness was rated before each session in the morning and evening and results showed no significant difference neither in the morning nor in the evening sessions across groups [please see p.37, highlighted in blue and yellow]. According to the two-process model of sleep regulation [19, 20], the magnitude of sleepiness and alertness is a function of sleep (S) and circadian rhythms (C). Non-significant differences in sleepiness, therefore, indicate that process S (sleep pressure) was not significantly different across groups. Furthermore, before each testing session, the time to go to bed and wake up were standardized and identical for all subjects. This should resolve the concerns about "*different sleep-wake cycle*" and "*partial sleep loss*" especially for the evening types.

Third (in response to 1.3.), sleep-wake history was indeed controlled 24 hours before each experimental session. Participants had to have 8 hours of sleep before each experimental session, and subjective sleepiness was assessed (no significant difference between groups). In case this criterion was not met and participants felt sleepy, respective sessions were postponed (we had to cancel 15 sessions during the experiment due to not meeting this criterion, proofed by high sleepiness ratings in the KSS questionnaire).

Fourth (in response to 1.1 & 1.3), this experiment was meant to examine the impact of chronotype and not sleep, which is why participants had standard sleep duration, and sleep times before the experimental sessions. Testing each group at different times in the morning and evening, makes it challenging to compare the results of each group due to different timing, and moreover, "optimal" times might gradually differ between individuals (and there is no objective criterion to make clear decisions), thus in our opinion there is no ideal solution in studies that have a "chronotype-based paradigm"[20]. Therefore, we decided to pick a fixed time for each group that has the closest correspondence to their circadian preference according to previous works, as described later. Any other time of measurement would raise the same concerns, according to the reviewer's points. Ensuring that participants had at least 8 h of sleep and evaluating subjective sleepiness before each session was the most reasonable and feasible option to rule out any potential impact of sleep loss or pressure. Nonetheless, we mention this point in the limitations of the study [please see p.26, highlighted in yellow].

Revised text, page 36-37: Definite evening (16-30), moderate evening (31-41), intermediate or neutral (42-58), moderate morning (59-69) and definite morning (70-86) are the five chronotype categories identified by the MEQ. The questionnaire shows high reported reliability and has a significant correlation with circadian rhythm-related hormonal changes, including melatonin[10]. In addition to the MEQ, we defined the dichotomous chronotype phenotype by asking two identical questions ("Are you naturally a night person or a morning person?")[11]. Individuals with discordant or neutral responses to both were excluded. The mean scores of chronotype for the early and late chronotypes were 62.25±3.47 and 35.37±4.31, showing that both groups represent moderate early and late chronotypes. Extreme chronotypes were excluded to reduce sleep-wake schedule variability.

Revised text, page 37: Timing of the experiments was determined based on the average of sleep times and level of activity of early and late chronotypes based on previous studies [4] as well as daily life routine schedules. For morning sessions, the start time was chosen based on the norm start of schooling and working (8:00 in the morning). This is also a time when both chronotypes have the lowest levels of activity [4]. The evening time (19:00) was specified based on the time when both chronotypes have a comparable level of activity according to previous dataset profiles [4]. Both groups were instructed to go to bed (around 22:00-23:00), and get up in the morning at identical times of the day, and have at least 8 hours of sleep for morning sessions ($mean_{ECs}$ = 8.41, $mean_{LCs}$ = 8.11). For the evening session, participants were allowed to go to bed at their preferred time but before 24:00 and were also allowed to

have more than 8 hours of sleep if needed ($mean_{ECs}$ = 8.36, $mean_{LCs}$ = 8.38). All participants were strongly recommended to comply with the sleep/wake cycle instruction and in case of irregularities, poor quality of sleep, and sleepiness (measured by participants' rating before each session), the experimental session was postponed to another day. Subjective sleepiness of participants and their alertness were evaluated with the Karolinska sleepiness scale (KSS)[5] which measures sleepiness in a 1-10 Likert type scale (supplementary materials). No significant difference was observed between the sleepiness ratings of both early and late chronotypes in the morning sessions ($mean_{ECs}$ = 3.2, $mean_{LCs}$ = 3.71; t = 1.61, p = 0.11) and evening sessions ($mean_{ECs}$ = 3.58, $mean_{LCs}$ = 3.65; t = 0.25, p = 0.80).

Revised text (page 26): It is important to consider that our study design has a chronotype-based design which is appropriate for studying the influence of circadian rhythmicity peaks on performance variations in optimal and non-optimal time of day[20]. While this approach has the advantage of studying cognition and brain function under normal day-night condition, it cannot take into account the interplay between clock- and sleep-dependent processes into account[20]. Considering that the sleep-wake cycle in different chronotypes is not identical, this might raise concerns about its potentially confounding effects. According to the two-process model of sleep regulation, the magnitude of sleepiness and alertness is determined by the interacting influence of sleep pressure, and circadian rhythms. The control over timing to go to bed and sleep duration during this experiment and non-significant difference between the sleepiness ratings before each session across groups indicates that potential sleep loss or different sleep-wake cycles do not account for the observed effects. Our resting-EEG data also confirmed no significant difference between theta oscillations, as a marker of sleep pressure[12], in the morning or evening sessions across groups.

Finally (overall response to major concerns), we have a new dataset available that hopefully resolves the reviewer's major concern about confounding effects of different sleep-wake cycles and resultant sleepiness. The data of this work come from a larger research project in which we systematically investigated the impact of both "***chronotype***" and "***lack of sleep***" on brain physiology, and cognition. We recently finished the second part of the project about the impact of sleep deprivation on the same physiological parameters we obtained for the chronotype study. Data of this work explicitly show that *lack of sleep* has a fundamentally different impact on brain physiology than chronotype, which addresses the concern about the potential confounding impact of different sleep-wake schedules of early vs late chronotypes and partial sleep loss of the latter group (although we controlled for this and reported sleepiness ratings above). In this new dataset, 14 of 30 participants were evening-types and they underwent the same measures (cortical excitability, neuroplasticity) after sufficient sleep and after lack of sleep. The measurement time was approximately identical (8:30 in the morning). We provide results of these groups of participants in the figures below. As shown in **Fig 1**, physiological parameters under lack of sleep have a completely different pattern compared to non-preferred time in late chronotypes (**Fig. 2**). Sleep deprivation leads to a significant increase and decrease of intracortical facilitation and inhibition respectively (**Fig. 1A-D**), while an opposite pattern occurs when participants are explored at their non-preferred circadian time (i.e., morning) (**Fig. 2A-D**). Similarly, qualitatively different results were obtained for neuroplasticity induction. Sleep deprivation diminished LTP-like and reversed LTD-like plasticity to LTP-like plasticity due to cortical *hyperactivation* in late chronotypes (**Fig. 1E-F**). Being at non-preferred time (morning), however, induces both LTP and LTD-like plasticity, but to a lesser extent than during the preferred time due to cortical *underactivation* (**Fig. 2F**).

We believe these results clearly suggest that if late-chronotypes had lack of sleep in the morning sessions (due to a different sleep-wake cycle) that could interfere with chronotype effects, physiological parameters should have been altered differently than those observed in the chronotype study. With these new data, we think that the results of the chronotype study are completely novel and have not been shown by previous works. We do hope this new dataset resolved the reviewer's major concerns and if s/he thinks adding this data to the manuscript is required, we would be willing to add results of these 14 participants from our sleep experiment.

Specific comments:

In response to comment 1.1. The term “*preferred*” was simply chosen to convey our point throughout the text because chronotype is defined as circadian-*preference*. Furthermore, according to the MEQ (item 11), the best time that fits the internal clock of evening types is 7:00-9:00 PM [21] which shows that our evening time measure can be labeled “*preferred*” time for the evening type. If this reviewer, however, prefers a different wording, we would be happy to change.

In response to comment 1.3. We are not sure if we understand the reviewers’ point based on his/her assumption (in support of…). But for figure 2a, the values are indeed absolute, this was a typo. We are sorry for that and corrected it in the revised version [please see Fig 2, page 10].

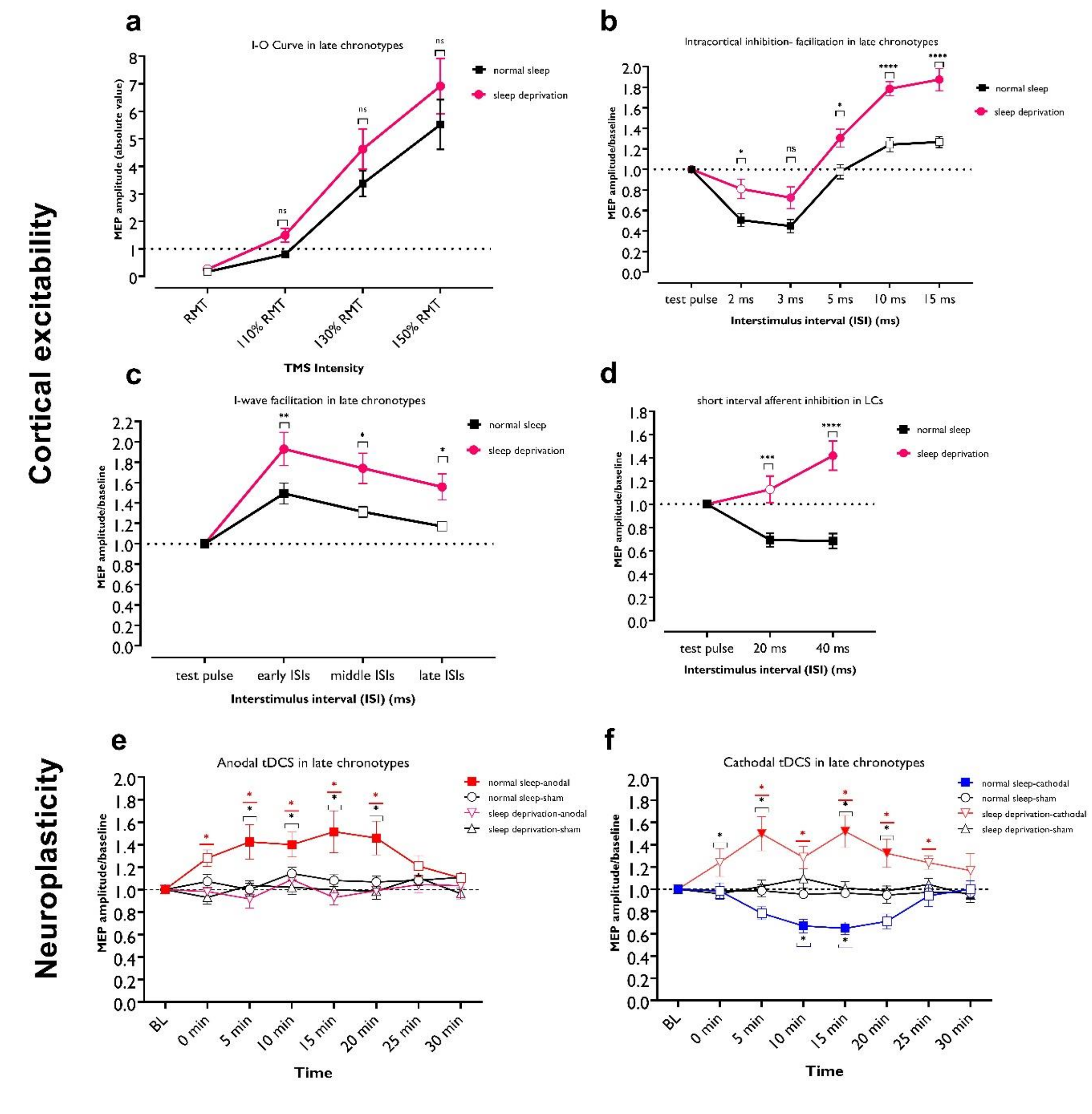

Fig. 1. Cortical excitability and neuroplasticity induction following sufficient sleep vs sleep deprivation in late chronotypes. **a**, Global cortico-spinal excitability monitored by MEP amplitudes at different TMS intensities via the I-O curve protocol. **b**, Intracortical inhibition and facilitation measured by the SICI-ICF paired-pulse TMS protocol. **c**, GABA-dependent intracortical inhibition after normal sleep vs sleep deprivation. **d**, Short latency afferent inhibition after normal sleep vs sleep deprivation. **e**, LTP-like plasticity induction in the motor cortex after normal sleep vs sleep deprivation. **f**, LTD-like plasticity induction in the motor cortex after normal sleep vs sleep deprivation. All pairwise comparisons were calculated using Bonferroni correction for multiple comparisons (n = 14). All error bars represent the standard error of means (s.e.m.). Filled symbols

represent a significant difference of MEP amplitudes compared to the respective test pulses. Asterisks represent statistically significant comparisons between sleep conditions. in **e** and **f** the black asterisks [*] indicate a significant difference between the real vs sham tDCS conditions, and the red asterisks [*] indicate a significant difference between respective timepoints of tDCS condition after normal sleep vs sleep deprivation.

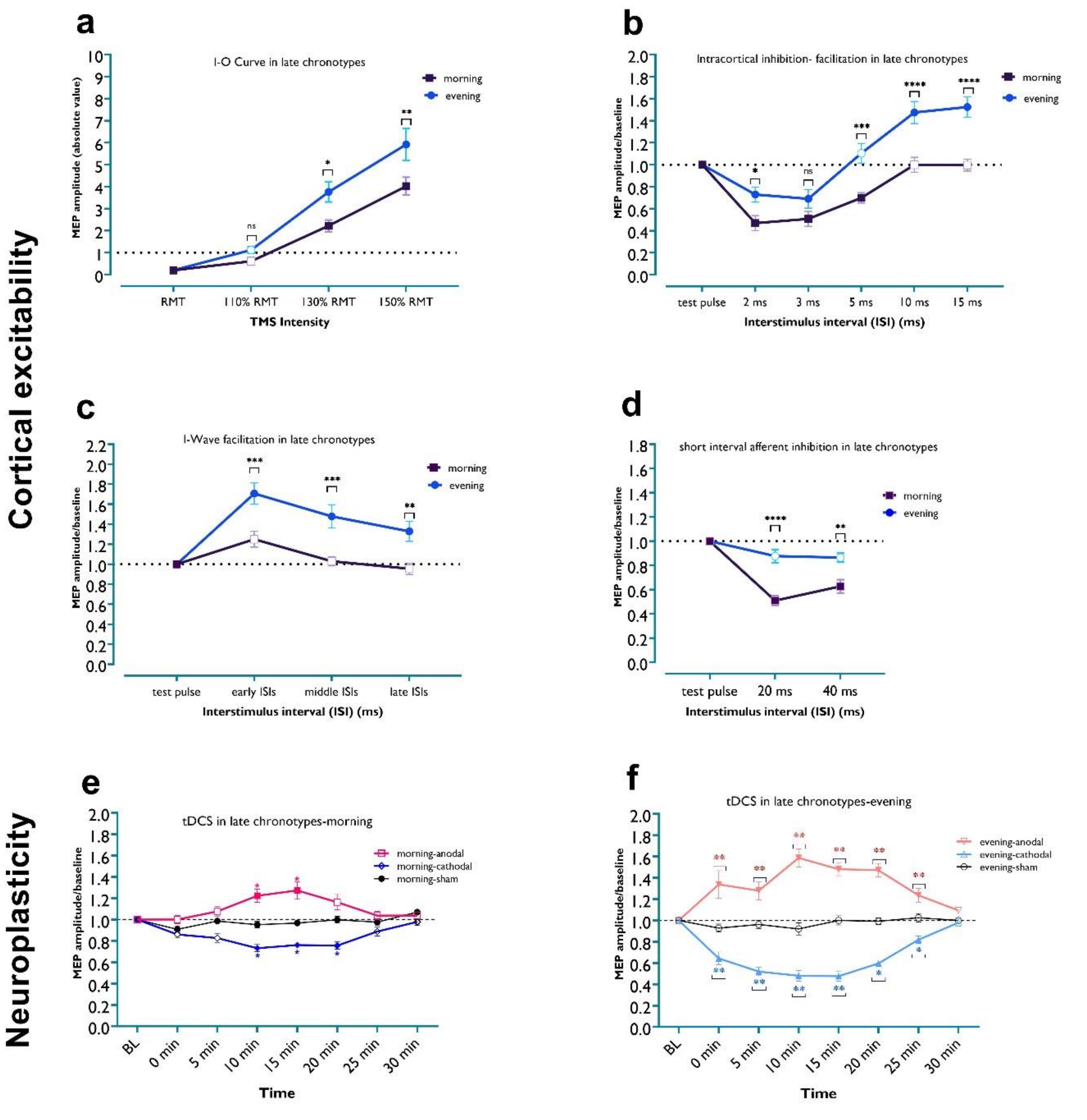

Fig. 2. Cortical excitability and neuroplasticity induction in late chronotypes at preferred and non-preferred times. a, Global cortico-spinal excitability monitored by MEP amplitudes at different TMS intensities via the I-O curve protocol. **b** Intracortical inhibition and facilitation measured by the SICI-ICF paired-pulse (pp)TMS protocol. **c** I-Wave facilitation for monitoring GABA-dependent intracortical inhibition. **d** Inhibitory effect of peripheral nerve stimulation on motor cortical inhibition, as measured by SAI. **e, f** post-tDCS cortical excitability alterations after anodal, cathodal, and sham stimulation at the circadian non-preferred (**e**) and preferred times (**f**) in LCs (*n* = 16). All pairwise comparisons are calculated using the Bonferroni correction for multiple comparisons. All error bars represent the standard error of means (s.e.m) across participants. In a-d, asterisks represent statistically significant comparisons and filled symbols represent a significant difference of MEP amplitudes compared to the respective test pulses. In **e** and **f** symbol [*] indicates a significant difference between the real vs sham tDCS conditions, and the floating symbol [**] indicates a significant difference between respective timepoints of tDCS condition at the circadian-preferred vs circadian non-preferred times. In **e** and **f**, filled symbols represent a significant difference of MEP amplitudes compared to the baseline MEP.

Evidence from resting EEG data: In addition to the data shown above, resting-EEG supports our claim on non-significant differences in the sleep-wake cycle and sleep loss of both groups in the morning. According to the two-process model [22], sleep regulation is determined by the interaction between sleep/wake cycle (Process S) and circadian rhythms (Process C). Process S, which sleep pressure, increases during wakefulness and declines during sleep. This process has several physiological markers including NREM sleep, SWA, and EEG. Theta activity in the awake state is a marker of the rise of S [19] occurring during sleep pressure. Recent sleep deprivation studies confirmed theta activity as a marker of sleep pressure as well [12]. We recorded resting EEG data in each group in the morning (8:30) and evening (19:00) sessions. We compared resting EEG theta oscillations of both ECS and LCs in the morning and the results show no significant difference. This shows that LCs in the morning were not under sleep pressure due to partial sleep loss. Theta oscillations for each group across the morning and evening sessions were significant neither. We would be happy to add this EEG new analysis to the main results [please see p. 21, highlighted in yellow].

Electrode		*t*	*Adjusted P Value*
Fz	ECs-morning vs. LCs-morning theta oscillations	0.33	>0.999
Cz	ECs-morning vs. LCs-morning theta oscillations	0.48	>0.999
Pz	ECs-morning vs. LCs-morning theta oscillations	1.22	>0.999
Fz	ECs-evening vs. LCs-evening theta oscillations	2.18	0.181
Cz	ECs-evening vs. LCs-evening theta oscillations	1.80	0.433
Pz	ECs-evening vs. LCs-evening theta oscillations	0.50	>0.999
Fz	ECs-morning vs. ECs-evening theta oscillations	2.07	0.238
Cz	ECs-morning vs. ECs-evening theta oscillations	1.27	>0.999
Pz	ECs-morning vs. ECs-evening theta oscillations	1.51	0.790
Fz	LCs-morning vs. LCs-evening theta oscillations	0.21	>0.999
Cz	LCs-morning vs. LCs-evening theta oscillations	0.05	>0.999
Pz	LCs-morning vs. LCs-evening theta oscillations	0.18	>0.999

Revised text, page 21:

No difference in sleepiness ratings and physiological markers of sleep pressure across groups

The different sleep-wake cycle of early and late chronotypes indicates that both groups could have different sleepiness which can interfere with chronotype-specific effects on brain physiology and cognition. To resolve this concern, we controlled the sleep-wake cycle and sleep duration of both groups within a defined range (see methods for details) and rated participants' sleepiness with the Karolinska Sleepiness Scale before each test session. All participants had at least 8 h of sleep before each session (see methods). The results of ANOVA showed no significant interaction of chronotype×daytime (F_1=1.03, p=0.325) or main effects of chronotype and daytime. This indicates that there was no significant difference between the sleepiness ratings in or between both groups across different times of day. Furthermore, we analyzed resting EEG theta oscillations, which is a marker of sleep pressure[12] in both groups in the morning and evening and found no significant differences in the theta oscillations when we compared both groups in the morning and evening. No significant differences were neither observed in each group across different times of the day. The same pattern of results was observed for alpha oscillations (supplementary materials).

2. The reproducibility of the positioning of the equipment across session is unclear. Most TMS systems come nom with neuronavigation which can guarantee high reproducibility of the TMS coil position across sessions. It seems that such a system was not used here such it is not possible to assess this aspect.

RESPONSE: Thank you for the comment. We acknowledge the reviewer's concern and added this as one of the limitations of our work [please see page 25, highlighted in yellow]. We do not use neuronvigation in our lab when the target region is the motor cortex, but use the physiological criterion of hot spot determination in accordance with many other groups (e.g. Pascual-Leone group: Pascual-Leone et

al. 1994, *Brain*, Pascual-Leone et al. 2005, *Annual Rev Neuroscience*, Rossi et al. 2009, *Clin Neurophysiol,* Rossi et al. 2015, *Clin Neurophysiol*; Ulf Ziemann group: Ulf Ziemann et al. 2009, *Brain Stimul*, Permoli et al. 2017, *Neuroimage*; Leonard G Cohen group: Bütefisch et al. 2000, *PNAS*, Dayan et al. 2018, *Brain Structure & Function*, Joseph Classen group: Stefan et al. 200, *Brain*, other relevant papers: Muellbacher et al. 2002, *Nature*). As described in the methods for coil positioning, we mark the coil position with a waterproof pen and make sure the coil position and angle are kept constant, which is an established procedure used by most expert groups in the field. The hotspot was identified in each session and quite stable between sessions, which is supported by identical stimulation intensities required to evoke the target MEP size between conditions. Specifically, there was no significant difference between the baseline MEPs (absolute values) as well as the %MSO (maximum stimulator output required for the SI1mV MEP amplitude and RMT values) between sessions and conditions, which supports the reliability of this approach. Afterall, if slight alterations of coil positions occurred between sessions, this would add variance, but follow no systematic trend and thus would not affect the results systematically. Whether in this case neuronavigation, which cannot take into account slight changes of motor maps between sessions, would be advantageous is unclear, because it shifts the neurophysiological criterion to a neuroanatomical criterion, which is not more precise. We hope that by making these points and mentioning the lack of neuronavigation as a limitation, the reviewer's concern is resolved.

Revised text, page 25:

Furthermore, although there was no significant difference between baseline MEPs and % of MSO across conditions in both groups, which supports the reliability of the acquired data, the use of neuronavigation for stimulation of the motor cortex might have been advantageous to further enhance reproducibility of the TMS coil position.

Revised text, supplementary materials:

Table S1: Baseline measurements of the cortical excitability protocols

Protocol	measurement	daytime	Group	
			ECs	LCs
Single-pulse MEP	SI 1_{mV} (%)	morning	51.31 ± 9.82	50.43 ± 8.46
		evening	50.50 ± 10.19	50.87 ± 7.91
RMT	%MSO	morning	42.87 ± 7.54	41.81 ± 6.88
		evening	41.68 ± 7.15	42.12 ± 7.09
AMT	%MSO	morning	35.18 ± 6.95	34.40 ± 8.23
		evening	33.53 ± 6.97	34.96 ± 8.18
I-O curve	MEP amplitude at RMT	morning	0.235 ± 0.213	0.197 ± 0.204
		evening	0.149 ± 0.091	0.200 ± 0.133
SICI-ICF	Single-pulse MEP	morning	1.073 ± 0.335	0.941 ± 0.152
		evening	0.910 ± 0.342	0.980 ± 0.121
I-wave facilitation	Single-pulse MEP	morning	1.020 ± 0.341	1.041± 0.119
		evening	1.237 ± 0.512	1.178 ± 0.438
SAI	Single-pulse MEP	morning	1.237 ± 0.512	1.178 ± 0.438
		evening	1.194 ± 0.269	1.195 ± 0.175

Note: MEP = motor-evoked potential; ECs = early chronotypes; LCs = late chronotypes; SI_{1mv} (%) = maximum stimulator output (%MSO) required for the SI1mV MEP amplitude; I-O curve = input-output curve; SICI-ICF = short latency intracortical inhibition and facilitation; SAI = short-latency afferent inhibition; RMT = resting motor threshold; AMT = Active motor threshold.

Table S2: Results of 2 × 2 ANOVAs for control MEPs (RMT intensity MEP for I-O curve, single-pulse MEP for other protocols), SI1mV, and %MSO for RMT/AMT.

Protocol	Measurement	Factor	*df*	*F*	*p*
Single-pulse MEP	SI_{1mV}	daytime	1	0.12	0.724

		chronotype	1	1.40	0.244
		daytime×chronotype	1	0.001	0.938
RMT	%MSO	daytime	1	0.86	0.359
		chronotype	1	0.01	0.901
		daytime×chronotype	1	2.54	0.120
AMT	%MSO	daytime	1	3.23	0.082
		chronotype	1	0.015	0.903
		daytime×chronotype	1	13.30	<**0.001**
I-O curve	MEP amplitude at RMT	daytime	1	1.04	0.315
		chronotype	1	0.02	0.882
		daytime×chronotype	1	1.169	0.288
SICI-ICF	Single-pulse MEP	daytime	1	0.98	0.329
		chronotype	1	0.22	0.641
		daytime×chronotype	1	2.65	0.113
I-wave facilitation	Single-pulse MEP	daytime	1	0.23	0.631
		chronotype	1	0.83	0.367
		daytime×chronotype	1	0.37	0.547
SAI	Single-pulse MEP	daytime	1	0.017	0.895
		chronotype	1	0.112	0.739
		daytime×chronotype	1	0.090	0.765

Note: MEP = motor-evoked potential; chronotype = early (ECs) and late (LCs) chronotypes; SI_{1mv} (%) = maximum stimulator output (%MSO) required for the SI1mV MEP amplitude; I-O curve = input-output curve; SICI-ICF = short latency intracortical inhibition and facilitation; SAI = short-latency afferent inhibition; RMT = resting motor threshold; AMT = active motor threshold.

3. It seems that the statistical model used did not include important covariates such as sex, age and BMI (which is linked overall health status). Statistics need to be recomputed with at least these covariates included. This applies also to correlation analyses between behaviour and neurophysiology which cannot only consist in Pearson's like approach. In addition, although the authors report using mixed repeated measure ANOVA in their statistics they do not state whether subject was the random factor or whether other factors were set as random. Likewise, the type of correlation applied between repeated measures is not specified.

RESPONSE: Thank you for this comment. To answer it, we need to explain some aspects of the design in more detail. We aimed for a relatively homogenous group of participants with respect to the mentioned covariates. We have an **equal number of males and females** in each group (N = 8 of 16, 16 of 32 in total) to prevent potential confounding effects of gender [page 35, highlighted in yellow]. Most participants (25 of 32) were in their 20s (mean age 26.62±5.01) [page 35, highlighted in yellow]. We calculated BMI upon request of the reviewer and the BMI values are also homogenous (mean BMI 23.61±2.63). Of the 32 participants, 26 had a normal BMI (mean BMI 22.61±1.45) and 6 were borderline overweight (mean BMI 27.96±2.11). Please refer to the table below for more details. Furthermore, we computed correlations between these variables (age, sex, BMI) and outcome measures (cortical excitability protocols, neuroplasticity induction, and task performance) [please see below]. There were no significant correlations, which clearly speaks against confounding effects of these variables. We added these details to the revision [please see pages, 7, 11, 17 highlighted in yellow]. Correlation tables are added at the end of this letter (Appendix 2). We think with these data there is no need to add these variables as covariates as it makes the results even more complex without adding relevant information. Nevertheless, we can re-run the analyses with ANCOVAs in case the reviewer insists. With regard to the type of correlation, we used Pearson's product-moment correlation (between variables with interval scale) and Point-Biserial correlation in case one variable was dichotomous (e.g., gender). We are open for other types, if the reviewer prefers so (please specify).

Variable		ECs	LCs	*p*
Gender	Male (female)	8 (8)	8 (8)	N/A
Age	Mean±SD	24.56 (4.66)	28.68 (4.60)	0.364
BMI	Mean±SD	23.03 (2.37)	24.20 (2.81)	0.210

Regarding the 2nd point, we think that there is a misunderstanding as far as we understand the reviewer's point. We did not use linear mixed-models (LMM) (that require definition of fixed and random effects) **but** mixed-factorial **ANOVAs** with repeated measures (with between-subject and within-subject factors) for each dataset that have different number of factors. To be specific, we used mixed-factorial ANOVAs with repeated measures (ISI × daytime × chronotype) for the cortical excitability results, a mixed-factorial design with repeated-measures ANOVA (stimulation × timepoint × daytime × chronotype) for the neuroplasticity results, a mixed-factorial ANOVA with repeated-measures (block × daytime × chronotype) for the motor learning, a series of mixed-factorial ANOVAs (daytime × chronotype) for the cognitive task outcome variables, and finally similar mixed-factorial ANOVAs (daytime × chronotype) for the analysis of ERP components. We clarify this in the text as well [please see pages 49-51, highlighted in yellow], and are sorry for the misunderstanding. The last point of the comments (type of correlation for repeated measures) does in our understanding not apply for the same reason because it is for mixed-model GLM (that requires fixed and random effects) **but we** conducted mixed-factorial ANOVAs.

Revised text, page 7, 11, 17:

P. 7: These TMS protocols are based on different neurotransmitter systems related to cortical facilitation (glutamatergic) and inhibition (GABAergic, cholinergic)[23-25] (see Methods). No correlation was found between age, gender, BMI, and MEPs obtained from TMS protocols and thus these variables were not entered as covariates in the ANOVA analyses.

P.11: Side effects were minor and did not differ between intervention conditions except for the tingling and burning sensations (Supplementary Tables 4,5). There was no correlation between age, gender, BMI, and TMS-induced MEP and thus these variables were not entered as potential covariates in the ANOVA analyses.

P.17: All participants conducted the 3-back letter (working memory task), Stroop and AX-Continuous Performance tasks (AX-CPT) (attention tasks), during EEG recording at their circadian-preferred and non-preferred times (see Methods). No significant correlations were observed between the major outcome measures of task performance and demographic variables ruling out their potential confounding effects.

OTHER ISSUES

1- Beyond the major issue raised above the rationale for the choice of 8:30AM and 7PM is not provided

RESPONSE: Thank you for the comment. In the revision, we added the rationale for choosing 8:30 AM and 7:00 PM. First, we selected these times based on previous works that compared levels of activity in the early and late chronotypes[4] (please see the figure below) as well as the two-process model of sleep regulation [22]. According to the figure, 8:30 AM and 7:00 PM are roughly the times when both early and late chronotypes have relatively comparable levels of activity. Considering that both groups were moderate early and late chronotype and were required to have at least 8 h of sleep, their level of activity was even more comparable. Furthermore, in these times there is no (8:30 AM) or less (19:00) sleep pressure according two-process model of sleep regulation. The body hormonal changes that are related to sleep pressures (e.g., melatonin) also usually start to secret after 8-9:00 PM[26]. Lastly, the other rationale for choosing 8-8:30 for morning sessions was to generalize findings to daily life routine schedules (e.g., schooling time, work time). We added these details to the methods [please see page 35, highlighted in blue and yellow].

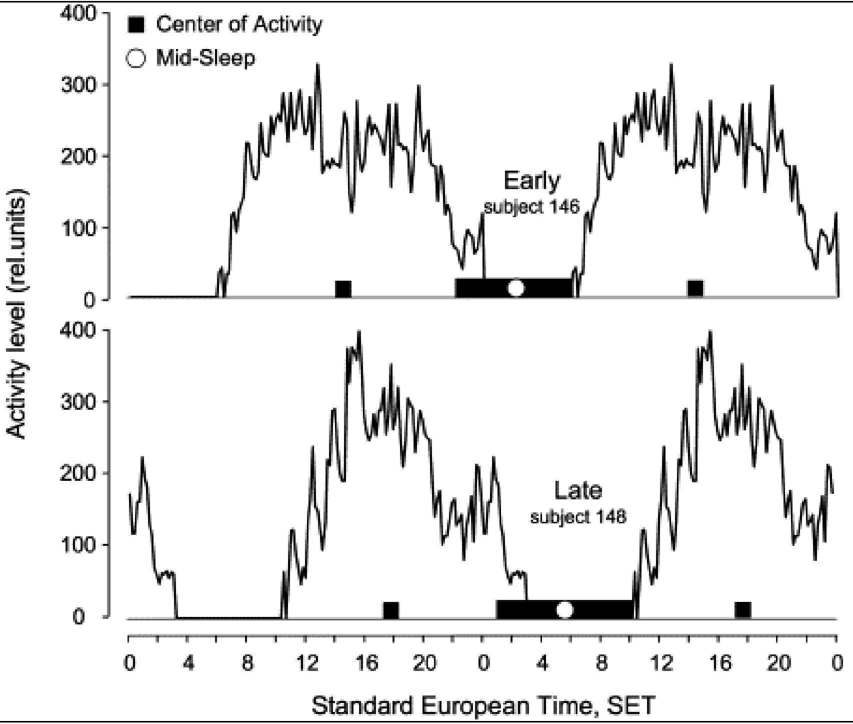

Fig. 3: Comparison of Sleep Times and Activity Profiles between early and late Chronotypes. (Kantermann et al., 2007, Curr Biol).

Revised text, page 35:

Experiment timing, sleep/wake cycle, and light variation

Timing of the experiments was determined based on the average of sleep times and level of activity of early and late chronotypes based on previous studies [4] as well as daily life routine schedules. For morning sessions, the start time was chosen based on the norm start of schooling and working (8:00 in the morning). This is also a time when both chronotypes have the lowest levels of activity [4]. The evening time (19:00) was specified based on the time when both chronotypes have a comparable level of activity according to previous dataset profiles [4]. Both groups were instructed to go to bed (around 22:00-23:00), and get up in the morning at identical times of the day, and have at least 8 hours of sleep for morning sessions ($mean_{ECs}$ = 8.41, $mean_{LCs}$ = 8.11). For the evening session, participants were allowed to go to bed at their preferred time but before 24:00 and were also allowed to have more than 8 hours of sleep if needed ($mean_{ECs}$ = 8.36, $mean_{LCs}$ = 8.38). All participants were strongly recommended to comply with the sleep/wake cycle instruction and in case of irregularities, poor quality of sleep, and sleepiness (measured by participants' rating before each session), the experimental session was postponed to another day. Subjective sleepiness of participants and their alertness were evaluated with the Karolinska sleepiness scale (KSS)[5] which measures sleepiness in a 1-10 Likert type scale (supplementary materials). No significant difference was observed between the sleepiness ratings of both early and late chronotypes in the morning sessions ($mean_{ECs}$ = 3.2, $mean_{LCs}$ = 3.71; t = 1.61, p = 0.11) and evening sessions ($mean_{ECs}$ = 3.58, $mean_{LCs}$ = 3.65; t = 0.25, p = 0.80).

2- The reference list should mention the original Horne and Ostberg publication for chronotype assessment. It'll be much clearer to the reader that may take the questionnaire as the Munich Chronotype Questionnaire.

RESPONSE: Respective reference is added [please see below].

Revised text, page 34:

The German version of the MEQ[27] (DMEQ)[10] was used to identify chronotypes.

3- The type of power mag stimulator needs to be specified

RESPONSE: Respective information is added [please see p. 42, highlighted in yellow]

Revised text, page 42:

Each cortical excitability session took 60-70 min. All TMS protocols were conducted with a PowerMag lab 30 magnetic stimulator (Mag & More, Munich, Germany) through a figure-of-eight magnetic coil (diameter of one winding, 70 mm; peak magnetic field, 2T) held 45° to the midline and applied over the left primary motor cortex

4- Ambient light levels during the experiments need to be specified as light may directly impact alertness and therefore potentially basic brain function.

RESPONSE: Respective information is added [please see below]

Revised text, page 38:

Furthermore, ambient light was kept constant (around 500 lux) across all experimental sessions in the morning and evening. In the cognitive sessions, tasks and EEG recordings were conducted in a sound-proof shielded room with constant temperature of 24-25°C for morning and evening sessions.

5- The percentage of hits included in the 3-back task has be provided. In addition, accuracy is not fully appropriate for n-back performance assessment. D-prime is more appropriate to take into account the unbalanced repartition of target and non-target items.

RESPONSE: Thank you for these suggestions. We added the percentage of hits for the 3-back task [please see p. 17 highlighted in yellow]. We also calculated D-prime and added the results to the main text [page 18, highlighted in yellow], Table 3 [please see p.66-67, highlighted in yellow] and updated Fig 5. accordingly. D prime follows the same pattern and shows an interaction with chronotype and time of day

Revised text, page 17-18:

For working memory performance, the ANOVA results revealed a significant chronotype×daytime interaction (F_1=11.70, p=0.002; ηp^2=0.28) for the N-back hits, which is the primary outcome of interest in this task (Table 2). Post *hoc* Student's t-tests showed a significantly enhanced WM performance in the morning for ECs and evening for LCs (Fig. 5a). The percentage of hits for ECs in the morning and evening was 65.39% and 57.15% respectively and for LCs in the evening and morning it was 72.71% and 62.65 respectively. The same response pattern was observed for the electrophysiological correlates of N-back task performance. Additionally, we calculated the sensitivity index *d* (or d prime) which represents the proportion of hit rates minus the proportion of false alarm rates. A significant interaction of chronotype×daytime (F_1=12.46, p<0.001; ηp^2=0.29) was found with no main effect of chronotype or daytime (Table 2). Post *hoc* Student's t-tests showed a significantly enhanced d prime index in the morning for ECs and evening for LCs (Fig. 5a).

6- Accuracy is not a standard outcome measure of the Stroop task but rather RT variability between congruent and incongruent trials. The author should focus on the latter.

RESPONSE: Thank you for the comment. Our primary focus was indeed on RT and we briefly mentioned accuracy. We emphasized more on this by adding RT variability as suggested. We analyzed this parameter across participants and results follow the same pattern (i,e. less variability at the preferred time in both groups). The results are added to the results section and Table 2 [please see page 18-19 highlighted in yellow].

Revised text, page 18-19:

For Stroop task performance, a similar trend of response was observed. We found a significant chronotype×daytime interaction (F_1=20.84, p=0.001; ηp^2=0.41) on overall RT, RT of congruent trials (F_1=14.36, p=0.001; ηp^2=0.32) and RT of incongruent trials (F_1=23.08, p=0.001; ηp^2=0.43). Congruency had a significant effect, but did not significantly interact with either daytime or chronotype. The main effects of daytime and chronotype were significant neither (Fig. 5). Post *hoc* comparisons of RTs revealed a significantly less Stroop effect (faster RT to incongruent trials) at the circadian-preferred time in both groups (Fig. 5d). This indicates less Stroop interference in participants when they were conducting the task at their circadian-preferred time. The same pattern of results was observed for RT variability in the Stroop block. The results of the ANOVA showed a significant interaction of chronotype×daytime (F_1=25.60, p<0.001; ηp^2=0.46) and post *hoc* comparisons revealed a significantly

reduced variability of RT in the Stroop block at the circadian-preferred time for both groups (ECs: t=2.81, p=0.013; LCs: t=4.62, p<0.001). Performance accuracy was not significantly affected by chronotype. The N200 and N450 are two prominent ERP markers related to Stroop conflict, and are observed at frontocentral and centroparietal regions[28, 29]. The less Stroop effect we observed at the circadian-preferred times was associated with higher N200 and N450 amplitudes which are indicative of higher selective attention and discriminating conflicting stimuli. The results of ANOVA for the N200 amplitudes showed a significant interaction of chronotype×daytime on overall (F_1=17.93, p=0.001; ηp^2=0.30), congruent (F_1=19.04, p<0.001; ηp^2=0.39) and incongruent (F_1=8.66, p=0.006; ηp^2=0.23) trials for the electrodes Fz and Cz. The N200 component of both congruent and incongruent trials over the Fz electrode was larger at the circadian preferred times in both groups (Fig. 5e,f), but the respective difference was significant only in ECs (incongruent: mean±$SEM_{morning}$, -0.38±0.31µV, mean±$SEM_{evening}$, 0.33±0.36µV; congruent: mean±$SEM_{morning}$, -0.59±0.29µV, mean±$SEM_{evening}$, 0.41±0.38µV). However, when we compared the amplitude difference values from morning to evening, chronotype had a significant effect (incongruent: F_1=7.57, p=0.010; ηp^2=0.21; congruent: F_1=14.49, p=0.001; ηp^2=0.33) yielding higher negativity of N200 at circadian-preferred times in both groups. The same pattern of response was found for the Cz electrode and the N450 component (Fig. S4a,b, Supplementary material).

1. Reis, J., et al., *Consensus: Can transcranial direct current stimulation and transcranial magnetic stimulation enhance motor learning and memory formation?* Brain Stimulation, 2008. **1**(4): p. 363-369.
2. Minarik, T., et al., *The Importance of Sample Size for Reproducibility of tDCS Effects.* Frontiers in Human Neuroscience, 2016. **10**(453).
3. Smith, M.J., et al., *Menstrual cycle effects on cortical excitability.* Neurology, 1999. **53**(9): p. 2069-2069.
4. Kantermann, T., et al., *The Human Circadian Clock's Seasonal Adjustment Is Disrupted by Daylight Saving Time.* Current Biology, 2007. **17**(22): p. 1996-2000.
5. Akerstedt, T. and M. Gillberg, *Subjective and objective sleepiness in the active individual.* Int J Neurosci, 1990. **52**(1-2): p. 29-37.
6. Huang, Y.-Z., et al., *Plasticity induced by non-invasive transcranial brain stimulation: A position paper.* Clinical Neurophysiology, 2017. **128**(11): p. 2318-2329.
7. Ridding, M. and U. Ziemann, *Determinants of the induction of cortical plasticity by non-invasive brain stimulation in healthy subjects.* The Journal of physiology, 2010. **588**(13): p. 2291-2304.
8. Rosenberg, J., et al., *"Early to bed, early to rise": Diffusion tensor imaging identifies chronotype-specificity.* NeuroImage, 2014. **84**: p. 428-434.
9. Ambrus, G.G., et al., *The fade-in – Short stimulation – Fade out approach to sham tDCS – Reliable at 1 mA for naïve and experienced subjects, but not investigators.* Brain Stimulation, 2012. **5**(4): p. 499-504.
10. Griefahn, B., et al., *Zur Validität der deutschen Übersetzung des Morningness-Eveningness-Questionnaires von Horne und Östberg.* Somnologie - Schlafforschung und Schlafmedizin, 2001. **5**(2): p. 71-80.
11. Jones, S.E., et al., *Genome-wide association analyses of chronotype in 697,828 individuals provides insights into circadian rhythms.* Nature Communications, 2019. **10**(1): p. 343.
12. Kuhn, M., et al., *Sleep recalibrates homeostatic and associative synaptic plasticity in the human cortex.* Nature Communications, 2016. **7**: p. 12455.
13. Stagg, C.J., A. Antal, and M.A. Nitsche, *Physiology of Transcranial Direct Current Stimulation.* The Journal of ECT, 2018. **34**(3): p. 144-152.
14. Kronberg, G., et al., *Direct current stimulation boosts hebbian plasticity in vitro.* Brain Stimulation, 2019.
15. Nitsche, M.A., et al., *Pharmacological Modulation of Cortical Excitability Shifts Induced by Transcranial Direct Current Stimulation in Humans.* The Journal of Physiology, 2003. **553**(1): p. 293-301.
16. Nitsche, M.A., et al., *Facilitation of Implicit Motor Learning by Weak Transcranial Direct Current Stimulation of the Primary Motor Cortex in the Human.* Journal of Cognitive Neuroscience, 2003. **15**(4): p. 619-626.
17. Rüsseler, J., et al., *Differences in incidental and intentional learning of sensorimotor sequences as revealed by event-related brain potentials.* Cognitive Brain Research, 2003. **15**(2): p. 116-126.

18. Rüsseler, J., T.F. Münte, and D. Wiswede, *On the influence of informational content and key-response effect mapping on implicit learning and error monitoring in the serial reaction time (SRT) task.* Experimental Brain Research, 2018. **236**(1): p. 259-273.
19. Borbély, A.A., et al., *The two-process model of sleep regulation: a reappraisal.* Journal of Sleep Research, 2016. **25**(2): p. 131-143.
20. Schmidt, C., et al., *A time to think: circadian rhythms in human cognition.* Cognitive neuropsychology, 2007. **24**(7): p. 755-789.
21. Horne, J.A. and O. Ostberg, *A self-assessment questionnaire to determine morningness-eveningness in human circadian rhythms.* Int J Chronobiol, 1976. **4**(2): p. 97-110.
22. Borbély, A.A., *A two process model of sleep regulation.* Hum neurobiol, 1982. **1**(3): p. 195-204.
23. Di Lazzaro, V., et al., *Muscarinic receptor blockade has differential effects on the excitability of intracortical circuits in the human motor cortex.* Experimental Brain Research, 2000. **135**(4): p. 455-461.
24. Chen, R., *Studies of human motor physiology with transcranial magnetic stimulation.* Muscle & Nerve, 2000. **23**(S9): p. S26-S32.
25. Di Lazzaro, V., et al., *Dissociated effects of diazepam and lorazepam on short-latency afferent inhibition.* The Journal of Physiology, 2005. **569**(1): p. 315-323.
26. Masri, S. and P. Sassone-Corsi, *The emerging link between cancer, metabolism, and circadian rhythms.* Nature Medicine, 2018. **24**(12): p. 1795-1803.
27. Horne, J.A. and O. Östberg, *A self-assessment questionnaire to determine morningness-eveningness in human circadian rhythms.* International Journal of Chronobiology, 1976. **4**: p. 97-110.
28. Hanslmayr, S., et al., *The Electrophysiological Dynamics of Interference during the Stroop Task.* Journal of Cognitive Neuroscience, 2008. **20**(2): p. 215-225.
29. Feroz, F.S., et al., *The Time Course of Activity within the Dorsal and Rostral-Ventral Anterior Cingulate Cortex in the Emotional Stroop Task.* Brain Topography, 2017. **30**(1): p. 30-45.

Appendix 1

Correlation between DMEQ score and neuroplasticity

Early chronotypes		DMEQ	SICI_inhibition_Mo	SICI_facilitation_Mo	SICI_inhibition_eve	SICI_facilitation_eve	SAI_Mo	SAI_eve	IWAVE_MO	IWAVE_EVE	IO_MO	IO_Eve
DMEQ	Pearson Correlation	1	.658**	.379	.259	.152	.101	.080	.204	-.021	.349	.436
	Sig. (2-tailed)		.006	.148	.332	.575	.711	.767	.449	.937	.186	.091
	N	16	16	16	16	16	16	16	16	16	16	16

Late chronotypes		DMEQ	SICI_inhibition_Mo	SICI_facilitation_Mo	SICI_inhibition_eve	SICI_facilitation_eve	SAI_Mo	SAI_eve	IWAVE_MO	IWAVE_EVE	IO_MO	IO_Eve
DMEQ	Pearson Correlation	1	-.514*	.204	-.313	.348	-.448	-.533*	-.130	-.127	.029	-.108
	Sig. (2-tailed)		.042	.449	.237	.187	.082	.033	.632	.639	.916	.691
	N	16	16	16	16	16	16	16	16	16	16	16

Correlation between DMEQ score and neuroplasticity

Early chronotypes		DMEQ1	anodal_mo	cathodal_mo	anodal_eve	cathodal_eve	anodal_mo_0_20	cathodal_mo_0_20	anodal_eve_0_20	cathodal_eve_0_20
DMEQ	Pearson Correlation	1	.037	.446	.094	-.042	.031	.491	-.020	-.018
	Sig. (2-tailed)		.891	.084	.730	.879	.909	.053	.941	.946
	N	16	16	16	16	16	16	16	16	16

Late chronotypes		DMEQ1	anodal_mo	cathodal_mo	anodal_eve	cathodal_eve	anodal_mo_0_20	cathodal_mo_0_20	anodal_eve_0_20	cathodal_eve_0_20
DMEQ1	Pearson Correlation	1	-.276	.116	-.322	.144	-.292	.144	-.357	.132
	Sig. (2-tailed)		.300	.670	.224	.595	.272	.594	.175	.625
	N	16	16	16	16	16	16	16	16	16

Correlation between DMEQ score and behavioral performance

Early chronotypes		DMEQ	nback_hit_mo	nback_hit_eve	nback_RT_mo	nback_RT_eve	CW_Acc_MO	RT_MOstroop	CW_Acc_Eve	RT_Eve_stroop	AXCPT_ACC_Mo	AXCPT_RT_Mo	AXCPT_ACC_eve	AXCPT_RT_eve
DMEQ	Pearson Correlation	1	.092	.161	.573*	.239	-.378	-.076	-.541*	-.330	-.020	.048	-.388	.084
	Sig. (2-tailed)		.734	.552	.020	.373	.149	.779	.031	.213	.940	.861	.137	.756
	N	16	16	16	16	16	16	16	16	16	16	16	16	16

Late chronotypes		DMEQ	nback_hit_mo	nback_hit_eve	nback_RT_mo	nback_RT_eve	CW_Acc_MO	RT_MO_stroop_	CW_Acc_Eve	RT_Eve_stroop_	AXCPT_ACC_MO	AXCPT_RT_MO	AXCPT_ACC_eve	AXCPT_RT_eve
DMEQ	Pearson Correlation	1	.029	.167	.409	.376	.464	-.171	.586*	-.094	-.264	.178	.270	-.024
	Sig. (2-tailed)		.915	.536	.116	.151	.070	.527	.017	.731	.324	.510	.311	.930
	N	16	16	16	16	16	16	16	16	16	16	16	16	16

Appendix 2

Correlation between demographics and neuroplasticity

Early chronotypes		anodal_mo	cathodal_mo	anodal_mo_0_20	cathodal_mo_0_20	anodal_eve	cathodal_eve	anodal_eve_0_20	cathodal_eve_0_20
Age	Pearson Correlation	-.180	-.073	-.249	-.100	.185	-.054	.145	-.032
	Sig. (2-tailed)	.505	.788	.352	.713	.492	.844	.591	.908
	N	16	16	16	16	16	16	16	16
Gender	Pearson Correlation	.065	-.162	.091	-.062	-.262	-.116	-.252	-.060
	Sig. (2-tailed)	.812	.548	.737	.819	.327	.669	.346	.824
	N	16	16	16	16	16	16	16	16
BMI	Pearson Correlation	-.027	.414	.002	.292	.321	-.448	.304	-.469
	Sig. (2-tailed)	.920	.111	.994	.272	.225	.082	.253	.067
	N	16	16	16	16	16	16	16	16

Late chronotypes		anodal_mo	cathodal_mo	anodal_mo_0_20	cathodal_mo_0_20	anodal_eve	cathodal_eve	anodal_eve_0_20	cathodal_eve_0_20
Age	Pearson Correlation	.095	.294	.122	.198	.110	-.245	.114	-.217
	Sig. (2-tailed)	.726	.268	.653	.463	.686	.360	.673	.421
	N	16	16	16	16	16	16	16	16
Gender	Pearson Correlation	.137	.104	.082	.093	.230	.046	.252	-.012
	Sig. (2-tailed)	.613	.700	.762	.733	.391	.865	.347	.966
	N	16	16	16	16	16	16	16	16
BMI	Pearson Correlation	.236	.268	.240	.154	.412	-.035	.364	.011
	Sig. (2-tailed)	.379	.316	.371	.569	.113	.896	.165	.968
	N	16	16	16	16	16	16	16	16

Correlation between demographics and cortical excitability

Early chronotypes		SICI_inhibition_Mo	SICI_facilitation_Mo	SICI_inhibition_eve	SICI_facilitation_eve	SAI_Mo	SAI_eve	IWAVE_MO	IWAVE_EVE	IO_MO	IO_Eve
Age	Pearson Correlation	-.145	.070	-.177	.190	.247	.032	.009	-.258	-.125	-.026
	Sig. (2-tailed)	.592	.797	.513	.482	.357	.905	.974	.335	.645	.925
	N	16	16	16	16	16	16	16	16	16	16
Gender	Pearson Correlation	-.018	.153	-.025	-.150	-.302	-.070	.113	-.185	.111	.193
	Sig. (2-tailed)	.947	.571	.926	.580	.255	.796	.676	.492	.681	.474
	N	16	16	16	16	16	16	16	16	16	16
BMI	Pearson Correlation	.364	-.212	.111	-.025	.263	-.215	.402	-.211	.420	.186
	Sig. (2-tailed)	.166	.430	.681	.926	.325	.425	.122	.434	.106	.491
	N	16	16	16	16	16	16	16	16	16	16

Late chronotypes		SICI_inhibition_Mo	SICI_facilitation_Mo	SICI_inhibition_eve	SICI_facilitation_eve	SAI_Mo	SAI_eve	IWAVE_MO	IWAVE_EVE	IO_MO	IO_Eve
Age	Pearson Correlation	-.216	.104	-.138	.370	-.242	.058	-.021	.269	-.323	-.158
	Sig. (2-tailed)	.422	.703	.611	.158	.366	.832	.939	.314	.223	.559
	N	16	16	16	16	16	16	16	16	16	16
Gender	Pearson Correlation	-.017	-.360	.085	-.340	.461	.409	-.331	-.414	-.280	-.122
	Sig. (2-tailed)	.950	.171	.753	.198	.072	.116	.211	.110	.293	.652
	N	16	16	16	16	16	16	16	16	16	16
BMI	Pearson Correlation	-.110	-.058	-.254	-.068	.117	.394	-.385	.014	-.392	.132
	Sig. (2-tailed)	.684	.830	.343	.802	.667	.131	.141	.960	.133	.625
	N	16	16	16	16	16	16	16	16	16	16

Correlation between demographics and cognitive performance

Early chronotypes		nback_hit_mo	nback_hit_eve	nback_RT_mo	nback_RT_eve	CW_RT_MO_stroop_effect	CW_RT_Eve_stroop_effect	AXCPT_ACC_MO	AXCPT_RT_MO	AXCPT_ACC_eve	AXCPT_RT_eve
Age	Pearson Correlation	-.497	-.395	.459	.141	.445	.291	-.281	.375	.392	-.488
	Sig. (2-tailed)	.050	.130	.074	.602	.084	.275	.291	.152	.133	.055
	N	16	16	16	16	16	16	16	16	16	16
Gender	Pearson Correlation	.361	.147	-.703**	-.353	-.377	-.090	-.183	-.117	-.232	-.017
	Sig. (2-tailed)	.169	.586	.002	.180	.149	.740	.497	.665	.388	.949
	N	16	16	16	16	16	16	16	16	16	16
BMI	Pearson Correlation	-.534*	-.269	.376	.153	.413	.373	.248	.146	.059	-.209
	Sig. (2-tailed)	.033	.314	.151	.571	.112	.155	.355	.590	.827	.438
	N	16	16	16	16	16	16	16	16	16	16

Late chronotypes		nback_hit_mo	nback_hit_eve	nback_RT_mo	nback_RT_eve	CW_RT_MO_stroop_effect	CW_RT_Eve_stroop_effect	AXCPT_ACC_MO	AXCPT_RT_MO	AXCPT_ACC_eve	AXCPT_RT_eve
Age	Pearson Correlation	.040	.187	-.129	-.010	.002	.077	.051	-.191	-.123	-.079
	Sig. (2-tailed)	.883	.487	.635	.970	.994	.778	.851	.479	.651	.771
	N	16	16	16	16	16	16	16	16	16	16
Gender	Pearson Correlation	-.224	-.292	-.093	.170	-.171	.058	-.151	-.129	-.072	-.243
	Sig. (2-tailed)	.404	.273	.732	.530	.526	.830	.578	.635	.790	.365
	N	16	16	16	16	16	16	16	16	16	16
BMI	Pearson Correlation	-.222	-.117	-.066	-.010	-.016	-.025	-.010	-.222	-.368	-.257
	Sig. (2-tailed)	.409	.666	.808	.971	.954	.927	.971	.409	.160	.336
	N	16	16	16	16	16	16	16	16	16	16

Reviewers' comments:

Reviewer #1 (Remarks to the Author):

The authors have addressed all my comments satisfactorily and the revised manuscript reads very well.

One minor thing - which I will not insist on - is that "daytime" in common usage tends to refer to any point during the day (as opposed to night-time) whereas "time of day" refers to a specific time in the 24 hour clock. They are both entirely grammatically correct but do not quite mean the same thing. I think the authors tend to mean the latter rather than the former, but this is up to them and to the copy editor.

I have no further concerns

Reviewer #2 (Remarks to the Author):

This is a very nice bit of work. It is original -- looking at chronotypes -- and does so across multiple levels of the biological hierarchy. My earlier comments have been carefully and thoughtfully addressed. The study itself and the subsequent review must have taken a huge amount of work.

Reviewer #3 (Remarks to the Author):

While I appreciate the efforts made by the authors to respond to my comments, my concerns about the findings remain the same.
1. Requesting that volunteers had at least 8h of sleep without an objective measure of compliance with actimetry (or potentially time-stamped voice mail call) basically means that there is no mean to assess compliance, which is a major issue in human research, particularly for sleep-wake schedule in chronobiology research.
2. Even if volunteer complied to the schedule, if they are truly differing in their chronotype (alse see 4), the imposed sleep-wake will inevitably be inappropriate for most of them, and particularly for late chronotypes which typically have difficulties sleeping early. This leads to a typical social jet-lag in late chronotypes (to which the authors do not refer to) which means that during week days, when constrained in the sleep-wake schedule late chronotypes build up a sleep debt that they “pay-off” over the week-end by sleeping-in in the morning.
3. The absence of subjective sleepiness difference give no support to the fact that volunteers slept an equal requested amount. Subjective scale are particularly useful for relative changes over repeated measures but not for comparisons of absolute values. In other words, one can adapt her/his judgement according to her/his habits. Late chronotypes are used to sleepiness in the morning as they are constrained to wake up earlier than what they want, and may adapt their judgement to their habit. This does not mean that they slept enough and do not undergo chronic sleep loss.
4. This means that at best, the authors show differences between two groups of volunteers, with one well-rested and chronically sleep-deprived, or one undergoing less sleep-wake constrain and the other highly constrained in their schedule. If, as claimed by the authors the two groups do not differ in their sleep duration, sleep quality and distance between preferred sleep timing and imposed sleep timing, then they are not studying groups with different chronotypes (potentially because they excuded extreme chronotypes – a point they omitted to mention in their original submission).
Again, overall this completely changes the scope of the paper, and its interest in my view, as it cover something much more trivial. I have no doubt about the quality of the data collected but rather about the interpretations of the finding based on a wrongly conceived protocol.
I disagree with the authors when they claim that all types of session timing selections would be suboptimal. As many other have done (e.g. Mongrain et al. 2005 Sleep, Schmidt et al. 2009 Science), one can ask preferred sleep-wake timing or even monitor it with unrestricted actimetry to set the

timing of the protocol. This is indeed difficult and complicated to do in practice, but is the only way to assess true chronotype differences.
The new data set the authors refer does not resolve the issue. The interaction between sleep homeostasis and the circadian system is in essence non-linear so that chronic sleep loss during the biological day cannot be inferred from total sleep deprivation.
Minor comments
500 lux light level is very high chronobiologically speaking. Given the different response to light according to chronotype (e.g. Wright et al. 2013), this additional protocol misconception may have induced further bias.
Not having a significant difference between groups with respect to covariate does not imply that there is difference at all and that the covariate is not playing some role in the association of interest. The statistical model need to include therefore the most important demographic characteristics such as sex, age and BMI.

Authors' Response to the Review Comments

Journal: Nature Communications

Manuscript #: NCOMMS-20-13170-T

Title of Paper: Cognitive functions and respective parameters of human brain physiology are chronotype-dependent

Authors: MA Salehinejad, M Wischnewski, E Ghanavati, M Mosayebi-Samani, M-F Kuo, and MA Nitsche

Reviewers' comments:

Reviewer #1 (Remarks to the Author):

This study was performed to examine the effects of chronotype on motor cortical physiology, plasticity and behaviour. The authors demonstrated an interaction between chronotype and time of day for motor cortical excitability and paired-pulse TMS measures of excitation and inhibition, which taken together suggest that subjects had decreased excitability / increased inhibition at their non-preferred time of day. The time of day also had a significant effect on plasticity induction with tDCS, with the largest effects being seen at the preferred time of day. Finally, chronotype had significant effects on both motor learning and working memory tasks.

This is an impressive study involving a substantial degree of data collection to answer an interesting question in several different ways. It adds substantially to the literature by extending our understanding of the neurophysiological underpinnings of chronotypic differences. I have a few comments and suggestions for the authors, which I hope will aid the manuscript.

RESPONSE: We appreciate the reviewer's time and effort in reading our work and the positive evaluation s/he made. The comments are insightful and improved the quality of our manuscript. In what follows, we provide a one-by-one response to each comment and revised the main text accordingly. For the convenience of the reviewer, we pasted the revised version of the text here as well highlighted in blue.

Major Points

1. I take the authors point, but I am not sure that the link between stimulation-induced neuroplasticity and motor learning is as well as established as they suggest (line 110). I think that this is a stretch and should be rephrased.

RESPONSE: We agree with the reviewers' point and rephrased the sentence [please see p. 5, highlighted in blue].

REVISED text, P. 5:

In this study, we first systematically investigated the impact of chronotype and time-of-day effects on *cortical excitability* and stimulation-induced *neuroplasticity* in the model of the human motor cortex. In the next step, we explored how chronotype affects performance on a *motor learning* task which is associated with motor cortical plasticity[1].

2. Given the variability inherent in tDCS protocols, how was the study powered? I think more details would be helpful here.

RESPONSE: In the methods participant section (page 35), we now describe in detail the power analysis and how we determined sample size based on previous tDCS work (e.g., Minarik et al., 2016) [please see p. 33, highlighted in blue].

REVISED text, P.35:

Sample size was calculated a-priori based on a power analyses which showed that for a medium effect size (partial eta squared=0.10) (suggested for NIBS studies[2]), a minimum of 24 subjects is required to achieve 95% power at an alpha of 0.05 for the primary statistical test of a mixed model ANOVA design. We increased the sample size to 32 to fully counterbalance task order in each group (N=16) and compensate for unforeseen variability and dropouts.

3. Given the importance of circadian rhythms, did the authors ensure that they kept records of sleep/wake cycles, caffeine intake, (nicotine intake?) other things that might affect a person's circadian rhythm? Was sleepiness or equivalent taken into account?

RESPONSE: Thank you for the comment. Yes, we controlled for sleep duration, sleep/wake cycle, caffeine intake, nicotine intake, and other potentially interfering factors. All participants were non-smokers [please see page 35-36 highlighted in blue]. Regarding the sleep/wake cycle and sleepiness, we added a new section in the methods explaining how we controlled for these factors [please see page 37-38, highlighted in blue and green].

REVISED text, P.35-36:

All participants were right-handed non-smokers and underwent a medical screening to verify no history of neurological diseases, epilepsy or seizures, medication, metal implants, and current pregnancy. Each participant took part in a test TMS session to become acquainted with experiencing stimulation and understanding the study protocol. Female subjects were not examined during the luteal phase of the menstrual cycle (around week 3 following menstruation) to ensure that hormonal changes that affect cortical excitability [3] would not interfere with their chronotype. Caffeine intake and other factors potentially interfering with cortical excitability (e.g., massive physical activity) were controlled before each experimental session and in case the respective criteria were not met, these sessions were postponed.

REVISED text, P.37-38:

Experiment timing, sleep/wake cycle, and light variation

Timing of the experiments was determined based on the average of sleep times and level of activity of early and late chronotypes based on previous studies [4] as well as daily life routine schedules. For morning sessions, the start time was chosen based on the norm start of schooling and working (8:00 in the morning). This is also a time when both chronotypes have the lowest levels of activity [4]. The evening time (19:00) was specified based on the time when both chronotypes have a comparable level of activity according to previous dataset profiles [4]. Both groups were instructed to go to bed (at around 22:00-23:00), and get up in the morning at identical times of the day, and have at least 8 hours of sleep for morning sessions ($mean_{ECs}$ = 8.41, $mean_{LCs}$ = 8.11). For the evening session, participants were allowed to go to bed at their preferred time but before 24:00 and were also allowed to have more than 8 hours of sleep if needed ($mean_{ECs}$ = 8.36, $mean_{LCs}$ = 8.38). All participants were strongly recommended to comply with the sleep/wake cycle instruction and in case of irregularities, poor quality of sleep, and sleepiness (measured by participants' rating before each session), the experimental session was postponed to another day. Subjective sleepiness of participants and their alertness were evaluated with the Karolinska sleepiness scale (KSS)[5] which measures sleepiness in a 1-10 Likert-type scale (supplementary materials). No significant difference was observed between the sleepiness ratings of both early and late chronotypes in the morning sessions ($mean_{ECs}$ = 3.2, $mean_{LCs}$ = 3.71; t = 1.61, p = 0.11) and evening sessions ($mean_{ECs}$ = 3.58, $mean_{LCs}$ = 3.65; t = 0.25, p = 0.80). Due to the variability of seasonal light, experimental sessions did not take place in summer when the difference between sunrise and sunset is largest in Germany. For other months, the experiment was scheduled to be randomly distributed across other seasons in both groups. Furthermore, ambient light was kept constant (around 500 lux) across all experimental sessions in the morning and evening. In the cognitive sessions, tasks and EEG recordings were conducted in a sound-proof shielded room with constant temperature of 24-25°C for morning and evening sessions.

4. Given the previously reported effects of several factors, including a number of genetic variants (e.g BDNF phenotype) the results presented in Figure 3 look remarkably homogenous. Could the authors comment on why this might be, compared with similar data (answering different questions) from other labs?

RESPONSE: Thank you for this comment. Our tDCS results are indeed homogeneous at the group level, this however does not exclude interindividual variability [please see below scatterplots of individual absolute data]. We think that stimulation at the optimal time, with sufficient sleep, and control of interfering factors are potential reasons for the relatively homogenous pattern of response in both preferred and non-preferred times. These data suggest that if tDCS is applied in a carefully controlled situation and with optimal brain states, then we can expect to have homogenous results. We discuss this also in the discussion [p. 25, highlighted in green and blue] as an implication for the variability of results in NIBS research.

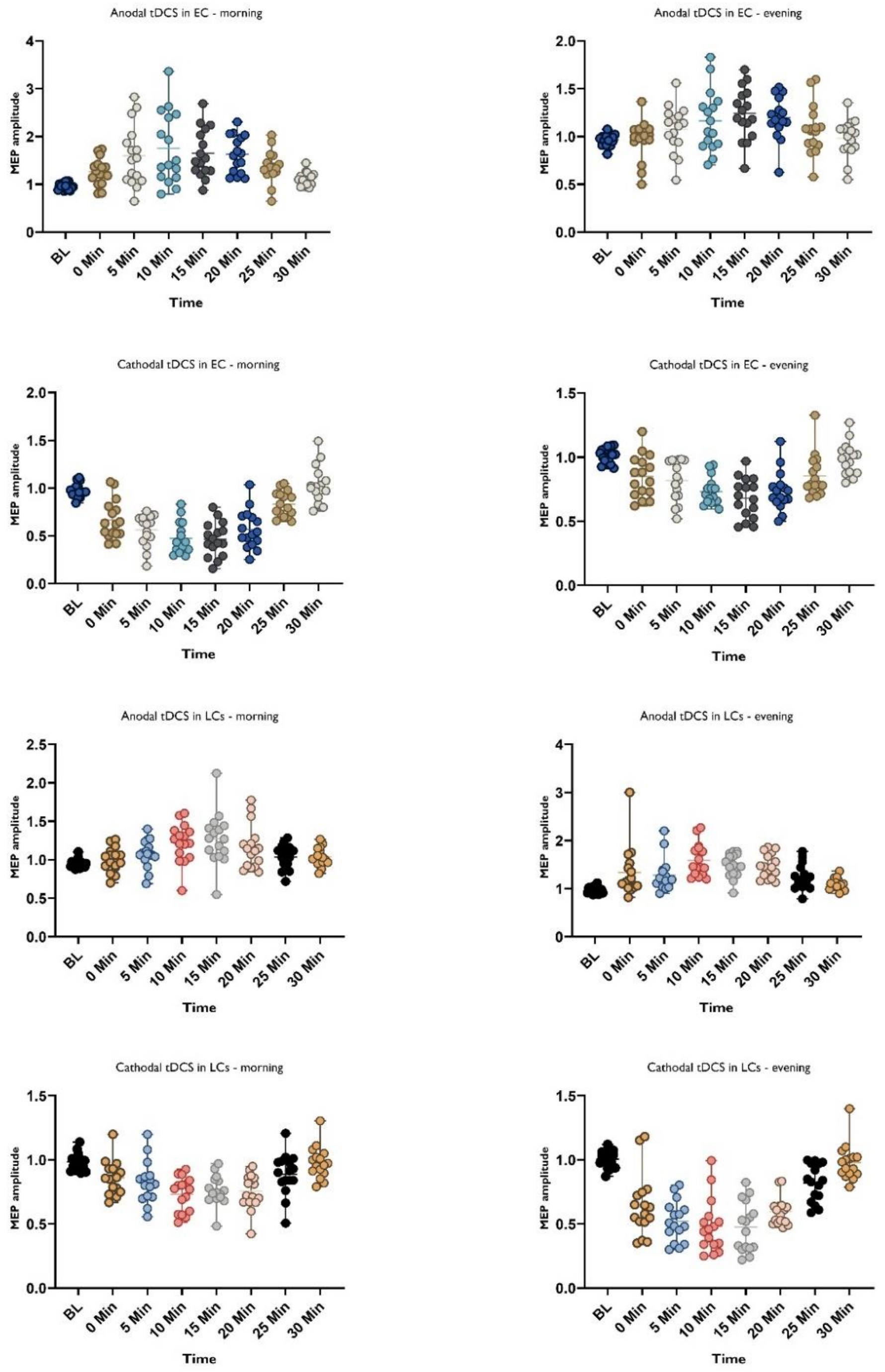

REVISED text, P.25:
The findings of this study have specific implications for the field of human neurophysiology and cognitive neuroscience as well as broad implications for human behavior in healthy and clinical populations. "Time-of-day" and chronotype are important, but rarely studied determinants of cortical plasticity induction by NIBS techniques[6, 7]. Our results show that cortical excitability and neuroplasticity are chronotype-dependent in humans. It is tempting to speculate that chronotype could account for variability in the efficacy of NIBS, and might explain partially heterogeneous effects in previous studies. This assumption might be likewise relevant for the performance of various cognitive tasks and suggest screening or considering its modulatory effects. Being studied at the optimal time of day, having sufficient sleep and control of interfering factors might enhance homogeneity of results, as our data suggest.

5. There is some evidence that chronotype affects other brain metrics that might then affect those demonstrated here, for example, DTI and grey matter volumes (e.g. Rosenberg et al., Neuroimage 2014; Rosenberg et al., BS&F 2018). How would more/less grey matter interact with the effects of tDCS, for example? The current model shown in figure 3 is for a standard brain, but the current density (and hence, probably, the effectiveness) must depend on the individual anatomy, which may be affected by chronotype. I understand that the authors will not have the data to answer this directly, but a discussion would be useful.

RESPONSE: Thank you for the comment. We agree with the reviewer's point. The purpose of the 3D-modeling of the current flow was simply to show to the broad readership electrical fields induced by the intervention. We thus did not discuss the modeling results in the main text but just mentioned it in the legend of Fig. 3. Nevertheless, as suggested, we mention the point of chronotype-dependent brain anatomy differences, and their possible impact on stimulation effects now in the discussion [please see p. 26-27, highlighted in blue]. We also added a clarification to the methods section to avoid misconceptions about the meaning of the modelling [please see methods page 43, highlighted in blue].

REVISED text, P.26-27:
Our physiological measures were based on the motor system and indirect measures of the involved neurotransmitters, yet they suggest systematic effects of chronotype at the whole-brain level. Interestingly, chronotype-specific individual differences in brain anatomy (e.g. grey matter volume) have been described by some studies[8], which could affect tDCS-induced neuroplasticity induction either in a facilitatory or inhibitory way. It is unlikely that differences of brain structure were the driving force of the results because both groups showed enhanced plasticity at specific times of the day congruent with their respective chronotype. It was beyond the scope of our study to investigate chronotype-specific differences of brain structure, if any. This, however, would be an interesting question for future neuroimaging studies to add additional evidence to the findings of current study.

REVISED text, P.43:
Based on the randomized condition, anodal, cathodal, or sham tDCS with 1 mA intensity were applied for 7 min with 15 s ramp up/down at the beginning and end of stimulation. For the sham condition, stimulation was delivered for 30 s, with a 30 s ramp up and down. Using this procedure, participants are not able to distinguish between real and sham tDCS[9]. We simulated electrical current flow in the head induced by this protocol to show how the primary motor cortex is affected by tDCS (Fig. 3a). The model is based on the standard head model which does not take into account potential structural differences (e.g. white matter) in the brains of different chronotypes [8]. The model should thus be taken into account for illustrative purposes only.

6. The authors have numerical data on the DMEQ which presumably could be used to quantify the degree of "evening preference" or morning preference. Are there correlations between the major findings (particularly the physiology and the behaviour) and this DMEQ score? While I understand the study was not powered to examine this, I think it might be of interest, and would strengthen the arguments if such a relationship was present.

RESPONSE: Thank you for making this point. We had to limit our words to meet the journal guidelines which is why not all data are presented. In the methods, however we mentioned that both groups were moderate types. We now added the numerical data too [please see page 36-37, highlighted in blue]. We calculated the respective correlations for each chronotype separately as suggested. There was no significant correlation between DMEQ scores and mean MEP amplitudes obtained from each stimulation condition. Similarly, no significant correlations were found between DMEQ scores and cortical excitability, except for cortical inhibition obtained by the SICI protocol. The correlations between task performance and DMEQ scores were neither significant, except for Stroop accuracy. Given that the study was not powered to examine this (as the reviewer put it) and that this outcome measure was not significantly different across groups in the morning and evening, we would prefer not to add these results to the manuscript. We added these data however as **appendix 1** at the end of the response letter [please see end of this letter].

REVISED text P.36-37:
The questionnaire shows high reported reliability and has a significant correlation with circadian rhythm-related hormonal changes, including melatonin[10]. In addition to the MEQ, we defined the dichotomous chronotype phenotype by asking two identical questions ("Are you naturally a night person or a morning person?")[11]. Individuals with discordant or neutral responses to both were excluded. The mean scores of chronotype for the early and late chronotypes were 62.25±3.47 and 35.37±4.31, showing that both groups represent moderate early and late chronotypes.

REVISED text, supplementary materials:

1.1. Correlation between chronotype score, physiological parameters, and behavior

We also explored potential correlations between the participants' scores on DMEQ (i.e., circadian preference) and major outcome measures for both groups separately. No significant correlations were observed between DMEQ scores and cortical excitability except for intracortical inhibition (measured by SICI) of ECs obtained in the morning, which was positively correlated with DMEQ scores (r= 0.658, p=0.006). No significant correlations were neither found between DMEQ scores and neuroplasticity, or major outcome measures of the behavioral tasks.

Minor points.

1. The title probably needs rephrasing – cognitive function and their physiological parameters are chronotype-dependent? The grammar doesn't quite work at the moment, though I have no issue with the content.
RESPONSE: Thank you for your suggestion. The title is indeed "*Cognitive functions and **respective** parameters of human brain physiology are chronotype-dependent*".

2. A minor point, but daytime would probably better be "time of day".
RESPONSE: Thank you for your suggestion. As far as we can see, both words are grammatically correct and we used both in the manuscript. In the results, we used the term "daytime" as a factor in the analyses. However, as "time of day" has more stress on time, which was an important variable in our study, we thought this would convey our points/argument better. But we would be willing to change terms if this reviewer insists.

3. While taking the IO slope as the dependent variable would reduce effects of differences in rMT – were there differences between the sessions in the rMT – both in terms of the amplitude of the MEPs evoked, and more interestingly, the % of stimulator output used to evoke them?
RESPONSE: Thank you very much for the comment. This point is important, and we have described in detail the threshold values of all TMS protocols, including MEP size at RMT intensity (for the I-O curve

protocol), %MSO for RMT and SI_{1mv} in the supplementary material [Please see section 1.1 and Tables S1, S2 in the supplementary materials highlighted in blue].

Revised text, supplementary material.

1.1. TMS protocols threshold values

MEP values of single-pulse conditions and other control conditions, as well as subject-specific baseline sensitivity to TMS (defined as the percentage of maximum stimulator output required for the SI1mV MEP amplitude), are summarized in Table S1. For each TMS protocol, we compared single-pulse condition MEPs (control condition) and the SI1mV obtained at different daytimes by a 2-factorial ANOVA with daytime (morning, evening) as the within-subject and chronotype (ECs, LCs) as the between-subject factor. The results of the respective ANOVAs show that the mean values of the single-pulse conditions MEP, MEP amplitude at RMT (for the I-O curve protocol), %MSO for RMT, and SI_{1mv} did not significantly differ between time of day and groups (Table S2). Only for AMT, the interaction of chronotype×daytime was significant, and post hoc t-tests revealed a significant difference between AMT values only for ECs across daytime (t = 3.58, p = 0.003).

Table S1: Baseline measurements of the cortical excitability protocols

**Protocol**	**measurement**	**daytime**	**Group**	
			**ECs**	**LCs**
Single-pulse MEP	SI 1_{mV} (%)	morning	51.31 ± 9.82	50.43 ± 8.46
		evening	50.50 ± 10.19	50.87 ± 7.91
RMT	%MSO	morning	42.87 ± 7.54	41.81 ± 6.88
		evening	41.68 ± 7.15	42.12 ± 7.09
AMT	%MSO	morning	35.18 ± 6.95	34.40 ± 8.23
		evening	33.53 ± 6.97	34.96 ± 8.18
I-O curve	MEP amplitude at RMT	morning	0.235 ± 0.213	0.197 ± 0.204
		evening	0.149 ± 0.091	0.200 ± 0.133
SICI-ICF	Single-pulse MEP	morning	1.073 ± 0.335	0.941 ± 0.152
		evening	0.910 ± 0.342	0.980 ± 0.121
I-wave facilitation	Single-pulse MEP	morning	1.020 ± 0.341	1.041± 0.119
		evening	1.237 ± 0.512	1.178 ± 0.438
SAI	Single-pulse MEP	morning	1.237 ± 0.512	1.178 ± 0.438
		evening	1.194 ± 0.269	1.195 ± 0.175

Note: MEP = motor-evoked potential; ECs = early chronotypes; LCs = late chronotypes; SI_{1mv} (%) = maximum stimulator output (%MSO) required for the SI1mV MEP amplitude; I-O curve = input-output curve; SICI-ICF = short latency intracortical inhibition and facilitation; SAI = short-latency afferent inhibition; RMT = resting motor threshold; AMT = Active motor threshold.

Table S2: Results of 2 × 2 ANOVAs for control MEPs (RMT intensity MEP for I-O curve, single-pulse MEP for other protocols), SI1mV, and %MSO for RMT/AMT.

**Protocol**	**Measurement**	**Factor**	***df***	***F***	***p***
Single-pulse MEP	SI_{1mV}	daytime	1	0.12	0.724
		chronotype	1	1.40	0.244
		daytime×chronotype	1	0.001	0.938
RMT	%MSO	daytime	1	0.86	0.359
		chronotype	1	0.01	0.901
		daytime×chronotype	1	2.54	0.120
AMT	%MSO	daytime	1	3.23	0.082
		chronotype	1	0.015	0.903
		daytime×chronotype	1	13.30	<**0.001**

I-O curve	MEP amplitude at RMT	daytime	1	1.04	0.315
		chronotype	1	0.02	0.882
		daytime×chronotype	1	1.169	0.288
SICI-ICF	Single-pulse MEP	daytime	1	0.98	0.329
		chronotype	1	0.22	0.641
		daytime×chronotype	1	2.65	0.113
I-wave facilitation	Single-pulse MEP	daytime	1	0.23	0.631
		chronotype	1	0.83	0.367
		daytime×chronotype	1	0.37	0.547
SAI	Single-pulse MEP	daytime	1	0.017	0.895
		chronotype	1	0.112	0.739
		daytime×chronotype	1	0.090	0.765

Note: MEP = motor-evoked potential; chronotype = early (ECs) and late (LCs) chronotypes; SI_{1mv} (%) = maximum stimulator output (%MSO) required for the SI1mV MEP amplitude; I-O curve = input-output curve; SICI-ICF = short latency intracortical inhibition and facilitation; SAI = short-latency afferent inhibition; RMT = resting motor threshold; AMT = active motor threshold.

4. I appreciate this group's transparency with their statistics, and the ANOVA tables are helpful with such complex approaches. Please could these be added somewhere for the other results too? Supplementary tables would be fine, but it is helpful to see them all for clarity.

RESPONSE: Thank you for this suggestion. We have space for another Table according to journal guidelines and added statistics for the cognition and EEG part as suggested (please see below and on page 66, highlighted in blue). For neuroplasticity results, we mentioned all statistics either in the text [page 11, highlighted in blue] or the figure legend [page 31, highlighted in blue], unfortunately, an additional table would exceed the limitations of the journal.

Table S2: Results of ANOVAs for cognitive task performance and respective ERP components.

**Task**	**Measurement**	**Factor**	***df***	***F***	***p***	**ηp^2**
SRTT (motor learning)	BL 5 vs 6 RT	learning blocks	1	70.31	**<0.001**	0.708
		chronotype	1	0.41	0.527	0.014
		daytime	1	0.015	0.902	0.001
		learning blocks×chronotype	1	4.07	0.053	0.123
		learning blocks×daytime	1	0.77	0.399	0.025
		learning blocks×chronotype×daytime	1	22.91	**<0.001**	0.441
	BL 7 vs 6 RT	learning blocks	1	72.18	**<0.001**	0.731
		chronotype	1	0.11	0.733	0.004
		daytime	1	0.35	0.852	0.001
		learning blocks×chronotype	1	1.81	0.188	0.059
		learning blocks×daytime	1	0.50	0.824	0.002
		learning blocks×chronotype×daytime	1	10.76	**0.003**	0.271
	P300	learning blocks (BL5-7)	1	29.20	**<0.001**	0.502
		chronotype	1	3.29	0.080	0.102
		daytime	1	0.62	0.438	0.21
		learning blocks×chronotype	1	0.33	0.716	0.011
		learning blocks×daytime	1	0.97	0.384	0.032
		learning blocks×chronotype×daytime	1	8.11	**<0.001**	0.219
3-back (working memory)	Accuracy	chronotype	1	0.881	0.365	0.029
		daytime	1	0.117	0.735	0.004
		daytime×chronotype	1	11.70	**0.002**	0.281

	d prime	chronotype	1	0.60	0.444	0.020
		daytime	1	0.22	0.642	0.007
		daytime×chronotype	1	12.46	**<0.001**	0.293
	RT	chronotype	1	0.01	0.978	0.001
		daytime	1	0.70	0.407	0.02
		daytime×chronotype	1	1.08	0.305	0.035
	P300-Pz	chronotype	1	1.13	0.295	0.037
		daytime	1	0.01	0.927	0.001
		daytime×chronotype	1	14.49	**<0.001**	0.326
	P300-Cz	chronotype	1	0.28	0.600	0.009
		daytime	1	2.05	0.162	0.064
		daytime×chronotype	1	11.19	**0.002**	0.272
Stroop (selective attention)	Stroop block RT	chronotype	1	0.15	0.696	0.005
		daytime	1	0.48	0.490	0.016
		congruency	1	75.34	**<0.001**	0.715
		daytime×chronotype	1	20.84	**<0.001**	0.410
		congruency×chronotype	1	1.02	0.320	0.033
		congruency×daytime	1	1.94	0.174	0.061
		congruency×chronotype daytime	1	0.85	0.362	0.028
	Congruent trials RT	chronotype	1	0.72	0.791	0.002
		daytime	1	1.21	0.279	0.039
		daytime×chronotype	1	14.30	**<0.001**	0.323
	Incongruent trials RT	chronotype	1	0.25	0.619	0.008
		daytime	1	0.02	0.870	0.001
		daytime×chronotype	1	23.08	**<0.001**	0.435
	RT variability	chronotype	1	0.79	0.378	0.026
		daytime	1	0.35	0.558	0.012
		daytime×chronotype	1	25.60	**<0.001**	0.460
	N200-Fz (congruent trials)	chronotype	1	1.58	0.218	0.052
		daytime	1	3.06	0.090	0.096
		daytime×chronotype	1	19.04	**<0.001**	0.396
	N200-Fz (incongruent trials)	chronotype	1	2.56	0.120	0.081
		daytime	1	0.09	0.767	0.003
		daytime×chronotype	1	8.66	**0.006**	0.230
AX-CPT (sustained attention)	Accuracy	chronotype	1	0.38	0.541	0.013
		daytime	1	0.07	0.788	0.002
		daytime×chronotype	1	13.81	**<0.001**	0.315
	RT	chronotype	1	0.08	0.769	0.003
		daytime	1	5.21	**0.031**	0.146
		daytime×chronotype	1	14.55	**<0.001**	0.331
	P300-Pz	chronotype	1	0.69	0.412	0.023
		daytime	1	1.44	0.239	0.046
		daytime×chronotype	1	5.46	**0.026**	0.154

Note: BL = Block; RT = Reaction Time; SRTT = Serial Reaction time Task. d prime = this index of performance is calculated as d = ZHit – ZFA where Hit represents the proportion of hits when a signal is present (hits/(hits + misses)), also known as the hit rate, and FA represents the proportion of false alarms when a signal is absent (false alarms/(false alarms + correct negative)), the false-alarm rate. Significances are marked in **bold** (where $p < 0.05$).

5. I am not sure I understand the statement "Female subjects were not examined during the menstrual period to ensure hormonal changes would not interfere with their chronotype. Hormonal changes occur throughout the menstrual cycle and not just during bleeding. Please could the authors clarify?

RESPONSE: Thank you for the comment. We did not mean "during bleeding" but during the time when cortical excitability is shown to be affected by the menstrual cycle. We added more information to clarify [please see page 35 highlighted in blue].

REVISED text P.35:

Female subjects were not examined during the luteal phase of the menstrual cycle (around week 3 following menstruation) to ensure that hormonal changes that affect cortical excitability [3] would not interfere with their chronotype.

6. Lines 296-8 I understand that there is not space to put all the results in the main text, but it would be useful to briefly summarise the key points here.

RESPONSE: Thank you for the comment. We summarized the key points as suggested

REVISED text P. 17:

We also explored the association between motor learning, and plasticity by calculating correlations between MEPs amplitudes and motor sequence learning. Briefly, we found a positive correlation between anodal tDCS effects (MEP amplitude enhancement) and sequence learning (block 6 - 5 RT difference) in the evening for LCs (r=0.543, p=0.017). No significant correlation between sequence learning and tDCS-induced plasticity was found in ECs (for detailed results see Supplementary Material)

7. Line 365 – should read chronotypes

REVISED text P.21:

Here we showed a converging impact of chronotypes on time-of-day dependent behavioural/cognitive performance of healthy individuals, and demonstrated the physiological foundations of these effects by daytime-dependent cortical excitability, neuroplasticity, and brain information processing processes.

8. I like the idea of figure 6, but I am not sure that it adds anything to the study. Could it be a graphical abstract? Or maybe there could be some mechanistic hypotheses added? At the moment it seems somewhat redundant.

RESPONSE: Thank you for the comment. We updated figure 6 with a proposed mechanistic hypothesis.

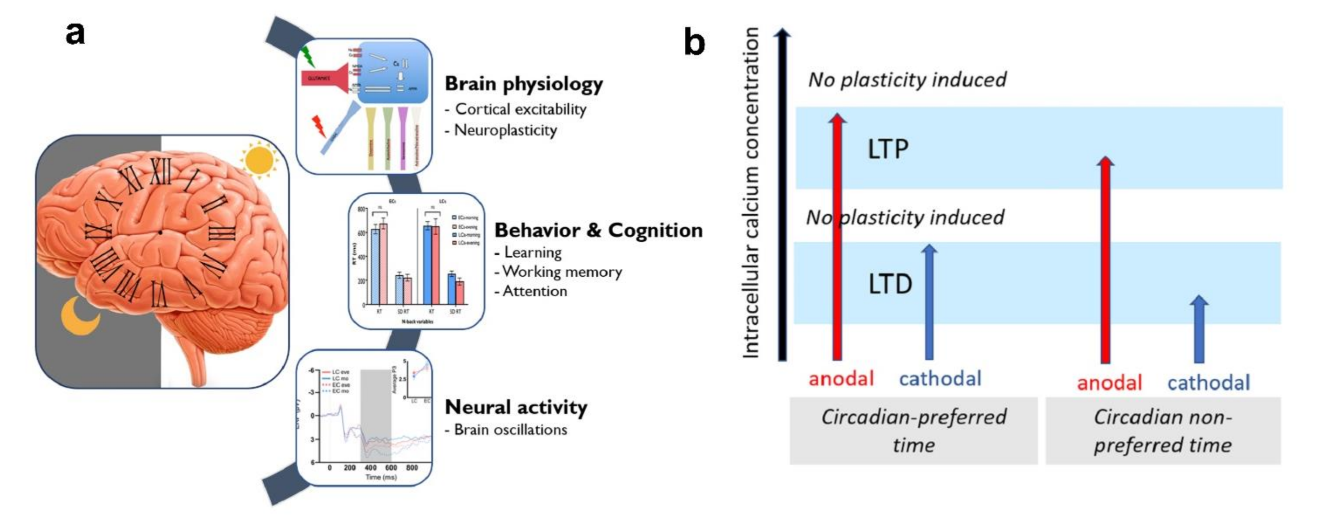

Fig. 6: **a**, A schematic illustration depicting the converging impact of chronotype on brain physiology, behavior, and cognition. **b**, Proposed mechanism of the neuroplasticity induction at circadian-preferred and non-preferred time based on the association between intracellular calcium concentration (x-axis) and induction of tDCS-induced neuroplastic changes. Gradual enhancement of calcium concentration can either induce LTD, LTP or no plasticity. The LTP-like plasticity induced by tDCS is linked to higher intracellular calcium concentration while LTD-like plasticity induced by tDCS is linked to a lower intracellular calcium concentration. It can be

assumed that intracellular calcium concentration at the optimal time of the day is at an optimal level leading to stronger tDCS-induced LTP/D like plasticity. This would be at least partially related to the higher glutamatergic and lower GABAergic activation at the circadian preferred time, shown by cortical excitability data.

9. Line 497 – should read "one week" I imagine.

REVISED text P.27:

Following the resting-EEG acquisition, participants performed motor learning and cognitive (working memory, attention) tasks in two randomly-assigned sessions at the same time in the morning and evening while their EEG was recorded (one-week interval).

Reviewer #2 (Remarks to the Author):

I really enjoyed reading this manuscript. It is very well written, thoughtful with a measured narrative based on a set of studies that were clearly a great deal of work examining multiple levels of the biological hierarchy from synaptic function to behaviour. The work is nicely illustrated and convincing. I have some comments that may strengthen the work further.

RESPONSE: We appreciate the reviewer's time and effort in reading our work, positive evaluation of our work, and the insightful comments s/he offered. In what follows, we provide a one-by-one response to each comment and revised the main text accordingly. For the convenience of the reviewer, we pasted the revised version of the text here as well in highlighted in green or blue.

1- Motor cortical excitability changed with the preferred time of day. As a consequence, are the differences in, for example, the double TMS experiments simply because a different stimulation intensity is being used and so what is being reported is simply a different position within the double pulse curve? For example, and admittedly an extreme example for illustrative purposes – at the preferred time of day 30% of machine output might be applied but at the non-preferred time of day 80% of machine output is used (due to changes in excitability). Then a change in the curve is really because there is a change in stimulation intensity (due to excitability changes) as opposed to some additional change that is specifically revealed in the double pulse experiments. Is it potentially possible that if the machine output had stayed the same – the excitability would show a change (MEP magnitude with chronotype and time of day) – however the double pulse curve would not differ significantly? Thus, the differences in the double pulse curve are driven by changes in excitability (i.e., non-independent measure). I think this is an important issue, which regardless of the concern being misplaced (or not) needs discussed to help other readers.

RESPONSE: Thank you very much for the comment. Due to lack of space, we had described this information in the supplementary material. The threshold values for all TMS protocols including baseline/test-pulse sensitivity to TMS (defined as the percentage of maximum stimulator output required for the SI1mV MEP amplitude), MEP amplitude at RMT (for I-O curve protocol), %MSO for RMT, and SI1mv are presented in the supplementary material and there was no significant difference between these values. This rules out that changes in the excitability were due to changes of stimulation intensity [Please see section 1.1 and Tables S1, S2 in the supplementary materials highlighted in blue].

REVISED text, supplementary material:

1.1. TMS protocols threshold values

MEP values of single-pulse conditions and other control conditions, as well as subject-specific baseline sensitivity to TMS (defined as the percentage of maximum stimulator output required for the SI1mV MEP amplitude), are summarized in Table S1. For each TMS protocol, we compared single-pulse condition MEPs (control condition) and the SI1mV obtained at different daytimes by a 2-factorial ANOVA with daytime (morning, evening) as the within-subject and chronotype (ECs, LCs) as the between-subject factor. The results of the respective ANOVAs show that the mean values of the single-pulse conditions MEP, MEP intensity at RMT (for I-O curve protocol), %MSO for RMT, and SI_{1mv} did not significantly differ between time of day and groups (Table S2). Only for AMT, the interaction of chronotype×daytime was significant, and post hoc t-tests revealed a significant difference between AMT values only for ECs across daytime (t = 3.58, *p* = 0.003).

Table S1: Baseline measurements of the cortical excitability protocols

Protocol	measurement	daytime	Group	
			ECs	LCs
Single-pulse MEP	$SI\ 1_{mV}$ (%)	morning	51.31 ± 9.82	50.43 ± 8.46
		evening	50.50 ± 10.19	50.87 ± 7.91

RMT	%MSO	morning	42.87 ± 7.54	41.81 ± 6.88
		evening	41.68 ± 7.15	42.12 ± 7.09
AMT	%MSO	morning	35.18 ± 6.95	34.40 ± 8.23
		evening	33.53 ± 6.97	34.96 ± 8.18
I-O curve	MEP amplitude at RMT	morning	0.235 ± 0.213	0.197 ± 0.204
		evening	0.149 ± 0.091	0.200 ± 0.133
SICI-ICF	Single-pulse MEP	morning	1.073 ± 0.335	0.941 ± 0.152
		evening	0.910 ± 0.342	0.980 ± 0.121
I-wave facilitation	Single-pulse MEP	morning	1.020 ± 0.341	1.041± 0.119
		evening	1.237 ± 0.512	1.178 ± 0.438
SAI	Single-pulse MEP	morning	1.237 ± 0.512	1.178 ± 0.438
		evening	1.194 ± 0.269	1.195 ± 0.175

Note: MEP = motor-evoked potential; ECs = early chronotypes; LCs = late chronotypes; SI_{1mv} (%) = maximum stimulator output (%MSO) required for the SI1mV MEP amplitude; I-O curve = input-output curve; SICI-ICF = short latency intracortical inhibition and facilitation; SAI = short-latency afferent inhibition; RMT = resting motor threshold; AMT = Active motor threshold.

Table S2: Results of 2 × 2 ANOVAs for control MEPs (RMT intensity MEP for I-O curve, single-pulse MEP for other protocols), SI1mV, and %MSO for RMT/AMT.

**Protocol**	**Measurement**	**Factor**	***df***	***F***	***p***
Single-pulse MEP	SI_{1mV}	daytime	1	0.12	0.724
		chronotype	1	1.40	0.244
		daytime×chronotype	1	0.001	0.938
RMT	%MSO	daytime	1	0.86	0.359
		chronotype	1	0.01	0.901
		daytime×chronotype	1	2.54	0.120
AMT	%MSO	daytime	1	3.23	0.082
		chronotype	1	0.015	0.903
		daytime×chronotype	1	13.30	<**0.001**
I-O curve	RMT intensity MEP	daytime	1	1.04	0.315
		chronotype	1	0.02	0.882
		daytime×chronotype	1	1.169	0.288
SICI-ICF	Single-pulse MEP	daytime	1	0.98	0.329
		chronotype	1	0.22	0.641
		daytime×chronotype	1	2.65	0.113
I-wave facilitation	Single-pulse MEP	daytime	1	0.23	0.631
		chronotype	1	0.83	0.367
		daytime×chronotype	1	0.37	0.547
SAI	Single-pulse MEP	daytime	1	0.017	0.895
		chronotype	1	0.112	0.739
		daytime×chronotype	1	0.090	0.765

Note: MEP = motor-evoked potential; chronotype = early (ECs) and late (LCs) chronotypes; SI_{1mv} (%) = maximum stimulator output (%MSO) required for the SI1mV MEP amplitude; I-O curve = input-output curve; SICI-ICF = short latency intracortical inhibition and facilitation; SAI = short-latency afferent inhibition; RMT = resting motor threshold; AMT = active motor threshold.

2- It would be helpful to know time of sleep onset, time of waking, sleep duration, and the

duration from waking to the start of the experiment for each participant (if possible). For example, from information through sleep-logs. I am suggesting this for two reasons: (a) the relationship between sleep and plasticity is topical (see below) and (b) to provide further information about the circadian cycle of the different groups (i.e., the phase lag between the groups).

RESPONSE: Thank you for the suggestion. We did not force participants to go to bed at a specific time but defined a range in which subjects should have gone to sleep (from 22:00-2300). Also, participants had to have at least 8 h of sleep and we controlled for these parameters before each experimental session. Additionally, we measured participants' sleepiness before each session and results show no significant difference across groups. In case there was a significant sleepiness, the session was held [please see page 37, highlighted in blue and yellow]. More details are explained in response to the next comment.

REVISED text, p 37:

Experiment timing, sleep/wake cycle, and light variation

Timing of the experiments was determined based on the average of sleep times and level of activity of early and late chronotypes based on previous studies [4] as well as daily life routine schedules. For morning sessions, the start time was chosen based on the norm start of schooling and working (8:00 in the morning). This is also a time when both chronotypes have the lowest levels of activity [4]. The evening time (19:00) was specified based on the time when both chronotypes have a comparable level of activity according to previous dataset profiles [4]. Both groups were instructed to go to bed (at around 22:00-23:00), and get up in the morning at identical times of the day, and have at least 8 hours of sleep for morning sessions ($mean_{ECs}$ = 8.41, $mean_{LCs}$ = 8.11). For the evening session, participants were allowed to go to bed at their preferred time but before 24:00 and were also allowed to have more than 8 hours of sleep if needed ($mean_{ECs}$ = 8.36, $mean_{LCs}$ = 8.38). All participants were strongly recommended to comply with the sleep/wake cycle instruction and in case of irregularities, poor quality of sleep, and sleepiness (measured by participants' rating before each session), the experimental session was postponed to another day. Subjective sleepiness of participants and their alertness were evaluated with the Karolinska sleepiness scale (KSS)[5] which measures sleepiness in a 1-10 Likert type scale (supplementary materials). No significant difference was observed between the sleepiness ratings of both early and late chronotypes in the morning sessions ($mean_{ECs}$ = 3.2, $mean_{LCs}$ = 3.71; t = 1.61, p = 0.11) and evening sessions ($mean_{ECs}$ = 3.58, $mean_{LCs}$ = 3.65; t = 0.25, p = 0.80).

3- Sleep and plasticity have been linked. For example, sleep deprivation impairs the same brain stimulation markers of plasticity used in the current work. Plasticity might then reasonably be expected to be higher in the hours following sleep than after substantial time awake. At first sight this would appear inconsistent with the current work that places the emphasis on chronotype rather than sleep proximity upon modifying plasticity. Perhaps these principles operate concurrently in all individuals, which would suggest that sleep proximity had some influence upon the plasticity measures – for instance, is plasticity "enhancement" at the circadian preferred time greatest when proximal to sleep? This would predict that plasticity and performance are greatest when the preferred time of day is most proximal to sleep. Broadly I am seeking for a way to reconcile both chronotype and sleep as important modifying factors upon plasticity.

RESPONSE: Thank you for the suggestion. The reviewer's point makes sense and we had in mind that sleep could interfere with chronotype-related results in case participants do not have enough sleep especially for the morning sessions. As explained in response to the previous comment, we controlled for sleep-wake cycle and obtained a sleepiness rating before each session and added respective information in the revision [please see previous comment p.37, highlighted in blue and yellow]. Furthermore, this concern was raised also by the other reviewers. In order to address this concern, we provide new data from another project in which we measured the impact of lack of sleep on the same physiological parameters. Our results showed a completely different pattern of cortical excitability and neuroplasticity as a result of sleep deprivation. This shows that plasticity will be affected differently

under lack of sleep compared to preferred vs non-preferred time of day. We also analyzed resting EEG theta activity which is a marker of sleep pressure and found no significant differences [please see p. 21. Highlighted in yellow]. For more details on these new analyses please refer to our response to the 3rd reviewer.

REVISED text, p 21:

No difference in sleepiness rating and physiological marker of sleep pressure across groups

The different sleep-wake cycle of early and late chronotypes indicates that both groups could have different sleepiness which can interfere with chronotype-specific effects on brain physiology and cognition. To resolve this concern, we controlled the sleep-wake cycle and sleep duration of both groups within a defined range (see methods for details) and rated participants' sleepiness with the Karolinska Sleepiness Scale before each test session. All participants had at least 8 h of sleep before each session (see methods). The results of ANOVA showed no significant interaction of chronotype×daytime (F_1=1.03, p=0.325) or main effects of chronotype and daytime. This indicates that there was no significant difference between the sleepiness ratings in or between both groups across different times of day. Furthermore, we analyzed resting EEG theta oscillations, which is a marker of sleep pressure[12] in both groups in the morning and evening and found no significant differences in the theta oscillations when we compared both groups in the morning and evening. No significant differences were neither observed in each group across different times of the day. The same pattern of results was observed for alpha oscillations (supplementary materials).

4- It would also be helpful to know whether there was any seasonal variation in the recruitment and conduct of the experiments. The interest here being in seasonal light variation and its entrainment upon sleep-wake/circadian cycles. It would be reassuring to know or have stated that the experiments were randomly distributed across seasons on the different chronotypes.

RESPONSE: Thank you for the comment. We added respective information in the text [Please see p.37-38, highlighted in green].

REVISED text, p 37-38:

Due to the variability of seasonal light, experimental sessions did not take place in summer when the difference between sunrise and sunset is largest in Germany. For other months, the experiment was scheduled to be randomly distributed across other seasons in both groups. Furthermore, ambient light was kept constant (around 500 lux) across all experimental sessions in the morning and evening. In the cognitive sessions, tasks and EEG recordings were conducted in a sound-proof shielded room with constant temperature of 24-25°C for morning and evening sessions.

5- Brain stimulation has been used to provide insight into synaptic function. One of the great strengths of this is that it allows plasticity to be measured in humans. However, as yet, there is not any direct evidence showing that stimulation affects synaptic function. There is good circumstantial evidence (i.e., those factors, e.g., pharmacological that modify plasticity also modify the effects of stimulation), but there is no direct evidence that stimulation in the living human modifies plasticity. It might be worth acknowledging the extent of the strength of evidence for brain stimulation as a measure of plasticity to provide further balance.

RESPONSE: Thank you for the suggestion. We absolutely agree and mention this limitation in the discussion [Please see p.27, highlighted in green].

REVISED text, p 27:

Finally, although NIBS allows us to causally modify and induce neuroplasticity in humans non-invasively, it is worth to acknowledge that the evidence for synaptic plasticity induction by NIBS, including tDCS, comes from animal and pharmacological studies, and is thus indirect[13-15].

6- The sequence learning task uses a delay between the response and subsequent stimulus of 500ms. A delay of this duration has been associated with participants developing awareness for the sequence more easily than shorter delays (for example of 200ms). Yet, it

doesn't appear as though any participant became aware of the sequence, and nor the criteria/criterion for their removal.

RESPONSE: Thank you for the comment. We adopted the task features from versions used in previous works of our group which showed that it measures implicit motor learning [16]. Furthermore, 500 ms is the commonly used interval in other studies with SRT tasks as well [17, 18]. But it is worth noting that participants are instructed to respond as fast as possible and RT is the primary outcome of interest. We explained in the task description [page 46, highlighted in green] about the awareness criterion and excluded the data if the sequence was correctly identified. Indeed, one EC participant correctly identified the sequence and his/her data was excluded as shown in Fig.4 legend [page 32, highlighted in green].

REVISED text, p 32:
All pairwise comparisons are calculated using *Student's* t-test. *n*=31 (15 ECs, 16 LCs; data of one EC participant excluded due to sequence awareness). All error bars represent the s.e.m across participants.

REVISED text, p 46:
Participants were not told about the repeating sequence and at the end of the session, they were asked whether they noticed a sequence and if so, to write the sequence in order to assess explicit learning of the task. In such case, respective data were excluded from the final analysis.

7- I wasn't quite sure what the statement "two different versions of the task were used in the two sessions" (see page 43) meant. I thought perhaps it meant that different sequences were used during the two sessions. If so, it would be helpful to know what those sequences were, if there were any triplets in common, and any effect of order. If not, does this mean that the same sequence was used during the two sessions – wouldn't this lead to an order effect with transfer of skill to the second session – and then what does it mean that different versions were used?

RESPONSE: Thank you for the comment. We clarified this point [please see pages 46, highlighted in green].

REVISED text, p 46:
Two different sequences of the task, with no overlapping parts, and comparable difficulties, were presented in the two sessions in counterbalanced order.

8- The duration of each trial in the sequence learning task was determined by the participant's response time. As a consequence, faster response times lead to shorter trials and more frequent movements. The greater learning during preferred times would lead to faster response times and more frequent movements. This then leads to a confound in the interpretation of the EEG work: are the differences due to learning, or the movement frequency associated with greater learning. Either way the EEG work provides nicely converging evidence with the behavioural work --- it is more an issue of being clear about what is shown by the EEG and the limits of interpretation.

RESPONSE: Thank you for your comment. We have to clarify that in this task the frequency of finger movements is fixed across sessions. Therefore, faster RT does not mean more frequent movements but faster movement due to enhanced learning, which would however also enhance movement frequency. This is a point worth considering and we highlight it in the EEG results [please see p. 16, highlighted in green].

REVISED text, p. 15:
Together, these results highlight the relationship between the circadian-preferred time and recruitment of motor learning-specific electrophysiological processes that are associated with performance. One aspect to consider in interpreting the ERP amplitude enhancements at the circadian-preferred time is however that these could reflect enhanced learning, but also the faster frequency of movements caused

by learning-related faster RT.

9- Was there a correlation between the performance in the various behavioural tasks? An absence of a correlation would suggest that the effects of chronotype upon behaviour operate independently upon one another; alternatively, a correlation might suggest that the effects are really due to changes within one task (and associated circuits) driving changes in another task. Earlier work has suggested a relationship between working memory and sequence learning (for example the work by Racheal Seidler and colleagues).

RESPONSE: Thank you for suggestion. This is an interesting point however, out study was not powered for this type of correlational analyses. Nonetheless, we explored the suggested correlations and added the results to the supplementary material (please refer to supplementary material, page 11, highlighted in green). No correlations were observed at the whole group data (both ECs and LCs). In addition, in early chronotypes, no significant correlations were found between working memory and motor learning in the morning, but a negative correlation between performance speed in both tasks was observed for LCs in the evening. For the other tasks, we found no significant relevant correlations as well. Overall, these data show no robust correlations between the performance of different tasks.

REVISED text, supplementary material, p 12:

1.1. Correlation between cognitive tasks

We also explored correlations of performance between the cognitive tasks. No significant correlations were observed between working memory and motor learning at the group level and in ECs alone. In LCs, however, a significant correlation was found between working memory RT in the evening and the sequence learning block 6 of the SRTT in the evening (r_{BL5}= -0.513, p=0.042; r_{BL6}= -0.614, p=0.011) indicating faster RT in both tasks in the evening were associated. For the other tasks, no significant correlations were found. Overall, these data show no robust correlations of performance between the different tasks.

10- The work seems to have implications for the design of further studies. For example, the removal of participants that have a clear chronotypes preference. The inclusion of such participants could perhaps reduce the appearance of a circadian influence on excitability, plasticity and behavioural performance?

RESPONSE: Thank you for your comment. We absolutely agree and had briefly mentioned this in the discussion, but added more details in the revision [please see p. 25, highlighted in green]. These results suggest that chronotypes should be screened in NIBS studies that investigate brain physiology and cognition. We don't necessarily suggest exclusion of early or late chronotypes, but e.g. adapting the experiment time at the circadian preferred time, or identify a time of the day which works for all chronotypes reasonably well. This is a novel finding in the field of NIBS that calls for studying potential factors that contribute to variability of results in NIBS research.

Revised text, p 25:

The findings of this study have specific implications for the field of human neurophysiology and cognitive neuroscience as well as broad implications for human behavior in healthy and clinical populations. "Time-of-day" and chronotype are important, but less-studied determinants of cortical plasticity induction by NIBS techniques [6, 7]. Our results show that cortical excitability and neuroplasticity are chronotype-dependent in humans. It is tempting to speculate that chronotype could account for variability of NIBS effects and might explain partially heterogeneous effects in previous studies. This assumption might be likewise relevant for the performance of various cognitive tasks and suggests to screen or consider these modulatory effects.

Reviewer #3 (Remarks to the Author):

Salehinejad et al. investigated chronotype-dependent modulation of human brain function in a multimodal approach including electrophysiology, neurostimulation and cognition. I first want to acknowledge the large amount of work involved and multiple aspects considered as well as the exhaustive methodological explanation favouring replicability. There is however a fundamental misconception of the study protocol that invalidates all the chronotype-related interpretations and turns down my initial enthusiasm. Because of this, the intrinsic novelty of the topic and potential findings with respect to existing literature is questionable.

RESPONSE: Thank you for reading our work and providing important comments and suggestions. We understand the concerns raised by the reviewer and in what follows we address them. Some of the concerns were due to a misunderstanding caused by a lack of information we provided in the original version. Due to the word limit and the study size (that includes 4 different large datasets), we had to choose the most relevant information. We now added this information in the revision. Furthermore, we have additional data from another dataset (in what follows) as well as new data from this dataset (EEG) that addresses the major concern of this reviewer. For the reviewer's convenience, we pasted the changes of the main text here as well.

MAJOR ISSUES

1. The protocol does not allow to conclude on chronotype differences for the following reasons:

1.1. clock time of data acquisition was fixed at 8:30AM and 7PM. Chronotype not only differ in the preferred time of day to perform tasks but also in the sleep-wake schedule, particularly so in extreme chronotype which, presumably, constituted part of the sample. This means that for a morning chronotype 8:30AM and 7PM can correspond to respectively, e.g. ~2.5h and 11h after wake up time, while for an evening time this could correspond to 2h BEFORE wake up time and 8.5h after wake up time. In other words the authors are not comparing comparable things across their 2 groups of interest. Nb: The term "preferred time" is therefore not applicable to this study, particularly for the evening type.

1.2. sleep-wake history was not controlled. There is no report of a regular sleep schedule prior to the study. This means that i) we cannot place any of the participant in time to response to the uncertainty reported in 1.1., ii) we do not know whether participants slept similarly between both experimental sessions which took place 1 week apart and iii) we do not know how much participants slept prior to the study when previous literature clear established the influence of lack of sleep on basic cortical function. It would not be surprising to have an evening participant sleeping less for the morning assessment and at different times in order to cope with the very early 8:30AM session

1.3. we do not know whether any regular sleep would be the preferred schedule or an imposed schedule due to social obligation or because the experimenter imposed a fixed schedule. This means that when comparing chronotypes, the authors may be in fact assessing the impact of time-of-day / elapse time wake from ~2h before wake up time (i.e. following partial sleep loss) to ~11h after wake up time across 2 groups of participants that completd part of the protocol. In support of this assumption (which I cannot proof or disproof due to lack of information), part of the data of evening type in the evening correspond to an intermediate between morning and evening session in morning time, when considering values in absolute terms (e.g. Fig 2a, Fig 3 b-c). This also implies that a lot of the potential novelty of the manuscript (i.e. chronotype) is removed.

Thank you for the comments. In what follows, we address the concerns raised by this reviewer by adding additional information, data, and analyses:

First (in response to 1.1), We mention in the methods that all participants were *moderate* early or late chronotype and extreme types were excluded to reduce sleep-wake schedule variability. We did not

explicitly mention this in the original version of the manuscript due to space limits but now clarified this in the revision and provide numerical scores of DMEQ scores as well [please see page 36-37, and below highlighted in blue].

Second (in response to 1.1, 1.2, 1.3), As we clarify in the revision, both groups had to have at least 8 h of sleep. Furthermore, subjective sleepiness was rated before each session in the morning and evening and results showed no significant difference neither in the morning nor in the evening sessions across groups [please see p.37, highlighted in blue and yellow]. According to the two-process model of sleep regulation [19, 20], the magnitude of sleepiness and alertness is a function of sleep (S) and circadian rhythms (C). Non-significant differences in sleepiness, therefore, indicate that process S (sleep pressure) was not significantly different across groups. Furthermore, before each testing session, the time to go to bed and wake up were standardized and identical for all subjects. This should resolve the concerns about "*different sleep-wake cycle*" and "*partial sleep loss*" especially for the evening types.

Third (in response to 1.3.), sleep-wake history was indeed controlled 24 hours before each experimental session. Participants had to have 8 hours of sleep before each experimental session, and subjective sleepiness was assessed (no significant difference between groups). In case this criterion was not met and participants felt sleepy, respective sessions were postponed (we had to cancel 15 sessions during the experiment due to not meeting this criterion, proofed by high sleepiness ratings in the KSS questionnaire).

Fourth (in response to 1.1 & 1.3), this experiment was meant to examine the impact of chronotype and not sleep, which is why participants had standard sleep duration, and sleep times before the experimental sessions. Testing each group at different times in the morning and evening, makes it challenging to compare the results of each group due to different timing, and moreover, "optimal" times might gradually differ between individuals (and there is no objective criterion to make clear decisions), thus in our opinion there is no ideal solution in studies that have a "chronotype-based paradigm"[20]. Therefore, we decided to pick a fixed time for each group that has the closest correspondence to their circadian preference according to previous works, as described later. Any other time of measurement would raise the same concerns, according to the reviewer's points. Ensuring that participants had at least 8 h of sleep and evaluating subjective sleepiness before each session was the most reasonable and feasible option to rule out any potential impact of sleep loss or pressure. Nonetheless, we mention this point in the limitations of the study [please see p.26, highlighted in yellow].

Revised text, page 36-37: Definite evening (16-30), moderate evening (31-41), intermediate or neutral (42-58), moderate morning (59-69) and definite morning (70-86) are the five chronotype categories identified by the MEQ. The questionnaire shows high reported reliability and has a significant correlation with circadian rhythm-related hormonal changes, including melatonin[10]. In addition to the MEQ, we defined the dichotomous chronotype phenotype by asking two identical questions ("Are you naturally a night person or a morning person?")[11]. Individuals with discordant or neutral responses to both were excluded. The mean scores of chronotype for the early and late chronotypes were 62.25±3.47 and 35.37±4.31, showing that both groups represent moderate early and late chronotypes. Extreme chronotypes were excluded to reduce sleep-wake schedule variability.

Revised text, page 37: Timing of the experiments was determined based on the average of sleep times and level of activity of early and late chronotypes based on previous studies [4] as well as daily life routine schedules. For morning sessions, the start time was chosen based on the norm start of schooling and working (8:00 in the morning). This is also a time when both chronotypes have the lowest levels of activity [4]. The evening time (19:00) was specified based on the time when both chronotypes have a comparable level of activity according to previous dataset profiles [4]. Both groups were instructed to go to bed (around 22:00-23:00), and get up in the morning at identical times of the day, and have at least 8 hours of sleep for morning sessions ($mean_{ECs}$ = 8.41, $mean_{LCs}$ = 8.11). For the evening session, participants were allowed to go to bed at their preferred time but before 24:00 and were also allowed to

have more than 8 hours of sleep if needed ($mean_{ECs}$ = 8.36, $mean_{LCs}$ = 8.38). All participants were strongly recommended to comply with the sleep/wake cycle instruction and in case of irregularities, poor quality of sleep, and sleepiness (measured by participants' rating before each session), the experimental session was postponed to another day. Subjective sleepiness of participants and their alertness were evaluated with the Karolinska sleepiness scale (KSS)[5] which measures sleepiness in a 1-10 Likert type scale (supplementary materials). No significant difference was observed between the sleepiness ratings of both early and late chronotypes in the morning sessions ($mean_{ECs}$ = 3.2, $mean_{LCs}$ = 3.71; t = 1.61, p = 0.11) and evening sessions ($mean_{ECs}$ = 3.58, $mean_{LCs}$ = 3.65; t = 0.25, p = 0.80).

Revised text (page 26): It is important to consider that our study design has a chronotype-based design which is appropriate for studying the influence of circadian rhythmicity peaks on performance variations in optimal and non-optimal time of day[20]. While this approach has the advantage of studying cognition and brain function under normal day-night condition, it cannot take into account the interplay between clock- and sleep-dependent processes into account[20]. Considering that the sleep-wake cycle in different chronotypes is not identical, this might raise concerns about its potentially confounding effects. According to the two-process model of sleep regulation, the magnitude of sleepiness and alertness is determined by the interacting influence of sleep pressure, and circadian rhythms. The control over timing to go to bed and sleep duration during this experiment and non-significant difference between the sleepiness ratings before each session across groups indicates that potential sleep loss or different sleep-wake cycles do not account for the observed effects. Our resting-EEG data also confirmed no significant difference between theta oscillations, as a marker of sleep pressure[12], in the morning or evening sessions across groups.

Finally (overall response to major concerns), we have a new dataset available that hopefully resolves the reviewer's major concern about confounding effects of different sleep-wake cycles and resultant sleepiness. The data of this work come from a larger research project in which we systematically investigated the impact of both "***chronotype***" and "***lack of sleep***" on brain physiology, and cognition. We recently finished the second part of the project about the impact of sleep deprivation on the same physiological parameters we obtained for the chronotype study. Data of this work explicitly show that *lack of sleep* has a fundamentally different impact on brain physiology than chronotype, which addresses the concern about the potential confounding impact of different sleep-wake schedules of early vs late chronotypes and partial sleep loss of the latter group (although we controlled for this and reported sleepiness ratings above). In this new dataset, 14 of 30 participants were evening-types and they underwent the same measures (cortical excitability, neuroplasticity) after sufficient sleep and after lack of sleep. The measurement time was approximately identical (8:30 in the morning). We provide results of these groups of participants in the figures below. As shown in **Fig 1**, physiological parameters under lack of sleep have a completely different pattern compared to non-preferred time in late chronotypes (**Fig. 2**). Sleep deprivation leads to a significant increase and decrease of intracortical facilitation and inhibition respectively (**Fig. 1A-D**), while an opposite pattern occurs when participants are explored at their non-preferred circadian time (i.e., morning) (**Fig. 2A-D**). Similarly, qualitatively different results were obtained for neuroplasticity induction. Sleep deprivation diminished LTP-like and reversed LTD-like plasticity to LTP-like plasticity due to cortical *hyperactivation* in late chronotypes (**Fig. 1E-F**). Being at non-preferred time (morning), however, induces both LTP and LTD-like plasticity, but to a lesser extent than during the preferred time due to cortical *underactivation* (**Fig. 2F**).

We believe these results clearly suggest that if late-chronotypes had lack of sleep in the morning sessions (due to a different sleep-wake cycle) that could interfere with chronotype effects, physiological parameters should have been altered differently than those observed in the chronotype study. With these new data, we think that the results of the chronotype study are completely novel and have not been shown by previous works. We do hope this new dataset resolved the reviewer's major concerns and if s/he thinks adding this data to the manuscript is required, we would be willing to add results of these 14 participants from our sleep experiment.

Specific comments:

In response to comment 1.1. The term “*preferred*” was simply chosen to convey our point throughout the text because chronotype is defined as circadian-*preference*. Furthermore, according to the MEQ (item 11), the best time that fits the internal clock of evening types is 7:00-9:00 PM [21] which shows that our evening time measure can be labeled “*preferred*” time for the evening type. If this reviewer, however, prefers a different wording, we would be happy to change.

In response to comment 1.3. We are not sure if we understand the reviewers’ point based on his/her assumption (in support of…). But for figure 2a, the values are indeed absolute, this was a typo. We are sorry for that and corrected it in the revised version [please see Fig 2, page 10].

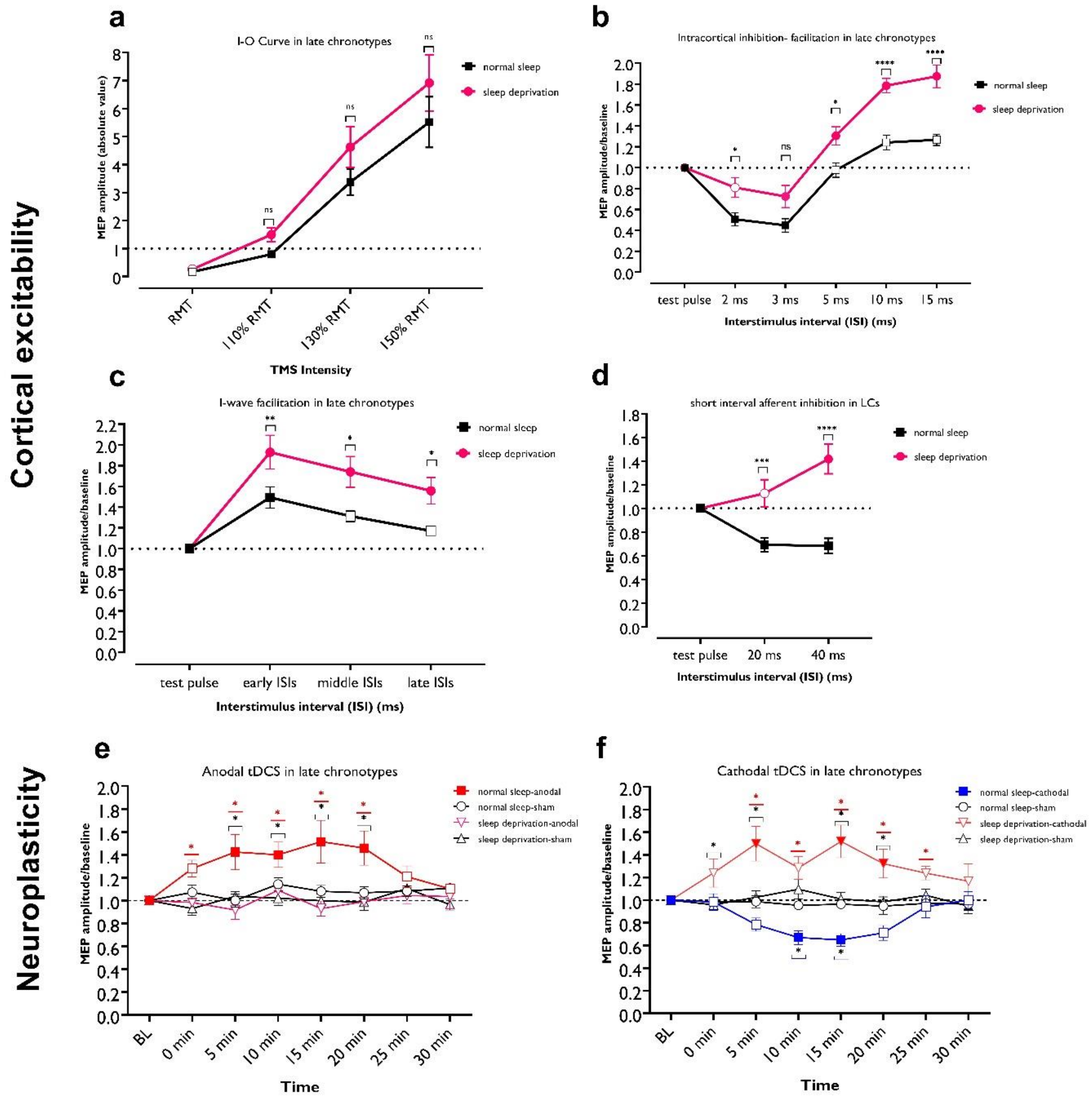

Fig. 1. Cortical excitability and neuroplasticity induction following sufficient sleep vs sleep deprivation in late chronotypes. **a**, Global cortico-spinal excitability monitored by MEP amplitudes at different TMS intensities via the I-O curve protocol. **b**, Intracortical inhibition and facilitation measured by the SICI-ICF paired-pulse TMS protocol. **c**, GABA-dependent intracortical inhibition after normal sleep vs sleep deprivation. **d**, Short latency afferent inhibition after normal sleep vs sleep deprivation. **e**, LTP-like plasticity induction in the motor cortex after normal sleep vs sleep deprivation. **f**, LTD-like plasticity induction in the motor cortex after normal sleep vs sleep deprivation. All pairwise comparisons were calculated using Bonferroni correction for multiple comparisons (*n* = 14). All error bars represent the standard error of means (s.e.m.). Filled symbols

represent a significant difference of MEP amplitudes compared to the respective test pulses. Asterisks represent statistically significant comparisons between sleep conditions. in **e** and **f** the black asterisks [*] indicate a significant difference between the real vs sham tDCS conditions, and the red asterisks [*] indicate a significant difference between respective timepoints of tDCS condition after normal sleep vs sleep deprivation.

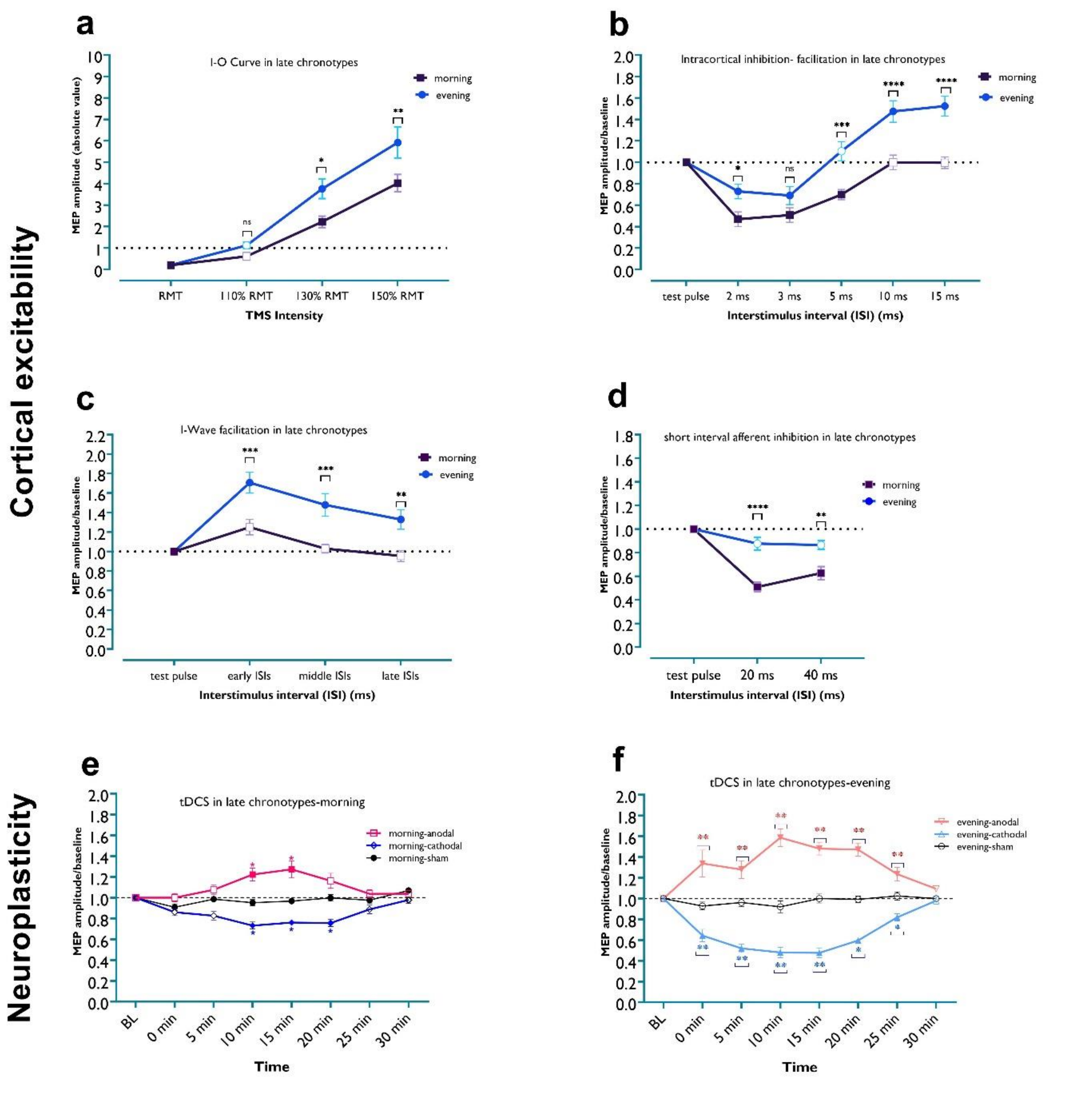

Fig. 2. Cortical excitability and neuroplasticity induction in late chronotypes at preferred and non-preferred times. a, Global cortico-spinal excitability monitored by MEP amplitudes at different TMS intensities via the I-O curve protocol. **b** Intracortical inhibition and facilitation measured by the SICI-ICF paired-pulse (pp)TMS protocol. **c** I-Wave facilitation for monitoring GABA-dependent intracortical inhibition. **d** Inhibitory effect of peripheral nerve stimulation on motor cortical inhibition, as measured by SAI. **e, f** post-tDCS cortical excitability alterations after anodal, cathodal, and sham stimulation at the circadian non-preferred (**e**) and preferred times (**f**) in LCs (n = 16). All pairwise comparisons are calculated using the Bonferroni correction for multiple comparisons. All error bars represent the standard error of means (s.e.m) across participants. In a-d, asterisks represent statistically significant comparisons and filled symbols represent a significant difference of MEP amplitudes compared to the respective test pulses. In **e** and **f** symbol [*] indicates a significant difference between the real vs sham tDCS conditions, and the floating symbol [**] indicates a significant difference between respective timepoints of tDCS condition at the circadian-preferred vs circadian non-preferred times. In **e** and **f**, filled symbols represent a significant difference of MEP amplitudes compared to the baseline MEP.

Evidence from resting EEG data: In addition to the data shown above, resting-EEG supports our claim on non-significant differences in the sleep-wake cycle and sleep loss of both groups in the morning. According to the two-process model [22], sleep regulation is determined by the interaction between sleep/wake cycle (Process S) and circadian rhythms (Process C). Process S, which sleep pressure, increases during wakefulness and declines during sleep. This process has several physiological markers including NREM sleep, SWA, and EEG. Theta activity in the awake state is a marker of the rise of S [19] occurring during sleep pressure. Recent sleep deprivation studies confirmed theta activity as a marker of sleep pressure as well [12]. We recorded resting EEG data in each group in the morning (8:30) and evening (19:00) sessions. We compared resting EEG theta oscillations of both ECS and LCs in the morning and the results show no significant difference. This shows that LCs in the morning were not under sleep pressure due to partial sleep loss. Theta oscillations for each group across the morning and evening sessions were significant neither. We would be happy to add this EEG new analysis to the main results [please see p. 21, highlighted in yellow].

Electrode		*t*	*Adjusted P Value*
Fz	ECs-morning vs. LCs-morning theta oscillations	0.33	>0.999
Cz	ECs-morning vs. LCs-morning theta oscillations	0.48	>0.999
Pz	ECs-morning vs. LCs-morning theta oscillations	1.22	>0.999
Fz	ECs-evening vs. LCs-evening theta oscillations	2.18	0.181
Cz	ECs-evening vs. LCs-evening theta oscillations	1.80	0.433
Pz	ECs-evening vs. LCs-evening theta oscillations	0.50	>0.999
Fz	ECs-morning vs. ECs-evening theta oscillations	2.07	0.238
Cz	ECs-morning vs. ECs-evening theta oscillations	1.27	>0.999
Pz	ECs-morning vs. ECs-evening theta oscillations	1.51	0.790
Fz	LCs-morning vs. LCs-evening theta oscillations	0.21	>0.999
Cz	LCs-morning vs. LCs-evening theta oscillations	0.05	>0.999
Pz	LCs-morning vs. LCs-evening theta oscillations	0.18	>0.999

Revised text, page 21:

No difference in sleepiness ratings and physiological markers of sleep pressure across groups

The different sleep-wake cycle of early and late chronotypes indicates that both groups could have different sleepiness which can interfere with chronotype-specific effects on brain physiology and cognition. To resolve this concern, we controlled the sleep-wake cycle and sleep duration of both groups within a defined range (see methods for details) and rated participants' sleepiness with the Karolinska Sleepiness Scale before each test session. All participants had at least 8 h of sleep before each session (see methods). The results of ANOVA showed no significant interaction of chronotype×daytime (F_1=1.03, p=0.325) or main effects of chronotype and daytime. This indicates that there was no significant difference between the sleepiness ratings in or between both groups across different times of day. Furthermore, we analyzed resting EEG theta oscillations, which is a marker of sleep pressure[12] in both groups in the morning and evening and found no significant differences in the theta oscillations when we compared both groups in the morning and evening. No significant differences were neither observed in each group across different times of the day. The same pattern of results was observed for alpha oscillations (supplementary materials).

2. The reproducibility of the positioning of the equipment across session is unclear. Most TMS systems come nom with neuronavigation which can guarantee high reproducibility of the TMS coil position across sessions. It seems that such a system was not used here such it is not possible to assess this aspect.

RESPONSE: Thank you for the comment. We acknowledge the reviewer's concern and added this as one of the limitations of our work [please see page 25, highlighted in yellow]. We do not use neuronvigation in our lab when the target region is the motor cortex, but use the physiological criterion of hot spot determination in accordance with many other groups (e.g. Pascual-Leone group: Pascual-Leone et

al. 1994, *Brain*, Pascual-Leone et al. 2005, *Annual Rev Neuroscience*, Rossi et al. 2009, *Clin Neurophysiol,* Rossi et al. 2015, *Clin Neurophysiol*; Ulf Ziemann group: Ulf Ziemann et al. 2009, *Brain Stimul*, Permoli et al. 2017, *Neuroimage*; Leonard G Cohen group: Bütefisch et al. 2000, *PNAS*, Dayan et al. 2018, *Brain Structure & Function*, Joseph Classen group: Stefan et al. 200, *Brain*, other relevant papers: Muellbacher et al. 2002, *Nature*). As described in the methods for coil positioning, we mark the coil position with a waterproof pen and make sure the coil position and angle are kept constant, which is an established procedure used by most expert groups in the field. The hotspot was identified in each session and quite stable between sessions, which is supported by identical stimulation intensities required to evoke the target MEP size between conditions. Specifically, there was no significant difference between the baseline MEPs (absolute values) as well as the %MSO (maximum stimulator output required for the SI1mV MEP amplitude and RMT values) between sessions and conditions, which supports the reliability of this approach. Afterall, if slight alterations of coil positions occurred between sessions, this would add variance, but follow no systematic trend and thus would not affect the results systematically. Whether in this case neuronavigation, which cannot take into account slight changes of motor maps between sessions, would be advantageous is unclear, because it shifts the neurophysiological criterion to a neuroanatomical criterion, which is not more precise. We hope that by making these points and mentioning the lack of neuronavigation as a limitation, the reviewer's concern is resolved.

Revised text, page 25:

Furthermore, although there was no significant difference between baseline MEPs and % of MSO across conditions in both groups, which supports the reliability of the acquired data, the use of neuronavigation for stimulation of the motor cortex might have been advantageous to further enhance reproducibility of the TMS coil position.

Revised text, supplementary materials:

Table S1: Baseline measurements of the cortical excitability protocols

Protocol	measurement	daytime	Group	
			ECs	LCs
Single-pulse MEP	SI 1_{mV} (%)	morning	51.31 ± 9.82	50.43 ± 8.46
		evening	50.50 ± 10.19	50.87 ± 7.91
RMT	%MSO	morning	42.87 ± 7.54	41.81 ± 6.88
		evening	41.68 ± 7.15	42.12 ± 7.09
AMT	%MSO	morning	35.18 ± 6.95	34.40 ± 8.23
		evening	33.53 ± 6.97	34.96 ± 8.18
I-O curve	MEP amplitude at RMT	morning	0.235 ± 0.213	0.197 ± 0.204
		evening	0.149 ± 0.091	0.200 ± 0.133
SICI-ICF	Single-pulse MEP	morning	1.073 ± 0.335	0.941 ± 0.152
		evening	0.910 ± 0.342	0.980 ± 0.121
I-wave facilitation	Single-pulse MEP	morning	1.020 ± 0.341	1.041± 0.119
		evening	1.237 ± 0.512	1.178 ± 0.438
SAI	Single-pulse MEP	morning	1.237 ± 0.512	1.178 ± 0.438
		evening	1.194 ± 0.269	1.195 ± 0.175

Note: MEP = motor-evoked potential; ECs = early chronotypes; LCs = late chronotypes; SI_{1mv} (%) = maximum stimulator output (%MSO) required for the SI1mV MEP amplitude; I-O curve = input-output curve; SICI-ICF = short latency intracortical inhibition and facilitation; SAI = short-latency afferent inhibition; RMT = resting motor threshold; AMT = Active motor threshold.

Table S2: Results of 2 × 2 ANOVAs for control MEPs (RMT intensity MEP for I-O curve, single-pulse MEP for other protocols), SI1mV, and %MSO for RMT/AMT.

Protocol	Measurement	Factor	*df*	*F*	*p*
Single-pulse MEP	SI_{1mV}	daytime	1	0.12	0.724

		chronotype	1	1.40	0.244
		daytime×chronotype	1	0.001	0.938
RMT	%MSO	daytime	1	0.86	0.359
		chronotype	1	0.01	0.901
		daytime×chronotype	1	2.54	0.120
AMT	%MSO	daytime	1	3.23	0.082
		chronotype	1	0.015	0.903
		daytime×chronotype	1	13.30	<**0.001**
I-O curve	MEP amplitude at RMT	daytime	1	1.04	0.315
		chronotype	1	0.02	0.882
		daytime×chronotype	1	1.169	0.288
SICI-ICF	Single-pulse MEP	daytime	1	0.98	0.329
		chronotype	1	0.22	0.641
		daytime×chronotype	1	2.65	0.113
I-wave facilitation	Single-pulse MEP	daytime	1	0.23	0.631
		chronotype	1	0.83	0.367
		daytime×chronotype	1	0.37	0.547
SAI	Single-pulse MEP	daytime	1	0.017	0.895
		chronotype	1	0.112	0.739
		daytime×chronotype	1	0.090	0.765

Note: MEP = motor-evoked potential; chronotype = early (ECs) and late (LCs) chronotypes; SI_{1mv} (%) = maximum stimulator output (%MSO) required for the SI1mV MEP amplitude; I-O curve = input-output curve; SICI-ICF = short latency intracortical inhibition and facilitation; SAI = short-latency afferent inhibition; RMT = resting motor threshold; AMT = active motor threshold.

3. It seems that the statistical model used did not include important covariates such as sex, age and BMI (which is linked overall health status). Statistics need to be recomputed with at least these covariates included. This applies also to correlation analyses between behaviour and neurophysiology which cannot only consist in Pearson's like approach. In addition, although the authors report using mixed repeated measure ANOVA in their statistics they do not state whether subject was the random factor or whether other factors were set as random. Likewise, the type of correlation applied between repeated measures is not specified.

RESPONSE: Thank you for this comment. To answer it, we need to explain some aspects of the design in more detail. We aimed for a relatively homogenous group of participants with respect to the mentioned covariates. We have an **equal number of males and females** in each group (N = 8 of 16, 16 of 32 in total) to prevent potential confounding effects of gender [page 35, highlighted in yellow]. Most participants (25 of 32) were in their 20s (mean age 26.62±5.01) [page 35, highlighted in yellow]. We calculated BMI upon request of the reviewer and the BMI values are also homogenous (mean BMI 23.61±2.63). Of the 32 participants, 26 had a normal BMI (mean BMI 22.61±1.45) and 6 were borderline overweight (mean BMI 27.96±2.11). Please refer to the table below for more details. Furthermore, we computed correlations between these variables (age, sex, BMI) and outcome measures (cortical excitability protocols, neuroplasticity induction, and task performance) [please see below]. There were no significant correlations, which clearly speaks against confounding effects of these variables. We added these details to the revision [please see pages, 7, 11, 17 highlighted in yellow]. Correlation tables are added at the end of this letter (**Appendix 2**). We think with these data there is no need to add these variables as covariates as it makes the results even more complex without adding relevant information. Nevertheless, we can re-run the analyses with ANCOVAs in case the reviewer insists. With regard to the type of correlation, we used Pearson's product-moment correlation (between variables with interval scale) and Point-Biserial correlation in case one variable was dichotomous (e.g., gender). We are open for other types, if the reviewer prefers so (please specify).

Variable		ECs	LCs	*p*
Gender	Male (female)	8 (8)	8 (8)	N/A
Age	Mean±SD	24.56 (4.66)	28.68 (4.60)	0.364
BMI	Mean±SD	23.03 (2.37)	24.20 (2.81)	0.210

Regarding the 2nd point, we think that there is a misunderstanding as far as we understand the reviewer's point. We did not use linear mixed-models (LMM) (that require definition of fixed and random effects) **but** mixed-factorial **ANOVAs** with repeated measures (with between-subject and within-subject factors) for each dataset that have different number of factors. To be specific, we used mixed-factorial ANOVAs with repeated measures (ISI × daytime × chronotype) for the cortical excitability results, a mixed-factorial design with repeated-measures ANOVA (stimulation × timepoint × daytime × chronotype) for the neuroplasticity results, a mixed-factorial ANOVA with repeated-measures (block × daytime × chronotype) for the motor learning, a series of mixed-factorial ANOVAs (daytime × chronotype) for the cognitive task outcome variables, and finally similar mixed-factorial ANOVAs (daytime × chronotype) for the analysis of ERP components. We clarify this in the text as well [please see pages 49-51, highlighted in yellow], and are sorry for the misunderstanding. The last point of the comments (type of correlation for repeated measures) does in our understanding not apply for the same reason because it is for mixed-model GLM (that requires fixed and random effects) **but we** conducted mixed-factorial ANOVAs.

Revised text, page 7, 11, 17:

P. 7: These TMS protocols are based on different neurotransmitter systems related to cortical facilitation (glutamatergic) and inhibition (GABAergic, cholinergic)[23-25] (see Methods). No correlation was found between age, gender, BMI, and MEPs obtained from TMS protocols and thus these variables were not entered as covariates in the ANOVA analyses.

P.11: Side effects were minor and did not differ between intervention conditions except for the tingling and burning sensations (Supplementary Tables 4,5). There was no correlation between age, gender, BMI, and TMS-induced MEP and thus these variables were not entered as potential covariates in the ANOVA analyses.

P.17: All participants conducted the 3-back letter (working memory task), Stroop and AX-Continuous Performance tasks (AX-CPT) (attention tasks), during EEG recording at their circadian-preferred and non-preferred times (see Methods). No significant correlations were observed between the major outcome measures of task performance and demographic variables ruling out their potential confounding effects.

OTHER ISSUES

1- Beyond the major issue raised above the rationale for the choice of 8:30AM and 7PM is not provided

RESPONSE: Thank you for the comment. In the revision, we added the rationale for choosing 8:30 AM and 7:00 PM. First, we selected these times based on previous works that compared levels of activity in the early and late chronotypes[4] (please see the figure below) as well as the two-process model of sleep regulation [22]. According to the figure, 8:30 AM and 7:00 PM are roughly the times when both early and late chronotypes have relatively comparable levels of activity. Considering that both groups were moderate early and late chronotype and were required to have at least 8 h of sleep, their level of activity was even more comparable. Furthermore, in these times there is no (8:30 AM) or less (19:00) sleep pressure according two-process model of sleep regulation. The body hormonal changes that are related to sleep pressures (e.g., melatonin) also usually start to secret after 8-9:00 PM[26]. Lastly, the other rationale for choosing 8-8:30 for morning sessions was to generalize findings to daily life routine schedules (e.g., schooling time, work time). We added these details to the methods [please see page 35, highlighted in blue and yellow].

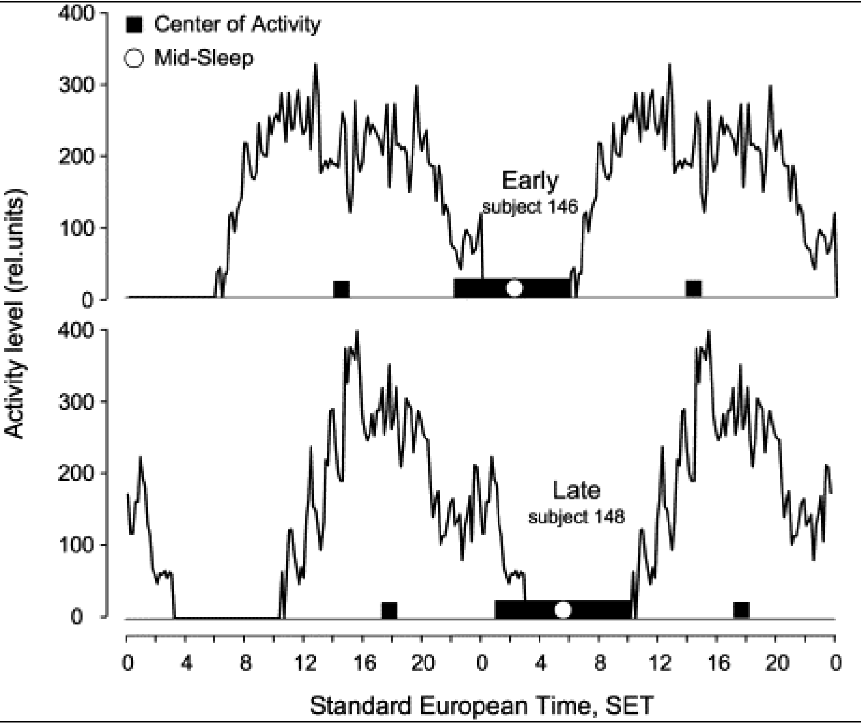

Fig. 3: Comparison of Sleep Times and Activity Profiles between early and late Chronotypes. (Kantermann et al., 2007, Curr Biol).

Revised text, page 35:

Experiment timing, sleep/wake cycle, and light variation

Timing of the experiments was determined based on the average of sleep times and level of activity of early and late chronotypes based on previous studies [4] as well as daily life routine schedules. For morning sessions, the start time was chosen based on the norm start of schooling and working (8:00 in the morning). This is also a time when both chronotypes have the lowest levels of activity [4]. The evening time (19:00) was specified based on the time when both chronotypes have a comparable level of activity according to previous dataset profiles [4]. Both groups were instructed to go to bed (around 22:00-23:00), and get up in the morning at identical times of the day, and have at least 8 hours of sleep for morning sessions ($mean_{ECs}$ = 8.41, $mean_{LCs}$ = 8.11). For the evening session, participants were allowed to go to bed at their preferred time but before 24:00 and were also allowed to have more than 8 hours of sleep if needed ($mean_{ECs}$ = 8.36, $mean_{LCs}$ = 8.38). All participants were strongly recommended to comply with the sleep/wake cycle instruction and in case of irregularities, poor quality of sleep, and sleepiness (measured by participants' rating before each session), the experimental session was postponed to another day. Subjective sleepiness of participants and their alertness were evaluated with the Karolinska sleepiness scale (KSS)[5] which measures sleepiness in a 1-10 Likert type scale (supplementary materials). No significant difference was observed between the sleepiness ratings of both early and late chronotypes in the morning sessions ($mean_{ECs}$ = 3.2, $mean_{LCs}$ = 3.71; t = 1.61, p = 0.11) and evening sessions ($mean_{ECs}$ = 3.58, $mean_{LCs}$ = 3.65; t = 0.25, p = 0.80).

2- The reference list should mention the original Horne and Ostberg publication for chronotype assessment. It'll be much clearer to the reader that may take the questionnaire as the Munich Chronotype Questionnaire.

RESPONSE: Respective reference is added [please see below].

Revised text, page 34:

The German version of the MEQ[27] (DMEQ)[10] was used to identify chronotypes.

3- The type of power mag stimulator needs to be specified

RESPONSE: Respective information is added [please see p. 42, highlighted in yellow]

Revised text, page 42:

Each cortical excitability session took 60-70 min. All TMS protocols were conducted with a PowerMag lab 30 magnetic stimulator (Mag & More, Munich, Germany) through a figure-of-eight magnetic coil (diameter of one winding, 70 mm; peak magnetic field, 2T) held 45° to the midline and applied over the left primary motor cortex

4- Ambient light levels during the experiments need to be specified as light may directly impact alertness and therefore potentially basic brain function.

RESPONSE: Respective information is added [please see below]

Revised text, page 38:

Furthermore, ambient light was kept constant (around 500 lux) across all experimental sessions in the morning and evening. In the cognitive sessions, tasks and EEG recordings were conducted in a sound-proof shielded room with constant temperature of 24-25°C for morning and evening sessions.

5- The percentage of hits included in the 3-back task has be provided. In addition, accuracy is not fully appropriate for n-back performance assessment. D-prime is more appropriate to take into account the unbalanced repartition of target and non-target items.

RESPONSE: Thank you for these suggestions. We added the percentage of hits for the 3-back task [please see p. 17 highlighted in yellow]. We also calculated D-prime and added the results to the main text [page 18, highlighted in yellow], Table 3 [please see p.66-67, highlighted in yellow] and updated Fig 5. accordingly. D prime follows the same pattern and shows an interaction with chronotype and time of day

Revised text, page 17-18:

For working memory performance, the ANOVA results revealed a significant chronotype×daytime interaction (F_1=11.70, p=0.002; ηp^2=0.28) for the N-back hits, which is the primary outcome of interest in this task (Table 2). Post *hoc* Student's t-tests showed a significantly enhanced WM performance in the morning for ECs and evening for LCs (Fig. 5a). The percentage of hits for ECs in the morning and evening was 65.39% and 57.15% respectively and for LCs in the evening and morning it was 72.71% and 62.65 respectively. The same response pattern was observed for the electrophysiological correlates of N-back task performance. Additionally, we calculated the sensitivity index *d* (or d prime) which represents the proportion of hit rates minus the proportion of false alarm rates. A significant interaction of chronotype×daytime (F_1=12.46, p<0.001; ηp^2=0.29) was found with no main effect of chronotype or daytime (Table 2). Post *hoc* Student's t-tests showed a significantly enhanced d prime index in the morning for ECs and evening for LCs (Fig. 5a).

6- Accuracy is not a standard outcome measure of the Stroop task but rather RT variability between congruent and incongruent trials. The author should focus on the latter.

RESPONSE: Thank you for the comment. Our primary focus was indeed on RT and we briefly mentioned accuracy. We emphasized more on this by adding RT variability as suggested. We analyzed this parameter across participants and results follow the same pattern (i,e. less variability at the preferred time in both groups). The results are added to the results section and Table 2 [please see page 18-19 highlighted in yellow].

Revised text, page 18-19:

For Stroop task performance, a similar trend of response was observed. We found a significant chronotype×daytime interaction (F_1=20.84, p=0.001; ηp^2=0.41) on overall RT, RT of congruent trials (F_1=14.36, p=0.001; ηp^2=0.32) and RT of incongruent trials (F_1=23.08, p=0.001; ηp^2=0.43). Congruency had a significant effect, but did not significantly interact with either daytime or chronotype. The main effects of daytime and chronotype were significant neither (Fig. 5). Post *hoc* comparisons of RTs revealed a significantly less Stroop effect (faster RT to incongruent trials) at the circadian-preferred time in both groups (Fig. 5d). This indicates less Stroop interference in participants when they were conducting the task at their circadian-preferred time. The same pattern of results was observed for RT variability in the Stroop block. The results of the ANOVA showed a significant interaction of chronotype×daytime (F_1=25.60, p<0.001; ηp^2=0.46) and post *hoc* comparisons revealed a significantly

reduced variability of RT in the Stroop block at the circadian-preferred time for both groups (ECs: t=2.81, p=0.013; LCs: t=4.62, p<0.001). Performance accuracy was not significantly affected by chronotype. The N200 and N450 are two prominent ERP markers related to Stroop conflict, and are observed at frontocentral and centroparietal regions[28, 29]. The less Stroop effect we observed at the circadian-preferred times was associated with higher N200 and N450 amplitudes which are indicative of higher selective attention and discriminating conflicting stimuli. The results of ANOVA for the N200 amplitudes showed a significant interaction of chronotype×daytime on overall (F_1=17.93, p=0.001; ηp^2=0.30), congruent (F_1=19.04, p<0.001; ηp^2=0.39) and incongruent (F_1=8.66, p=0.006; ηp^2=0.23) trials for the electrodes Fz and Cz. The N200 component of both congruent and incongruent trials over the Fz electrode was larger at the circadian preferred times in both groups (Fig. 5e,f), but the respective difference was significant only in ECs (incongruent: mean±$SEM_{morning}$, -0.38±0.31μV, mean±$SEM_{evening}$, 0.33±0.36μV; congruent: mean±$SEM_{morning}$, -0.59±0.29μV, mean±$SEM_{evening}$, 0.41±0.38μV). However, when we compared the amplitude difference values from morning to evening, chronotype had a significant effect (incongruent: F_1=7.57, p=0.010; ηp^2=0.21; congruent: F_1=14.49, p=0.001; ηp^2=0.33) yielding higher negativity of N200 at circadian-preferred times in both groups. The same pattern of response was found for the Cz electrode and the N450 component (Fig. S4a,b, Supplementary material).

1. Reis, J., et al., *Consensus: Can transcranial direct current stimulation and transcranial magnetic stimulation enhance motor learning and memory formation?* Brain Stimulation, 2008. **1**(4): p. 363-369.
2. Minarik, T., et al., *The Importance of Sample Size for Reproducibility of tDCS Effects.* Frontiers in Human Neuroscience, 2016. **10**(453).
3. Smith, M.J., et al., *Menstrual cycle effects on cortical excitability.* Neurology, 1999. **53**(9): p. 2069-2069.
4. Kantermann, T., et al., *The Human Circadian Clock's Seasonal Adjustment Is Disrupted by Daylight Saving Time.* Current Biology, 2007. **17**(22): p. 1996-2000.
5. Akerstedt, T. and M. Gillberg, *Subjective and objective sleepiness in the active individual.* Int J Neurosci, 1990. **52**(1-2): p. 29-37.
6. Huang, Y.-Z., et al., *Plasticity induced by non-invasive transcranial brain stimulation: A position paper.* Clinical Neurophysiology, 2017. **128**(11): p. 2318-2329.
7. Ridding, M. and U. Ziemann, *Determinants of the induction of cortical plasticity by non-invasive brain stimulation in healthy subjects.* The Journal of physiology, 2010. **588**(13): p. 2291-2304.
8. Rosenberg, J., et al., *"Early to bed, early to rise": Diffusion tensor imaging identifies chronotype-specificity.* NeuroImage, 2014. **84**: p. 428-434.
9. Ambrus, G.G., et al., *The fade-in – Short stimulation – Fade out approach to sham tDCS – Reliable at 1 mA for naïve and experienced subjects, but not investigators.* Brain Stimulation, 2012. **5**(4): p. 499-504.
10. Griefahn, B., et al., *Zur Validität der deutschen Übersetzung des Morningness-Eveningness-Questionnaires von Horne und Östberg.* Somnologie - Schlafforschung und Schlafmedizin, 2001. **5**(2): p. 71-80.
11. Jones, S.E., et al., *Genome-wide association analyses of chronotype in 697,828 individuals provides insights into circadian rhythms.* Nature Communications, 2019. **10**(1): p. 343.
12. Kuhn, M., et al., *Sleep recalibrates homeostatic and associative synaptic plasticity in the human cortex.* Nature Communications, 2016. **7**: p. 12455.
13. Stagg, C.J., A. Antal, and M.A. Nitsche, *Physiology of Transcranial Direct Current Stimulation.* The Journal of ECT, 2018. **34**(3): p. 144-152.
14. Kronberg, G., et al., *Direct current stimulation boosts hebbian plasticity in vitro.* Brain Stimulation, 2019.
15. Nitsche, M.A., et al., *Pharmacological Modulation of Cortical Excitability Shifts Induced by Transcranial Direct Current Stimulation in Humans.* The Journal of Physiology, 2003. **553**(1): p. 293-301.
16. Nitsche, M.A., et al., *Facilitation of Implicit Motor Learning by Weak Transcranial Direct Current Stimulation of the Primary Motor Cortex in the Human.* Journal of Cognitive Neuroscience, 2003. **15**(4): p. 619-626.
17. Rüsseler, J., et al., *Differences in incidental and intentional learning of sensorimotor sequences as revealed by event-related brain potentials.* Cognitive Brain Research, 2003. **15**(2): p. 116-126.

18. Rüsseler, J., T.F. Münte, and D. Wiswede, *On the influence of informational content and key-response effect mapping on implicit learning and error monitoring in the serial reaction time (SRT) task.* Experimental Brain Research, 2018. **236**(1): p. 259-273.
19. Borbély, A.A., et al., *The two-process model of sleep regulation: a reappraisal.* Journal of Sleep Research, 2016. **25**(2): p. 131-143.
20. Schmidt, C., et al., *A time to think: circadian rhythms in human cognition.* Cognitive neuropsychology, 2007. **24**(7): p. 755-789.
21. Horne, J.A. and O. Ostberg, *A self-assessment questionnaire to determine morningness-eveningness in human circadian rhythms.* Int J Chronobiol, 1976. **4**(2): p. 97-110.
22. Borbély, A.A., *A two process model of sleep regulation.* Hum neurobiol, 1982. **1**(3): p. 195-204.
23. Di Lazzaro, V., et al., *Muscarinic receptor blockade has differential effects on the excitability of intracortical circuits in the human motor cortex.* Experimental Brain Research, 2000. **135**(4): p. 455-461.
24. Chen, R., *Studies of human motor physiology with transcranial magnetic stimulation.* Muscle & Nerve, 2000. **23**(S9): p. S26-S32.
25. Di Lazzaro, V., et al., *Dissociated effects of diazepam and lorazepam on short-latency afferent inhibition.* The Journal of Physiology, 2005. **569**(1): p. 315-323.
26. Masri, S. and P. Sassone-Corsi, *The emerging link between cancer, metabolism, and circadian rhythms.* Nature Medicine, 2018. **24**(12): p. 1795-1803.
27. Horne, J.A. and O. Östberg, *A self-assessment questionnaire to determine morningness-eveningness in human circadian rhythms.* International Journal of Chronobiology, 1976. **4**: p. 97-110.
28. Hanslmayr, S., et al., *The Electrophysiological Dynamics of Interference during the Stroop Task.* Journal of Cognitive Neuroscience, 2008. **20**(2): p. 215-225.
29. Feroz, F.S., et al., *The Time Course of Activity within the Dorsal and Rostral-Ventral Anterior Cingulate Cortex in the Emotional Stroop Task.* Brain Topography, 2017. **30**(1): p. 30-45.

Appendix 1

Correlation between DMEQ score and neuroplasticity

Early chronotypes		DMEQ	SICI_inhibition_Mo	SICI_facilitation_Mo	SICI_inhibition_eve	SICI_facilitation_eve	SAI_Mo	SAI_eve	IWAVE_MO	IWAVE_EVE	IO_MO	IO_Eve
DMEQ	Pearson Correlation	1	.658**	.379	.259	.152	.101	.080	.204	-.021	.349	.436
	Sig. (2-tailed)		.006	.148	.332	.575	.711	.767	.449	.937	.186	.091
	N	16	16	16	16	16	16	16	16	16	16	16

Late chronotypes		DMEQ	SICI_inhibition_Mo	SICI_facilitation_Mo	SICI_inhibition_eve	SICI_facilitation_eve	SAI_Mo	SAI_eve	IWAVE_MO	IWAVE_EVE	IO_MO	IO_Eve
DMEQ	Pearson Correlation	1	-.514*	.204	-.313	.348	-.448	-.533*	-.130	-.127	.029	-.108
	Sig. (2-tailed)		.042	.449	.237	.187	.082	.033	.632	.639	.916	.691
	N	16	16	16	16	16	16	16	16	16	16	16

Correlation between DMEQ score and neuroplasticity

Early chronotypes		DMEQ1	anodal_mo	cathodal_mo	anodal_eve	cathodal_eve	anodal_mo_0_20	cathodal_mo_0_20	anodal_eve_0_20	cathodal_eve_0_20
DMEQ	Pearson Correlation	1	.037	.446	.094	-.042	.031	.491	-.020	-.018
	Sig. (2-tailed)		.891	.084	.730	.879	.909	.053	.941	.946
	N	16	16	16	16	16	16	16	16	16

Late chronotypes		DMEQ1	anodal_mo	cathodal_mo	anodal_eve	cathodal_eve	anodal_mo_0_20	cathodal_mo_0_20	anodal_eve_0_20	cathodal_eve_0_20
DMEQ1	Pearson Correlation	1	-.276	.116	-.322	.144	-.292	.144	-.357	.132
	Sig. (2-tailed)		.300	.670	.224	.595	.272	.594	.175	.625
	N	16	16	16	16	16	16	16	16	16

Correlation between DMEQ score and behavioral performance

Early chronotypes		DMEQ	nback_hit_mo	nback_hit_eve	nback_RT_mo	nback_RT_eve	CW_Acc_MO	RT_MOstroop	CW_Acc_Eve	RT_Eve_stroop	AXCPT_ACC_Mo	AXCPT_RT_Mo	AXCPT_ACC_eve	AXCPT_RT_eve
DMEQ	Pearson Correlation	1	.092	.161	.573*	.239	-.378	-.076	-.541*	-.330	-.020	.048	-.388	.084
	Sig. (2-tailed)		.734	.552	.020	.373	.149	.779	.031	.213	.940	.861	.137	.756
	N	16	16	16	16	16	16	16	16	16	16	16	16	16

Late chronotypes		DMEQ	nback_hit_mo	nback_hit_eve	nback_RT_mo	nback_RT_eve	CW_Acc_MO	RT_MO_stroop_	CW_Acc_Eve	RT_Eve_stroop_	AXCPT_ACC_MO	AXCPT_RT_MO	AXCPT_ACC_eve	AXCPT_RT_eve
DMEQ	Pearson Correlation	1	.029	.167	.409	.376	.464	-.171	.586*	-.094	-.264	.178	.270	-.024
	Sig. (2-tailed)		.915	.536	.116	.151	.070	.527	.017	.731	.324	.510	.311	.930
	N	16	16	16	16	16	16	16	16	16	16	16	16	16

Appendix 2

Correlation between demographics and neuroplasticity

Early chronotypes		anodal_mo	cathodal_mo	anodal_mo_0_20	cathodal_mo_0_20	anodal_eve	cathodal_eve	anodal_eve_0_20	cathodal_eve_0_20
Age	Pearson Correlation	-.180	-.073	-.249	-.100	.185	-.054	.145	-.032
	Sig. (2-tailed)	.505	.788	.352	.713	.492	.844	.591	.908
	N	16	16	16	16	16	16	16	16
Gender	Pearson Correlation	.065	-.162	.091	-.062	-.262	-.116	-.252	-.060
	Sig. (2-tailed)	.812	.548	.737	.819	.327	.669	.346	.824
	N	16	16	16	16	16	16	16	16
BMI	Pearson Correlation	-.027	.414	.002	.292	.321	-.448	.304	-.469
	Sig. (2-tailed)	.920	.111	.994	.272	.225	.082	.253	.067
	N	16	16	16	16	16	16	16	16

Late chronotypes		anodal_mo	cathodal_mo	anodal_mo_0_20	cathodal_mo_0_20	anodal_eve	cathodal_eve	anodal_eve_0_20	cathodal_eve_0_20
Age	Pearson Correlation	.095	.294	.122	.198	.110	-.245	.114	-.217
	Sig. (2-tailed)	.726	.268	.653	.463	.686	.360	.673	.421
	N	16	16	16	16	16	16	16	16
Gender	Pearson Correlation	.137	.104	.082	.093	.230	.046	.252	-.012
	Sig. (2-tailed)	.613	.700	.762	.733	.391	.865	.347	.966
	N	16	16	16	16	16	16	16	16
BMI	Pearson Correlation	.236	.268	.240	.154	.412	-.035	.364	.011
	Sig. (2-tailed)	.379	.316	.371	.569	.113	.896	.165	.968
	N	16	16	16	16	16	16	16	16

Correlation between demographics and cortical excitability

Early chronotypes		SICI_inhibition_Mo	SICI_facilitation_Mo	SICI_inhibition_eve	SICI_facilitation_eve	SAI_Mo	SAI_eve	IWAVE_MO	IWAVE_EVE	IO_MO	IO_Eve
Age	Pearson Correlation	-.145	.070	-.177	.190	.247	.032	.009	-.258	-.125	-.026
	Sig. (2-tailed)	.592	.797	.513	.482	.357	.905	.974	.335	.645	.925
	N	16	16	16	16	16	16	16	16	16	16
Gender	Pearson Correlation	-.018	.153	-.025	-.150	-.302	-.070	.113	-.185	.111	.193
	Sig. (2-tailed)	.947	.571	.926	.580	.255	.796	.676	.492	.681	.474
	N	16	16	16	16	16	16	16	16	16	16
BMI	Pearson Correlation	.364	-.212	.111	-.025	.263	-.215	.402	-.211	.420	.186
	Sig. (2-tailed)	.166	.430	.681	.926	.325	.425	.122	.434	.106	.491
	N	16	16	16	16	16	16	16	16	16	16

Late chronotypes		SICI_inhibition_Mo	SICI_facilitation_Mo	SICI_inhibition_eve	SICI_facilitation_eve	SAI_Mo	SAI_eve	IWAVE_MO	IWAVE_EVE	IO_MO	IO_Eve
Age	Pearson Correlation	-.216	.104	-.138	.370	-.242	.058	-.021	.269	-.323	-.158
	Sig. (2-tailed)	.422	.703	.611	.158	.366	.832	.939	.314	.223	.559
	N	16	16	16	16	16	16	16	16	16	16
Gender	Pearson Correlation	-.017	-.360	.085	-.340	.461	.409	-.331	-.414	-.280	-.122
	Sig. (2-tailed)	.950	.171	.753	.198	.072	.116	.211	.110	.293	.652
	N	16	16	16	16	16	16	16	16	16	16
BMI	Pearson Correlation	-.110	-.058	-.254	-.068	.117	.394	-.385	.014	-.392	.132
	Sig. (2-tailed)	.684	.830	.343	.802	.667	.131	.141	.960	.133	.625
	N	16	16	16	16	16	16	16	16	16	16

Correlation between demographics and cognitive performance

Early chronotypes		nback_hit_mo	nback_hit_eve	nback_RT_mo	nback_RT_eve	CW_RT_MO_stroop_effect	CW_RT_Eve_stroop_effect	AXCPT_ACC_MO	AXCPT_RT_MO	AXCPT_ACC_eve	AXCPT_RT_eve
Age	Pearson Correlation	-.497	-.395	.459	.141	.445	.291	-.281	.375	.392	-.488
	Sig. (2-tailed)	.050	.130	.074	.602	.084	.275	.291	.152	.133	.055
	N	16	16	16	16	16	16	16	16	16	16
Gender	Pearson Correlation	.361	.147	-.703**	-.353	-.377	-.090	-.183	-.117	-.232	-.017
	Sig. (2-tailed)	.169	.586	.002	.180	.149	.740	.497	.665	.388	.949
	N	16	16	16	16	16	16	16	16	16	16
BMI	Pearson Correlation	-.534*	-.269	.376	.153	.413	.373	.248	.146	.059	-.209
	Sig. (2-tailed)	.033	.314	.151	.571	.112	.155	.355	.590	.827	.438
	N	16	16	16	16	16	16	16	16	16	16

Late chronotypes		nback_hit_mo	nback_hit_eve	nback_RT_mo	nback_RT_eve	CW_RT_MO_stroop_effect	CW_RT_Eve_stroop_effect	AXCPT_ACC_MO	AXCPT_RT_MO	AXCPT_ACC_eve	AXCPT_RT_eve
Age	Pearson Correlation	.040	.187	-.129	-.010	.002	.077	.051	-.191	-.123	-.079
	Sig. (2-tailed)	.883	.487	.635	.970	.994	.778	.851	.479	.651	.771
	N	16	16	16	16	16	16	16	16	16	16
Gender	Pearson Correlation	-.224	-.292	-.093	.170	-.171	.058	-.151	-.129	-.072	-.243
	Sig. (2-tailed)	.404	.273	.732	.530	.526	.830	.578	.635	.790	.365
	N	16	16	16	16	16	16	16	16	16	16
BMI	Pearson Correlation	-.222	-.117	-.066	-.010	-.016	-.025	-.010	-.222	-.368	-.257
	Sig. (2-tailed)	.409	.666	.808	.971	.954	.927	.971	.409	.160	.336
	N	16	16	16	16	16	16	16	16	16	16

Reviewers' comments:

Reviewer #2 (Remarks to the Author):

This continues to look good to me.

Reviewer #3 (Remarks to the Author):

I was asked by the editor to assess whether the arguments provided by the authors would resolve the issue I had in my previous reviews. While I appreciate the efforts put in by the authors to appeal the editor decision, all I can state is that, for me, the issues remain the same.
The way the authors address the issues I raised highlights a fundamental misunderstanding about the regulation of sleep and wakefulness: the interaction between sleep homeostasis and circadian rhythmicity is non-linear. This means that even if markers of one process are the same (e.g. sleep need, as assessed by wake EEG measures – which is not a measure of sleep homeostasis alone as it is influenced by the circadian system), the output of the interaction (e.g. activity/response of a given brain area or behaviour) may be very different if the other process is not the same (e.g. circadian phase, which was not assessed in the present study). In addition, the output of the interaction (e.g. activity/response of a given brain area or behaviour) maybe the same despite very different status of both processes. It is therefore not surprising that theta activity does not vary from morning to evening time points of the protocol as it is subjected to the influence of both sleep homeostasis and circadian processes (as reported many times in the literature).
Author response to issue #2 highlights an additional important issue with the protocol and another misunderstanding of sleep-wake regulation: the imposed 8h sleep wake schedule was only requested for one 1 night. In addition to inducing likely partial sleep loss, this procedure cannot guarantee proper circadian entrainment, particularly in late chronotypes.
Most of my arguments are the same in than in the previous review. I will just briefly add the following.
As previously stated, subjective sleepiness values are most appropriate to assess relative changes not to compare group of subjects. One can get used to higher sleep pressure and therefore rate sleepiness lower that some else for the same sleep pressure.
Resting state EEG data were taken into account in the previous evaluation of the study.
The new statistical results with more covariates resolve the particular issue I had there (minor comment).

Authors' Response to the Review Comments

Journal: Nature Communications

Manuscript #: NCOMMS-20-13170-T

Reviewer #3 (Remarks to the Author):

I was asked by the editor to assess whether the arguments provided by the authors would resolve the issue I had in my previous reviews. While I appreciate the efforts put in by the authors to appeal the editor decision, all I can state is that, for me, the issues remain the same.
The way the authors address the issues I raised highlights a fundamental misunderstanding about the regulation of sleep and wakefulness: the interaction between sleep homeostasis and circadian rhythmicity is non-linear. This means that even if markers of one process are the same (e.g. sleep need, as assessed by wake EEG measures – which is not a measure of sleep homeostasis alone as it is influenced by the circadian system), the output of the interaction (e.g. activity/response of a given brain area or behaviour) may be very different if the other process is not the same (e.g. circadian phase, which was not assessed in the present study). In addition, the output of the interaction (e.g. activity/response of a given brain area or behaviour) maybe the same despite very different status of both processes. It is therefore not surprising that theta activity does not vary from morning to evening time points of the protocol as it is subjected to the influence of both sleep homeostasis
and circadian processes (as reported many times in the literature).
Author response to issue #2 highlights an additional important issue with the protocol and another misunderstanding of sleep-wake regulation: the imposed 8h sleep wake schedule was only requested for one 1 night. In addition to inducing likely partial sleep loss, this procedure cannot guarantee proper circadian entrainment, particularly in late chronotypes.
Most of my arguments are the same in than in the previous review. I will just briefly add the following.
As previously stated, subjective sleepiness values are most appropriate to assess relative changes not to compare group of subjects. One can get used to higher sleep pressure and therefore rate sleepiness lower that some else for the same sleep pressure.
Resting state EEG data were taken into account in the previous evaluation of the study.
The new statistical results with more covariates resolve the particular issue I had there (minor comment).

Response: Thank you for providing more details on the previous comments. We agree that we cannot completely rule out the confounding effects of sleep pressure. Although both, subjective sleepiness rating and EEG theta/alpha makers showed no difference between groups at the time of experimentation, these measures are indirect measures of sleep pressure.

We also agree that a minimum of 8 h of sleep during the whole experiment could have been more advantageous, although we made sure participants were not under sleep pressure during experimental sessions. Having that said, we still think if sleep pressure had a significant confounding effect it would have been reflected in EEG results, and the results should have been similar to our recent data that show a different pattern on the same variables under sleep pressure [1].

Nevertheless, we now explicitly acknowledge these shortcomings to a larger extent than before in the revision and mention them as limitations as well. Please see the respective changes in the results (page 21) and discussion sections on pages 22-23 (opening paragraph), 27, and 28 (concluding paragraph) highlighted in blue.

Revised text:

Page 21: All participants had moderate chronotypes which reduces the variability of sleep-wake cycle, however, this can interfere with chronotype-specific effects on brain physiology and cognition in case of higher sleep pressure in either group at non-preferred times. We did not directly control for the potential influence of sleep and sleep pressure, but used indirect measures of sleep pressure (e.g., subject sleepiness rating, EEG markers of sleep pressure).

Page 22: Previous studies have shown that chronotype has distinctive behavioural, physical, and genetic manifestations in humans[2]. Here we show converging evidence of how chronotypes and time-of-day are associated with behavioural/cognitive performance of healthy individuals, and demonstrate the physiological foundations of these effects by daytime-dependent cortical excitability, neuroplasticity, and brain information processing processes (Fig. 7a). A specific causal effect of chronotype on these variables, however, cannot be definitely concluded unless the confounding influence of sleep pressure is controlled for directly, which could be done by extended adaptation of sleep times.

Page 27: Our findings, though convergent at different levels, need to be interpreted with care. First, the observed findings cannot be solely attributed to chronotype. There are likely interindividual confounding effects due to sleep pressure that were not accounted for directly. These can be caused by different sleep-wake patterns of early and late chronotypes and the fact that experimental timing was fixed across groups. It is important to note here that our study question was whether brain physiology and cognition differ at different times of day due to chronotype but not different levels of sleep pressure observed in different chronotypes. Therefore, we picked a fixed time of measurement based on sleep-wake cycle and level of activity across groups[3] and did not individually adapt the experimental timing. Second, our measures of sleep pressure (e.g., subjective rating of sleepiness, resting EEG markers) are indirect. Direct and specific measures of sleep parameters and homeostasis (e.g. polysomnography) would be advantageous to reliably account for the confounding effect of sleep pressure. Finally, although we imposed at least 8 h of sleep before each experimental session and instructed participants to comply with this during the experimental course, it would be advantageous to extend this control during and before the course of the experiments to guarantee proper circadian entrainment, particularly in late chronotypes. Having that said, our findings still show a strong association of chronotype with human brain physiology and cognition, and the observed pattern here differs from what is seen under sleep pressure[1].

Page 28: In conclusion, our results show an association of circadian preference with learning and cognition including memory formation and attentional functions as well as brain physiology underlying these cognitive processes including cortical excitability, neuroplasticity, and electrophysiological correlates of cognitive processes. These findings cannot be exclusively attributed to chronotype, but are likely affected by the interaction of chronotype and individual sleep-wake cycle.

Additional expert (Remarks to the Author):

In particular, this expert agrees with Reviewer 3's suggestion that the confounding effects of sleep pressure have not been directly controlled for, given that subjective sleepiness and resting EEG power are indirect measures of sleep quality and duration for the previous night. Since this measure isn't direct, there is likely interindividual noise that is not accounted for, and there may have been more reliable ways to control for the potential influence of sleep and sleep pressure.

Response: We appreciate the comment of the new reviewer and agree that without direct measures of sleep quality and duration, there are likely some confounding noises. We acknowledged and mentioned this in the discussion.

Page 27: Our findings, though convergent at different levels, need to be interpreted with care. First, the observed findings cannot be solely attributed to chronotype. There are likely interindividual confounding effects due to sleep pressure that were not accounted for directly. These can be caused by different sleep-wake patterns of early and late chronotypes and the fact that experimental timing was fixed across groups. It is important to note here that our study question was whether brain physiology and cognition differ at different times of day due to chronotype but not different levels of sleep pressure observed in different chronotypes.

1. Salehinejad, M.A., *et al.* Sleep-dependent upscaled excitability and saturated neuroplasticity in the human brain: From brain physiology to cognition. *bioRxiv*, 2021.2004.2028.441823 (2021).
